# Structure–Activity Relationship (SAR) Study of Spautin-1 to Entail the Discovery of Novel NEK4 Inhibitors

**DOI:** 10.3390/ijms22020635

**Published:** 2021-01-10

**Authors:** Mathias Elsocht, Philippe Giron, Laila Maes, Wim Versées, Gustavo J. Gutierrez, Jacques De Grève, Steven Ballet

**Affiliations:** 1 Research Group of Organic Chemistry, Faculty of Sciences and Bioengineering Sciences, Vrije Universiteit Brussel, Pleinlaan 2, 1050 Brussels, Belgium; Mathias.Elsocht@vub.be; 2 Laboratory of Medical and Molecular Oncology and Center of Medical Genetics, Faculty of Medicine and Pharmacy, Vrije Universiteit Brussel, Laarbeeklaan 103, 1090 Brussels, Belgium; Philippe.Giron@vub.be (P.G.); Jacques.DeGreve@uzbrussel.be (J.D.G.); 3 Laboratory of Pathophysiological Cell Signalling (PACS), Department of Biology, Faculty of Sciences and Bioengineering Sciences, Vrije Universiteit Brussel, Pleinlaan 2, 1050 Brussels, Belgium; Gustavo.Gutierrez.Gonzalez@vub.be; 4 VIB-VUB Center for Structural Biology, Pleinlaan 2, 1050 Brussels, Belgium; Laila.Maes@vub.be (L.M.); Wim.Versees@vub.be (W.V.); 5 Structural Biology Brussels, Vrije Universiteit Brussel, Pleinlaan 2, 1050 Brussels, Belgium

**Keywords:** non-small cell lung cancer, USP13, NEK4, EGFR, quinazolinamines

## Abstract

Lung cancer is one of the most frequently diagnosed cancers accounting for the highest number of cancer-related deaths in the world. Despite significant progress including targeted therapies and immunotherapy, the treatment of advanced lung cancer remains challenging. Targeted therapies are highly efficacious at prolonging life, but not curative. In prior work we have identified Ubiquitin Specific Protease 13 (USP13) as a potential target to significantly enhance the efficacy of mutant EGFR inhibition. The current study aimed to develop lead molecules for the treatment of epidermal growth factor receptor (EGFR)-mutant non-small cell lung cancer (NSCLC) by developing potent USP13 inhibitors initially starting from Spautin-1, the only available USP13 inhibitor. A SAR study was performed which revealed that increasing the chain length between the secondary amine and phenyl group and introducing a halogen capable of inducing a halogen bond at position 4’ of the phenyl group, dramatically increased the activity. However, we could not confirm the binding between Spautin-1 (or its analogues) and USP13 using isothermal titration calorimetry (ITC) or thermal shift assay (TSA) but do not exclude binding under physiological conditions. Nevertheless, we found that the anti-proliferative activity displayed by Spautin-1 towards EGFR-mutant NSCLC cells in vitro was at least partially associated with kinase inhibition. In this work, we present *N*-[2-(substituted-phenyl)ethyl]-6-fluoro-4-quinazolinamines as promising lead compounds for the treatment of NSCLC. These analogues are significantly more effective towards EGFR-mutant NSCLC cells than Spautin-1 and act as potent never in mitosis A related kinase 4 (NEK4) inhibitors (IC_50_~1 µM) with moderate selectivity over other kinases.

## 1. Introduction

**Figure 1:**
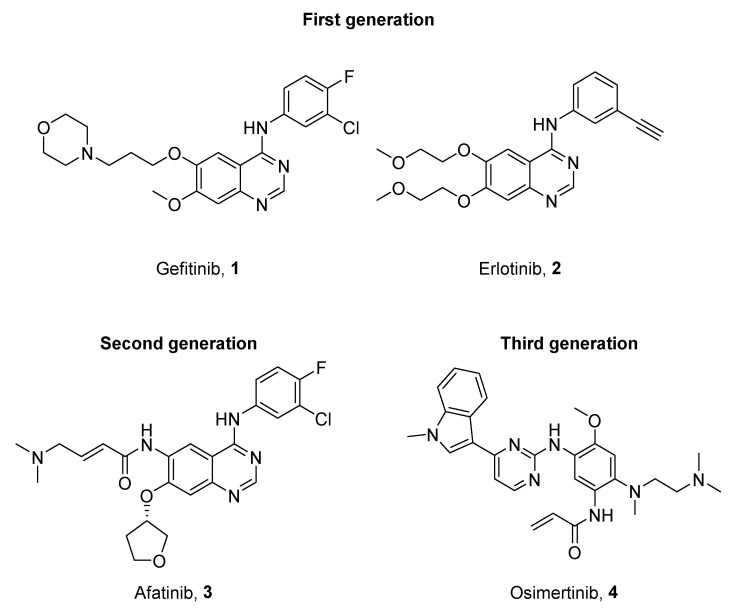
Examples of first, second and third generations epidermal growth factor receptor tyrosine kinase inhibitors.

The World Health Organization estimated that 1.76 million people died of lung cancer in 2018, which represents almost 20% of all cancer-related deaths [1]. The predominant subtype of lung cancer is NSCLC, which accounts for 80–85% of all lung cancer patients [2]. In NSCLC patients with little or no smoking history, mutations in the EGFR, a major regulator of cell proliferation and apoptosis, are frequently observed [3]. The exon 19 in frame deletion (EGFR del E746-A750) and the exon 21 point mutation (L858R) represent together 90% of all activating mutations [4]. Despite the promising initial response of EGFR-mutant NSCLC patients towards first (reversible), second (irreversible), and third (EGFR mutant specific) generations of EGFR tyrosine kinase inhibitors (examples depicted in Figure 1), patients generally developed acquired resistance (by a secondary mutation or activation of alternative pathways within the cancer cells) which illustrates the need for improved treatment strategies in this subset of lung cancers [4,5]. 

The importance of posttranslational modifications in tumorigenesis is widely accepted [6,7,8]. Insight in these processes can lead to new therapeutic strategies to improve current treatments [8]. Ubiquitination is, besides phosphorylation, an important posttranslational modification linked to cancer. It consists of the reversible attachment of one or multiple ubiquitin moieties to target proteins, altering their stability, activity, interactors and/or localization [9,10]. It is a three-step process catalyzed by the consecutive action of the E1 activating enzyme, the E2 conjugating enzyme and the E3 ligase [9,11]. Conversely, deubiquitinating enzymes (DUBs) reverse this process by selectively cleaving ubiquitin or poly-ubiquitin chains from ubiquitinated proteins [12]. 

USP13, a DUB enzyme part of the USP-family, has been linked to multiple cancers. This enzyme acts as a tumor suppressor in breast cancer, human bladder cancer, and oral squamous cell carcinoma [13,14,15]. However, it also acts as a tumor promotor in glioblastoma (brain tumor), ovarian cancer, melanoma (skin cancer), and prostate cancer [16,17,18,19,20,21,22,23]. In NSCLC, USP13 has been frequently found to be amplified. Moreover NSCLC cell proliferation (in vitro) and tumor growth (in vivo) is inhibited by downregulating USP13 [24]. 

**Figure 2:**
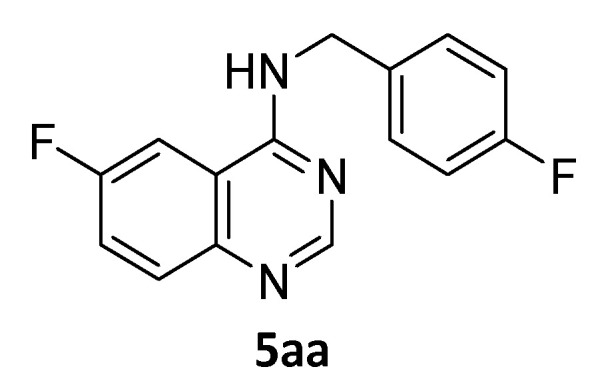
Chemical structure of Spautin-1 (**5aa**).

Despite the differential role of USP13 in tumorigenesis, this enzyme is considered as a potential therapeutic target. Recently, we have reported that inhibition of USP13 destabilizes EGFR and that co-inhibition of USP13 and EGFR suppresses oncogenic signaling in NSCLC cells [25]. These findings motivated us to initiate the development of more potent USP13 inhibitors, starting from the only USP13 inhibitor described in the literature to date (Spautin-1, **5aa**, Figure 2) [26], by exploring the chemical space around Spautin-1 and determining the importance of the quinazoline core by performing a “*N*-screening”. The quinoline [27,28,29,30], isoquinoline [31,32], cinnoline [33,34,35], and quinoxaline [36] cores were considered, all privileged scaffolds which have shown their effectiveness as kinase inhibitors. 

**Figure 3:**
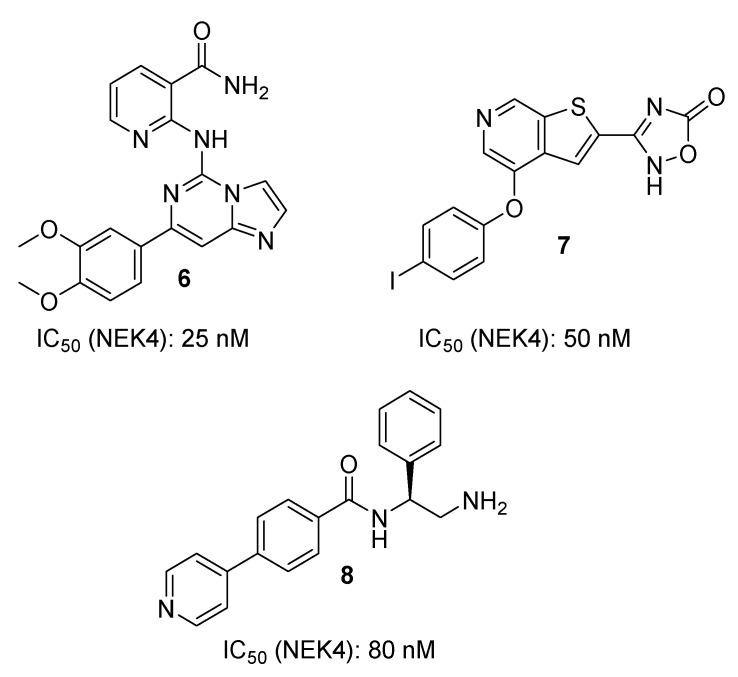
Non-selective NEK4 inhibitors reported in the literature so far.

Herein, we elaborate upon *N*-[2-(substituted-phenyl)ethyl]-6-fluoro-4-quinazolinamines as promising lead compounds for the treatment of NSCLC. Based on an EGFR-mutant NSCLC cell viability screening, these analogues were found to be significantly more potent when compared to Spautin-1. We attempted to demonstrate binding of the spautin-1 analogues to USP13 using isothermal titration calorimetry (ITC) or thermal shift assay (TSA) but were unsuccessful. Nevertheless, the *N*-[2-(substituted-phenyl)ethyl]-6-fluoro-4-quinazolinamines showed good activity and moderate selectivity towards NEK4, a kinase previously linked to NSCLC. Park and co-workers reported, for instance, the overexpression of NEK4 in lung cancer and suggested that NEK 4 suppresses Tumor necrosis factor-Related Apoptosis Inducing Ligand (TRAIL)-induced apoptosis, which in turn results into tumor resistance. In contrast, downregulation of NEK4 sensitizes tumor cells to TRIAL-induced apoptosis via decreased levels of survivin, an anti-apoptotic protein [37]. In 2018, along the same lines, Ding and co-workers published that NEK4 is a positive regulator of epithelial-to-mesenchymal transition (EMT) which plays an important role in lung cancer metastasis [38]. To our best knowledge, there are no potent and selective NEK4 inhibitors described in the literature. Some previously reported kinase inhibitors showed good off-target activity for NEK4 but lacked selectivity. Some examples are: BAY 61-3606 (**6**, SYK inhibitor)**,** Abbott compound 17 (**7**, MAP3K8 inhibitor), and Abbott 1141 (**8**, multikinase inhibitor) (structures depicted in Figure 3) [39,40,41,42]. 

In light of the above, we hypothesize that the activity of our analogues towards EGFR-mutant NSCLC cells is at least in part related to inhibition of NEK4. As far as we know, these are the first inhibitors described in the literature targeting NEK4, which also show promising single agent anticancer activity towards EGFR-mutant NSCLC cells.

## 2. Results

### 2.1. Chemistry

**Scheme 1:**
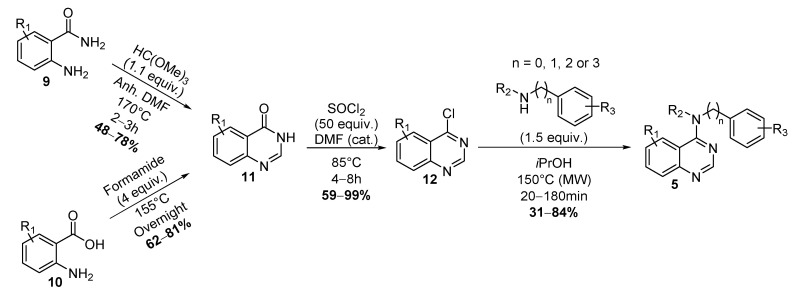
Synthesis of Spautin-1 analogues **5** starting from substituted 2-aminobenzamides **9** or 2-aminobenzoic acids **10**.

The chemical space around Spautin-1 was explored by synthesizing analogues based on the 4-aminoquinazoline core. An efficient three-step protocol was developed (Scheme 1), which only required one purification at the end of the synthesis. The substituted quinazolin-4(3*H*)-ones **11** were synthesized starting from substituted 2-aminobenzamides **9** using trimethyl orthoformate or substituted 2-aminobenzoic acids **10** using formamide [43,44]. Subsequently, the 4-chloroquinazoline core **12** was obtained using thionyl chloride and a catalytic amount of *N*,*N*-dimethylformamide (DMF) [45]. Finally, a microwave-assisted nucleophilic aromatic substitution was performed to obtain the desired analogues **5** in yields varying between 31 and 84% [46].

**Scheme 2:**
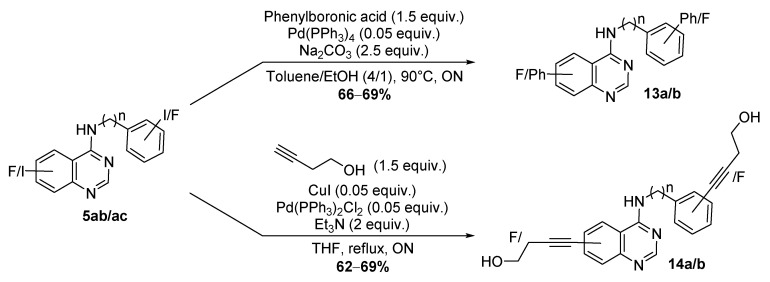
Palladium-catalyzed cross coupling reactions: Suzuki and Sonogashira reactions.

Further exploratory diversification was introduced using palladium-catalyzed cross-coupling reactions. In literature described strategies were applied to introduce a phenyl group as in **13**, using the Suzuki reaction, and an alkyne substituent to reach compounds of type **14**, via the Sonogashira reaction (Scheme 2) [47,48]. These larger substituents provided insight in the available chemical space around the quinazoline core. These conversions made use of the I>>F selectivity during Pd-catalyzed transformations.

**Scheme 3:**
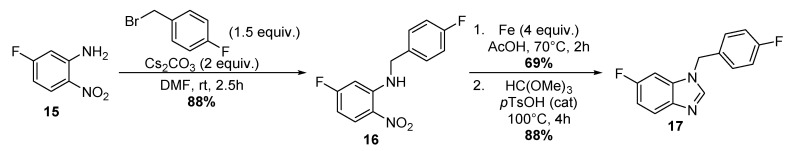
Synthesis of a ring contracted spautin-1 analogue bearing the benzimidazole core.

As a ring-contracted analogue, the synthesis of a Spautin-1 analogue bearing the benzimidazole core **17**, was pursued (Scheme 3). The benzimidazole system is a well-established scaffold in medicinal chemistry, but in the current study, it can be regarded as a quinazoline analogue in which one of both rings is compacted. In consequence, the substituents will be oriented differently as compared to the topological orientation in its homologue. The substituted benzimidazole was synthesized starting from 5-fluoro-2-nitroaniline **15**. In our hands, the highest isolated yield for the nucleophilic substitution reaction towards **16** was obtained in DMF using Cs_2_CO_3_ as a base. Finally, the benzimidazole core was obtained after reduction of the nitro-group using an excess of iron in acetic acid, followed by the cyclisation using trimethyl orthoformate and a catalytic amount of *p*-toluene sulfonic acid yielding **17** [49,50]. 

**Scheme 4:**
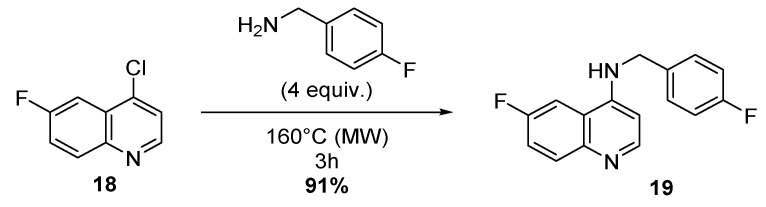
Synthesis of a spautin-1 analogue bearing the quinoline core.

Thereafter, a “*N*-screening” was performed to determine how important the quinazoline scaffold is for the biological activity. First, the quinazoline core was replaced by the quinoline and isoquinoline core to determine the importance of each individual nitrogen. In one step, compound **19** was obtained from the commercially available 4-chloro-6-fluoroquinoline **18** through a microwave-assisted nucleophilic aromatic substitution (Scheme 4). The previously described conditions of Felts et al. were not efficient in this case, because of which we opted for a solvolytic reaction [51]. 

**Scheme 5:**
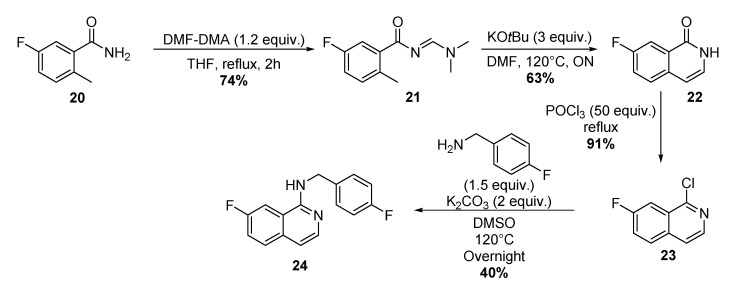
Synthesis of a spautin-1 analogue bearing the isoquinoline core.

The isoquinoline core was obtained following the procedure reported by Zhao and co-workers (Scheme 5) [52]. Starting from 5-fluoro-2-methylbenzamide **20**, 7-fluoro-isoquinolinone **22** was obtained in two steps. First **20** was allowed to react with *N*,*N*-dimethylformamide dimethyl acetal yielding intermediate **21**, which was subsequently cyclized under basic conditions. Next, 1-chloro-7-fluoroisoquinoline **23** was synthesized using phosphorus oxychloride, which was subsequently converted to the Spautin-1 analogue **24** via a nucleophilic aromatic substitution. 

**Scheme 6:**
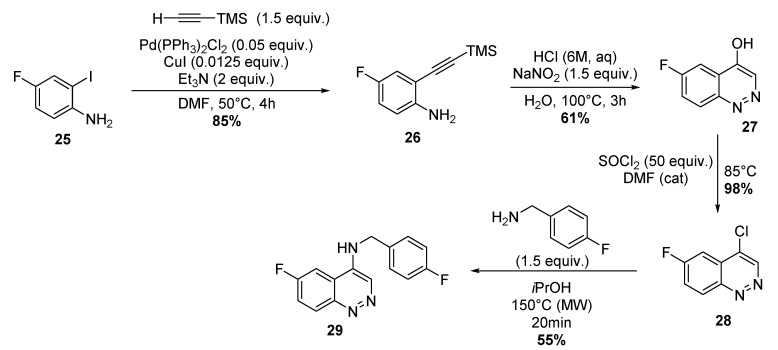
Synthesis of a Spautin-1 analogue bearing the cinnoline core.

Alternatively, the two nitrogens in the central scaffold were preserved but positioned differently. The cinnoline core was accessed via a Sonogashira coupling, followed by a cyclisation through in situ formation of nitrous acid (Scheme 6) [53]. The obtained 6-fluorocinnoline-4-ol **27** was then converted to 4-chloro-6-fluorocinnoline **28** using thionyl chloride in the presence of a catalytic amount of DMF. Finally, a nucleophilic aromatic substitution was performed yielding the desired product **29** in moderate yield. 

**Scheme 7:**
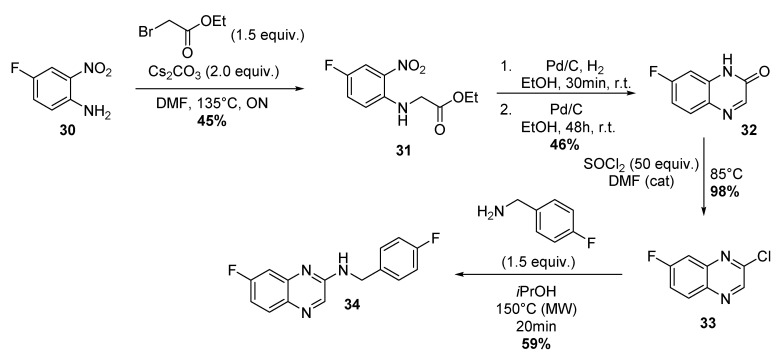
Synthesis of a Spautin-1 analogue bearing the quinoxaline core.

An alternative to the cinnoline core is the quinoxaline scaffold. 7-Fluoro-3,4-dihydro-1*H*-quinoxalin-2-one **32** was obtained from 4-fluoro-2-nitroaniline **30**, which was allowed to react with ethyl bromoacetate, yielding **31** (Scheme 7). Afterwards the nitro group was reduced, which immediately resulted in the formation of the cyclized product. After stirring the mixture for another 48 h in the absence of hydrogen gas, the desired core structure **32** was obtained. Similarly to abovementioned schemes, an analogous two-step strategy was used to couple the 4-fluorobenzylamine yielding **34**.

### 2.2. Biological Evaluation

#### 2.2.1. NSCLC Cell Viability Screening

First, the synthesized Spautin-1 analogues were subjected to an EGFR-mutant NSCLC cell viability screening to determine which compounds were the most promising in terms of reducing the viability of EGFR-mutant NSCLC cells in vitro. A reduction of cell viability in the presence of the analogues indicates induction of apoptosis and/or an inhibition of proliferation. The viability assay also helped to get insights in the structure–activity relationships (SARs) of the prepared analogues. As shown below, the analogues were subdivided in nine series in order to efficiently discover SAR trends.

**Figure 4:**
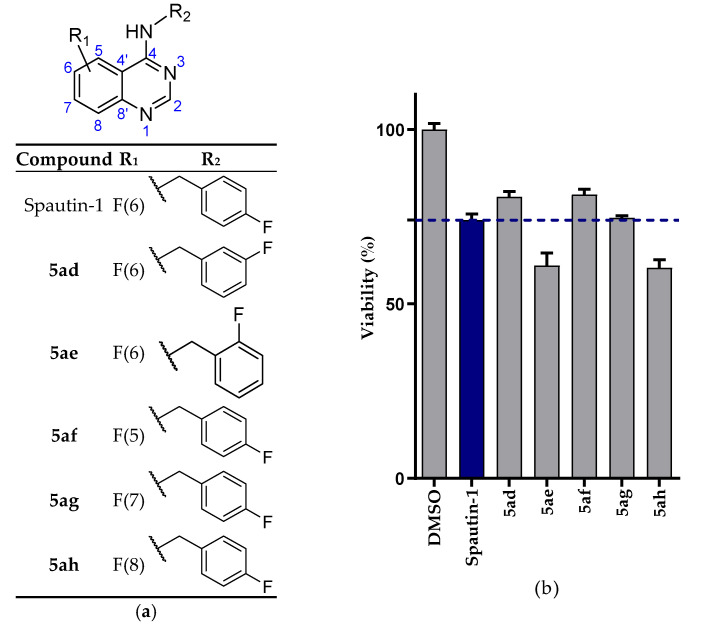
(**a**) Spautin-1 analogues synthesized for the “F-screening”; (**b**) viability assay (5 µM) with Spautin-1 as reference.

At first, a “*F*-screening” was performed to determine the importance of the fluorine substituent positioning (Figure 4). Remarkably, two compounds showed a significant decrease in tumor cell numbers compared to Spautin-1 (**5aa**), namely the analogue with a fluorine at the ortho position of the benzyl group **5ae** and the analogue with a fluorine at position 8 of the quinazoline core **5ah**. The other analogues were equally **5ag** or less potent **5ad** and **5af** as compared to Spautin-1.

**Figure 5:**
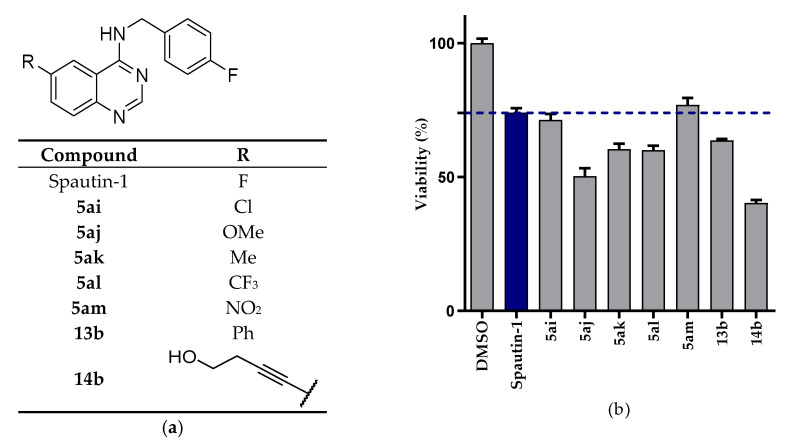
(**a**) Substituents at the 6-position of the quinazoline core were altered; (**b**) viability assay (5µM) with Spautin-1 as reference.

In the second series, multiple substituents, which differ in lipophilicity and electronic properties, were introduced at position 6 of the quinazoline core (Figure 5). It was observed that electron-donating substituents **5aj, 5ak** had a positive effect on the activity, compared to Spautin-1 **5aa,** while the effect of electron withdrawing groups **5ai, 5al**, **5am** was less consistent. A large hydrophobic phenyl group **13b** slightly improved the activity and confirmed that large substituents are probably allowed at this site of the central scaffold. Interestingly, a 4-hydroxybutynyl substituent at this position **14b**, previously described in EGFR TKIs [48], led to the strongest growth inhibition of the EGFR-mutant NSCLC cells. 

**Figure 6:**
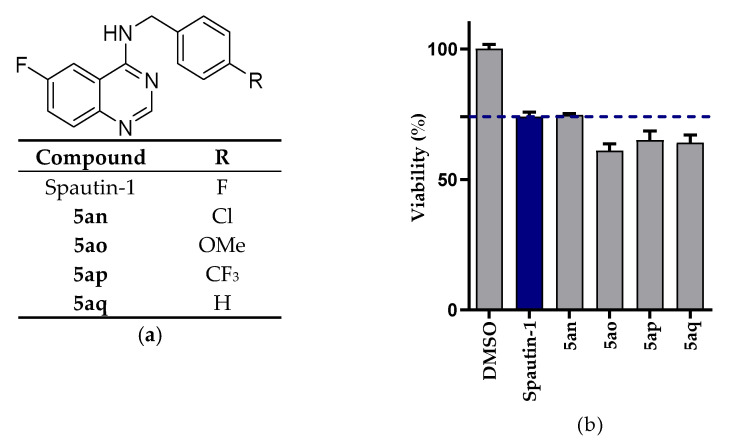
(**a**) Substituent effect on the para position of the phenyl group; (**b**) viability assay (5 µM) with Spautin-1 as reference.

The fluorine at the para position of the benzyl group was replaced by a variety of groups (Cl, OMe, CF_3_, H) (Figure 6). Notably, a methoxy **5ao**, trifluoromethyl **5ap** group and H atom **5aq** significantly improved the activity while the chlorinated analogue **5an** was equally potent to Spautin-1 (**5aa**). Nevertheless, the electron donating methoxy group resulted in the strongest reduction in the viability of the lung cancer cells in vitro.

**Figure 7:**
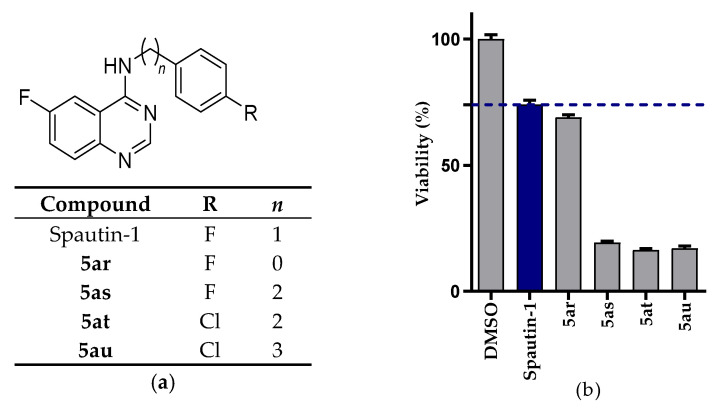
(**a**) Influence of the chain length; (**b**) viability assay (5µM) with Spautin-1 as reference.

To further improve the activity, the aliphatic chain length was shortened or extended, by altering the number of methylene groups (-CH_2_-) between the secondary amine and the phenyl group (Figure 7). Remarkably, both a reduction **5ar** and extension **5as-5au** resulted in slightly and significantly more potent analogues, respectively. The extended chain length clearly resulted in an improved activity, while the increased activity of the reduced chain length can be potentially linked to inhibition of EGFR (cfr. examples in Figure 1) [54,55]. We hypothesize that the additional methylene groups increase the flexibility and in this way improve the activity. 

**Figure 8:**
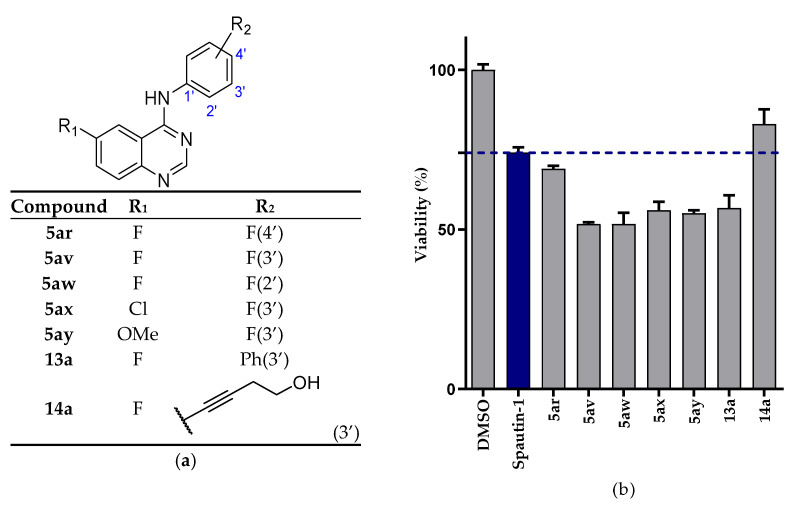
(**a**) Spautin-1 analogues with a reduced chain length; (**b**) viability assay (5 µM) with Spautin-1 as reference.

Despite the fact that analogues with *n* = 0 might act as EGFR TKIs, a small series of analogues was tested (Figure 8). Remarkably, a fluorine at the 3’ or 2’ position (**5av**, **5aw**) of the phenyl group significantly improved the activity as compared to the fluorine at the 4’ position **5ar**. Moreover, for *n* = 1, a fluorine substituent at the 2’ position also gave the best result (Figure 4, **5ae**). However, in the *n* = 1 series, a fluorine at the 3’ position resulted in a less active compound, as compared to Spautin-1, which indicates that a fluorine in proximity of the quinazoline core is beneficial. An electron-donating group at position R_1_, on the other hand, providing **5ay**, resulted in a weaker reduction in tumor cell viability as compared to **5av**. Remarkably, a phenyl group at the 3’ position of the phenyl group **13a** was allowed while the 1-hydroxy-3-butynyl group **14a** completely abolished the activity.

**Figure 9:**
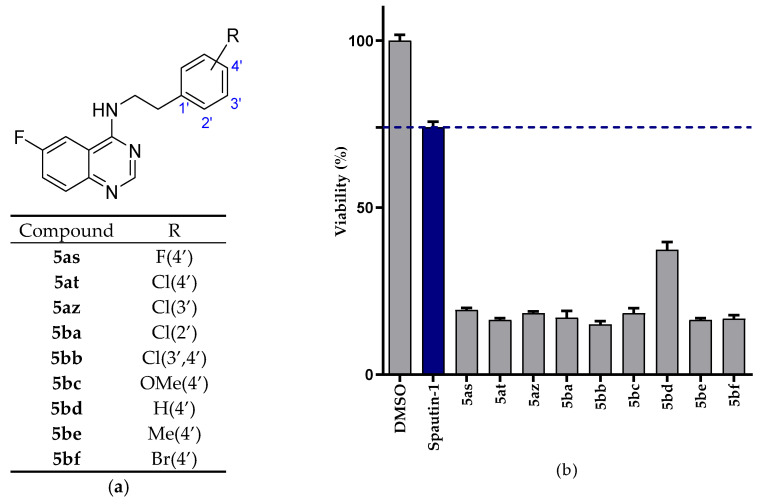
(**a**) Spautin-1 analogues with an extended chain length; (**b**) viability assay (5 µM) with Spautin-1 as reference.

Nonetheless, the most promising compounds have an extended chain between the secondary amine and the phenyl group. Highly comparable results were obtained for all analogues, except for analogue **5bd**, which was significantly less potent at a test concentration of 5 µM (Figure 9).

**Figure 10:**
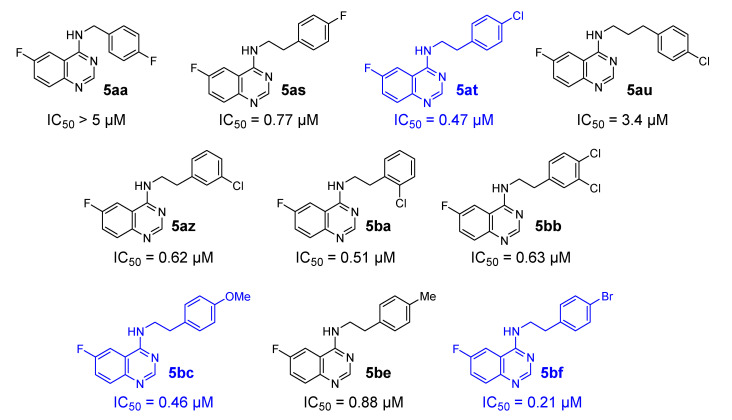
IC_50_ values of promising analogues with Spautin-1 as reference (most active compounds (IC_50_ < 0.5 µM) highlighted in blue).

To distinguish these analogues more precisely, IC_50_ values were determined to unravel differences in activity (see Figure S1). These data confirmed that the phenethylamine bearing analogues were definitely more potent than Spautin-1 **5aa** (Figure 10). Additionally, a chlorine **5at** at the para position of the phenyl group of the phenethylamine seemed beneficial for the potency compared to the fluorinated counterpart **5as** but further extension of the chain length, as in **5au**, lowered the potency by a factor of seven. Subsequently, the influence of the substitution pattern of the phenyl group on the activity was evaluated which revealed that the para substituted analogue **5at** is about 1.3 times more potent as compared to the meta **5az** and disubstituted **5bb**, and about 1.1 times more potent than the ortho substituted analogue **5ba**. Replacing the chlorine **5at** by a methoxy group **5bc** resulted in a slightly more active analogue. Remarkably, a methyl group at the para position was disadvantageous **5be**, while the brominated analogue **5bf** resulted in a further increase in activity as compared to **5at** and **5bc**. The observed trend in the IC_50_ values (F > Cl > Br) makes us belief that the formation of a halogen bond in the active site plays a significant role in the activity [56,57].

**Figure 11:**
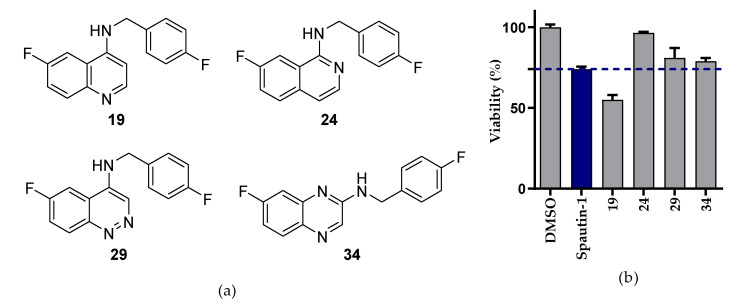
(**a**) Spautin-1 analogues for *N*-screening; (**b**) viability assay (5 µM) with Spautin-1 as reference.

Finally, a *N*-screening was performed to determine the importance of the quinazoline core (Figure 11). Interestingly, the nitrogen atom at the 3-position does not seem to be important for the activity, as the Spautin-1 analogue bearing the quinoline core (i.e., **19**) showed a higher potency as compared to the quinazoline core **5aa**. This is in contrast to the nitrogen at the 1-position, which appears to be crucial for the activity since its absence resulted in the inactive analogue **24** while the cinnoline **29** and quinoxaline **34** bearing Spautin-1 analogues showed some activity towards EGFR mutant NSCLC cells; however, they were less active compared to Spautin-1.

**Figure 12:**
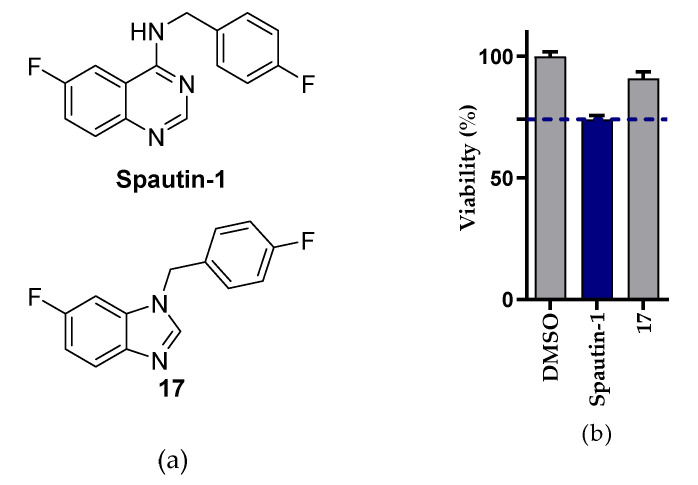
(**a**) Spautin-1 analogue **17** bearing the benzimidazole core; (**b**) viability assay (5 µM) with Spautin-1 as reference.

Remarkably, the Spautin-1 analogue bearing the benzimidazole core **17** turned out to be less active in the viability screening (Figure 12). We hypothesized that the lack of the secondary amine was responsible for the dramatically decreased activity.

**Figure 13:**
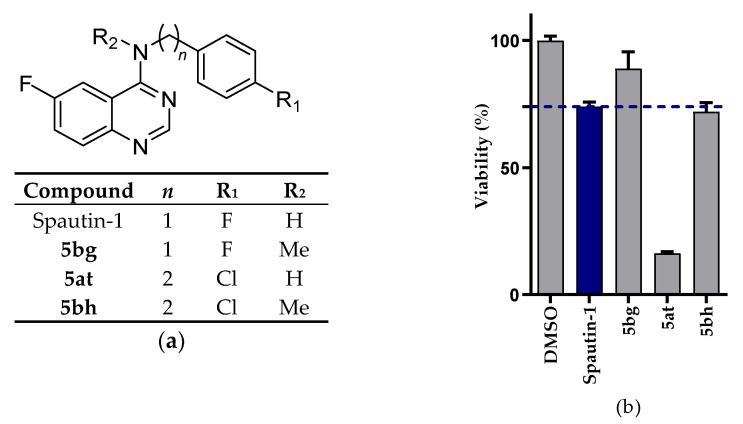
(**a**) Alkylation of the secondary amine; (**b**) viability assay (5 µM) with Spautin-1 as reference.

As a control reaction, the secondary amine present in most of the Spautin-1 analogues described above, was alkylated (Figure 13), since it has been reported that this secondary amine is crucial for the activity of EGRF-TKIs, [55] but also kinase inhibitors in general [58,59,60]. Methylation of the secondary amine dramatically reduced the activity of the analogues (**5aa** vs. **5bg** and **5at** vs. **5bh**) and as such, indicated that this secondary amine was also crucial for the activity in this study.

Next, the binding between the spautin-1 analogues and USP13 was evaluated to confirm that the reduced viability of the EGFR mutant NSCLC cells was caused by inhibition of USP13.

#### 2.2.2. ITC/TSA

In order to confirm the binding event between spautin-1 and USP13, an isothermal titration calorimetry (ITC) experiment was performed on recombinant USP13. No binding curve could be fitted on the obtained data (see Figure S2), thus no binding was detected. The addition of DMSO further complicated the experiment, resulting in buffer mismatches and aggregation of the protein. Since the purification yield of protein was low, it was decided to shift to another less consuming method for the analysis of compound binding.

An additional screening was performed by means of a thermal shift assay (TSA). Ligand interactions usually increase protein stability, resulting in a shift of the melting temperature with a few degrees. This assay would thus allow to determine the melting temperature (T_m_) and to detect a thermal shift upon binding of the ligand to the USP13 protein. Therefore, the protein was incubated with Spautin-1 **5aa** or **5bc** and a melting curve was determined for each sample (see Figure S3). Fitting a Boltzmann–sigmoidal curve to this data allows to determine the corresponding melting temperature, shown in Table 1. To determine a temperature difference, the reference condition was the protein in buffer with the corresponding percentage of DMSO, since addition of DMSO seemed to increase aggregation during the ITC experiment. However, for both compounds no consistent thermal shift was seen after incubation with the ligands. While binding could not be observed from this dataset, a potential impact on any binding resulting from producing the protein recombinantly in *Escherichia coli* can also not be excluded. 

#### 2.2.3. Kinase Screening

Since we could not confirm the interaction between Spautin-1 analogues and USP13, a kinase screening was performed using a set of four compounds (Spautin-1 and three analogues) to discover off-targets and identify kinases that are potentially responsible for the reduction in cell viability observed in our screen (Table 2). The KINOMEscan from Eurofins was performed (see Table S1). This screen consists of a competitive binding assay in which the DNA-tagged kinase is incubated with one of the compounds in the presence of immobilized active site binding ligands. Binding of the compounds to the kinase hampers the binding of the kinase to the ligands and this inhibition was quantified by qPCR. The residual kinase activity, which represents the percentage of kinase attached to the immobilized ligands, was determined for 428 kinases at a standard screening concentration of 10 µM of the compounds dissolved in DMSO [61]. 

Remarkably, Spautin-1 **5aa** showed moderate EGFR TK inhibitory activity at the standard screening concentration (Table 2), however we did not observe a reduction in pEGFR at 10 µM in EGFR mutant NSCLC cells [25]. We reasoned that the concentration used in the kinase screening does not represent the actual intracellular concentration upon treatment with the same concentration on a cellular basis. As expected, reducing the chain length, as in **5ay**, augments the EGFR TK inhibitory activity (Table 2), which probably also induced cell death in the viability screening. Conversely, extending the chain length (e.g., **5as** and **5at**), reduced the EGFR TK inhibitory activity, which implies that the improved activity, observed during the EGFR-mutant NSCLC cell viability screening, was not caused by inhibition of EGFR. Interestingly, the newly synthesized analogues (**5ay**, **5as** and **5at**) inhibited NEK4 more efficiently than Spautin-1 (**5aa**). Moreover, it has been reported that NEK4 is frequently overexpressed in lung cancer [37]. We confirmed the expression of NEK4 in our PC9 cell line model based on the publicly RNA-seq data available through the Cancer Cell Line Encyclopedia (CCLE) [62,63]. The available data represents RNA-seq expression data of a total of 1103 cell lines, including 205 lung cancer cell lines. Based on this data (see Table S2), we conclude that NEK4 is expressed throughout most NSCLC cell lines. Moreover, we found that PC9 expresses 19.3% more mRNA than the A549 NSCLC cell line, in which NEK4 has been functionally characterized and its expression confirmed through western blotting [38]. Collectively, this suggests that NEK4 inhibition possibly causes growth inhibition of EGFR mutant NSCLC cells.

**Figure 14:**
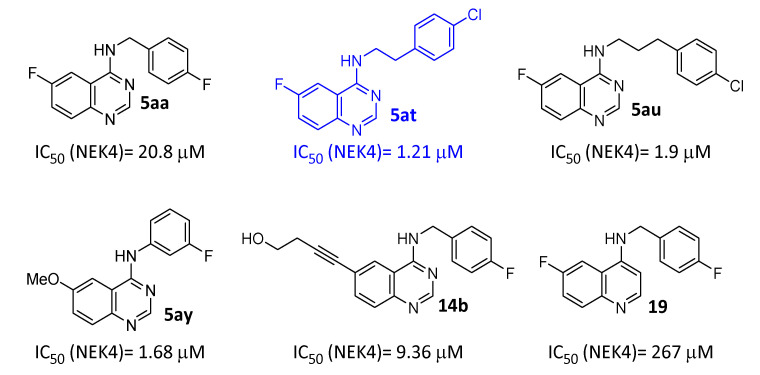
IC_50_ values NEK4 for Spautin-1 and 5 diverse analogues (most active compound highlighted in blue).

For the above stated reasons, we decided to determine the IC_50_ (NEK4) values for Spautin-1 and 5 diverse analogues (NEK4 Human Other Protein Kinase Enzymatic Radiometric Assay by Eurofins) (see Figure S4) [64]. Interestingly, a reduced (**5ay**) and extended (**5at**, **5au**) chain length between the secondary amine and the phenyl group improved the inhibitory activity for NEK4 (Figure 14), which is in line with the viability data. However, only for *n* ≥ 2 a dramatic decrease in NSCLC cell viability was observed and the activity of **5au** (*n* = 3) was significantly better as compared to **5ay** (*n* = 0) (Figure 7 and Figure 8) which is contrary to the IC_50_ values. Therefore, we postulate that inhibition of NEK4 only, does not fully explain the results of the viability screening. The introduction of a 1-hydroxy-3-butynyl group at position 6 of the quinazoline core **14b** resulted in a two-fold improved NEK4 inhibitory activity compared to Spautin-1, which suggests that the reduced cell viability was potentially caused by inhibition of NEK4. Remarkably, the Spautin-1 analogue bearing the quinoline core **19** was clearly less potent for NEK4 which is also in contrast to the viability data. 

Even though the IC_50_ (NEK4) values are not fully in line with the viability data, we have strong indications that our compounds bear great potential for the treatment of EGFR mutant NSCLC. The reduced viability is presumably a multifactorial effect of which USP13 and EGFR inhibition cannot be excluded. Further research is needed due to the lack of experimental data on NEK4 and EGFR inhibition at the doses used on a cellular level. The kinase screening also revealed a low remaining activity for CLK1 and CLK4 (Table 2), to our best knowledge, two kinases which have not been linked to lung cancer. However, these can probably be considered as moderate off-targets at the relatively high concentration of inhibitors used (10 μM).

## 3. Discussion

In this work, a SAR-study of spautin-1 was performed to discover lead compounds for the treatment of EGFR mutant NSCLC. The most important SAR trends can be summarized as follow: The secondary amine is crucial for the activity, which has been previously reported for kinase inhibitors in general [55,58,59,60]; an increased chain length (*n* ≥ 2) between the secondary amine and phenyl group dramatically increases the activity, in contrast to EGFR-TKIs [55]; however, the highest potency was observed for *n* = 2; a halogen capable of inducing an halogen bond at position 4’ of the phenyl group is beneficial for the activity. A *N*-screening was performed to determine the importance of the quinazoline core, which lead to the conclusion that the nitrogen atom at the 3-position does not seem to be important for the activity, as the analogue bearing the quinoline core showed a high potency. On the contrary, the nitrogen at the 1-position appears to be crucial for the activity, while the cinnoline and quinoxaline bearing Spautin-1 analogues showed limited activity towards EGFR mutant NSCLC cells. In line with these results, quinoline containing kinase inhibitors have been widely reported and some of these inhibitors were even approved by the FDA [27,28,29,30]. Altogether, the extensive SAR study provided compounds with IC_50_ values as low as 210 nM, as determined in the applied cell viability assay, opening up a gateway towards more potent lead structures.

Surprisingly, we were unable to confirm binding between spautin-1, or the analogues, and USP13. Inability to detect binding using TSA or ITC does not exclude a binding under physiological conditions. Previously published assays did not show direct binding either and it might be that Spautin-1 inhibits USP13 via an indirect way [26]. On the other hand, these assays used USP13 purified from eukaryotic cells, while our experiments were performed with recombinant human USP13 [26]. Possible differences in post-translational modifications, protein folding, protein stability and solubility of recombinant USP13 may create a bias which cannot be controlled for, as spautin-1 is the only known small molecule that may bind USP13. In search of off-targets of the investigated analogues, a broad kinase screening unraveled NEK4 as a potential target. The latter kinase is often overexpressed in lung cancer and the expression in our PC9 cell line model was confirmed [37]. The highest activity towards NEK4 was observed for the *N*-[2-(substituted-phenyl)ethyl]-4-quinazolinamines, which were also responsible for the lowest EGFR-mutant NSCLC cell viability. For this reason, we hypothesize that the decreased viability of NSCLC cells was at least partially caused by the inhibition of NEK4. As far as we know, *N*-[2-(substituted-phenyl)ethyl]-4-quinazolinamines have been reported as acetyl- and butyrylcholinesterase inhibitors (AChE IC_50_ = 6.2 μM; BuChE IC_50_ = 14.1 μM) for the treatment of Parkinson disease and inhibitors of NFκB [65,66]. These compounds showed moderate potency towards EGFR as compared to the anilino- and benzylamino derivatives [55], but were never related to NEK4.

In summary, we report *N*-[2-(substituted-phenyl)ethyl]-6-fluoro-4-quinazolinamines as promising single agent lead compounds for the future treatment of EGFR mutant NSCLC. To the best of our knowledge, the *N*-[2-(substituted-phenyl)ethyl]-6-fluoro-4-quinazolinamines are the first potent and relatively selective NEK4 inhibitors reported in literature to date. More research is ongoing to further increase the potency and selectivity of these lead compounds (e.g., replacing the quinazoline core by the quinoline core) and get insight in the mechanism of action. Such optimized inhibitors may eventually be combined with FDA approved EGFR-TKIs to obtain a stronger initial response, increase the progression-free survival and improve the quality of life in EGFR-mutant NSCLC patients [26].

## 4. Materials and Methods 

### 4.1. Chemistry

Unless stated otherwise, all commercial materials were used without further purification and were purchased from fluorochem Ltd. (14 Graphite Way, Hadfield, Derbyshire SK13 1QH United Kingdom) or Merck (2000 Galloping Hill Road, Kenilworth, NJ 07033 U.S.A.). Anhydrous *N*,*N*-dimethylformamide was obtained by storing under argon atmosphere on activated 4Å molecular sieves for 24 h prior to use. Non-commercial starting materials were prepared based on literature procedures and are described below. ^1^H and ^13^C NMR spectra were recorded using different spectrometers. A Bruker Avance II 500 spectrometer (Bruker Scientific LLC, 40 Manning Road, Manning Park, Billerica, MA 01821, USA) was used at 500 MHz (^1^H NMR) and 126 MHz (^13^C NMR) at ambient temperature. To obtain spectra at 250 MHz and 63 MHz, the Bruker Avance DRX 250 was used. The chemical shifts were reported in delta (δ) units in parts per million (ppm) relative to the signal of the deuterated solvent. For the CDCl_3_, the singlet in ^1^H NMR was calibrated at 7.26 ppm and the ^13^C NMR at the central line of the triplet at 77.16 ppm. For DMSO-d_6_, the calibration was performed at 2.50 ppm for the ^1^H NMR and 39.52 ppm for the ^13^C NMR, respectively. The assignments were made using one dimensional (1D) ^1^H and ^13^C spectra and two-dimensional (2D) HSQC, HMBC and COSY spectra. Multiplicities were described as singlet (s), doublet (d), triplet (t), quartet (q), multiplet (m), broad (br), or a combination thereof. The corresponding coupling constants (*J* values) were reported in Hertz (Hz). Analytical RP-HPLC analyses were carried out on a Chromaster system (VWR Hitachi, Researchpark Haasrode 2020, Geldenaaksebaan 464, 3001 Leuven) equipped with a Hitachi 5430 DAD, 5310 column oven, 5260 autosampler, and 5160 pump. Chromolith High Resolution RP-18e from Merck (150 Å, 1.1 µm, 50 × 4.6 mm, 3 mL/min flow rate) columns were used for analysis using UV detection at 214 nm. Solvents A and B are 0.1% TFA in water and 0.1% TFA in acetonitrile, respectively. Gradients are 1 to 100% B/A over 4 min. Mass spectra were recorded with a LC-MS triplequadrupole system. HPLC unit was a Waters 600 system combined to a Waters 2487 UV detector at 215 nm (Waters Corporation, 34 Maple Street, Milford, MA 01757, USA) and as stationary phase a Vydac MS RP C18-column (150 mm × 2.1 mm, 3 µm, 300 Å). The solvent system was 0.1% formic acid in water (A) and 0.1% formic acid in acetonitrile (B) with a gradient going from 3% to 100% B over 20 min with a flow rate of 0.3 mL/min. The MS unit, coupled to the HPLC system, was a Micromass QTOF-micro system. For the high resolution mass spectroscopy, the same MS system was used with reserpine (2.10–3 mg/mL) solution in H_2_O:CH_3_CN (1:1) as reference. Automated flash chromatography was performed using Grace^®^ Reveleris X2 system equipped with ELSD and UV detector (254 nm or 280 nm). The used normal phase column for the systems were Interchim^®^ Puriflash^®^ Silica HC 25 µM Flash Cartridges of 40 g or 80 g and Interchim^®^ Puriflash^®^ C18-HP 30 µM flash column of 40 g with a flow rate of 40 mL/min unless stated otherwise. Preparative RP-HPLC was performed on a Knauer system (Wuppertal, Germany) equipped with a RP-18C ReproSil-Pur ODS-3 column (150 mm × 16 mm, 5 mm) with a UV detector set at 214 nm. The same solvent system is used as for the analytical RP-HPLC with a flow rate of 10 mL/min. A Biotage^®^ Initiator + SPWave (Biotage Sweden AB, Box 8, 751 03 Uppsala Sweden) in organic synthesis mode was used for the microwave-assisted reactions. The microwave runs at a frequency of 2.45 GHz and the maximal pressure and temperature are respectively 20–30 bar and 250–300 °C. Upon completion of the reaction and cooling down to 40–50 °C, the cavity lid opens. The reactions were performed in microwave vials with a volume capacity ranging between 0.5–2.0 mL and 2.0–5.0 mL.

#### 4.1.1. General Procedure for the Synthesis of Substituted-Quinazolin-4-One **11**

Method 1: The synthesis was performed according to a literature procedure [43]. Into a flame-dried 10 mL microwave vial, the substituted-2-aminobenzamide **9** (3.0 mmol, 1.0 equiv.) was dissolved in anhydrous DMF (1.5 mL). Subsequently trimethyl orthoformate (3.3 mmol, 1.1 equiv.) was added and the mixture was heated for 2–3 h at 170 °C.

Then the mixture was allowed to cool to room temperature. The precipitate was collected by filtration, washed with CH_2_Cl_2_ and dried in vacuo yielding the desired compound in good purity. The product was used in the next step without further purification.

Method 2: The synthesis was performed according to a slightly adapted literature procedure [44]. Into a flame-dried 10 mL microwave vial, a mixture of substituted-2-aminobenzoic acid **10** (6.0 mmol, 1.0 equiv.) and formamide (0.95 mL, 24 mmol, 4.0 equiv.) was overnight stirred at 155 °C.

Then the mixture was allowed to cool to room temperature. The precipitate was collected by filtration, washed with CH_2_Cl_2_ and dried in vacuo yielding the desired compound in good purity. The product was used in the next step without further purification.

##### Synthesis of 6-Fluoro-Quinazolin-4-One **11a**

The title compound was prepared following general procedure Section 4.1.1. Method 1, from 2-amino-5-fluorobenzamide **9a** (0.500 g, 3.24 mmol, 1.00 equiv.) and HC(OMe)_3_ (0.390 mL, 3.57 mmol, 1.10 equiv.) in anhydrous DMF (1.62 mL). This yielded, after work-up, the desired compound as a white solid with 55% (324 mg, 1.79 mmol) yield. ^1^H NMR (500 MHz, DMSO): δ 8.09 (s, 1H), 7.79 (dd, *J* = 8.7, 2.9 Hz, 1H), 7.77–7.73 (m, 1H), 7.70 (dt, *J* = 8.7, 2.9 Hz, 1H). HPLC: 1.34 min. MS(ES+): [M+H]^+^ = 165.

##### Synthesis of 5-Fluoro-Quinazolin-4-One **11b**

The title compound was prepared following general procedure Section 4.1.1. Method 1, from 2-amino-4-fluorobenzamide **9b** (0.500 g, 3.24 mmol, 1.00 equiv.) and HC(OMe)_3_ (0.390 mL, 3.57 mmol, 1.10 equiv.) in anhydrous DMF (1.62 mL). This yielded, after work-up, the desired compound as a white solid with 52% (275 mg, 1.67 mmol) yield. ^1^H NMR (500 MHz, DMSO): δ 8.18 (dd, *J* = 8.8, 6.4 Hz, 1H), 8.15–8.12 (m, 1H), 7.45 (dd, *J* = 10.10, 2.5 Hz, 1H), 7.39 (dt, *J* = 8.8, 2.5 Hz, 1H). HPLC: 1.40 min. MS(ES+): [M+H]^+^ = 165.

##### Synthesis of 7-Fluoro-Quinazolin-4-One **11c**

The title compound was prepared following general procedure Section 4.1.1. Method 1, from 2-amino-6-fluorobenzamide **9c** (1.00 g, 6.49 mmol, 1.00 equiv.) and HC(OMe)_3_ (0.78 mL, 7.14 mmol, 1.10 equiv.) in anhydrous DMF (3.24 mL). This yielded, after work-up, the desired compound as a white solid with 48% (515 mg, 3.14 mmol) yield. ^1^H NMR (500 MHz, DMSO): δ 8.11 (s, 1H), 7.79 (dt, *J* = 8.3, 5.6 Hz, 1H), 7.48 (d, 8.3 Hz, 1H), 7.27 (dd, *J* = 11.2, 8.3 Hz, 1H). HPLC: 1.29 min. MS(ES+): [M+H]^+^ = 165.

##### Synthesis of 8-Fluoro-Quinazolin-4-One **11d**

The title compound was prepared following general procedure Section 4.1.1. Method 2, from 2-amino-3-fluorobenzoic acid **10a** (1.00 g, 6.45 mmol, 1.00 equiv.) and formamide (1.05 mL, 26.5 mmol, 4.00 equiv.). This yielded, after work-up, the desired compound as a white solid with 77% (809 mg, 4.93 mmol) yield. ^1^H NMR (500 MHz, DMSO): δ 8.14 (d, *J* = 3.5 Hz, 1H), 7.95–7.91 (m, 1H), 7.69 (ddd, *J* = 10.9, 8.0, 1.4 Hz, 1H), 7.51 (dt, *J* = 8.0, 4.8 Hz, 1H). HPLC: 1.40 min. MS(ES+): [M+H]^+^ = 165.

##### Synthesis of 6-Chloro-Quinazolin-4-One **11e**

The title compound was prepared following general procedure Section 4.1.1. Method 1, from 2-amino-5-Chlorobenzamide **9d** (0.600 g, 3.52 mmol, 1.00 equiv.) and HC(OMe)_3_ (0.42 mL, 3.87 mmol, 1.10 equiv.) in anhydrous DMF (1.76 mL). This yielded, after work-up, the desired compound as a white solid with 59% (374 mg, 2.07 mmol) yield. ^1^H NMR (500 MHz, DMSO): δ 8.14–8.11 (m, 1H), 8.06 (d, *J* = 2.5 Hz, 1H), 7.85 (dd, *J* = 8.7, 2.5 Hz, 1H), 7.70 (d, *J* = 8.7 Hz, 1H). HPLC: 1.67 min. MS(ES+): [M+H]^+^ = 181 and 183.

##### Synthesis of 6-Methoxy-Quinazolin-4-One **11f**

The title compound was prepared following general procedure Section 4.1.1. Method 1, from 2-amino-5-methoxybenzamide **9e** (0.500 g, 3.01 mmol, 1.00 equiv.) and HC(OMe)_3_ (0.360 mL, 3.31 mmol, 1.10 equiv.) in anhydrous DMF (1.50 mL). This yielded, after work-up, the desired compound as a white solid with 78% (413 mg, 2.34 mmol) yield. ^1^H NMR (500 MHz, DMSO): δ 8.18 (s, 1H), 7.64 (d, *J* = 8.9 Hz, 1H), 7.52 (d, *J* = 3.0 Hz, 1H), 7.45 (dd, *J* = 8.9, 3.0 Hz, 1H), 3.88 (s, 3H). HPLC: 1.40 min. MS(ES+): [M+H]^+^ = 177.

##### Synthesis of 6-Methyl-Quinazolin-4-One **11g**

The title compound was prepared following general procedure Section 4.1.1. Method 2, from 2-amino-5-Methylbenzoic acid **10b** (1.00 g, 6.62 mmol, 1.00 equiv.) and formamide (1.05 mL, 26.5 mmol, 4.00 equiv.). This yielded, after work-up, the desired compound as a white solid with 81% (858 mg, 5.36 mmol) yield. ^1^H NMR (500 MHz, DMSO): δ 8.28 (s, 1H), 7.95–7.92 (m, 1H), 7.67 (dd, *J* = 8.3, 1.9 Hz, 1H), 7.59 (d, *J* = 8.3 Hz, 1H), 2.45 (s, 3H). HPLC: 1.39 min. MS(ES+): [M+H]^+^ = 161.

##### Synthesis of 6-Trifluoromethyl-Quinazolin-4-One **11h**

The title compound was prepared following general procedure Section 4.1.1. Method 2, from 2-amino-5-(trifluoromethyl)benzoic acid **10c** (0.500 g, 2.44 mmol, 1.00 equiv.) and formamide (0.390 mL, 9.75 mmol, 4.00 equiv.). This yielded, after work-up, the desired compound as a white solid with 64% (334 mg, 1.56 mmol) yield. ^1^H NMR (500 MHz, DMSO): δ 8.39–8.35 (m, 1H), 8.28–8.23 (m, 1H), 8.12 (dd, *J* = 8.5, 2.3 Hz, 1H), 7.87 (d, *J* = 8.5 Hz, 1H). HPLC: 1.87 min. MS(ES+): [M+H]^+^ = 215.

##### Synthesis of 6-Nitro-Quinazolin-4-One **11i**

The title compound was prepared following general procedure Section 4.1.1. Method 2, from 2-amino-5-Nitrobenzoic acid **10d** (1.00 g, 5.49 mmol, 1.00 equiv.) and formamide (0.870 mL, 22.0 mmol, 4.00 equiv.). This yielded, after work-up, the desired compound as a yellow solid with 77% (809 mg, 4.23 mmol) yield. ^1^H NMR (500 MHz, DMSO): δ 8.81 (d, *J* = 2.7 Hz, 1H), 8.55 (dd, *J* = 8.9, 2.7 Hz, 1H), 8.32 (s, 1H), 7.87 (d, *J* = 8.9 Hz, 1H). HPLC: 1.52 min. MS(ES+): [M+H]^+^ = 192.

##### Synthesis of 6-Iodo-Quinazolin-4-One **11j**

The title compound was prepared following general procedure Section 4.1.1. Method 2, from 2-amino-5-Iodobenzoic acid **10e** (1.00 g, 3.80 mmol, 1.00 equiv.) and formamide (0.600 mL, 15.2 mmol, 4.00 equiv.). This yielded, after work-up, the desired compound as a white solid with 62% (641 mg, 2.36 mmol) yield. ^1^H NMR (500 MHz, DMSO): δ 8.39 (d, *J* = 2.1 Hz, 1H), 8.13 (s, 1H), 8.10 (dd, *J* = 8.6, 2.1 Hz, 1H), 7.46 (d, *J* = 8.6 Hz, 1H). HPLC: 1.81 min. MS(ES+): [M+H]^+^ = 273.

#### 4.1.2. General Procedure for the Synthesis of Substituted-4-Chloro-Quinazoline **12**

For the synthesis of substituted-4-Chloro-quinazolines, the procedure described by Zhang et al. was followed [45]. Into a flame dried round bottom flask equipped with an oven-dried reflux condenser, a mixture of substituted-quinazolin-4-one (1 equiv.) in SOCl_2_ (50 equiv.), containing DMF (2 drops) was prepared. The mixture was refluxed for 4–8 h under argon atmosphere. SOCl_2_ was removed under reduced pressure. The residue was dissolved in CH_2_Cl_2_ (50 mL) and washed three times with HCl (1M, aq). Subsequently, the organic phase was dried over Na_2_SO_4_, filtered and the solvent removed under reduced pressure.

##### Synthesis of 4-Chloro-6-Fluoroquinazoline **12a**

The title compound was prepared following general procedure Section 4.1.2, from 6-Fluoroquinazolin-4-one **11a** (0.450 g, 2.74 mmol, 1.00 equiv.) in SOCl_2_ (10.0 mL). This yielded, after work-up, the desired compound as a pale yellow solid with 99% (496 mg, 2.72 mmol) yield. ^1^H NMR (250 MHz, CDCl_3_): δ 9.01 (s, 1H), 8.13–8.03 (m, 1H), 7.90–7.81 (m, 1H), 7.77–7.65 (m, 1H). HPLC: 2.01 min. MS(ES+): [M+H]^+^ = 183 and 185.

##### Synthesis of 4-Chloro-5-Fluoroquinazoline **12b**

The title compound was prepared following general procedure Section 4.1.2, from 5-Fluoroquinazolin-4-one **11b** (0.450 g, 2.74 mmol, 1.00 equiv.) in SOCl_2_ (10.0 mL). This yielded, after work-up, the desired compound as a pale yellow solid with 98% (492 mg, 2.69 mmol) yield. ^1^H NMR (250 MHz, CDCl_3_): δ 9.00 (s, 1H), 7.94–7.85 (m, 2H), 7.42–7.31 (m, 1H). HPLC: 2.04 min. MS(ES+): [M+H]^+^ = 183 and 185.

##### Synthesis of 4-Chloro-7-Fluoroquinazoline **12c**

The title compound was prepared following general procedure Section 4.1.2, from 7-Fluoroquinazolin-4-one **11c** (0.225 g, 1.37 mmol, 1.00 equiv.) in SOCl_2_ (5.00 mL). This yielded, after work-up, the desired compound as a pale yellow solid with 97% (243 mg, 1.33 mmol) yield. ^1^H NMR (250 MHz, CDCl_3_): δ 9.03 (s, 1H), 8.32 (dd, *J* = 9.3, 5.8 Hz, 1H), 7.69 (dd, *J* = 9.3, 2.2 Hz, 1H), 7.56–7.42 (m, 1H). HPLC: 2.00 min. MS(ES+): [M+H]^+^ = 183 and 185.

##### Synthesis of 4-Chloro-8-Fluoroquinazoline **12d**

The title compound was prepared following general procedure Section 4.1.2, from 8-Fluoroquinazolin-4-one **11d** (0.500 g, 3.05 mmol, 1.00 equiv.) in SOCl_2_ (11.1 mL). This yielded, after work-up, the desired compound as a pale yellow solid with 94% (523 mg, 2.86 mmol) yield. ^1^H NMR (250 MHz, CDCl_3_): δ 9.06 (s, 1H), 8.09–8.00 (m, 1H), 7.76–7.60 (m, 2H). HPLC: 1.83 min. MS(ES+): [M+H]^+^ = 183 and 185.

##### Synthesis of 4,6-Dichloroquinazoline **12e**

The title compound was prepared following general procedure Section 4.1.2, from 6-Chloroquinazolin-4-one **11e** (0.500 g, 2.77 mmol, 1.00 equiv.) in SOCl_2_ (10.1 mL). This yielded, after work-up, the desired compound as a pale yellow solid with 99% (546 mg, 2.74 mmol) yield. ^1^H NMR (250 MHz, CDCl_3_): δ 9.03 (s, 1H), 8.22 (d, *J* = 2.3 Hz, 1H), 8.01 (d, *J* = 9.0 Hz, 1H), 7.87 (dd, *J* = 9.0, 2.3 Hz, 1H). HPLC: 2.37 min. MS(ES+): [M+H]^+^ = 199, 201 and 203.

##### Synthesis of 4-Chloro-6-Methoxyquinazoline **12f**

The title compound was prepared following general procedure Section 4.1.2, from 6-Methoxyquinazolin-4-one **11f** (0.250 g, 1.42 mmol, 1.00 equiv.) in SOCl_2_ (5.20 mL). This yielded, after work-up, the desired compound as a pale yellow solid with 99% (274 mg, 1.41 mmol) yield. ^1^H NMR (250 MHz, CDCl_3_): δ 8.92 (s, 1H), 7.96 (d, J = 9.2 Hz, 1H), 7.58 (dd, *J* = 9.2, 2.8 Hz, 1H), 7.42 (d, J = 2.8 Hz, 1H), 3.99 (s, 3H). HPLC: 2.12 min. MS(ES+): [M+H]^+^ = 195 and 197.

##### Synthesis of 4-Chloro-6-Methylquinazoline **12g**

The title compound was prepared following general procedure Section 4.1.2, from 6-Methylquinazolin-4-one **11g** (0.200 g, 1.11 mmol, 1.00 equiv.) in SOCl_2_ (4.04 mL). This yielded, after work-up, the desired compound as a pale yellow solid with 77% (152 mg, 0.85 mmol) yield. ^1^H NMR (250 MHz, CDCl_3_): δ 8.98 (s, 1H), 8.04–8.00 (m, 1H), 7.96 (d, *J* = 8.6 Hz, 1H), 7.78 (dd, *J* = 8.6, 1.8 Hz, 1H), 2.60 (s, 3H). HPLC: 2.18 min. MS(ES+): [M+H]^+^ = 179 and 181.

##### Synthesis of 4-Chloro-6-Trifluoromethyl-Quinazoline **12h**

The title compound was prepared following general procedure Section 4.1.2, from 5-Trifluoromethyl-quinazolin-4-one **11h** (0.200 g, 0.930 mmol, 1.00 equiv.) in SOCl_2_ (3.41 mL). This yielded, after work-up, the desired compound as a pale yellow solid with 59% (128 mg, 0.55 mmol) yield. ^1^H NMR (250 MHz, CDCl_3_): Too unstable to perform NMR-analysis. HPLC: 2.46 min. MS(ES+): [M+H]^+^ = 233 and 235.

##### Synthesis of 4-Chloro-6-Nitroquinazoline **12i**

The title compound was prepared following general procedure Section 4.1.2, from 6-Nitroquinazolin-4-one **11i** (0.500 g, 2.60 mmol, 1.00 equiv.) in SOCl_2_ (9.50 mL). This yielded, after work-up, the desired compound as a yellow solid with 70% (380 mg, 1.81 mmol) yield. ^1^H NMR (250 MHz, CDCl_3_): δ 9.22–9.19 (m, 2H), 8.73 (dd, *J* = 9.2, 2.5 Hz, 1H), 8.26 (d, *J* = 9.2 Hz, 1H). HPLC: 1.99 min. MS(ES+): [M+H]^+^ = 210 and 212.

##### Synthesis of 4-Chloro-6-Iodoquinazoline **12j**

The title compound was prepared following general procedure Section 4.1.2, from 6-Iodoquinazolin-4-one **11j** (0.500 g, 1.84 mmol, 1.00 equiv.) in SOCl_2_ (6.70 mL). This yielded, after work-up, the desired compound as a pale yellow solid with 93% (495 mg, 1.71 mmol) yield. ^1^H NMR (250 MHz, CDCl_3_): δ 9.06 (s, 1H), 8.65 (d, J = 1.9 Hz, 1H), 8.20 (dd, *J* = 8.9, 1.9 Hz, 1H), 7.79 (d, *J* = 8.9 Hz, 1H). HPLC: 2.50 min. MS(ES+**):** [M+H]^+^ = 291 and 293.

#### 4.1.3. General Procedure for the Synthesis of Spautin-1 and Analogues **5**

The synthesis of 4-amino-6-substituted quinazolines was performed according a literature procedure [46]. Into a 5 mL microwave vial, 4-Chloro-substituted-quinazoline (30 mg, 1.0 equiv.) was dissolved in *i*PrOH (0.30–0.80 mL). Subsequently the corresponding amine (1.5 equiv.) and Et_3_N (3.0 equiv.) were added. The mixture was heated for 20–180 min (depending on the amine) at 150 °C using microwave irradiation. 

Afterwards the solvent was removed under reduced pressure and the crude mixture was purified using preparative HPLC (AcN + 0.1% TFA/H_2_O + 0.1% TFA).

##### Synthesis of 6-Fluoro-N-(4-fluorobenzyl)-4-Quinazolinamine.TFA **5aa**

The title compound was prepared following general procedure Section 4.1.3, from 4-Chloro-6-fluoroquinazoline **12a** (0.030 g, 0.16 mmol, 1.0 equiv.), 4-fluorobenzylamine (0.028 mL, 0.25 mmol, 1.5 equiv.) and Et_3_N (0.069 mL, 0.49 mmol, 3.0 equiv.) in *i*PrOH (0.40 mL). This yielded, after purification, the desired compound as a white solid with 63% (38.9 mg, 0.10 mmol) yield. ^1^H NMR (500 MHz, DMSO): δ 10.51–10.44 (m, 1H), 8.89 (s, 1H), 8.38 (dd, *J* = 9.7, 2.1 Hz, 1H), 7.99–7.90 (m, 2H), 7.49–7.43 (m, 2H), 7.20–7.15 (m, 2H), 4.92 (d, *J* = 5.5 Hz, 2H). ^13^C NMR (126 MHz, DMSO): δ 162.49 (C), 161.14 (C), 160.56 (C), 160.25 (C), 159.18 (C), 151.47 (CH), 136.33 (C), 133.33 (C), 129.78 (CH), 129.72 (CH), 124.72 (CH), 124.52 (CH), 123.62 (CH), 123.56 (CH), 115.30 (CH), 115.13 (CH), 114.45 (C), 114.38 (C), 109.19 (CH), 109.00 (CH), 44.07 (CH_2_). HPLC: 1.95 min. HRMS (ESI^+^) *m*/*z* calc. for C_15_H_12_F_2_N_3_ [M+H]^+^ = 272.0994, found 272.0997.

##### Synthesis of 6-fluoro-N-(3-iodophenyl)-4-quinazolinamine.TFA **5ab**

The title compound was prepared following general procedure Section 4.1.3, from 4-Chloro-6-fluoroquinazoline **12a** (0.060 g, 0.33 mmol, 1.0 equiv.), 3-Iodoaniline (0.059 mL, 0.49 mmol, 1.5 equiv.) and Et_3_N (0.14 mL, 0.99 mmol, 3.0 equiv.) in *i*PrOH (0.80 mL). This yielded, after purification, the desired compound as pale yellow solid with 45% (70.4 mg, 0.14 mmol) yield. ^1^H NMR (500 MHz, DMSO): δ 10.32 (br s, 1H), 8.79 (s, 1H), 8.49 (dd, J = 10.0, 2.4 Hz, 1H), 8.29–8.26 (m, 1H), 7.95–7.87 (m, 3H), 7.60–7.55 (m, 1H), 7.25 (t, *J* = 8.0 Hz, 1H). ^13^C NMR (126 MHz, DMSO): δ 160.83 (C), 158.87 (C), 158.04 (C), 152.81 (CH), 142.84 (C), 139.57 (C), 133.27 (CH), 130.92 (CH), 130.64 (CH), 127.98 (CH), 123.80 (CH), 123.60 (CH), 122.09 (CH), 115.38 (C), 115.31 (C), 108.25 (CH), 108.05 (CH), 94.19 (C). HPLC: 1.92 min. HRMS (ESI^+^) *m*/*z* calc. for C_14_H_10_FIN_3_ [M+H]^+^ = 365.9904, found 365.9923.

##### Synthesis of N-(4-Fluorobenzyl)-6-iodo-4-Quinazolinamine.TFA **5ac**

The title compound was prepared following general procedure Section 4.1.3, from 4-Chloro-6-iodoquinazoline **12j** (0.030 g, 0.10 mmol, 1.0 equiv.), 4-fluorobenzylamine (0.017 mL, 0.15 mmol, 1.5 equiv.) and Et_3_N (0.042 mL, 0.30 mmol, 3.0 equiv.) in *i*PrOH (0.33 mL). This yielded, after purification, the desired compound as a pale yellow solid with 66% (32.6 mg, 0.07 mmol) yield. ^1^H NMR (500 MHz, DMSO): δ 10.40–10.26 (m, 1H), 8.91 (s, 1H), 8.87 (s, 1H), 8.30–8.25 (m, 1H), 7.57 (d, *J* = 8.8 Hz, 1H), 7.49–7.42 (m, 2H), 7.22–7.15 (m, 2H), 4.89 (d, *J* = 5.6 Hz, 2H). ^13^C NMR (126 MHz, DMSO): δ 162.50 (C), 160.57 (C), 159.14 (C), 152.21 (CH), 143.51 (C), 139.23 (C), 133.45 (C), 132.34 (CH), 129.85 (CH), 129.78 (CH), 122.82 (CH), 115.35 (CH), 115.18 (CH), 115.08 (C), 93.24 (C), 44.05 (CH_2_). HPLC: 2.17 min. HRMS (ESI^+^) *m*/*z* calc. for C_15_H_12_FIN_3_ [M+H]^+^ = 380.0060, found 380.0101.

##### Synthesis of 6-Fluoro-N-(3-Fluorobenzyl)-4-Quinazolinamine.TFA **5ad**

The title compound was prepared following general procedure Section 4.1.3, from 4-Chloro-6-fluoroquinazoline **12a** (0.030 g, 0.16 mmol, 1.0 equiv.), 3-fluorobenzylamine (0.028 mL, 0.25 mmol, 1.5 equiv.) and Et_3_N (0.069 mL, 0.49 mmol, 3.0 equiv.) in *i*PrOH (0.40 mL). This yielded, after purification, the desired compound as a colorless oil with 70% (43.3 mg, 0.11 mmol) yield. ^1^H NMR (500 MHz, DMSO): δ 10.54–10.49 (m, 1H), 8.90 (s, 1H), 8.39 (dd, *J* = 9.6, 2.4 Hz, 1H), 8.00–7.91 (m, 2H), 7.43–7.37 (m, 1H), 7.29–7.23 (m, 2H), 7.14–7.09 (m, 1H), 4.96 (d, *J* = 5.6 Hz, 2H). ^13^C NMR (126 MHz, DMSO): δ 163.26 (C), 161.32 (C), 161.19 (C), 160.50 (C), 160.47 (C), 159.22 (C), 151.53 (CH), 140.17 (C), 140.11 (C), 136.29 (C), 130.53 (CH), 130.47 (CH) 124.85 (CH), 124.65 (CH), 123.62 (CH), 123.60 (CH), 123.54 (CH), 114.55 (C), 114.48 (C), 114.45 (CH), 114.30 (CH), 114.27 (CH), 114.14 (CH), 109.32 (CH), 109.12 (CH), 44.25 (CH_2_). HPLC: 1.93 min. HRMS (ESI^+^) *m*/*z* calc. for C_15_H_12_F_2_N_3_ [M+H]^+^ = 272.0994, found 272.0986.

##### Synthesis of 6-Fluoro-N-(2-Fluorobenzyl)-4-Quinazolinamine.TFA **5ae**

The title compound was prepared following general procedure Section 4.1.3, from 4-Chloro-6-fluoroquinazoline **12a** (0.030 g, 0.16 mmol, 1.0 equiv.), 2-fluorobenzylamine (0.028 mL, 0.25 mmol, 1.5 equiv.) and Et_3_N (0.069 mL, 0.49 mmol, 3.0 equiv.) in *i*PrOH (0.40 mL). This yielded, after purification, the desired compound as a colorless oil with 63% (39.0 mg, 0.10 mmol) yield. ^1^H NMR (500 MHz, DMSO): δ 10.50–10.43 (m, 1H), 8.91 (s, 1H), 8.43 (dd, *J* = 9.6, 2.4 Hz, 1H), 8.00–7.92 (m, 2H), 7.47 (dt, *J* = 7.7, 1.4 Hz, 1H), 7.40–7.34 (m, 1H), 7.26–7.21 (m, 1H), 7.20–7.15 (m, 1H), 4.97 (d, *J* = 5.3 Hz, 2H). ^13^C NMR (126 MHz, DMSO): δ 161.27 (C), 161.23 (C), 160.47 (C), 160.44 (C), 159.32 (C), 159.26 (C), 151.47 (CH), 136.23 (C), 130.01 (CH), 129.98 (CH), 129.72 (CH), 129.65 (CH), 124.92 (CH), 124.72 (CH), 124.54 (CH), 124.51 (CH), 123.86 (C), 123.74 (C), 123.62 (CH), 123.55 (CH), 115.44 (CH), 115.28 (CH), 114.46 (C), 114.39 (C), 109.33 (CH), 109.13 (CH), 39.04 (CH_2_), 39.00 (CH_2_). HPLC: 1.92 min. HRMS (ESI^+^) *m*/*z* calc. for C_15_H_12_F_2_N_3_ [M+H]^+^ = 272.0994, found 272.0985.

##### Synthesis of 5-Fluoro-N-(4-Fluorobenzyl)-4-Quinazolinamine.TFA **5af**

The title compound was prepared following general procedure Section 4.1.3, from 4-Chloro-5-fluoroquinazoline **12b** (0.035 g, 0.19 mmol, 1.0 equiv.), 4-fluorobenzylamine (0.033 mL, 0.29 mmol, 1.5 equiv.) and Et_3_N (0.080 mL, 0.58 mmol, 3.0 equiv.) in *i*PrOH (0.48 mL). This yielded, after purification, the desired compound as a white solid with 69% (50.3 mg, 0.13 mmol) yield. ^1^H NMR (500 MHz, DMSO): δ 9.70–9.59 (m, 1H), 8.82 (s, 1H), 8.03–7.96 (m, 1H), 7.65–7.55 (m, 2H), 7.50–7.44 (m, 2H), 7.20–7.12 (m, 2H), 4.90 (d, *J* = 5.8 Hz, 2H). ^13^C NMR (126 MHz, DMSO): δ 162.34 (C), 160.41 (C), 159.28 (C), 158.37 (C), 158.34 (C), 157.25 (C), 152.57 (CH), 142.12 (C), 136.31 (CH), 136.23 (CH), 133.80 (C), 133.78 (C), 129.58 (CH), 129.51 (CH), 117.43 (CH), 115.19 (CH), 115.03 (CH), 113.57 (CH), 113.39 (CH), 104.04 (C), 103.93 (C), 44.39 (CH_2_). HPLC: 1.92 min. HRMS (ESI^+^) *m*/*z* calc. for C_15_H_12_F_2_N_3_ [M+H]^+^ = 272.0994, found 272.0975.

##### Synthesis of 7-Fluoro-N-(4-Fluorobenzyl)-4-Quinazolinamine.TFA **5ag**

The title compound was prepared following general procedure Section 4.1.3, from 4-Chloro-7-fluoroquinazoline **12c** (0.035 g, 0.19 mmol, 1.0 equiv.), 4-fluorobenzylamine (0.033 mL, 0.29 mmol, 1.5 equiv.) and Et_3_N (0.080 mL, 0.58 mmol, 3.0 equiv.) in *i*PrOH (0.48 mL). This yielded, after purification, the desired compound as a white solid with 66% (48.0 mg, 0.12 mmol) yield. ^1^H NMR (500 MHz, DMSO): δ 10.70–10.64 (m, 1H), 8.91 (s, 1H), 8.61 (dd, *J* = 9.2, 5.3 Hz, 1H), 7.72 (dt, J = 9.2, 2.4 Hz, 1H), 7.64 (dd, *J* = 9.2, 2.4 Hz, 1H), 7.49–7.43 (m, 2H), 7.21–7.15 (m, 2H), 4.93 (d, *J* = 5.6 Hz, 2H). ^13^C NMR (126 MHz, DMSO): δ 166.55 (C), 164.52 (C), 162.53 (C), 160.60 (C), 160.17 (C), 152.50 (CH), 140.86 (C), 140.76 (C), 133.37 (C), 133.35 (C), 129.79 (CH), 129.73 (CH), 127.92 (CH), 127.83 (CH), 117.55 (CH), 117.36 (CH), 115.38 (CH), 115.21 (CH), 110.36 (C), 110.35 (C), 105.78 (CH), 105.58 (CH), 44.08 (CH_2_). HPLC: 1.92 min. HRMS (ESI^+^) *m*/*z* calc. for C_15_H_12_F_2_N_3_ [M+H]^+^ = 272.0994, found 272.0977.

##### Synthesis of 8-Fluoro-N-(4-Fluorobenzyl)-4-Quinazolinamine.TFA **5ah**

The title compound was prepared following general procedure Section 4.1.3, from 4-Chloro-8-fluoroquinazoline **12d** (0.035 g, 0.19 mmol, 1.0 equiv.), 4-fluorobenzylamine (0.033 mL, 0.29 mmol, 1.5 equiv.) and Et_3_N (0.080 mL, 0.58 mmol, 3.0 equiv.) in *i*PrOH (0.47 mL). This yielded, after purification, the desired compound as a white solid with 61% (44.3 mg, 0.11 mmol) yield. ^1^H NMR (500 MHz, DMSO): **δ** 10.10–10.01 (m, 1H), 8.73 (s, 1H), 8.24 (d, *J* = 8.4 Hz, 1H), 7.86–7.79 (m, 1H), 7.70–7.63 (m, 1H), 7.47–7.41 (m, 2H), 7.20–7.13 (m, 2H), 4.88 (d, *J* = 5.5 Hz, 2H). ^13^C NMR (126 MHz, DMSO): δ 162.40 (C), 160.47 (C), 159.48 (C), 154.79 (C), 153.32 (CH), 152.78 (C), 134.04 (C), 133.09 (C), 132.98 (C), 129.59 (CH), 129.53 (CH), 127.23 (CH), 127.17 (CH), 119.41 (CH), 119.38 (CH), 119.13 (CH), 118.99 (CH), 115.51 (C), 115.29 (CH), 115.12 (CH), 43.69 (CH_2_). HPLC: 1.84 min. HRMS (ESI^+^) *m*/*z* calc. for C_15_H_12_F_2_N_3_ [M+H]^+^ = 272.0994, found 272.1015.

##### Synthesis of 6-Chloro-N-(4-Fluorobenzyl)-4-Quinazolinamine.TFA **5ai**

The title compound was prepared following general procedure Section 4.1.3, from 4,6-dichloroquinazoline **12e** (0.030 g, 0.15 mmol, 1.0 equiv.), 4-fluorobenzylamine (0.026 mL, 0.23 mmol, 1.5 equiv.) and Et_3_N (0.063 mL, 0.45 mmol, 3.0 equiv.) in *i*PrOH (0.37 mL). This yielded, after purification, the desired compound as a white solid with 71% (42.5 mg, 0.11 mmol) yield. ^1^H NMR (500 MHz, DMSO): δ 10.65–10.56 (m, 1H), 8.92 (s, 1H), 8.68–8.65 (m, 1H), 8.09–8.05 (m, 1H), 7.86 (d, *J* = 8.9 Hz, 1H), 7.50–7.44 (m, 2H), 7.21–7.15 (m, 2H), 4.92 (d, *J* = 5.6 Hz, 2H). ^13^C NMR (126 MHz, DMSO): δ 162.56 (C), 160.62 (C), 159.81 (C), 151.92 (CH), 137.63 (C), 135.87 (CH), 133.21 (C), 133.18 (C), 132.34 (C), 129.92 (CH), 129.85 (CH), 123.60 (CH), 122.43 (CH), 115.38 (CH), 115.21 (CH), 114.38 (C), 44.22 (CH_2_). **HPLC**: 2.07 min. **HRMS** (ESI^+^) *m*/*z* calc. for C_15_H_12_ClFN_3_ [M+H; ^35^Cl]^+^ = 288.0698 and [M+H; ^37^Cl]^+^ = 290.0673, found 288.0695 and 290.0682 in the expected 3:1 ratio.

##### Synthesis of N-(4-Fluorobenzyl)-6-Methoxy-4-Quinazolinamine.TFA **5aj**

The title compound was prepared following general procedure Section 4.1.3, from 4-Chloro-6-methoxyquinazoline **12f** (0.030 g, 0.15 mmol, 1.0 equiv.), 4-fluorobenzylamine (0.026 mL, 0.23 mmol, 1.5 equiv.) and Et_3_N (0.065 mL, 0.46 mmol, 3.0 equiv.) in *i*PrOH (0.38 mL). This yielded, after purification, the desired compound as a white solid with 65% (39.8 mg, 0.10 mmol) yield. ^1^H NMR (500 MHz, DMSO): δ 10.33–10.21 (m, 1H), 8.83 (s, 1H), 7.96–7.91 (m, 1H), 7.79–7.74 (m, 1H), 7.70–7.65 (m, 1H), 7.49–7.43 (m, 2H), 7.23–7.16 (m, 2H), 4.95 (d, *J* = 5.6 Hz, 2H) 3.92 (s, 3H). ^13^C NMR (126 MHz, DMSO): δ 162.50 (C), 160.57 (C), 160.02 (C), 158.78 (C), 149.60 (CH), 133.60 (C), 133.57 (C), 129.71 (CH), 129.64 (CH), 126.47 (CH), 121.76 (CH), 115.42 (CH), 115.25 (CH), 114.16 (C), 103.77 (CH), 56.29 (CH_3_), 43.98 (CH_2_). HPLC: 2.06 min. HRMS (ESI^+^) *m*/*z* calc. for C_16_H_15_FN_3_O [M+H]^+^ = 284.1194, found 284.1193.

##### Synthesis of N-(4-Fluorobenzyl)-6-Methyl-4-Quinazolinamine.TFA **5ak**

The title compound was prepared following general procedure Section 4.1.3, from 4-Chloro-6-methylquinazoline **12g** (0.035 g, 0.20 mmol, 1.0 equiv.), 4-fluorobenzylamine (0.034 mL, 0.29 mmol, 1.5 equiv.) and Et_3_N (0.082 mL, 0.59 mmol, 3.0 equiv.) in *i*PrOH (0.5 mL). This yielded, after purification, the desired compound as a white solid with 50% (38.1 mg, 0.10 mmol) yield. ^1^H NMR (500 MHz, DMSO): δ 10.61–10.50 (m, 1H), 8.90 (s, 1H), 8.34 (s, 1H), 7.90 (d, *J* = 8.6 Hz, 1H), 7.75 (d, *J* = 8.6 Hz, 1H), 7.49–7.43 (m, 2H), 7.22–7.15 (m, 2H), 4.93 (d, *J* = 5.7 Hz, 2H), 2.51 (s, 3H). ^13^C NMR (126 MHz, DMSO): δ 162.51 (C), 160.57 (C), 160.30 (C), 150.73 (CH), 138.65 (C), 137.42 (CH), 135.97 (C), 133.42 (C), 129.80 (CH), 129.74 (CH), 123.19 (CH), 119.42 (CH), 115.37 (CH), 115.20 (CH), 112.90 (C), 44.07 (CH_2_), 21.13 (CH_3_). HPLC: 2.05 min. HRMS (ESI^+^) *m*/*z* calc. for C_16_H_15_FN_3_ [M+H]^+^ = 268.1250, found 268.1226.

##### Synthesis of N-(4-Fluorobenzyl)-6-Trifluoromethyl-4-Quinazolinamine.TFA **5al**

The title compound was prepared following general procedure Section 4.1.3 from 4-Chloro-6-trifluoromethyl quinazoline **12h** (0.035 g, 0.15 mmol, 1.0 equiv.), 4-fluorobenzylamine (0.026 mL, 0.23 mmol, 1.5 equiv.) and Et_3_N (0.063 mL, 0.45 mmol, 3.0 equiv.) in *i*PrOH (0.37 mL). This yielded, after purification, the desired compound as a colorless oil with 37% (24.3 mg, 0.06 mmol) yield. ^1^H NMR (500 MHz, DMSO): δ 10.37 (br s, 1H), 8.96 (s, 1H), 8.88 (s, 1H), 8.25 (dd, *J* = 8.8, 1.5 Hz, 1H), 7.94 (d, *J* = 8.8 Hz, 1H), 7.50–7.45 (m, 2H), 7.22–7.16 (m, 2H), 4.91 (d, *J* = 5.7 Hz, 2H). ^13^C NMR (126 MHz, DMSO): δ 162.49 (C), 160.55 (C), 160.33 (C), 154.32 (CH), 133.55 (C), 133.52 (C), 130.58 (CH), 129.82 (CH), 129.76 (CH), 127.19 (q, *J* = 32.8 Hz, CF_3_), 124.73 (C), 123.80 (CH), 122.56 (C), 122.39 (CH), 122.36 (CH), 115.34 (CH), 115.17 (CH), 113.48 (C), 43.98 (CH_2_). HPLC: 2.18 min. HRMS (ESI^+^) *m*/*z* calc. for C_16_H_12_F_4_N_3_ [M+H]^+^ = 322.0967, found 322.0981.

##### Synthesis of N-(4-Fluorobenzyl)-6-Nitro-4-Quinazolinamine.TFA **5am**

The title compound was prepared following general procedure Section 4.1.3, from 4-Chloro-6-nitroquinazoline **12i** (0.036 g, 0.17 mmol, 1.0 equiv.), 4-fluorobenzylamine (0.029 mL, 0.26 mmol, 1.5 equiv.) and Et_3_N (0.072 mL, 0.52 mmol, 3.0 equiv.) in *i*PrOH (0.5 mL). This yielded, after purification, the desired compound as a brown solid with 42% (29.4 mg, 0.07 mmol) yield. ^1^H NMR (500 MHz, DMSO): δ 10.45–10.24 (m, 1H), 9.49 (d, *J* = 2.4 Hz, 1H), 8.83 (s, 1H), 8.62 (dd, *J* = 9.2, 2.4 Hz, 1H), 7.91 (d, *J* = 9.2 Hz, 1H), 7.50–7.44 (m, 2H), 7.22–7.15 (m, 2H), 4.89 (d, *J* = 5.6 Hz, 2H). ^13^C NMR (126 MHz, DMSO): δ 162.45 (C), 160.55 (C), 155.83 (CH), 144.92 (C), 133.76 (C), 129.83 (CH), 129.76 (CH), 128.06 (CH), 125.34 (CH), 121.05 (CH), 115.31 (CH), 115.14 (CH), 113.57 (C), 43.89 (CH_2_). HPLC: 1.95 min. HRMS (ESI^+^) *m*/*z* calc. for C_15_H_12_FN_4_O_2_ [M+H]^+^ = 299.0944, found 299.0900.

##### Synthesis of N-(4-Chlorobenzyl)-6-Fluoro-4-Quinazolinamine.TFA **5an**

The title compound was prepared following general procedure Section 4.1.3, from 4-Chloro-6-fluoroquinazoline **12a** (0.030 g, 0.16 mmol, 1.0 equiv.), 4-chlorobenzylamine (0.030 mL, 0.25 mmol, 1.5 equiv.) and Et_3_N (0.069 mL, 0.49 mmol, 3.0 equiv.) in *i*PrOH (0.40 mL). This yielded, after purification, the desired compound as a colorless oil with 73% (46.7 mg, 0.12 mmol) yield. ^1^H NMR (500 MHz, DMSO): δ 10.40 (br s, 1H), 8.88 (s, 1H), 8.37 (dd, *J* = 9.7, 2.1 Hz, 1H), 7.99–7.88 (m, 2H), 7.46–7.39 (m, 4H), 4.92 (d, *J* = 5.7 Hz, 2H). ^13^C NMR (126 MHz, DMSO): δ 161.12 (C), 160.29 (C), 159.16 (C), 151.67 (CH), 136.73 (C), 136.31 (C), 132.01 (C), 129.49 (CH), 128.46 (CH), 124.74 (CH), 124.54 (CH), 123.90 (CH), 114.52 (C), 114.45 (C), 109.14 (CH), 108.95 (CH), 44.07 (CH_2_). HPLC: 2.14 min. HRMS (ESI^+^) *m*/*z* calc. for C_15_H_12_ClFN_3_ [M+H; ^35^Cl]^+^ = 288.0698 and [M+H; ^37^Cl]^+^ = 290.0673, found 288.0699 and 290.0698 in the expected 3:1 ratio.

##### Synthesis of 6-Fluoro-N-(4-Methoxybenzyl)-4-Quinazolinamine.TFA **5ao**

The title compound was prepared following general procedure Section 4.1.3, from 4-Chloro-6-fluoroquinazoline **12a** (0.040 g, 0.22 mmol, 1.0 equiv.), 4-methoxybenzylamine (0.043 mL, 0.33 mmol, 1.5 equiv.) and Et_3_N (0.092 mL, 0.66 mmol, 3.0 equiv.) in *i*PrOH (0.53 mL). This yielded, after purification, the desired compound as a colorless oil with 48% (42.2 mg, 0.11 mmol) yield. ^1^H NMR (500 MHz, DMSO): δ 10.60–10.54 (m, 1H), 8.91 (s, 1H), 8.42–8.37 (m, 1H), 7.98–7.91 (m, 2H), 7.37–7.32 (m, 2H), 6.92–6.88 (m, 2H), 4.87 (d, *J* = 5.6 Hz, 2H), 3.72 (s, 3H). ^13^C NMR (126 MHz, DMSO): δ 161.26 (C), 160.16 (C), 160.13 (C), 159.30 (C), 158.77 (C), 151.27 (CH), 135.60 (C), 129.26 (CH), 128.89 (C), 124.92 (CH), 124.72 (CH), 123.13 (CH), 123.06 (CH), 114.40 (C), 114.33 (C), 113.93 (CH), 109.39 (CH), 109.19 (CH), 55.12 (CH_3_), 44.44 (CH_2_). HPLC: 1.53 min. HRMS (ESI^+^) *m*/*z* calc. for C_16_H_15_FN_3_O [M+H]^+^ = 284.1194, found 284.1195.

##### Synthesis of 6-Fluoro-N-(4-(Trifluoromethyl)Benzyl)-4-Quinazolinamine.TFA **5ap**

The title compound was prepared following general procedure Section 4.1.3, from 4-Chloro-6-fluoroquinazoline **12a** (0.030 g, 0.16 mmol, 1.0 equiv.), 4-(trifluoromethyl)benzylamine (0.035 mL, 0.25 mmol, 1.5 equiv.) and Et_3_N (0.069 mL, 0.49 mmol, 3.0 equiv.) in *i*PrOH (0.40 mL). This yielded, after purification, the desired compound as a white solid with 84% (58.5 mg, 0.13 mmol) yield. ^1^H NMR (500 MHz, DMSO): δ 10.45 (br s, 1H), 8.86 (s, 1H), 8.38 (dd, *J* = 9.6, 2.4 Hz, 1H), 7.99–7.91 (m, 2H), 7.71 (d, *J* = 8.2 Hz, 2H), 7.63 (d, *J* = 8.2 Hz, 2H), 5.02 (d, *J* = 5.5 Hz, 2H). ^13^C NMR (126 MHz, DMSO): δ 161.13 (C), 160.45 (C), 160.42 (C), 159.17 (C), 151.77 (CH), 142.28 (C), 137.05 (C), 128.24 (CH), 128.00 (q, *J* = 31.8 Hz, CF_3_), 125.41 (CH), 125.38 (CH), 125.35 (CH), 125.32 (CH), 124.72 (CH), 124.52 (CH), 124.19 (CH), 124.12 (CH), 123.18 (C), 114.59 (C), 114.52 (C), 109.12 (CH), 108.93 (CH), 44.31 (CH_2_). HPLC: 2.23 min. HRMS (ESI^+^) *m*/*z* calc. for C_16_H_12_F_4_N_3_ [M+H]^+^ = 322.0962, found 322.0958.

##### Synthesis of 6-Fluoro-N-(Phenylmethyl)-4-Quinazolinamine.TFA **5aq**

The title compound was prepared following general procedure Section 4.1.3, from 4-Chloro-6-fluoroquinazoline **12a** (0.035 g, 0.19 mmol, 1.0 equiv.), benzylamine (0.031 mL, 0.29 mmol, 1.5 equiv.) and Et_3_N (0.080 mL, 0.58 mmol, 3.0 equiv.) in *i*PrOH (0.47 mL). This yielded, after purification, the desired compound as a white solid with 76% (53.1 mg, 0.14 mmol) yield. ^1^H NMR (500 MHz, DMSO): δ 10.60–10.51 (m, 1H), 8.90 (s, 1H), 8.41 (d, *J* = 9.6, 2.3 Hz, 1H), 8.00–7.91 (m, 2H), 7.47–7.26 (m, 5H), 4.95 (d, *J* = 5.7 Hz, 2H). ^13^C NMR (126 MHz, DMSO): δ 161.22 (C), 160.35 (C), 160.32 (C), 159.25 (C), 151.43 (CH), 137.12 (C), 136.05 (C), 128.54 (CH), 127.66 (CH), 127.46 (CH), 124.85 (CH), 124.65 (CH), 123.45 (CH), 123.38 (CH), 114.45 (C), 114.37 (C), 109.30 (CH), 109.10 (CH), 44.83 (CH_2_). HPLC: 1.71 min. HRMS (ESI+) *m*/*z* calc. for C_15_H_13_FN_3_ [M+H]^+^ = 254.1093, found 254.1082.

##### Synthesis of 6-Fluoro-N-(4-Fluorophenyl)-4-Quinazolinamine.TFA **5ar**

The title compound was prepared following general procedure Section 4.1.3, from 4-Chloro-6-fluoroquinazoline **12a** (0.030 g, 0.16 mmol, 1.0 equiv), 4-fluoroaniline (0.023 mL, 0.25 mmol, 1.5 equiv) and Et_3_N (0.069 mL, 0.49 mmol, 3.0 equiv) in *i*PrOH (0.40 mL). This yielded, after purification, the desired compound as a pale yellow solid with 80% (47.5 mg, 0.13 mmol) yield. ^1^H NMR (500 MHz, DMSO): δ 10.98 (br s, 1H), 8.84 (s, 1H), 8.54 (d, *J* = 9.6 Hz, 1H), 7.96 (d, *J* = 6.5 Hz, 2H), 7.82–7.74 (m, 2H), 7.36–7.29 (m, 2H). ^13^C NMR (126 MHz, DMSO): δ 161.14 (C), 160.76 (C), 159.17 (C), 158.97 (C), 158.94 (C), 158.82 (C), 151.92 (CH), 139.15 (C), 133.52 (C), 133.50 (C), 126.05 (CH), 125.98 (CH), 125.42 (CH), 125.35 (CH), 124.60 (CH), 124.40 (CH), 115.64 (CH), 115.46 (CH), 115.01 (C), 114.94 (C), 108.98 (CH), 108.78 (CH). HPLC: 1.71 min. HRMS (ESI^+^) *m*/*z* calc. for C_14_H_10_F_2_N_3_ [M+H]^+^ = 258.0837, found 258.0826.

##### Synthesis of 6-Fluoro-N-[2-(4-Fluorophenyl)Ethyl]-4-Quinazolinamine.TFA **5as**

The title compound was prepared following general procedure Section 4.1.3, from 4-Chloro-6-fluoroquinazoline **12a** (0.030 g, 0.16 mmol, 1.0 equiv.), 4-fluorophenethylamine (0.032 mL, 0.25 mmol, 1.5 equiv.) and Et_3_N (0.069 mL, 0.49 mmol, 3.0 equiv.) in *i*PrOH (0.40 mL). This yielded, after purification, the desired compound as a white solid with 59% (37.8 mg, 0.10 mmol) yield. ^1^H NMR (500 MHz, DMSO): δ 10.10–10.01 (m, 1H), 8.86 (s, 1H), 8.31 (dd, *J* = 9.7, 2.3 Hz, 1H), 7.98–7.88 (m, 2H), 7.35–7.28 (m, 2H), 7.16–7.08 (m, 2H), 3.96–3.87 (m, 2H), 3.00 (t, *J* = 7.3 Hz, 2H). ^13^C NMR (126 MHz, DMSO): δ 161.95 (C), 161.11 (C), 160.16 (C), 160.13 (C), 160.03 (C), 159.14 (C), 151.40 (CH), 136.23 (C), 134.82 (C), 134.80 (C), 130.61 (CH), 130.55 (CH), 124.65 (CH), 124.45 (CH), 123.68 (CH), 123.61 (CH), 115.21 (CH), 115.04 (CH), 114.40 (C), 114.33 (C), 109.05 (CH), 108.85 (CH), 43.15 (CH_2_), 33.03 (CH_2_). HPLC: 2.04 min. HRMS (ESI^+^) *m*/*z* calc. for C_16_H_14_F_2_N_3_ [M+H]^+^ = 286.1150, found 286.1136.

##### Synthesis of N-[2-(4-Chlorophenyl)Ethyl]-6-Fluoro-4-Quinazolinamine.TFA **5at**

The title compound was prepared following general procedure Section 4.1.3, from 4-Chloro-6-fluoroquinazoline **12a** (0.030 g, 0.16 mmol, 1.0 equiv.), 4-chlorophenethylamine (0.035 mL, 0.25 mmol, 1.5 equiv.) and Et_3_N (0.069 mL, 0.49 mmol, 3.0 equiv.) in *i*PrOH (0.40 mL). This yielded, after purification, the desired compound as a white solid with 54% (35.9 mg, 0.09 mmol) yield. ^1^H NMR (500 MHz, DMSO): δ 9.77 (br s, 1H), 8.84 (s, 1H), 8.27 (dd, *J* = 9.6, 2.6 Hz, 1H), 7.95–7.90 (m, 2H), 7.86 (dd, *J* = 9.6, 5.0 Hz, 2H), 3.94–3.87 (m, 2H), 3.00 (t, *J* = 7.2 Hz, 2H). ^13^C NMR (126 MHz, DMSO): δ 161.00 (C), 160.08 (C), 160.05 (C), 159.04 (C), 151.74 (CH), 137.78 (C), 131.05 (C), 130.67 (CH), 128.35 (CH), 124.45 (CH), 124.25 (CH), 114.48 (C), 114.41 (C), 108.84 (CH), 108.64 (CH), 42.83 (CH_2_), 33.18 (CH_2_). HPLC: 2.21 min. HRMS (ESI^+^) *m*/*z* calc. for C_16_H_14_ClFN_3_ [M+H; ^35^Cl]^+^ = 302.0855 and [M+H; ^37^Cl]^+^ = 304.0830, found 302.0843 and 304.0826 in the expected 3:1 ratio.

##### Synthesis of 6-Fluoro-N-[3-(4-Chlorophenyl)Propyl]-4-Quinazolinamine.TFA **5au**

The title compound was prepared following general procedure Section 4.1.3, from 4-Chloro-6-fluoroquinazoline **12a** (0.040 g, 0.22 mmol, 1.0 equiv.), 3-(4-Chlorophenyl)-1-propanamine (0.048 mL, 0.33 mmol, 1.5 equiv.) and Et_3_N (0.092 mL, 0.66 mmol, 3.0 equiv.) in *i*PrOH (0.53 mL). This yielded, after purification, the desired compound as a white solid with 69% (65.1 mg, 0.15 mmol) yield. ^1^H NMR (500 MHz, DMSO): δ 10.09–10.00 (m, 1H), 8.86 (s, 1H), 8.32 (dd, *J* = 9.7, 2.4 Hz, 1H), 7.96–7.88 (m, 2H), 7.31–7.23 (m, 4H), 3.74–3.67 (m, 2H), 2.69 (t, *J* = 7.5 Hz, 2H), 2.00 (p, *J* = 7.5 Hz, 2H). ^13^C NMR (126 MHz, DMSO): δ 161.10 (C), 160.15 (C), 160.12 (C), 159.14 (C), 151.18 (CH), 140.32 (C), 135.63 (C), 130.45 (C), 130.16 (CH), 128.16 (CH), 124.64 (CH), 124.44 (CH), 123.18 (CH), 123.11 (CH), 114.39 (C), 114.32 (C), 109.31 (CH), 109.11 (CH), 41.39 (CH_2_), 31.70 (CH_2_), 29.28 (CH_2_), 29.16 (CH_2_). HPLC: 2.25 min. HRMS (ESI^+^) *m*/*z* calc. for C_17_H_16_ClFN_3_ [M+H; ^35^Cl]^+^ = 316.1017 and [M+H; ^37^Cl]^+^ = 318.0992, found 316.0995 and 318.0993 in the expected 3:1 ratio.

##### Synthesis of 6-Fluoro-N-(3-Fluorophenyl)-4-Quinazolinamine.TFA **5av**

The title compound was prepared following general procedure Section 4.1.3, from 4-Chloro-6-fluoroquinazoline **12a** (0.060 g, 0.33 mmol, 1.0 equiv.), 3-Fluoroaniline (0.047 mL, 0.49 mmol, 1.5 equiv.) and Et_3_N (0.14 mL, 0.99 mmol, 3.0 equiv.) in *i*PrOH (0.80 mL). This yielded, after purification, the desired compound as pale yellow solid with 45% (70.4 mg, 0.14 mmol) yield. ^1^H NMR (500 MHz, DMSO): δ 10.77 (br s, 1H), 8.88 (s, 1H), 8.58–8.53 (m, 1H), 7.99–7.93 (m, 2H), 7.83 (dt, *J* = 11.3, 2.2 Hz, 1H), 7.64–7.60 (m, 1H), 7.53–7.47 (m, 1H), 7.12–7.07 (m, 1H). ^13^C NMR (126 MHz, DMSO): δ 162.88 (C), 161.08 (C), 160.95 (C), 159.12 (C), 158.62 (C), 158.58 (C), 152.24 (CH), 140.69 (C), 139.42 (C), 139.33 (C), 130.39 (CH), 130.32 (CH), 126.54 (CH), 126.47 (CH), 124.44 (CH), 124.24 (CH), 119.07 (CH), 119.05 (CH), 115.26 (C), 115.19 (C), 112.08 (CH), 111.91 (CH), 110.37 (CH), 110.17 (CH), 108.74 (CH), 108.54 (CH). HPLC: 1.81 min. HRMS (ESI^+^) *m*/*z* calc. for C_14_H_10_F_2_N_3_ [M+H]^+^ = 258.0887, found 258.0833.

##### Synthesis of 6-Fluoro-N-(2-Fluorophenyl)-4-Quinazolinamine.TFA **5aw**

The title compound was prepared following general procedure Section 4.1.3, from 4-Chloro-6-fluoroquinazoline **12a** (0.030 g, 0.16 mmol, 1.0 equiv.), 2-fluoroaniline (0.024 mL, 0.25 mmol, 1.5 equiv.) and Et_3_N (0.069 mL, 0.49 mmol, 3.0 equiv.) in *i*PrOH (0.40 mL). This yielded, after purification, the desired compound as a colorless oil with 65% (38.7 mg, 0.10 mmol) yield. ^1^H NMR (500 MHz, DMSO): δ 10.79 (br s, 1H), 8.75 (s, 1H), 8.48–8.43 (m, 1H), 7.99–7.93 (m, 2H), 7.57 (dt, *J* = 7.8, 2.5 Hz, 1H), 7.46–7.37 (m, 2H), 7.35–7.30 (m, 1H). ^13^C NMR (126 MHz, DMSO): δ 161.00 (C), 159.59 (C), 159.03 (C), 157.75 (C), 155.78 (C), 152.52 (CH), 140.91 (C), 128.86 (CH), 128.79 (CH), 128.49 (CH), 126.64 (CH), 124.78 (CH), 124.75 (CH), 124.50 (CH), 124.30 (CH), 116.36 (CH), 116.21 (CH), 114.78 (C), 114.71 (C), 108.61 (CH), 108.42 (CH). HPLC: 1.55 min. HRMS (ESI^+^) *m*/*z* calc. for C_14_H_10_F_2_N_3_ [M+H]^+^ = 258.0837, found 258.0827.

##### Synthesis of 6-Chloro-N-(3-Fluorophenyl)-4-Quinazolinamine.TFA **5ax**

The title compound was prepared following general procedure Section 4.1.3, from 4,6-Dichloroquinazoline **12e** (0.030 g, 0.15 mmol, 1.0 equiv.), 3-fluoroaniline (0.016 mL, 0.17 mmol, 1.5 equiv.) and Et_3_N (0.063 mL, 0.45 mmol, 3.0 equiv.) in *i*PrOH (0.37 mL). This yielded, after purification, the desired compound as a white solid with 63% (36.9 mg, 0.10 mmol) yield. ^1^H NMR (500 MHz, DMSO): δ 10.78 (br s, 1H), 8.87 (s, 1H), 8.82 (s, 1H), 8.06–8.01 (m, 1H), 7.88 (d, *J* = 8.9 Hz, 1H), 7.85–7.81 (m, 1H), 7.65–7.60 (m, 1H), 7.53–7.46 (m, 1H), 7.09 (t, *J* = 8.3 Hz, 1H). ^13^C NMR (126 MHz, DMSO): δ 162.86 (C), 160.93 (C), 157.97 (C), 153.07 (CH), 142.87 (C), 139.48 (C), 139.39 (C), 135.10 (CH), 131.88 (C), 130.36 (CH), 130.29 (CH), 126.07 (CH), 123.11 (CH), 118.98 (CH), 118.95 (CH), 115.34 (C), 111.99 (CH), 111.82 (CH), 110.27 (CH), 110.07 (CH). HPLC: 1.95 min. HRMS (ESI^+^) *m*/*z* calc. for C_14_H_10_ClFN_3_ [M+H; ^35^Cl]^+^ = 274.0542 and [M+H; ^37^Cl]^+^ = 276.0516, found 274.0533 and 276.0521 in the expected 3:1 ratio.

##### Synthesis of N-(3-Fluorophenyl)-6-Methoxy-4-Quinazolinamine.TFA **5ay**

The title compound was prepared following general procedure Section 4.1.3, from 4-Chloro-6-methoxyquinazoline **12f** (0.030 g, 0.15 mmol, 1.0 equiv.), 3-fluoroaniline (0.022 mL, 0.23 mmol, 1.5 equiv.) and Et_3_N (0.065 mL, 0.46 mmol, 3.0 equiv.) in *i*PrOH (0.38 mL). This yielded, after purification, the desired compound as a pale yellow solid with 55% (31.6 mg, 0.08 mmol) yield. ^1^H NMR (500 MHz, DMSO): δ 10.82 (br s, 1H), 8.83 (s, 1H), 8.09 (d, J = 2.6 Hz, 1H), 7.84 (d, *J* = 9.2 Hz, 1H), 7.77 (dt, *J* = 11.1, 2.1 Hz, 1H), 7.71 (dd, *J* = 9.2, 2.6 Hz, 1H), 7.59–7.50 (m, 2H), 7.16–7.11 (m, 1H), 3.98 (s, 3H). ^13^C NMR (126 MHz, DMSO): δ 162.88 (C), 160.95 (C), 158.68 (C), 158.51 (C), 150.00 (CH), 139.19 (C), 139.11 (C), 136.90 (C), 130.41 (CH), 130.34 (CH), 126.38 (CH), 123.97 (CH), 119.75 (CH), 119.73 (CH), 114.95 (C), 112.46 (CH), 112.29 (CH), 111.11 (CH), 110.90 (CH), 103.51 (CH), 56.35 (CH_3_). HPLC: 1.94 min. HRMS (ESI^+^) *m*/*z* calc. for C_15_H_13_FN_3_O [M+H]^+^ = 270.1037, found 270.1020.

##### Synthesis of 6-Fluoro-N-(3-Chlorophenethyl)-4-Quinazolinamine.TFA **5az**

The title compound was prepared following general procedure Section 4.1.3, from 4-Chloro-6-fluoroquinazoline **12a** (0.032 g, 0.17 mmol, 1.0 equiv.), 3-Chlorophenethylamine (0.036 mL, 0.26 mmol, 1.5 equiv.) and Et_3_N (0.073 mL, 0.53 mmol, 3.0 equiv.) in *i*PrOH (0.43 mL). This yielded, after purification, the desired compound as a white solid with 70% (49.7 mg, 0.12 mmol) yield. ^1^H NMR (500 MHz, DMSO): δ 10.01–9.90 (m, 1H), 8.87 (s, 1H), 8.30 (dd, J = 9.6, 2.6 Hz, 1H), 7.99–7.86 (m, 2H), 7.40–7.22 (m, 4H), 3.99–3.89 (m, 2H), 3.02 (t, J = 7.2 Hz, 2H). ^13^C NMR (126 MHz, DMSO): δ 161.55 (C), 160.65 (C), 159.59 (C), 151.97 (CH), 141.77 (C), 136.98 (C), 133.49 (C), 130.72 (CH), 129.13 (CH), 128.03 (CH), 126.87 (CH), 125.08 (CH), 124.88 (CH), 124.36 (CH), 124.29 (CH), 114.92 (C), 114.85 (C), 109.44 (CH), 109.24 (CH), 43.22 (CH_2_), 33.90 (CH_2_). HPLC: 2.20 min. HRMS (ESI^+^) *m*/*z* calc. for C_16_H_14_ClFN_3_ [M+H; ^35^Cl]^+^ = 302.0860 and [M+H; ^37^Cl]^+^ = 304.0835, found 302.0805 and 304.0861 in the expected 3:1 ratio.

##### Synthesis of 6-Fluoro-N-(2-Chlorophenethyl)-4-Quinazolinamine.TFA **5ba**

The title compound was prepared following general procedure Section 4.1.3, from 4-Chloro-6-fluoroquinazoline **12a** (0.035 g, 0.19 mmol, 1.0 equiv.), 2-Chlorophenethylamine (0.040 mL, 0.29 mmol, 1.5 equiv.) and Et_3_N (0.072 mL, 0.52 mmol, 3.0 equiv.) in *i*PrOH (0.47 mL). This yielded, after purification, the desired compound as a white solid with 49% (38.9 mg, 0.09 mmol) yield. ^1^H NMR (500 MHz, DMSO): δ 10.11–10.02 (m, 1H), 8.89 (s, 1H), 8.29 (dd, J = 9.5, 2.5 Hz, 1H), 7.99–7.93 (m, 1H), 7.89 (dd, *J* = 9.5, 5.0 Hz, 1H), 7.47–7.42 (m, 1H), 7.41–7.36 (m, 1H), 7.30–7.24 (m, 2H), 4.00–3.92 (m, 2H), 3.14 (t, *J* = 7.2 Hz, 2H). ^13^C NMR (126 MHz, DMSO): δ 161.13 (C), 160.32 (C), 160.30 (C), 159.16 (C), 151.35 (CH), 136.05 (C), 135.97 (C), 133.23 (C), 131.26 (CH), 129.35 (CH), 128.56 (CH), 127.41 (CH), 124.78 (CH), 124.58 (CH), 123.54 (CH), 123.47 (CH), 114.38 (C), 114.31 (C), 109.08 (CH), 108.89 (CH), 41.44 (CH_2_), 31.75 (CH_2_). HPLC: 2.06 min. HRMS (ESI^+^) *m*/*z* calc. for C_16_H_14_ClFN_3_ [M+H; ^35^Cl]^+^ = 302.0860 and [M+H; ^37^Cl]^+^ = 304.0835, found 302.0865 and 304.0837 in the expected 3:1 ratio.

##### Synthesis of 6-Fluoro-N-[2-(3,4-Dichlorophenyl)Ethyl]-4-Quinazolinamine.TFA **5bb**

The title compound was prepared following general procedure Section 4.1.3, from 4-Chloro-6-fluoroquinazoline **12a** (0.035 g, 0.19 mmol, 1.0 equiv.), 3,4-dichlorophenethylamine (0.043 mL, 0.29 mmol, 1.5 equiv.) and Et_3_N (0.080 mL, 0.58 mmol, 3.0 equiv.) in *i*PrOH (0.47 mL). This yielded, after purification, the desired compound as a white solid with 52% (44.9 mg, 0.10 mmol) yield. ^1^H NMR (500 MHz, DMSO): δ 9.53 (br s, 1H), 8.80 (s, 1H), 8.23 (dd, *J* = 9.7, 2.6 Hz, 1H), 7.92–7.83 (m, 2H), 7.58 (d, *J* = 2.0 Hz, 1H), 7.55 (d, *J* = 8.3 Hz, 1H), 7.27 (dd, *J* = 8.3, 2.0 Hz, 1H), 3.93-3.87 (m, 2H), 3.01 (t, *J* = 7.0 Hz, 2H). ^13^C NMR (126 MHz, DMSO): δ 161.36 (C), 160.48 (C), 159.39 (C), 140.68 (C), 131.36 (CH), 130.93 (CH), 129.82 (CH), 129.45 (C), 124.61 (CH), 124.41 (CH), 115.10 (C), 115.03 (C), 109.06 (CH), 108.87 (CH), 42.87 (CH_2_), 33.38 (CH_2_). HPLC: 2.31 min. HRMS (ESI^+^) *m*/*z* calc. for C_16_H_13_Cl_2_FN_3_ [M+H; ^35^Cl]^+^ = 336.0471 and [M+H; ^37^Cl]^+^ = 338.0443, found 336.0488 and 338.0482 in the expected 3:1 ratio.

##### Synthesis of 6-Fluoro-N-[2-(4-Methoxyphenyl)Ethyl]-4-Quinazolinamine.TFA **5bc**

The title compound was prepared following general procedure Section 4.1.3, from 4-Chloro-6-fluoroquinazoline **12a** (0.030 g, 0.16 mmol, 1.0 equiv.), 4-methoxyphenethylamine (0.036 mL, 0.25 mmol, 1.5 equiv.) and Et_3_N (0.069 mL, 0.49 mmol, 3.0 equiv.) in *i*PrOH (0.40 mL). This yielded, after purification, the desired compound as a white solid with 42% (27.8 mg, 0.07 mmol) yield. ^1^H NMR (500 MHz, DMSO): δ 9.84 (br s, 1H), 8.85 (s, 1H), 8.29 (dd, *J* = 9.5, 2.1 Hz, 1H), 7.96–7.90 (m, 1H), 7.86 (dd, *J* = 9.5, 5.0 Hz, 1H), 7.19 (d, *J* = 8.4 Hz, 2H), 6.86 (d, *J* = 8.4 Hz, 2H), 3.91-3.83 (m, 2H), 3.71 (s, 3H), 2.94 (t, *J* = 7.4 Hz, 2H). ^13^C NMR (126 MHz, DMSO): δ 161.01 (C), 160.02 (C), 159.99 (C), 159.05 (C), 157.88 (C), 151.64 (CH), 130.47 (C), 129.70 (CH), 124.46 (CH), 124.26 (CH), 124.11 (CH), 114.44 (C), 114.37 (C), 113.87 (CH), 108.89 (CH), 108.70 (CH), 55.00 (CH_3_), 43.35 (CH_2_), 33.07 (CH_2_). HPLC: 2.05 min. HRMS (ESI^+^) *m*/*z* calc. for C_17_H_17_F_2_N_3_O [M+H]^+^ = 298.1350, found 298.1347.

##### Synthesis of 6-Fluoro-N-(2-Phenylethyl)-4-Quinazolinamine.TFA **5bd**

The title compound was prepared following general procedure Section 4.1.3, from 4-Chloro-6-fluoroquinazoline **12a** (0.035 g, 0.19 mmol, 1.0 equiv.), phenethylamine (0.036 mL, 0.29 mmol, 1.5 equiv.) and Et_3_N (0.080 mL, 0.58 mmol, 3.0 equiv.) in *i*PrOH (0.47 mL). This yielded, after purification, the desired compound as a white solid with 61% (44.2 mg, 0.12 mmol) yield. ^1^H NMR (500 MHz, DMSO): δ 9.98–9.84 (m, 1H), 8.86 (s, 1H), 8.30 (dd, *J* = 9.6, 2.6 Hz, 1H), 7.96–7.91 (m, 1H), 7.88 (dd, *J* = 9.6, 5.1 Hz, 1H), 7.34–7.26 (m, 4H), 7.25–7.20 (m, 1H), 3.95–3.88 (m, 2H), 3.01 (t, *J* = 7.4 Hz, 2H). ^13^C NMR (126 MHz, DMSO): δ 161.04 (C), 160.07 (C), 160.05 (C), 159.08 (C), 151.59 (CH), 138.64 (C), 136.63 (C), 128.72 (CH), 128.46 (CH), 126.40 (CH), 124.53 (CH), 124.33 (CH), 123.92 (CH), 114.44 (C), 114.36 (C), 108.93 (CH), 108.73 (CH), 43.10 (CH_2_), 33.93 (CH_2_). HPLC: 1.86 min. HRMS (ESI+) *m*/*z* calc. for C_16_H_15_FN_3_ [M+H]^+^ = 268.1250, found 268.1213.

##### Synthesis of 6-Fluoro-N-[2-(4-Methylphenyl)ethyl]-4-Quinazolinamine.TFA **5be**

The title compound was prepared following general procedure Section 4.1.3, from 4-Chloro-6-fluoroquinazoline **12a** (0.035 g, 0.19 mmol, 1.0 equiv.), 4-methylphenethylamine (0.042 mL, 0.29 mmol, 1.5 equiv.) and Et_3_N (0.080 mL, 0.58 mmol, 3.0 equiv.) in *i*PrOH (0.47 mL). This yielded, after purification, the desired compound as a white solid with 46% (34.8 mg, 0.09 mmol) yield. ^1^H NMR (500 MHz, DMSO): δ 9.94 (br s, 1H), 8.88 (s, 1H), 8.30 (dd, *J* = 9.5, 2.6 Hz, 1H), 7.97–7.91 (m, 1H), 7.88 (dd, *J* = 9.5, 5.0 Hz, 1H), 7.18–7.09 (m, 4H), 3.92–3.85 (m, 2H), 2.96 (t, *J* = 7.4 Hz, 2H), 2.26 (s, 3H). ^13^C NMR (126 MHz, DMSO): δ 161.07 (C), 160.07 (C), 159.11 (C), 151.49 (CH), 136.33 (C), 135.49 (C), 135.38 (C), 129.02 (CH), 128.58 (CH), 124.60 (CH), 124.40 (CH), 123.78 (CH), 123.71 (CH), 114.41 (C), 114.34 (C), 108.99 (CH), 108.79 (CH), 43.25 (CH_2_), 33.49 (CH_2_), 20.61 (CH_3_). HPLC: 2.10 min. HRMS (ESI+) *m*/*z* calc. for C_17_H_17_FN_3_ [M+H]^+^ = 282.1407, found 282.1422.

##### Synthesis of 6-Fluoro-N-[2-(4-Bromophenyl)ethyl]-4-Quinazolinamine.TFA **5bf**

The title compound was prepared following general procedure Section 4.1.3, from 4-Chloro-6-fluoroquinazoline **12a** (0.035 g, 0.19 mmol, 1.0 equiv.), 4-bromophenethylamine (0.045 mL, 0.29 mmol, 1.5 equiv.) and Et_3_N (0.080 mL, 0.58 mmol, 3.0 equiv.) in *i*PrOH (0.47 mL). This yielded, after purification, the desired compound as a white solid with 31% (27.0 mg, 0.06 mmol) yield. ^1^H NMR (500 MHz, DMSO): δ 9.97–9.85 (m, 1H), 8.88 (s, 1H), 8.28 (dd, *J* = 9.6, 2.6 Hz, 1H), 7.99–7.92 (m, 1H), 7.88 (dd, *J* = 9.6, 5.0 Hz, 1H), 7.54–7.47 (m, 2H), 7.30–7.23 (m, 2H), 3.95–3.88 (m, 2H), 2.99 (t, *J* = 7.2 Hz, 2H). ^13^C NMR (126 MHz, DMSO): δ 161.09 (C), 160.16 (C), 160.13 (C), 159.12 (C), 151.52 (CH), 138.15 (C), 131.29 (CH), 131.09 (CH), 124.65 (CH), 124.45 (CH), 123.81 (CH), 119.55 (C), 114.42 (C), 114.35 (C), 108.98 (CH), 108.78 (CH), 42.84 (CH_2_), 33.22 (CH_2_). HPLC: 2.22 min. HRMS (ESI^+^) *m*/*z* calc. for C_16_H_14_BrFN_3_ [M+H; ^79^Br]^+^ = 346.0355 and [M+H; ^81^Br]^+^ = 348.0336, found 346.0331 and 348.0348 in the expected 1:1 ratio.

##### Synthesis of 6-Fluoro-N-(4-Fluorobenzyl)-N-Methyl-4-Quinazolinamine.TFA **5bg**

The title compound was prepared following general procedure Section 4.1.3, from 4-Chloro-6-fluoroquinazoline **12a** (0.030 g, 0.16 mmol, 1.0 equiv.), 4-fluoro-*N*-methylbenzylamine (0.033 mL, 0.25 mmol, 1.5 equiv.) and Et_3_N (0.069 mL, 0.49 mmol, 3.0 equiv.) in *i*PrOH (0.40 mL). This yielded, after purification, the desired compound as a yellow oil with 57% (36.6 mg, 0.09 mmol) yield. ^1^H NMR (500 MHz, DMSO): δ 8.85 (s, 1H), 8.16 (dd, *J* = 10.4, 2.3 Hz, 1H), 8.01–7.91 (m, 2H), 7.49–7.44 (m, 2H), 7.25–7.18 (m, 2H), 5.23 (s, 2H), 3.59 (s, 3H). ^13^C NMR (126 MHz, DMSO): δ 162.70 (C), 161.82 (C), 161.79 (C), 160.76 (C), 159.89 (C), 157.94 (C), 148.99 (CH), 138.32 (C), 131.55 (C), 131.52 (C), 129.84 (CH), 129.77 (CH), 124.57 (CH), 124.37 (CH), 122.78 (CH), 122.71 (CH), 115.62 (CH), 115.45 (CH), 113.91 (C), 113.84 (C), 112.98 (CH), 112.78 (CH), 55.37 (CH_2_), 40.87 (CH_3_). HPLC: 2.02 min. HRMS (ESI^+^) *m*/*z* calc. for C_16_H_14_F_2_N_3_ [M+H]^+^ = 286.1150, found 286.1149.

##### Synthesis of 6-Fluoro-N-(4-Chlorophenethyl)-N-Methyl-4-Quinazolinamine.TFA **5bh**

The title compound was prepared following general procedure Section 4.1.3, from 4-Chloro-6-fluoroquinazoline **12a** (0.034 g, 0.19 mmol, 1.0 equiv.), *N*-Methyl-4-chlorophenethylamine (0.047 g, 0.28 mmol, 1.5 equiv.) and Et_3_N (0.078 mL, 0.56 mmol, 3.0 equiv.) in *i*PrOH (0.45 mL). This yielded, after purification, the desired compound as a white solid with 52% (41.6 mg, 0.10 mmol) yield. ^1^H NMR (500 MHz, DMSO): δ 8.76 (s, 1H), 8.12 (dd, *J* = 9.9, 2.6 Hz, 1H), 7.97–7.91 (m, 1H), 7.88 (dd, *J* = 9.9, 5.3 Hz, 1H), 7.37–7.32 (m, 4H), 4.18–4.13 (m, 2H), 3.62 (s, 3H), 3.07–3.02 (m, 2H). ^13^C NMR (126 MHz, DMSO): δ 161.64 (C), 160.22 (C), 158.27 (C), 149.43 (CH), 139.10 (C), 137.78 (C), 131.65 (C), 131.22 (CH), 128.85 (CH), 124.71 (CH), 124.52 (CH), 123.54 (CH), 123.47 (CH), 114.23 (C), 114.16 (C), 113.29 (CH), 113.09 (CH), 55.09 (CH_2_), 41.65 (CH_3_), 31.82 (CH_2_). HPLC: 2.27 min. HRMS (ESI^+^) *m*/*z* calc. for C_17_H_16_ClFN_3_ [M+H; ^35^Cl]^+^ = 316.1017 and [M+H; ^37^Cl]^+^ = 318.0992, found 316.1078 and 318.1075 in the expected 3:1 ratio.

#### 4.1.4. General Procedure for the Suzuki Reaction 

The Suzuki reaction was performed according to the procedure of Liu and co-workers [47]. In a 5 mL microwave vial, a mixture of the organo-iodine compound (0.11 mmol, 1.0 equiv.), phenylboronic acid (0.019 g, 0.16 mmol, 1.5 equiv.), and Na_2_CO_3_ (0.029 mL, 0.27 mmol, 2.5 equiv., 2M, aq) was dissolved in a solvent mixture of toluene and ethanol (8/2, 1 mL). The mixture was purged with argon gas prior addition of tetrakis(triphenylphosphine)palladium (6.5 mg, 0.006 mmol, 0.050 equiv.). After overnight heating at 90 °C, the mixture was cooled to room temperature and the solvent removed under reduced pressure. The crude product was dissolved in methanol and filtered over celite. Afterwards, the solvent was removed under reduced pressure and the desired product was isolated via preparative chromatography (AcN + 0.1% TFA/H_2_O + 0.1% TFA).

##### Synthesis of N-(1, 1’-Biphenyl)-3-yl-6-Fluoro-4-Quinazolinamine.TFA **13a**

The title compound was prepared following general procedure Section 4.1.4, from 6-fluoro-*N*-(3-iodophenyl)-4-quinazolinamine.TFA **8ab** (0.051 g, 0.11 mmol, 1.0 equiv.), phenylboronic acid (0.019 g, 0.16 mmol, 1.5 equiv.), tetrakis(triphenylphosphine)palladium (6.5 mg, 0.006 mmol, 0.05 equiv.) and Na_2_CO_3_ (0.014 mL, 0.26 mmol, 2.5 equiv., 2M, aq) in toluene/ethanol (8/2, 1 mL). This yielded, after purification, the desired compound as a pale yellow solid with 66% (31.1 mg, 0.07 mmol) yield. ^1^H NMR (500 MHz, DMSO): δ 10.83 (br s, 1H), 8.86 (s, 1H), 8.59 (dd, *J* = 9.9, 2.1 Hz, 1H), 8.10–8.08 (m, 1H), 8.00–7.93 (m, 2H), 7.84–7.79 (m, 1H), 7.72–7.68 (m, 2H), 7.60–7.55 (m, 2H), 7.53–7.48 (m, 2H), 7.44–7.39 (m, 1H). ^13^C NMR (126 MHz, DMSO): δ 161.07 (C), 159.11 (C), 158.80 (C), 158.77 (C), 152.23 (CH), 140.77 (C), 139.65 (C), 138.03 (C), 129.41 (CH), 129.06 (CH), 127.80 (CH), 126.68 (CH), 126.13 (CH), 124.41 (CH), 124.21 (CH), 123.99 (CH), 122.54 (CH), 121.91 (CH), 115.20 (C), 115.13 (C), 108.81 (CH), 108.62 (CH). HPLC: 2.34 min. HRMS (ESI^+^) *m*/*z* calc. for C_20_H_15_FN_3_ [M+H]^+^ = 316.1245, found 316.1238.

##### Synthesis of N-(4-Fluorobenzyl)-6-Phenyl-4-Quinazolinamine.TFA **13b**

The title compound was prepared following general procedure Section 4.1.4, from *N*-(4-fluorobenzyl)-6-iodo-4-quinolinamine.TFA **8ac** (0.088 g, 0.18 mmol, 1.0 equiv.), phenylboronic acid (0.033 g, 0.27 mmol, 1.5 equiv.), tetrakis(triphenylphosphine)palladium (10.4 mg, 0.009 mmol, 0.05 equiv.) and Na_2_CO_3_ (0.022 mL, 0.45 mmol, 2.5 equiv., 2M, aq) in a solvent mixture of toluene and ethanol (8/2, 2 mL). This yielded, after purification, the desired compound as a white solid with 69% (55.4 mg, 0.12 mmol) yield. ^1^H NMR (500 MHz, DMSO): δ 10.49 (br s, 1H), 8.89 (s, 1H), 8.84–8.82 (m, 1H), 8.38 (dd, *J* = 8.7, 1.6 Hz, 1H), 7.90–7.83 (m, 3H), 7.59–7.54 (m, 2H), 7.52–7.45 (m, 3H), 7.23–7.17 (m, 2H), 4.97 (d, *J* = 5.8 Hz, 2H). ^13^C NMR (126 MHz, DMSO): δ 162.49 (C), 160.54 (C), 139.72 (C), 138.01 (C), 134.03 (CH), 133.57 (C), 129.74 (CH), 129.67 (CH), 129.20 (CH), 128.58 (CH), 127.05 (CH), 121.38 (CH), 115.38 (CH), 115.21 (CH), 113.68 (C), 44.00 (CH_2_). HPLC: 2.43 min. HRMS (ESI^+^) *m*/*z* calc. for C_21_H_17_FN_3_ [M+H]^+^ = 330.1407, found 330.1426.

#### 4.1.5. General Procedure Sonogashira Reaction 

The procedure of Liu, L. et al. was used to perform the Sonogashira reaction [48]. The organo-iodine compound (0.1 mmol, 1.0 equiv.) was dissolved in THF (1.0 mL) and the solution was purged with argon gas. Subsequently the alkyne (0.15 mmol, 1.5 equiv.), CuI (0.005 mmol, 0.05 equiv., 1 mg), Pd(PPh_3_)_2_Cl_2_ (0.005 mmol, 0.05 equiv., 3.5 mg) and triethylamine (0.2 mmol, 2.0 equiv., 27.9 µL) were added. The mixture was refluxed overnight under argon atmosphere. Then the solvent was removed under reduced pressure and the crude product was dissolved in methanol and filtered over celite. Afterwards, the solvent was removed under reduced pressure and the desired product was isolated via preparative chromatography (AcN + 0.1% TFA/H_2_O + 0.1% TFA).

##### Synthesis of 4-[3-[(6-Fluoro-4-Quinazolinyl)Amino]Phenyl]-3-Butyn-1-ol.TFA **14a**

The title compound was prepared following general procedure Section 4.1.5, from 6-fluoro-*N*-(3-iodophenyl)-4-quinazolinamine.TFA **8ab** (0.048 g, 0.10 mmol, 1.0 equiv.), 3-Butyn-1-ol (0.011 mL, 0.15 mmol, 1.5 equiv.), CuI (1.0 mg, 0.005 mmol, 0.05 equiv.), Pd(PPh_3_)_2_Cl_2_ (3.5 mg, 0.005 mmol, 0.05 equiv.) and triethylamine (27.9 µL, 0.2 mmol, 2 equiv.) in THF (1.0 mL). This yielded, after purification, the desired compound as a pale yellow solid with 62% (26.1 mg, 0.06 mmol) yield. ^1^H NMR (500 MHz, DMSO): δ 10.52 (br s, 1H), 8.83 (s, 1H), 8.54–8.50 (m, 1H), 7.96–7.90 (m, 3H), 7.78–7.73 (m, 1H), 7.43 (t, *J* = 7.9 Hz, 1H), 7.28–7.24 (m, 1H), 3.60 (t, *J* = 6.8 Hz, 2H), 2.58 (t, *J* = 6.8 Hz, 2H). ^13^C NMR (126 MHz, DMSO): δ 160.95 (C), 158.99 (C), 158.42 (C), 152.53 (CH), 137.94 (C), 129.07 (CH), 128.05 (CH), 127.03 (CH), 125.83 (CH), 124.12 (CH), 123.92 (CH), 123.54 (C), 122.77 (CH), 115.28 (C), 115.21 (C), 108.55 (CH), 108.35 (CH), 89.09 (C), 80.65 (C), 59.71 (CH_2_), 23.52 (CH_2_). HPLC: 1.72 min. HRMS (ESI^+^) *m*/*z* calc. for C_18_H_15_FN_3_O [M+H]^+^ = 308.1199, found 308.1187.

##### Synthesis of, 4-[4-[(4-Fluorobenzyl)Amino]-6-Quinazolinyl]-3-Butyn-1-ol.TFA **14b**

The title compound was prepared following general procedure Section 4.1.5, from *N*-(4-fluorobenzyl)-6-iodo-4-quinolinamine.TFA **8ac** (0.049 g, 0.1 mmol, 1.0 equiv.), 3-Butyn-1-ol (0.011 mL, 0.15 mmol, 1.5 equiv.), CuI (1.0 mg, 0.005 mmol, 0.05 equiv.), Pd(PPh_3_)_2_Cl_2_ (3.5 mg, 0.005 mmol, 0.05 equiv.) and triethylamine (27.9 µL, 0.2 mmol, 2.0 equiv.) in THF (1.0 mL). This yielded, after purification, the desired compound as a pale yellow solid with 69% (30.0 mg, 0.069 mmol) yield. ^1^H NMR (500 MHz, DMSO): δ 10.26 (br s, 1H), 8.82 (s, 1H), 8.59–8.56 (m, 1H), 7.94 (dd, *J* = 8.6, 1.5 Hz, 1H), 7.73 (d, *J* = 8.6 Hz, 1H), 7.48–7.43 (m, 2H), 7.21–7.15 (m, 2H), 4.88 (d, *J* = 5.7 Hz, 2H), 3.62 (t, *J* = 6.7 Hz, 2H), 2.62 (t, *J* = 6.7 Hz, 2H). ^13^C NMR (126 MHz, DMSO): δ 162.45 (C), 160.52 (C), 159.69 (C), 152.35 (CH), 137.47 (CH), 133.56 (C), 129.79 (C), 129.72 (CH), 126.68 (CH), 122.55 (C), 115.30 (CH), 115.13 (CH), 113.49 (C), 91.45 (C), 79.68 (C), 59.52 (CH_2_), 43.98 (CH_2_), 23.24 (CH_2_). HPLC: 1.99 min. HRMS (ESI^+^) *m*/*z* calc. for C_19_H_17_FN_3_O [M+H]^+^ = 322.1356, found 322.1376.

#### 4.1.6. Three-step Synthesis of 6-Fluoro-1-[(4-Fluorophenyl)Methyl]-1H-Benzimidazole **17**

##### Synthesis of 4-Fluoro-N-(5-Fluoro-2-Nitrophenyl)-Benzenemethanamine **16**

To a solution of 5-Fluoro-2-nitroaniline **15** (0.573 g, 3.67 mmol, 1.00 equiv.) in DMF (6.3 mL), were added Cs_2_CO_3_ (2.39 g, 7.34 mmol, 2.00 equiv.) and 4-fluorobenzyl bromide (0.686 mL, 5.50 mmol, 1.50 equiv.). The mixture was stirred at room temperature for 2.5 h. Subsequently water (50 mL) was added and the mixture was extracted with EtOAc. The combined organic layers were dried over MgSO_4_, filtered and the solvent removed under reduced pressure. Afterwards, the crude product was purified via Normal Phase silicagel flash chromatography (40 g column, petroleumether/EtOAc), yielding the desired compound as a white solid with 88% (850 mg, 3.22 mmol) yield. ^1^H NMR (250 MHz, CDCl_3_): δ 8.51 (br s, 1H), 8.30–8.20 (m, 1H), 7.37–7.27 (m, 2H), 7.13–7.02 (m, 2H), 6.49–6.36 (m, 2H), 4.48 (d, *J* = 5.3 Hz, 2H). HPLC: 2.95 min. MS(ES+/ES-): Mass not found.

##### Synthesis of 4-Fluoro-N2-[(4-Fluorophenyl)Methyl]-1,2-Benzenediamine 

The benzylated aniline **16** (0.20 g, 0.76 mmol, 1.0 equiv.) was stirred in acetic acid (4.5 mL). The solution was heated at 50 °C prior addition of iron powder (0.17 g, 3.0 mmol, 4.0 equiv.) in one portion. Afterwards, the mixture was heated at 70 °C for 2 h. Acetic acid was removed under reduced pressure. The crude reaction mixture was dissolved in CH_2_Cl_2_ (50 mL) and the organic layer was extracted with 1M HCl (3 × 50 mL). Then the combined aqueous layer was washed with CH_2_Cl_2_, basified to pH 9 using a 1M NaOH solution and extracted with CH_2_Cl_2_. The combined organic phase was washed with brine, dried over MgSO_4_, filtered and the solvent removed under reduced pressure yielding the desired product as a white solid with 69% (123 mg, 0.53 mmol) yield. ^1^H NMR (250 MHz, CDCl_3_): δ 7.40–7.29 (m, 2H), 7.10–6.99 (m, 2H), 6.70–6.60 (m, 1H), 6.41–6.29 (m, 2H), 4.25 (s, 2H), 4.03 (br s, 1H), 3.12 (br s, 2H). HPLC: 2.06 min. MS(ES+/ES-): Mass not found.

##### Synthesis of 6-Fluoro-1-[(4-Fluorophenyl)Methyl]-1H-Benzimidazole **17**

4-Fluoro-*N*2-[(4-fluorophenyl)methyl]-1,2-Benzenediamine (0.050 g, 0.21 mmol, 1.0 equiv.) was stirred with trimethyl orthoformate (0.5 mL) and *p*-toluene sulfonic acid monohydrate (2.0 mg, 0.01 mmol, 0.05 equiv.). The mixture was heated at 100 °C for 4 h. Afterwards, the mixture was concentrated under reduced pressure and purified by preparative chromatography (AcN + 0.1% TFA/H_2_O + 0.1% TFA) yielding the desired compound as a white solid with 88% (45.1 mg, 0.18 mmol) yield. ^1^H NMR (500 MHz, DMSO): δ 9.11 (br s, 1H), 7.83–7.77 (m, 1H), 7.71 (dd, *J* = 9.2, 2.1 Hz, 1H), 7.53–7.48 (m, 2H), 7.29–7.18 (m, 3H), 5.58 (s, 2H). ^13^C NMR (126 MHz, DMSO): δ 162.90 (C), 160.96 (C), 160.43 (C), 158.52 (C), 131.71 (C), 131.69 (C), 130.28 (CH), 130.21 (CH), 118.90 (CH), 118.82 (CH), 115.80 (CH), 115.63 (CH), 112.52 (CH), 112.32 (CH), 98.89 (CH), 98.66 (CH), 47.90 (CH_2_). **HPLC**: 1.91 min. **HRMS** (ESI^+^) *m*/*z* calc. for C_14_H_11_F_2_N_2_ [M+H]^+^ = 245.0885, found 245.0876.

#### 4.1.7. One-step Synthesis of 6-Fluoro-N-(4-Fluorobenzyl)-4-Quinolinamine **19**

##### Synthesis of 6-Fluoro-N-(4-Fluorobenzyl)-4-Quinolinamine.TFA **19**

Into a microwave vial were added, 4-Chloro-6-fluoroquinoline **16** (0.035 g, 0.19 mmol, 1.0 equiv.) and 4-fluorobenzylamine (0.088 mL, 0.77 mmol, 4.0 equiv.). The mixture was heated for 3 h at 160 °C using microwave irradiation. This yielded, after evaporation and purification using preparative chromatography (AcN + 0.1% TFA/H_2_O + 0.1% TFA), the desired compound as a white solid with 64% (46.5 mg, 0.12 mmol) yield. ^1^H NMR (500 MHz, DMSO): δ 9.75–9.69 (m, 1H), 8.55 (d, *J* = 7.0 Hz, 1H), 8.45 (dd, *J* = 10.5, 2.6 Hz, 1H), 8.04–8.00 (m, 1H), 7.95–7.89 (m, 1H), 7.51–7.46 (m, 2H), 7.24–7.18 (m, 2H), 6.84 (d, *J* = 7.0 Hz, 1H), 4.80 (d, *J* = 5.8 Hz, 2H). ^13^C NMR (126 MHz, DMSO): δ 162.54 (C), 160.62 (C), 158.67 (C), 155.19 (C), 155.16 (C), 142.65 (CH), 134.93 (C), 132.73 (C), 132.71 (C), 129.44 (CH), 129.38 (CH), 123.45 (CH), 123.38 (CH), 123.07 (CH), 122.87 (CH), 117.96 (C), 117.89 (C), 115.56 (CH), 115.39 (CH), 108.07 (CH), 107.87 (CH), 98.49 (CH), 45.39 (CH_2_). HPLC: 1.87 min. HRMS (ESI^+^) *m*/*z* calc. for C_16_H_13_F_2_N_2_ [M+H]^+^ = 271.1047, found 271.1058.

#### 4.1.8. Four-step Synthesis of 7-Fluoro-N-(4-Fluorobenzyl)-1-Isoquinoline **24**

##### Synthesis of (E)-N-((Dimethylamino)Methyl)-5-Fluoro-2-Methylbenzamide **21**

The title compound was prepared from 5-Fluoro-2-methylbenzamide **20** (0.600 g, 3.92 mmol, 1.00 equiv.), dissolved in anhydrous THF (6.11 mL). Then *N*,*N*-dimethylformamide dimethyl acetal (0.625 mL, 4.70 mmol, 1.20 equiv.) was added and the mixture was refluxed for 2 h. Subsequently the solvent was removed under reduced pressure and the obtained oil was crystallized from n-hexane, yielding the desired compound as a white, crystalline solid with 74% (0.605 g, 2.91 mmol) yield. ^1^H NMR (250 MHz, CDCl_3_): δ 8.57 (s, 1H), 7.81 (d, *J* = 9.9 Hz, 1H), 7.19–7.11 (m, 1H), 7.06–6.96 (m, 1H), 3.17 (s, 6H), 2.58 (s, 3H). HPLC: 1.28 min. MS(ES+): [M+H]^+^ = 209.

##### Synthesis of 7-Fluoro-1-Isoquinolonone **22**

Potassium *tert*-butoxide (0.808 g, 7.20 mmol, 3.00 equiv.) was added to a solution of (*E*)-*N*-((Dimethylamino)methyl)-5-fluoro-2-methylbenzamide **21** (0.500 g, 2.40 mmol, 1.00 equiv.) in anhydrous DMF (4.00 mL). The mixture was heated at 120 °C for 30 min after which it was poured into water. The pH was adjusted to 5 by addition of 1M HCl (aq). The solvent and water were removed under reduced pressure and the crude product was purified using reversed phase automated flash chromatography (AcN + 0.1% TFA/H_2_O + 0.1% TFA) yielding the desired compound, after lyophilization, as a white solid with 63% (0.245 g, 1.50 mmol) yield. ^1^H NMR (500 MHz, DMSO): δ 11.37 (br s, 1H), 7.83 (dd, *J* = 9.6, 2.7 Hz, 1H), 7.73 (dd, *J* = 8.8, 5.4 Hz, 1H), 7.56 (dt, *J* = 8.8, 2.7 Hz, 1H), 7.15 (d, *J* = 7.1 Hz, 1H), 6.57 (d, *J* = 7.1 Hz, 1H). HPLC: 1.50 min. MS(ES+): [M+H]^+^ = 164.

##### Synthesis of 1-Chloro-7-Fluoroisoquinoline **23**

Into a round bottom flask, equipped with reflux condenser, were added 7-Fluoro-1-isoquinolonone **22** (0.100 g, 0.61 mmol, 1.00 equiv.) and POCl_3_ (2.86 mL, 30.6 mmol, 50.0 equiv.). The mixture was refluxed for 2 h after which the mixture was poured into a saturated NaHCO_3_ solution (aq.). The aqueous layer was extracted using CH_2_Cl_2_ (3 × 50 mL). The combined organic layers were dried over Na_2_SO_4_, filtered and the solvent removed under reduced pressure yielding the desired compound as a pale yellow solid with 91% (101 mg, 0.56 mmol) yield. This compound was used in the next step without further purification. ^1^H NMR (250 MHz, CDCl_3_): δ 8.27 (d, *J* = 5.7 Hz, 1H), 7.96 (dd, *J* = 9.5, 2.5 Hz, 1H), 7.88 (dd, *J* = 9.5, 5.3 Hz, 1H), 7.61 (d, *J* = 5.7 Hz, 1H), 7.54 (dt, *J* = 8.6, 2.5 Hz, 1H). HPLC: 2.13 min. MS(ES+): [M+H]^+^ = 182 and 184.

##### Synthesis of 7-Fluoro-N-(4-Fluorobenzyl)-1-Isoquinoline.TFA **24**

To a mixture of 1-Chloro-7-fluoroisoquinoline **23** (0.050 g, 0.27 mmol, 1.0 equiv.) and 4-fluorobenzylamine (0.047 mL, 0.41 mmol, 1.50 equiv.) in anhydrous DMSO (0.67 mL), was added potassium carbonate (0.076 mL, 0.55 mmol, 2.0 equiv.). The mixture was heated overnight at 120 °C. The crude product was purified using preparative HPLC (AcN + 0.1% TFA/H_2_O + 0.1% TFA), which yielded the desired compound as a white solid with 40% (41.1 mg, 0.11 mmol) yield. ^1^H NMR (500 MHz, DMSO): δ 9.64 (br s, 1H), 8.51–8.44 (m, 1H), 8.06 (dd, *J* = 9.1, 5.7 Hz, 1H), 7.90–7.83 (m, 1H), 7.71 (d, *J* = 6.7 Hz, 1H), 7.51–7.46 (m, 2H), 7.27 (d, *J* = 6.7 Hz, 1H), 7.24–7.18 (m, 2H), 4.81 (d, *J* = 5.6 Hz, 2H). ^13^C NMR (126 MHz, DMSO): δ 162.59 (C), 161.96 (C), 160.66 (C), 160.00 (C), 152.00 (C), 133.66 (C), 132.58 (C), 130.69 (CH), 130.62 (CH), 129.65 (CH), 129.59 (CH), 122.91 (CH), 122.72 (CH), 119.29 (C), 119.22 (C), 115.39 (CH), 115.22 (CH), 110.99 (CH), 109.82 (CH), 109.63 (CH), 44.48 (CH_2_). HPLC: 1.89 min. HRMS (ESI^+^) *m*/*z* calc. for C_16_H_13_F_2_N_2_ [M+H]^+^ = 271.1047, found 271.1058.

#### 4.1.9. Four-step Synthesis of 6-Fluoro-N-(4-Fluorobenzyl)-4-Cinnolinamine 29 

##### Synthesis of 4-Fluoro-2-(2-(Trimethylsilyl)Ethynyl)-Aniline **26**

Into a microwave vial were added, 2-Iodo-4-fluoroaniline **25** (0.651 mL, 5.00 mmol, 1.00 equiv.), copper iodide (0.012 g, 0.063 mmol, 0.01 equiv.), triethylamine (1.39 mL, 2.00 mmol, 10.0 equiv.), ethynyltrimethylsilane (1.04 mL, 7.50 mmol, 1.50 equiv.) and DMF (12.7 mL). The mixture was purged using argon gas prior addition of Bis(triphenylphosphine)palladium(II) dichloride (0.175 g, 0.25 mmol, 0.05 equiv.). The mixture was overnight heated at 50 °C. Then the solvent was removed under reduced pressure and the crude product was filtered over celite (MeOH). Finally, after evaporation of the solvent, the desired product was isolated using normal phase automated flash chromatography (40 g column, petroleumether/EtOAc) as a white solid with 85% (881 mg, 4.25 mmol) yield. ^1^H NMR (250 MHz, CDCl_3_): δ 7.01 (d, *J* = 8.8 Hz, 1H), 6.92–6.81 (m, 1H), 6.63 (dd, *J* = 8.8, 4.3 Hz, 1H), 4.11 (s, 2H), 0.28 (s, 9H). HPLC: 2.80 min. MS(ES+): [M+H]^+^ = 208.

##### Synthesis of 6-FluoroCinnolin-4-ol **27**

Into a round bottom flask, equipped with reflux condenser, 4-fluoro-2-(2-(trimethylsilyl)ethynyl)-benzenamine **26** (0.829 g, 4.00 mmol, 1.00 equiv.) was dissolved in distilled water (8.00 mL). Afterwards 6N HCl (7.00 mL, 42.0 mmol, 10.5 equiv., aq.) was added followed by the dropwise addition of sodium nitrite (0.414 g, 6.00 mmol, 1.50 equiv.) dissolved in water (1.9 mL). The mixture was heated at 100 °C for 3 h, cooled to room temperature and quenched into a saturated NaHCO_3_ solution (aq.). The obtained solid was isolated via filtration, washed with H_2_O an CH_2_Cl_2_ and dried under reduced pressure. The desired product was obtained after reversed phase flash column chromatography (H_2_O + 0.1% TFA/can + 0.1% TFA) as a white solid with 28% (187 mg, 1.14 mmol) yield. ^1^H NMR (500 MHz, DMSO): δ 7.76–7.68 (m, 4H). HPLC: 1.34 min. MS(ES+): [M+H]^+^ = 165.

##### Synthesis of 4-Chloro-6-Fluoro-Cinnoline **28**

The title compound was prepared following general procedure Section 4.1.2, from 6-Fluoro-4-cinnolinol **27** (0.070 g, 0.43 mmol, 1.0 equiv.) in SOCl_2_ (1.6 mL). This yielded, after work-up, the desired compound as a pale yellow solid with 98% (76.3 mg, 0.42 mmol) yield. ^1^H NMR (250 MHz, CDCl_3_): δ 9.25 (s, 1H), 8.56 (dd, *J* = 9.0, 5.1 Hz, 1H), 7.73 (dd, *J* = 9.0, 2.4 Hz, 1H), 7.72–7.63 (m, 1H). HPLC: 1.86 min. MS(ES+): [M+H;^35^Cl]^+^ = 183 and [M+H;^37^Cl]^+^ = 185.

##### Synthesis of 6-Fluoro-N-(4-Fluorobenzyl)-4-Cinnolinamine.TFA **29**

The title compound was prepared following general procedure Section 4.1.3, from 4-Chloro-6-fluorocinnoline **28** (0.036 g, 0.20 mmol, 1.0 equiv.), 4-fluorobenzylamine (0.034 mL, 0.30 mmol, 1.5 equiv.) and Et_3_N (0.084 mL, 0.60 mmol, 3.0 equiv.) in *i*PrOH (0.49 mL). This yielded, after purification, the desired compound as a pale brown oil with 55% (42.1 mg, 0.11 mmol) yield. ^1^H NMR (500 MHz, DMSO): δ 10.36–10.17 (m, 1H), 8.78 (s, 1H), 8.43 (dd, *J* = 9.6, 2.6 Hz, 1H), 8.11 (dd, *J* = 9.6, 5.1 Hz, 1H), 8.06-8.00 (m, 1H), 7.57–7.51 (m, 2H), 7.27–7.21 (m, 2H), 4.95 (d, *J* = 3.9 Hz, 2H). ^13^C NMR (126 MHz, DMSO): δ 162.72 (C), 161.54 (C), 160.78 (C), 159.55 (C), 146.74 (C), 137.75 (C), 132.26 (C), 132.23 (C), 129.87 (CH), 129.81 (CH), 128.41 (CH), 125.39 (CH), 125.17 (CH), 123.23 (CH), 123.15 (CH), 116.74 (C), 116.66 (C), 115.65 (CH), 115.48 (CH), 107.18 (CH), 106.98 (CH), 45.51 (CH_2_). HPLC: 1.81 min. HRMS (ESI^+^) *m*/*z* calc. for C_15_H_12_F_2_N_3_ [M+H]^+^ = 272.0999, found 272.1003.

#### 4.1.10. Four-Step Synthesis of 7-Fluoro-N-(4-Fluorobenzyl)-2-Quinoxalinamine 34

##### Synthesis of Ethyl 2-(4-Fluoro-2-Nitrophenylamino)Acetate **31**

Into a flame-dried round bottom flask were added 4-Fluoro-2-nitroaniline **30** (1.00 g, 6.41 mmol, 1.00 equiv.), Cs_2_CO_3_ (3.34 g, 10.2 mmol, 1.60 equiv.) and ethyl bromoacetate (2.81 mL, 32.0 mmol, 5.00 equiv.). The mixture was heated overnight at 135 °C under inert atmosphere. Subsequently the obtained mixture was added to a 1N NaOH solution (aq.). The aqueous phase was extracted using CH_2_Cl_2_ (3 × 50 mL). The combined organic layers were dried over Na_2_SO_4_, filtered and the solvent removed under reduced pressure. The crude product was subjected to reversed phase flash column chromatography (H_2_O/AcN) yielding the desired compound as a white solid with 45% (0.70 g, 2.89 mmol) yield. ^1^H NMR (250 MHz, CDCl_3_): δ 8.29 (br s, 1H), 7.93 (d, *J* = 9.0 Hz, 1H), 7.33–7.22 (m, 1H), 6.69 (dd, *J* = 9.0, 4.4 Hz, 1H), 4.29 (q, *J* = 7.2 Hz, 2H), 4.09 (d, *J* = 5.2 Hz, 2H), 1.33 (t, *J* = 7.2 Hz, 3H). HPLC: 2.39 min. MS(ES+): [M+H]^+^ = 243.

##### Synthesis of 7-Fluoro-1H-Quinoxalin-2-One **32**

To a solution of Ethyl 2-(4-fluoro-2-nitrophenylamino)acetate **31** (0.250 g, 1.04 mmol, 1.00 equiv.) in methanol (20.0 mL) was added Pd/C (0.222 g, 0.21 mmol, 0.20 equiv., 10 wt%). Then H_2_-gas was bubbled for 1 h to the stirred mixture at room temperature. Subsequently Argon gas was bubbled through the mixture and the mixture was stirred for 2 days at room temperature. After filtration of the mixture over celite using methanol, the solvent was removed under reduced pressure. The crude product was triturated with CH_2_Cl_2_ to obtain the desired product with 46% (79.1 mg, 0.48 mmol) yield. The product was used in the next step without further purification. ^1^H NMR (500 MHz, DMSO): δ 8.12 (d, *J* = 2.2 Hz, 1H), 7.84 (dd, *J* = 8.9, 5.8 Hz, 1H), 7.17 (dt, *J* = 8.9, 2.8 Hz, 1H), 7.03 (dd, *J* = 9.5, 2.8 Hz, 1H). HPLC: 1.40 min. HPLC: 1.40 min. MS(ES+): [M+H]^+^ = 165.

##### Synthesis of 2-Chloro-7-Fluoro-Quinoxaline **33**

The title compound was prepared following general procedure Section 4.1.2, from 7-Fluoro-1*H*-quinoxalin-2-one **32** (0.050 g, 0.30 mmol, 1.0 equiv.) and SOCl_2_ (1.1 mL). This yielded, after work-up, the desired compound as a white solid with 98% (53.8 mg, 0.29 mmol) yield. ^1^H NMR (250 MHz, CDCl_3_): δ 8.76 (s, 1H), 8.13 (dd, *J* = 9.3, 5.9 Hz, 1H), 7.77–7.50 (m, 2H). HPLC: 2.08 min. MS(ES+): [M+H;^35^Cl]^+^ = 183 and [M+H;^37^Cl]^+^ = 185.

##### Synthesis of 7-Fluoro-N-(4-Fluorobenzyl)-2-Quinoxalinamine.TFA **34**

The title compound was prepared following general procedure Section 4.1.3, from 2-Chloro-7-fluoroquinoxaline **33** (0.040 g, 0.22 mmol, 1.0 equiv.), 4-fluorobenzylamine (0.038 mL, 0.33 mmol, 1.5 equiv.) and Et_3_N (0.092 mL, 0.66 mmol, 3.0 equiv.) in *i*PrOH (0.53 mL). This yielded, after purification, the desired compound as a pale yellow solid with 59% (35.3 mg, 0.13 mmol) yield. ^1^H NMR (500 MHz, DMSO): δ 8.33 (s, 1H), 8.31–8.26 (m, 1H), 7.81 (dd, *J* = 9.1, 6.2 Hz, 1H), 7.47–7.42 (m, 2H), 7.27 (dd, *J* = 10.4, 2.8 Hz, 1H), 7.23–7.13 (m, 3H), 4.60 (d, *J* = 5.5 Hz, 2H). ^13^C NMR (126 MHz, DMSO): δ 163.49 (C), 162.26 (C), 161.53 (C), 160.33 (C), 152.36 (C), 142.84 (C), 142.73 (C), 139.37 (CH), 135.15 (C), 133.50 (C), 130.61 (CH), 130.52 (CH) 129.72 (CH), 129.65 (CH), 115.16 (CH), 114.99 (CH), 112.52 (CH), 112.32 (CH), 109.73 (CH), 109.56 (CH), 42.91 (CH_2_). HPLC: 2.37 min. HRMS (ESI^+^) *m*/*z* calc. for C_15_H_12_F_2_N_3_ [M+H]^+^ = 272.0999, found 272.1003.

### 4.2. NSCLC Cells Viability Assay

PC9 (CVCL_B260; Merck) cells were cultured in RPMI 1640 (Gibco) supplemented with 10% FBS (Greiner). The cells were cultured according to standard procedures in a humid incubator at 37 °C and 5% CO_2_. 

PC9 cells were seeded at a cell density of 500 cells/well in clear bottom 384 well plates (Greiner Bio-One) in a total volume of 50 µL per well. Compounds were added 24 h post seeding and the viability was measured using the ATP-based luminescence CellTiter-Glo assay (Promega Corporation, 2800 Woods Hollow Road, Madison, WI 53711 USA) upon 72 h incubation with the compounds. Luminescence output was measured using a Spectramax M3 (Molecular Devices, LLC., 3860 N First Street, San Jose, CA 95134 USA). Absolute values were normalized to the mean of the control conditions. An identical setup was used to determine the IC_50_ values. PC9 cells were treated with the indicated compounds at concentrations ranging between 0 and 5 µM (as indicated in the ESI). Cell viability was measured using CellTiter Glo upon 72 h of treatment. Data was plotted and IC_50_ values were calculated based on a sigmoidal, 4PL standard curve interpolation using Graphpad Prism version 8.4.2 (GraphPad Software, 2365 Northside Dr. Suite 560, San Diego, CA 92108).

### 4.3. Kinase Screening

Eurofins performed a “Full KP Panel [Km ATP], KinaseProfile” which contains 429 radiometric kinase activity assays. The remaining kinase activity (%) was determined after treatment with the compounds at a concentration of 10 µM (Cfr. supporting information). 

### 4.4. ITC

Pure and untagged USP13 protein was produced for the purpose of analysing ligand binding. The pGEX4T2-USP13 vector encoding for an N-terminal GST tag fused to full-length human USP13 was transformed into *E. coli* BL21(DE3) cells for expression. Cells were grown at 37 °C in Terrific Broth (TB) medium, supplemented with 100 µg/mL Ampicillin, until the OD595 reached 0.7–0.8. Expression was then induced with 1 mM Isopropyl β-D-1-thiogalactopyranoside (IPTG). After 6 h induction at 28 °C, cells were collected by centrifugation and resuspended in PBS buffer. The cells were lysed with a cell disruptor (Constant Systems) and centrifuged to remove cell debris. The cell lysate was added to glutathione sepharose resin beads (17-0756-01, GE healthcare) equilibrated in PBS. Washing steps with PBS were performed to remove all other proteins before proceeding. To cleave the GST-tag, 10U/mL Thrombin was added to the beads and incubated at 4 °C on a shaker at slow rocking speed for 6 h. USP-13 was subsequently eluted in PBS and further purified on size exclusion chromatography, using a Superdex 200 16/900 column (GE Healthcare Life Sciences) in protein buffer (20 mM Tris-HCl (pH = 8), 50 mM NaCl, 0.1 mM EDTA), supplemented with 0.5 mM DTT and 5% glycerol for storage purposes. 

An isothermal titration calorimetry (ITC) experiment was performed with the MicroCal iTC200 (GE Healthcare) at 25 °C. Spautin-1 was dissolved in DMSO and diluted in the protein buffer (25 mM Tris pH 7.5; 150 mM NaCl; 0.1 mM EDTA) to an end concentration of 300 µM with 5% DMSO. The compound was added to the syringe. The sample cell was filled with 30 µM of the protein in buffer to which 5% DMSO was added to avoid buffer mismatch. A control titration of buffer-buffer and compound-buffer was performed according to the same protocol. Data analysis was done with the Origin software accompanying the ITC instrument (Origin 7, OriginLab Corporation, Northampton, MA, USA). 

### 4.5. TSA

The melting temperature (T_m_) of the protein sample can be determined by following the fluorescence of SYPRO orange with a CFX Connect real-time PCR instrument (Bio-Rad, Hercules, CA, USA). Samples contained 0.4 mg/mL USP13 protein in 25 mM Tris Ph 7.5; 150 mM NaCl; 0.1 mM EDTA; 0.5 mM DTT. Compounds were added to the protein and incubated for 30 min before adding 20× SYPRO Orange Protein Gel Stain (Thermo Fisher Scientific, Waltham, MA, USA). Control measurements were done for the protein in the corresponding percentages of DMSO. 

Fluorescence was measured while increasing the temperature from 10 °C to 85 °C in 0.5 °C/30 s increments. The melting temperature for each protein sample could be determined from the relative fluorescence versus temperature curve by deleting post-peak quenching data and subsequently fitting the Boltzmann–sigmoidal equation, using Graphpad Prism software version 8.4.2 (GraphPad Software, 2365 Northside Dr. Suite 560, San Diego, CA 92108). 

Due to lack of protein material, the measurements were only performed once in a screening setup.

## Figures and Tables

**Table 1:** Melting temperatures, Tm (°C), and the thermal shift, ΔTm (°C), are given for each sample analyzed in a thermal shift assay. The melting temperatures were derived by fitting a Boltzmann–sigmoidal equation to the data. To determine a temperature difference (ΔTm), the reference condition was the protein in buffer with the corresponding percentage of DMSO.

Sample	T_m_ (°C)	ΔT_m_ (°C)
USP13 in buffer	44.56	N.D.
USP13 in 5% DMSO	45.18	0.00
USP13 in 10% DMSO	44.80	0.00
USP13 + 50 μM of **5aa** in 5% DMSO	45.79	0.61
USP13 + 100 μM of **5aa** in 10% DMSO	45.86	1.06
USP13 + 100 μM of **5aa** in 10% DMSO	44.44	−0.36
USP13 + 50 μM of **5bc** in 5% DMSO	46.58	1.40
USP13 + 100 μM of **5bc** in 10% DMSO	43.27	−1.53
USP13 + 100 μM of **5bc** in 10% DMSO	44.44	−0.36

**Table 2:** Kinases which had a residual activity < 60% for at least one of the analogues at a 10 µM concentration (highlighted in red).

	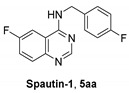	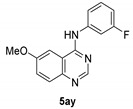	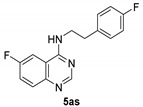	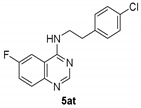
Kinase	Residual Activity (%)			
AAK1 (h)	95	53	81	72
Abl (m)	90	56	83	99
ACK1 (h)	85	56	84	71
ALK (h)	82	72	59	86
ALK2 (h)	60	50	45	58
Aurora-B (h)	85	59	69	48
BrSK1 (h)	85	30	89	112
BrSK2 (h)	79	42	65	71
CaMKIIα (h)	94	58	88	89
CaMKIIγ (h)	83	41	74	75
CaMKIIδ (h)	90	41	79	77
CDK2/cyclinE (h)	69	89	103	54
CDK7/cyclinH/ MAT1 (h)	62	58	49	59
CDK13/cyclinK(h)	75	105	35	114
CDKL3 (h)	77	29	65	71
CDKL4 (h)	73	38	94	92
CLK1 (h)	23	14	28	28
CLK2 (h)	47	43	53	60
CLK4 (h)	19	6	12	16
DCAMKL2 (h)	81	80	64	57
DDR1 (h)	65	7	29	39
DRAK1 (h)	98	57	79	85
DYRK1A (h)	74	29	68	63
DYRK1B (h)	88	53	82	92
DYRK3 (h)	117	57	98	98
EGFR (h)	27	1	46	72
EGFR (L858R) (h)	23	3	37	60
EGFR (L861Q) (h)	36	1	51	73
EphB4 (h)	68	63	59	68
ErbB2 (h)	74	20	79	65
Flt3 (D835Y) (h)	91	49	87	80
Haspin (h)	71	36	72	78
Hck (h) activated	85	46	85	108
HIPK4 (h)	103	42	92	93
LOK (h)	91	35	81	87
LRRK2 (h)	91	44	91	101
Met (h)	82	72	57	112
Mnk2 (h)	93	19	83	80
MST4 (h)	58	64	81	72
NEK4 (h)	71	30	27	12
NEK11 (h)	41	53	71	45
PASK (h)	66	65	44	30
PDGFRα (D842V) (h)	114	50	92	86
Pim-1 (h)	66	39	73	70
Ret (h)	69	43	99	62
RIPK2 (h)	82	22	86	86
TAF1L (h)	80	32	66	70
TRB2 (h)	69	31	65	60
PI3KC2g (h)	70	40	57	34

## Data Availability

All data are available upon reasonable request.

## Supplementary Materials

Supplementary Materials can be found at https://www.mdpi.com/1422-0067/22/2/635/s1.

## Abbreviations

SAR	Structure–activity relationship
PACS	Pathophysiological cell signaling
USP13	Ubiquitin Specific Protease 13
EGFR	Epidermal growth factor receptor
NSCLC	Non-small cell lung cancer
ITC	Isothermal titration calorimetry
TSA	Thermal shift assay
DUBs	Deubiquitinating enzymes
NEK4	Never in mitosis A related kinase 4
TRAIL	Tumor necrosis factor-Related Apoptosis Inducing Ligand
EMT	Epithelial to mesenchymal transition
ON	Overnight
DMF	*N*,*N*-dimethylformamide
T_m_	Melting temperature
ΔT_m_	Melting temperature difference
h	Human
TB	Terrific Broth
IPTG	Isopropyl β-*D*-1-thiogalactopyranoside
EDTA	Ethylenediaminetetraacetic acid
DTT	1,4-dithiothreitol
IRP	Interdisciplinary Research Programmes
WFWG	Wetenschappelijk Fonds Willy Gepts
VUB	Vrije Universiteit Brussel
