# Supplementary material for: Structure–Activity Relationship (SAR) Study of Spautin-1 to Entail the Discovery of Novel NEK4 Inhibitors
Source: Int J Mol Sci. 2021 Jan 10;22(2):635. doi: 10.3390/ijms22020635 (PMC7827406; doi:10.3390/ijms22020635)
Supplement: Supplementary file 1 [file ijms-22-00635-s001.pdf]

Supplementary data for the paper entitled:

Structure-activity relationship (SAR) study of spautin-1 to entail the discovery of novel NEK4 inhibitors

Mathias Elsocht [1], Philippe Giron [2,5], Laila Maes [3,4], Wim Versées [3,4], Gustavo J. Gutierrez [5], Jacques De Grève [2], Steven Ballet [1,*]

[1] Research Group of Organic Chemistry, Faculty of Sciences and Bioengineering Sciences, Vrije Universiteit Brussel, Pleinlaan 2, 1050 Brussels, Belgium; Mathias.Elsocht@vub.be

[2] Laboratory of Medical and Molecular Oncology and Center of Medical Genetics, Faculty of Medicine and Pharmacy, Vrije Universiteit Brussel, Laarbeeklaan 103, 1090 Brussels, Belgium; Jacques.DeGreve@uzbrussel.be

[3] VIB-VUB Center for Structural Biology, Pleinlaan 2, 1050 Brussels, Belgium; Wim.Versees@vub.be

[4] Structural Biology Brussels, Vrije Universiteit Brussel, Pleinlaan 2, 1050 Brussels, Belgium; Wim.Versees@vub.be

[5] Laboratory of Pathophysiological Cell Signalling (PACS), Department of Biology, Faculty of Sciences and Bioengineering Sciences, Vrije Universiteit Brussel, Pleinlaan 2, 1050 Brussels, Belgium; Gustavo.Gutierrez.Gonzalez@vub.be

* Correspondence: Steven.Ballet@vub.be; Tel.: +32-2-6293292

Table of content

Kinase screening S2

Determination IC_{50} values for NSCLC cells S11

ITC data S13

TSA data S13

Expression of NEK4 in lung cancer cell lines S14

Determination IC_{50} values for NEK4 .... S16

NMR spectra S18

Kinase screening

A kinase screening was performed for compounds **spautin-1**, **5ay**, **5as** and **5at** (**Table 1**). The KINOMEscan from Eurofins consists of a competitive binding assay where the DNA-tagged kinase was incubated with one of the compounds in the presence of immobilized active site binding ligands. Binding of the compounds to the kinase hampers the binding of the kinase to the ligands which was determined by a quantitative polymerase chain reaction. The residual kinase activity, which represents the percentage of kinase attached to the immobilized ligands, was determined for 428 kinases at a concentration of 10 μM of the compounds dissolved in DMSO relative to DMSO.

Table S1. *Residual kinase activity at 10 μM of Spautin-1 and analogues **5ay**, **5as** and **5at** (Kinases which had a residual activity < 60% were highlighted).*

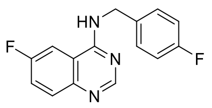

Spautin-1, **5aa**

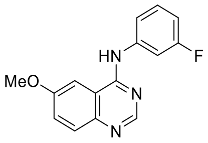

5ay

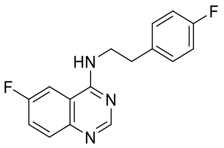

5as

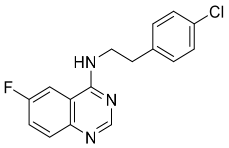

5at

Kinase	Residual activity (%)			
	Spautin-1, 5aa	5ay	5as	5at
AAK1(h)	95	53	81	72
Abl(h)	83	74	91	96
Abl(m)	90	56	83	99
Abl (H396P) (h)	95	76	86	80
Abl (M351T)(h)	93	76	73	69
Abl (Q252H) (h)	104	80	104	88
Abl(T315I)(h)	73	91	101	80
Abl(Y253F)(h)	106	66	91	71
ACK1(h)	85	56	84	71
ACTR2(h)	92	85	83	97
ALK(h)	82	72	59	86
ALK1(h)	104	96	85	93
ALK2(h)	60	50	45	58
ALK4(h)	121	73	84	61
ALK6(h)	94	87	86	92
Arg(h)	79	73	82	81
AMPKα1(h)	110	100	93	85
AMPKα2(h)	108	97	108	109
A-Raf(h)	103	100	106	108
Arg(m)	91	83	105	98
ARK5(h)	99	93	101	105
ASK1(h)	95	99	85	80
Aurora-A(h)	81	79	87	70
Aurora-B(h)	85	59	69	48
Aurora-C(h)	120	106	102	118
Axl(h)	62	64	90	75

**BIKe(h)**	95	67	98	88
**Blk(h)**	97	85	100	91
**Blk(m)**	91	102	100	98
**BMPR2(h)**	115	99	108	103
**Bmx(h)**	79	77	78	78
**BRK(h)**	105	71	87	96
**BrSK1(h)**	85	30	89	112
**BrSK2(h)**	79	42	65	71
**BTK(h)**	123	126	113	96
**BTK(R28H)(h)**	88	103	103	101
**B-Raf(h)**	90	82	83	92
**B-Raf(V599E)(h)**	125	98	99	101
**CaMKI(h)**	90	93	60	104
**CaMKIß(h)**	85	92	84	81
**CaMKIγ(h)**	95	94	94	87
**CaMKIIα(h)**	94	58	88	89
**CaMKIIβ(h)**	81	72	91	91
**CaMKIIγ(h)**	83	41	74	75
**CaMKIδ(h)**	91	89	90	92
**CaMKIIδ(h)**	90	41	79	77
**CaMKIV(h)**	81	68	75	81
**CaMKK1(h)**	96	90	99	91
**CaMKK2(h)**	79	78	87	96
**Cdc7/cyclinB1(h)**	92	73	79	76
**CDK1/cyclinB(h)**	87	72	90	91
**CDK2/cyclinA(h)**	95	83	82	86
**CDK2/cyclinE(h)**	69	89	103	54
**CDK3/cyclinE(h)**	93	101	105	107
**CDK4/cyclinD3(h)**	101	100	97	95
**CDK5/p25(h)**	73	80	89	86
**CDK5/p35(h)**	87	89	87	87
**CDK6/cyclinD3(h)**	102	87	96	98
**CDK7/cyclinH/MAT1(h)**	62	58	49	59
**CDK9/cyclin T1(h)**	104	100	93	86
**CDK12/cyclinK(h)**	84	69	72	93
**CDK13/cyclinK(h)**	75	105	35	114
**CDK14/cyclinY(h)**	107	93	103	109
**CDK16/cyclinY(h)**	90	81	114	104
**CDK17/cyclinY(h)**	82	74	80	88
**CDK18/cyclinY(h)**	87	86	102	116
**CDKL1(h)**	94	119	111	111
**CDKL2(h)**	87	67	103	67
**CDKL3(h)**	77	29	65	71
**CDKL4(h)**	73	38	94	92
**ChaK1(h)**	86	77	81	82
**CHK1(h)**	80	76	69	73
**CHK2(h)**	67	67	73	75
**CHK2(I157T)(h)**	88	91	105	99
**CHK2(R145W)(h)**	101	87	90	91
**CK1α(h)**	76	100	98	89

**CK1ε(h)**	87	78	76	82
**CK1γ1(h)**	99	94	80	80
**CK1γ2(h)**	84	62	72	76
**CK1γ3(h)**	81	83	87	73
**CK1δ(h)**	74	86	80	80
**CK1(y)**	95	80	101	81
**CK2(h)**	83	75	78	87
**CK2α1(h)**	105	75	84	78
**CK2α2(h)**	101	83	95	86
**CLIK1(h)**	97	64	74	77
**CLK1(h)**	23	14	28	28
**CLK2(h)**	47	43	53	60
**CLK3(h)**	88	114	117	116
**CLK4(h)**	19	6	12	16
**cKit(h)**	93	83	78	71
**cKit(D816V)(h)**	105	125	111	98
**cKit(D816H)(h)**	115	89	95	95
**cKit(V560G)(h)**	85	106	105	90
**cKit(V654A)(h)**	112	92	85	85
**CRIK(h)**	90	76	101	89
**CSK(h)**	82	91	85	79
**c-RAF(h)**	110	102	114	117
**cSRC(h)**	133	106	81	120
**DAPK1(h)**	102	95	90	89
**DAPK2(h)**	94	91	100	101
**DCAMKL1(h)**	95	84	82	89
**DCAMKL2(h)**	81	80	64	57
**DCAMKL3(h)**	102	108	108	107
**DDR1(h)**	65	7	29	39
**DDR2(h)**	87	84	80	82
**DMPK(h)**	79	99	90	80
**DRAK1(h)**	98	57	79	85
**DRAK2(h)**	88	65	73	88
**DYRK1A(h)**	74	29	68	63
**DYRK1B(h)**	88	53	82	92
**DYRK2(h)**	99	64	100	103
**DYRK3(h)**	117	57	98	98
**eEF-2K(h)**	87	79	85	85
**EGFR(h)**	27	1	46	72
**EGFR(L858R)(h)**	23	3	37	60
**EGFR(L861Q)(h)**	36	1	51	73
**EGFR(T790M)(h)**	90	65	87	74
**EGFR(T790M,L858R)(h)**	96	69	93	85
**EphA1(h)**	93	61	66	74
**EphA2(h)**	73	75	90	99
**EphA3(h)**	116	110	108	94
**EphA4(h)**	89	86	83	80
**EphA5(h)**	90	95	99	87
**EphA7(h)**	100	90	84	87
**EphA8(h)**	87	84	88	71

**EphB2(h)**	96	97	82	93
**EphB1(h)**	84	88	90	95
**EphB3(h)**	93	84	79	76
**EphB4(h)**	68	63	59	68
**ErbB2(h)**	74	20	79	65
**ErbB4(h)**	90	74	84	79
**FAK(h)**	84	84	88	83
**Fer(h)**	76	80	98	94
**Fes(h)**	91	93	92	81
**FGFR1(h)**	91	81	105	123
**FGFR1(V561M)(h)**	123	108	100	95
**FGFR2(h)**	91	96	99	84
**FGFR2(N549H)(h)**	72	66	80	68
**FGFR3(h)**	97	94	108	99
**FGFR4(h)**	99	98	93	96
**Fgr(h)**	112	90	114	95
**Flt1(h)**	101	87	86	80
**Flt3(D835Y)(h)**	91	49	87	80
**Flt3(h)**	71	70	73	62
**Flt4(h)**	92	66	81	90
**Fms(h)**	94	90	72	64
**Fms(Y969C)(h)**	81	87	80	78
**Fyn(h)**	63	66	61	75
**GCK(h)**	109	60	88	87
**GCN2(h)**	85	83	85	97
**GRK1(h)**	101	88	79	81
**GRK2(h)**	107	95	101	107
**GRK3(h)**	95	93	92	97
**GRK5(h)**	86	89	95	96
**GRK6(h)**	100	110	104	99
**GRK7(h)**	87	91	95	93
**GSK3α(h)**	93	69	101	102
**GSK3β(h)**	85	76	91	97
**Haspin(h)**	71	36	72	78
**Hck(h)**	72	64	74	62
**Hck(h) activated**	85	46	85	108
**HIPK1(h)**	94	78	120	103
**HIPK2(h)**	109	86	96	94
**HIPK3(h)**	93	84	98	95
**HIPK4(h)**	103	42	92	93
**HPK1(h)**	92	92	98	96
**HRI(h)**	96	99	99	101
**ICK(h)**	97	92	90	88
**IGF-1R(h)**	90	101	83	83
**IGF-1R(h), activated**	78	73	72	76
**IKKα(h)**	72	86	100	91
**IKKβ(h)**	106	107	110	98
**IKKε(h)**	64	67	72	79
**IR(h)**	73	98	88	70
**IR(h), activated**	98	71	64	76

**IRE1(h)**	99	83	89	88
**IRR(h)**	93	99	87	79
**IRAK1(h)**	114	87	87	106
**IRAK4(h)**	96	60	92	103
**Itk(h)**	117	88	97	103
**JAK1(h)**	117	90	93	99
**JAK2(h)**	91	108	93	86
**JAK3(h)**	94	91	94	92
**JNK1α1(h)**	98	94	83	98
**JNK2α2(h)**	89	82	78	88
**JNK3(h)**	90	90	98	107
**KDR(h)**	86	64	77	74
**LATS1(h)**	102	79	79	83
**LATS2(h)**	94	72	74	75
**Lck(h)**	90	78	94	82
**Lck(h) activated**	93	73	100	91
**LIMK1(h)**	97	64	76	72
**LIMK2(h)**	94	103	87	84
**LKB1(h)**	86	100	81	73
**LOK(h)**	91	35	81	87
**Lyn(h)**	103	80	89	91
**Lyn(m)**	81	93	91	77
**LRRK2(h)**	91	44	91	101
**LTK(h)**	88	76	76	74
**MAK(h)**	89	74	77	76
**MAPK1(h)**	96	84	93	114
**MAPK2(h)**	96	100	87	83
**MAPK2(m)**	104	118	105	91
**MAP4K3(h)**	99	87	87	93
**MAP4K4(h)**	75	73	75	92
**MAP4K5(h)**	92	80	89	97
**MAPKAP-K2(h)**	79	80	85	97
**MAPKAP-K3(h)**	95	78	66	83
**MEK1(h)**	91	93	84	73
**MEK2(h)**	92	95	96	99
**MARK1(h)**	89	89	96	82
**MARK3(h)**	95	89	87	85
**MARK4(h)**	107	100	104	105
**MEKK2(h)**	113	108	99	102
**MEKK3(h)**	93	83	107	98
**MELK(h)**	86	95	90	73
**Mer(h)**	83	68	83	89
**Met(h)**	82	72	57	112
**Met(D1246H)(h)**	116	119	111	82
**Met(D1246N)(h)**	130	118	114	103
**Met(M1268T)(h)**	91	109	101	101
**Met(Y1248C)(h)**	121	122	107	103
**Met(Y1248D)(h)**	109	103	96	104
**Met(Y1248H)(h)**	107	97	86	81
**MINK(h)**	86	67	88	89
**MKK3(h)**	101	105	82	86

**MKK4(m)**	105	110	112	118
**MKK6(h)**	78	74	84	95
**MLCK(h)**	83	69	88	86
**MLK1(h)**	89	68	77	76
**MLK2(h)**	95	96	97	93
**MLK3(h)**	99	97	99	114
**MLK4(h)**	105	91	93	94
**Mnk2(h)**	93	19	83	80
**MOK(h)**	89	65	86	100
**MRCKα(h)**	91	87	93	98
**MRCKβ(h)**	80	79	84	103
**MRCKγ(h)**	105	92	98	102
**MSK1(h)**	90	74	70	79
**MSK2(h)**	91	88	96	97
**MSSK1(h)**	85	81	91	95
**MST1(h)**	91	70	78	97
**MST2(h)**	98	102	112	81
**MST3(h)**	80	86	89	74
**MST4(h)**	58	64	81	72
**mTOR(h)**	79	77	76	80
**mTOR/FKBP12(h)**	100	88	89	94
**MuSK(h)**	74	70	81	97
**MYLK2(h)**	97	89	80	93
**MYO3B(h)**	89	88	84	87
**NDR1(h)**	108	98	96	89
**NDR2(h)**	120	103	99	115
**NEK1(h)**	81	60	97	70
**NEK2(h)**	91	92	88	95
**NEK4(h)**	71	30	27	12
**NEK3(h)**	71	87	80	99
**NEK6(h)**	103	91	94	79
**NEK7(h)**	95	76	74	83
**NEK9(h)**	98	92	103	92
**NIM1(h)**	108	97	113	110
**NEK11(h)**	41	53	71	45
**NLK(h)**	85	80	93	82
**NUAK2(h)**	121	110	112	117
**OSR1(h)**	100	107	95	108
**p70S6K(h)**	91	76	81	83
**PAK1(h)**	94	97	81	90
**PAK2(h)**	87	98	94	78
**PAK4(h)**	90	83	92	90
**PAK3(h)**	96	99	107	93
**PAK5(h)**	95	83	85	86
**PAK6(h)**	92	86	84	92
**PAR-1Bα(h)**	84	83	86	101
**PASK(h)**	66	65	44	30
**PEK(h)**	105	108	112	110
**PDGFRα(h)**	111	101	90	95
**PDGFRα(D842V)(h)**	114	50	92	86

**PDGFRα(V561D)(h)**	86	98	92	90
**PDGFRβ(h)**	97	113	104	109
**PDHK2(h)**	103	94	93	87
**PDHK4(h)**	106	104	102	101
**PDK1(h)**	102	90	88	84
**PhKγ1(h)**	102	121	120	141
**PhKγ2(h)**	78	109	91	82
**Pim-1(h)**	66	39	73	70
**Pim-2(h)**	80	76	73	70
**Pim-3(h)**	123	96	92	97
**PKA(h)**	103	97	101	106
**PKAcβ(h)**	87	85	97	107
**PKBα(h)**	86	88	98	89
**PKBβ(h)**	106	106	84	75
**PKBγ(h)**	101	89	102	105
**PKCα(h)**	89	86	94	79
**PKCβI(h)**	95	86	100	93
**PKCβII(h)**	107	110	100	104
**PKCγ(h)**	86	81	74	72
**PKCδ(h)**	81	75	97	88
**PKCε(h)**	95	93	93	86
**PKCη(h)**	80	82	103	93
**PKCι(h)**	84	89	114	98
**PKCμ(h)**	97	93	85	80
**PKCθ(h)**	80	117	100	90
**PKCζ(h)**	85	91	75	71
**PKD2(h)**	106	77	74	76
**PKD3(h)**	100	91	114	102
**PKG1α(h)**	91	95	96	93
**PKG1β(h)**	91	94	84	76
**PKR(h)**	100	71	98	94
**Plk1(h)**	93	96	96	92
**Plk3(h)**	88	92	94	99
**Plk4(h)**	91	87	80	78
**PRAK(h)**	107	102	108	91
**PRKG2(h)**	103	113	96	79
**PRK1(h)**	89	77	72	80
**PRK2(h)**	108	95	104	96
**PrKX(h)**	97	93	97	112
**PRP4(h)**	104	100	105	99
**PTK5(h)**	85	64	84	95
**Pyk2(h)**	86	72	74	69
**Ret(h)**	69	43	99	62
**Ret (V804L)(h)**	82	91	78	71
**Ret(V804M)(h)**	99	102	85	73
**RIPK1(h)**	86	78	73	80
**RIPK2(h)**	82	22	86	86
**ROCK-I(h)**	91	96	98	93
**ROCK-II(h)**	88	84	81	79
**ROCK-II(r)**	90	98	105	103

**Ron(h)**	98	88	109	101
**Ros(h)**	95	101	101	105
**Rse(h)**	104	107	100	103
**Rsk1(h)**	102	116	110	103
**Rsk1(r)**	117	129	130	113
**Rsk2(h)**	93	70	79	73
**Rsk3(h)**	126	87	108	111
**Rsk4(h)**	117	100	81	92
**SAPK2a(h)**	95	99	98	88
**SAPK2a(T106M)(h)**	95	121	115	103
**SAPK2b(h)**	79	83	95	84
**SAPK3(h)**	106	101	114	87
**SAPK4(h)**	97	105	105	92
**SBK1(h)**	106	83	95	103
**SGK(h)**	82	82	86	92
**SGK2(h)**	108	103	115	89
**SGK3(h)**	95	91	88	93
**SIK(h)**	98	74	90	86
**SIK2(h)**	91	65	97	84
**SIK3(h)**	95	81	79	71
**SLK(h)**	98	70	77	78
**Snk(h)**	98	80	89	100
**SNRK(h)**	103	79	104	103
**Src(1-530)(h)**	107	90	101	81
**Src(T341M)(h)**	94	96	103	83
**SRMS(h)**	86	78	84	71
**SRPK1(h)**	121	116	112	108
**SRPK2(h)**	80	80	87	104
**STK16(h)**	88	76	75	78
**STK25(h)**	114	114	112	111
**STK32A(h)**	95	103	99	100
**STK32B(h)**	95	93	92	97
**STK32C(h)**	106	102	95	106
**STK33(h)**	82	77	90	100
**STK39(h)**	75	114	100	91
**Syk(h)**	113	110	84	92
**TAF1L(h)**	80	32	66	70
**TAK1(h)**	74	74	83	82
**TAO1(h)**	99	95	104	97
**TAO2(h)**	110	112	109	107
**TAO3(h)**	94	77	78	82
**TBK1(h)**	126	117	108	108
**Tec(h) activated**	78	76	100	86
**TGFBR1(h)**	87	94	97	86
**TGFBR2(h)**	97	96	91	93
**Tie2 (h)**	104	94	92	75
**Tie2(R849W)(h)**	91	110	98	90
**Tie2(Y897S)(h)**	88	78	94	85
**TLK1(h)**	112	107	112	97
**TLK2(h)**	99	97	98	103
**TNIK(h)**	72	65	83	63

**TRB2(h)**	69	31	65	60
**TrkA(h)**	65	70	87	60
**TrkB(h)**	87	96	81	81
**TrkC(h)**	111	114	104	97
**TSSK1(h)**	82	81	82	77
**TSSK2(h)**	101	103	97	103
**TSSK3(h)**	111	104	97	95
**TSSK4(h)**	89	134	112	125
**TTBK1(h)**	119	112	104	114
**TTBK2(h)**	117	114	107	111
**TTK(h)**	64	104	84	89
**Txk(h)**	92	85	83	82
**TYK2(h)**	109	75	86	84
**ULK1(h)**	99	93	91	84
**ULK2(h)**	101	97	93	94
**ULK3(h)**	96	90	92	93
**VRK1(h)**	94	89	82	79
**VRK2(h)**	91	88	99	94
**Wee1(h)**	109	102	114	89
**Wee1B(h)**	95	106	99	100
**WNK1(h)**	78	75	81	92
**WNK2(h)**	77	71	74	76
**WNK3(h)**	90	104	98	86
**WNK4(h)**	105	100	94	92
**Yes(h)**	92	72	86	84
**ZAK(h)**	70	61	61	72
**ZAP-70(h)**	146	113	146	130
**ZIPK(h)**	97	108	103	109
**ATM(h)**	93	90	88	86
**DNA-PK(h)**	89	72	74	85
**PI3 Kinase (p110b/p85a)(h)**	102	103	99	93
**PI3 Kinase (p120g)(h)**	91	91	92	90
**PI3 Kinase (p110d/p85a)(h)**	93	96	84	80
**PI3 Kinase (p110a/p85a)(m)**	86	84	83	82
**PI3 Kinase (p110a/p65a)(m)**	98	101	86	87
**PI3 Kinase (p110a(E545K)/p85a)(m)**	94	91	91	91
**PI3 Kinase (p110a(H1047R)/p85a)(m)**	96	93	95	94
**PI3 Kinase (p110b/p85b)(m)**	97	89	86	87
**PI3 Kinase (p110b/p85a)(m)**	102	99	98	101
**PI3 Kinase (p110d/p85a)(m)**	97	96	93	93
**PI3 Kinase (p110a(E542K)/p85a)(m)**	96	93	91	94

**PI3 Kinase (p110a/p85a)(h)**	100	101	103	94
**PI3 Kinase (p110a(E542K)/p85a)(h)**	94	88	91	93
**PI3 Kinase (p110a(H1047R)/p85a)(h)**	99	96	97	94
**PI3 Kinase (p110a(E545K)/p85a)(h)**	96	81	93	91
**PI3 Kinase (p110a/p65a)(h)**	98	91	95	94
**PI3KC2a(h)**	102	106	100	105
**PI3KC2g(h)**	70	40	57	34
**PIP4K2a(h)**	93	102	96	92
**PIP5K1a(h)**	104	101	103	104
**PIP5K1g(h)**	100	107	104	101

Determination IC_{50} values for NSCLC cells

To get more insight in the activity of the different compounds, the IC_{50} values were determined. PC9 cells were treated with the indicated compounds at concentrations ranging between 0 and 5 μM. Cell viability was measured using CellTiter Glo upon 72 hours of treatment. Data was plotted and IC_{50}s were calculated based on a sigmoidal, 4PL standard curve interpolation using Graphpad Prism.

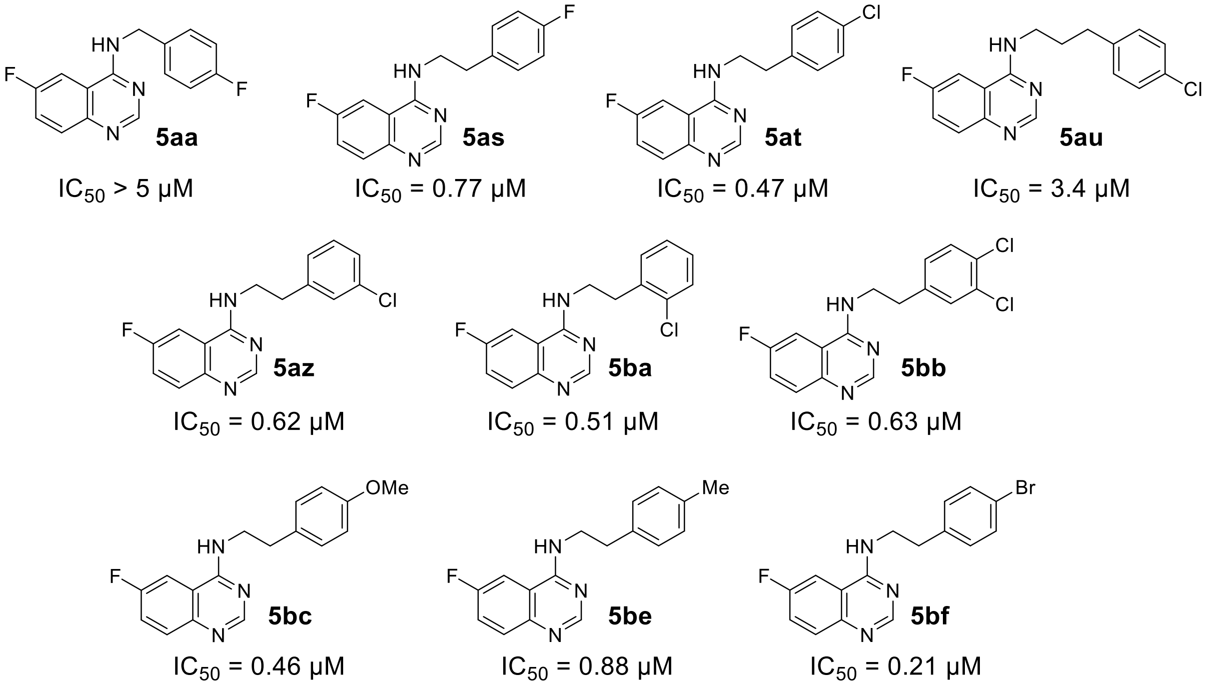

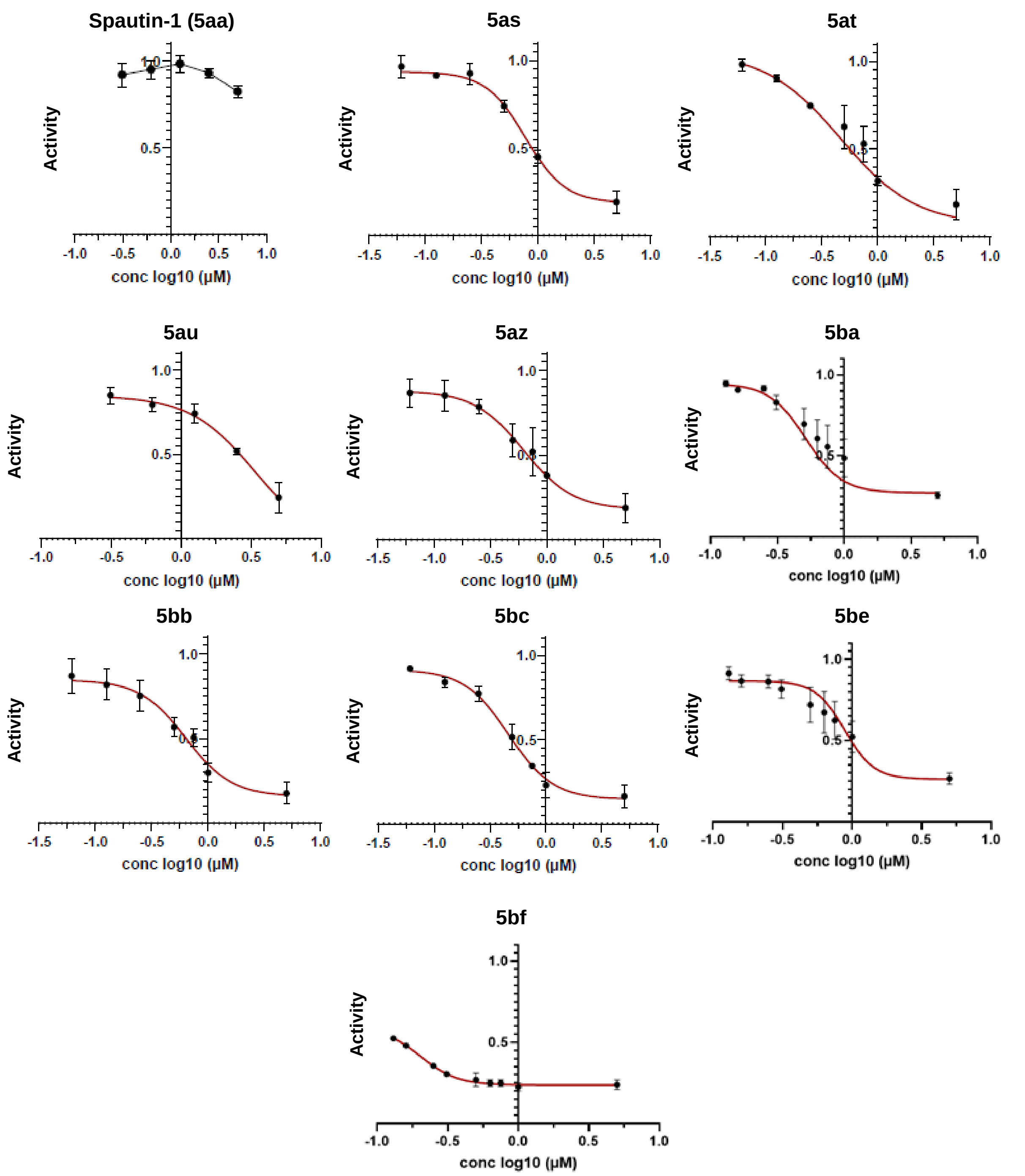

Figure S1. *The IC₅₀ values for the most promising compounds based on the EGFR-mutant NSCLC cell viability essay were determined.*

ITC data

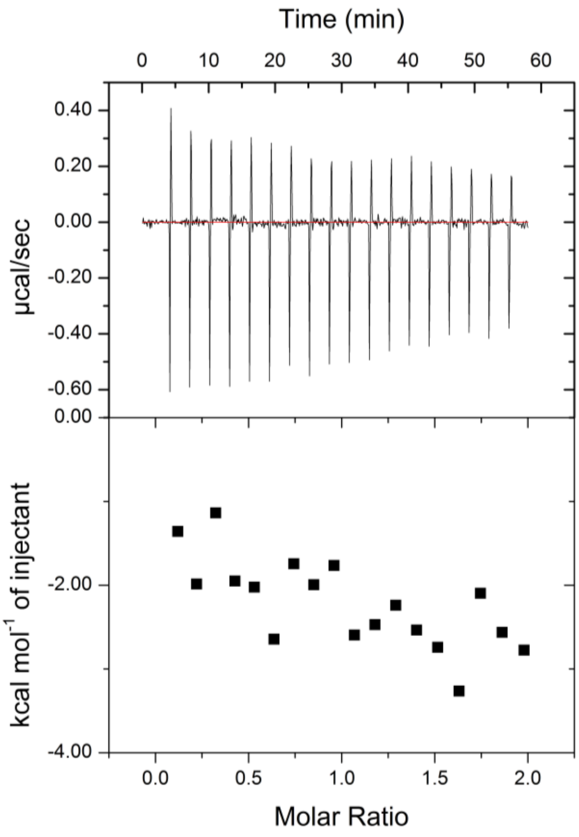

Figure S2. *Result of the Isothermal Titration Calorimetry (ITC) experiment for USP13 with Spautin-1.*

TSA data

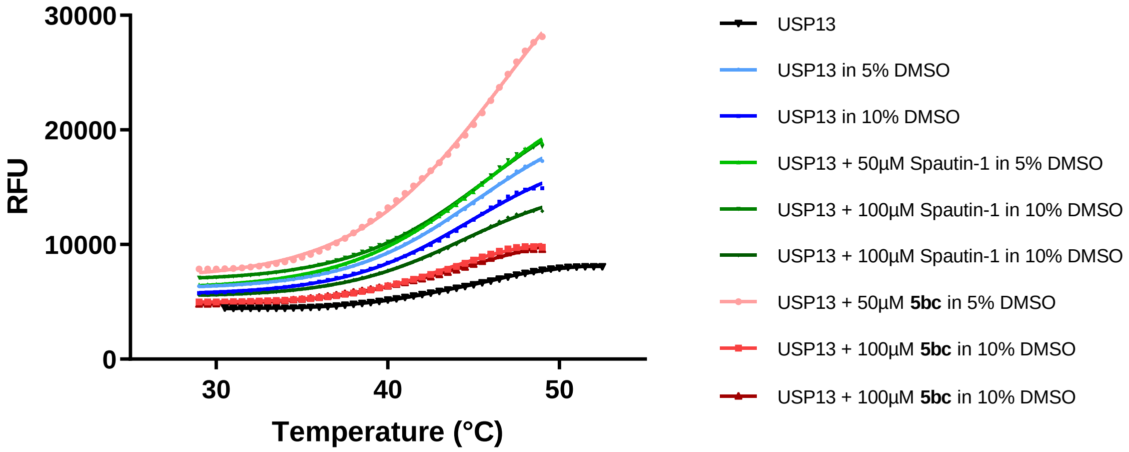

Figure S3. *Thermal shift assay (TSA) showing the thermal unfolding of the protein in combination with the different compounds. USP13 protein was at a concentration of 0.4 mg/ml and the temperatures were increased from 10°C to 80°C with 0.5°C per 30 seconds. Relative Fluorescence Unit (RFU) was plotted and the melting temperatures were determined by fitting a Boltzmann sigmoidal equation to the data using GraphPad Prism.*

Expression of NEK4 in lung cancer cell lines

The RNA-seq data were obtained from the Cancer Cell Line Encyclopedia (CCLE) database, RMA normalised and represented as the log2. These data suggests that PC9 expresses 19.3% more mRNA than the A549 NSCLC cell line.

Table S2. NEK4 expression in lung cancer cell lines

Gene	NEK4 (RMA normalized, log2)	Gene	NEK4 (RMA normalized, log2)
A549_LUNG	2.827601	NCIH810_LUNG	2.578587
PC9_LUNG	3.082981	RERFLCMS_LUNG	2.747514
DMS53_LUNG	2.307848	NCIH460_LUNG	3.018252
NCIH1694_LUNG	2.386839	LU99_LUNG	3.6338
NCIH1184_LUNG	3.611097	CAL12T_LUNG	2.959077
NCIH2227_LUNG	3.483929	LUDLU1_LUNG	2.687402
RERFLCAD2_LUNG	2.757626	CORL23_LUNG	2.728065
NCIH2347_LUNG	3.936043	MORCPR_LUNG	2.59087
NCIH2087_LUNG	3.069918	HARA_LUNG	3.125519
NCIH2085_LUNG	3.012699	NCIH1568_LUNG	3.442216
NCIH510_LUNG	3.847098	NCIH1563_LUNG	2.94666
NCIH2066_LUNG	3.05717	NCIH358_LUNG	3.652477
NCIH1341_LUNG	3.186498	NCIH1650_LUNG	3.484508
NCIH2029_LUNG	3.776141	NCIH1793_LUNG	3.616278
LU65_LUNG	3.298798	NCIH1437_LUNG	1.784392
NCIH1573_LUNG	2.708165	KNS62_LUNG	2.612259
NCIH1651_LUNG	2.992846	SW1271_LUNG	2.466821
NCIH226_LUNG	1.979705	NCIH1792_LUNG	3.498902
NCIH1666_LUNG	2.110469	NCIH82_LUNG	3.030941
NCIH1734_LUNG	3.078244	IALM_LUNG	2.780714
NCIH2342_LUNG	3.058066	EPLC272H_LUNG	2.840902
EBC1_LUNG	3.243013	NCIH1355_LUNG	2.971251
NCIH322_LUNG	3.092271	HCC95_LUNG	2.765512
HS618T_LUNG	2.621223	HCC1438_LUNG	3.548591
NCIH2291_LUNG	3.685822	HCC2108_LUNG	3.281732
HCC33_LUNG	3.269083	HCC1359_LUNG	3.10483
CORL105_LUNG	2.251828	NCIH2110_LUNG	2.920672
COLO668_LUNG	3.392451	CHAGOK1_LUNG	2.458839
NCIH1915_LUNG	3.707645	DMS454_LUNG	2.390835
HCC2279_LUNG	2.00153	CORL47_LUNG	3.056463
HCC1588_LUNG	3.105903	DMS273_LUNG	3.144994
NCIH838_LUNG	3.027055	CORL311_LUNG	2.686798
HCC1171_LUNG	3.151546	NCIH2286_LUNG	2.325288
NCIH2228_LUNG	2.159578	NCIH1339_LUNG	3.578247
RERFLCAI_LUNG	3.345584	NCIH2081_LUNG	3.578637
SKMES1_LUNG	2.743384	NCIH1092_LUNG	3.511286
NCIH520_LUNG	2.995088	NCIH1105_LUNG	4.146481

NCIH1963_LUNG	2.994291	NCIH1436_LUNG	2.784224
HCC15_LUNG	2.321403	NCIH1876_LUNG	3.228404
CALU6_LUNG	3.229614	CORL24_LUNG	2.686496
SKLU1_LUNG	2.848037	CORL88_LUNG	3.923138
SW900_LUNG	2.877169	CORL95_LUNG	3.125332
PC14_LUNG	2.915832	NCIH522_LUNG	3.229351
NCIH596_LUNG	2.249992	NCIH2172_LUNG	2.549502
NCIH650_LUNG	3.64937	NCIH661_LUNG	3.240212
SW1573_LUNG	3.680344	HCC78_LUNG	2.509668
HCC366_LUNG	3.544885	RERFLCAD1_LUNG	2.247014
NCIH292_LUNG	2.959466	NCIH1155_LUNG	4.306699
HCC1833_LUNG	3.536775	ABC1_LUNG	2.804218
CORL279_LUNG	2.734352	NCIH1703_LUNG	2.638764
NCIH3255_LUNG	3.133862	NCIH1581_LUNG	3.589011
HCC1195_LUNG	3.095506	NCIH1395_LUNG	2.799249
NCIH854_LUNG	2.063449	NCIH1781_LUNG	3.296869
LC1F_LUNG	2.847374	NCIH146_LUNG	3.16201
T3M10_LUNG	1.710536	DMS79_LUNG	3.746567
NCIH2023_LUNG	2.90636	NCIH2106_LUNG	3.210801
DMS153_LUNG	3.239519	NCIH526_LUNG	3.46031
NCIH1048_LUNG	3.985948	NCIH2196_LUNG	3.464676
NCIH1618_LUNG	3.818546	NCIH446_LUNG	2.707119
NCIH69_LUNG	3.659112	CALU1_LUNG	2.607501
DV90_LUNG	3.225003	NCIH2170_LUNG	3.359562
NCIH1648_LUNG	1.89814	SQ1_LUNG	3.287637
NCIH23_LUNG	2.973317	NCIH2444_LUNG	2.579775
NCIH1373_LUNG	2.462822	NCIH1299_LUNG	3.237432
NCIH1930_LUNG	3.38046	SALE_LUNG	2.862842
NCIH1623_LUNG	3.810277	HCC2814_LUNG	3.197453
HS229T_LUNG	2.882935	TIG3TD_LUNG	2.361958
NCIH1838_LUNG	3.442035	HCC364_LUNG	3.374548
NCIH1836_LUNG	3.833778	HCC827GR5_LUNG	2.997448
NCIH1755_LUNG	3.149542	NCIH2126_LUNG	2.564153
RERFLCKJ_LUNG	3.467386	NCIH1385_LUNG	1.316245
NCIH1693_LUNG	3.26566	HCC827_LUNG	2.599864
DMS114_LUNG	3.763492	LXF289_LUNG	2.855473
NCIH524_LUNG	4.107009	SCLC21H_LUNG	2.324181
NCIH209_LUNG	3.197037	CALU3_LUNG	2.956813
SBC5_LUNG	2.89073	NCIH1435_LUNG	3.441531
NCIH196_LUNG	3.248309	NCIH2009_LUNG	3.660598
NCIH889_LUNG	3.332097	NCIH1869_LUNG	3.517293
NCIH211_LUNG	3.343948	HCC4006_LUNG	2.422417
NCIH2171_LUNG	3.126809	SHP77_LUNG	3.836029
LOUNH91_LUNG	3.186683	NCIH841_LUNG	3.252339
LK2_LUNG	3.217948	NCIH1944_LUNG	3.47576
NCIH441_LUNG	3.180062	NCIH2405_LUNG	2.562631
RERFLCSQ1_LUNG	2.503171	BEN_LUNG	1.695045
NCIH2030_LUNG	3.098277	NCIH727_LUNG	3.123455

NCIH2122_LUNG	3.167637	HCC2935_LUNG	2.644161
NCIH1975_LUNG	3.393545	LCLC103H_LUNG	2.752507
NCIH647_LUNG	2.846299	LCLC97TM1_LUNG	3.507663
HLFA_LUNG	2.442031	NCIH2073_LUNG	2.796147
HCC44_LUNG	2.686453	HOP62_LUNG	3.487867
EKVX_LUNG	2.858168	HCC515_LUNG	3.233624
HOP92_LUNG	2.689385	NCIH2077_LUNG	3.118293
A427_LUNG	3.372143	NCIH2882_LUNG	2.318502
HCC2429_LUNG	4.037009	NCIH2887_LUNG	3.194312
HCC2450_LUNG	2.872654	NCIH1819_LUNG	3.629495
HCC461_LUNG	2.754879	NCIH3122_LUNG	3.432579

Determination IC_{50} values for NEK4

The IC_{50} values for NEK4 were determined by Eurofins. The procedure consists of incubation of NEK4(h) with 8 mM MOPS (3-(n-morpholino)propanesulfonic acid), 0.2 mM EDTA (ethylenediamine tetraacetic acid), 0.33 mg/ml myelin basic protein, 10 mM Magnesium acetate and [gamma-33P-ATP] at pH 7.0. The reaction was initiated by the addition of the Mg/ATP mix. After incubation (2 hours at room temperature), the reaction was stopped by adding phosphoric acid to a concentration of 0.5%. Then, 10 µL of the reaction mixture was spotted onto a P30 filtermat and was washed four times for four minutes in 0.425% phosphoric acid and once in methanol prior drying and scintillation counting.

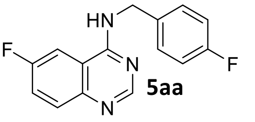

IC_{50} (NEK4)= 20.8 µM

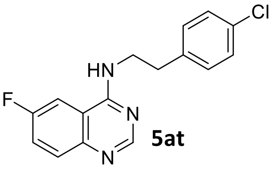

IC_{50} (NEK4)= 1.21 µM

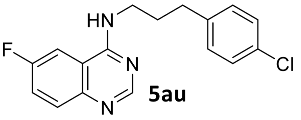

IC_{50} (NEK4)= 1.9 µM

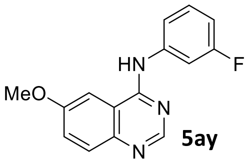

IC_{50} (NEK4)= 1.68 µM

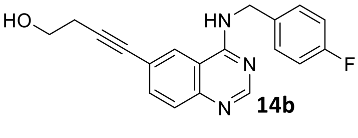

IC_{50} (NEK4)= 9.36 µM

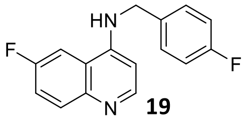

IC_{50} (NEK4)= 267 µM

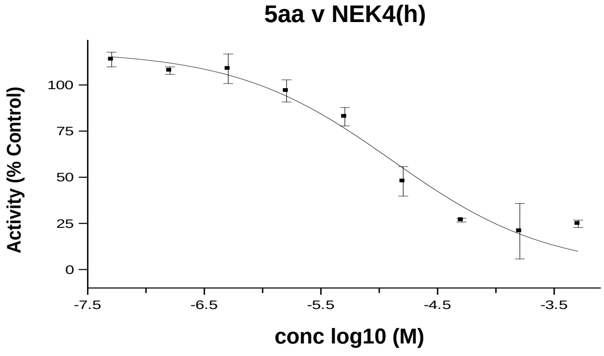

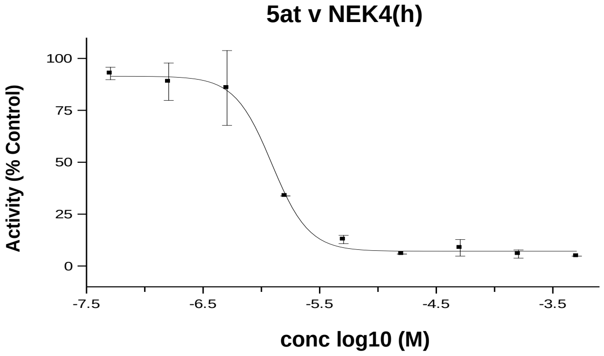

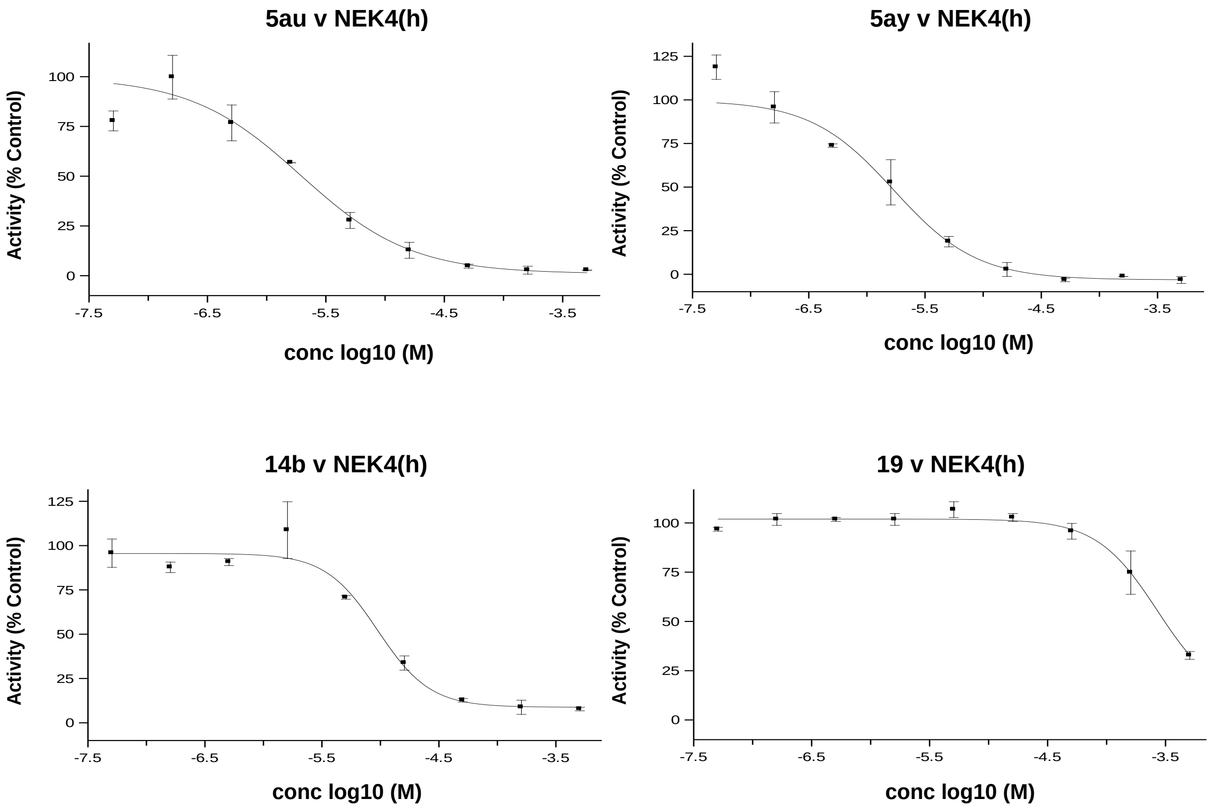

Figure S4. *The IC50 (NEK4) values were determined for Spautin-1 and 5 promising analogues.*

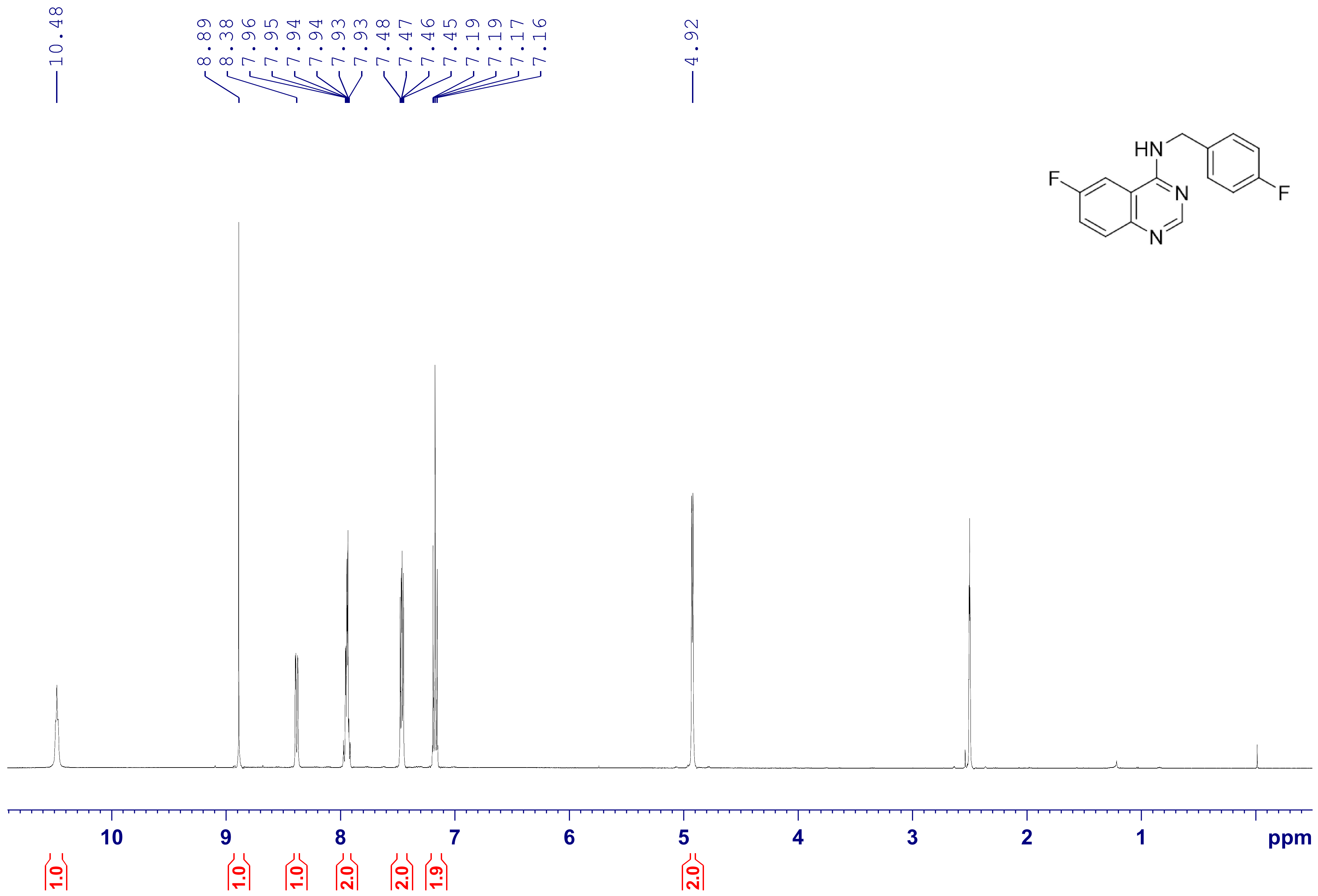

10.48
8.89
8.38
7.96
7.95
7.94
7.94
7.93
7.93
7.48
7.47
7.46
7.45
7.19
7.19
7.17
7.16
4.92
HN
F
N
N
F
10
9
8
7
6
5
4
3
2
1
ppm
1.0
1.0
1.0
2.0
2.0
1.9
2.0

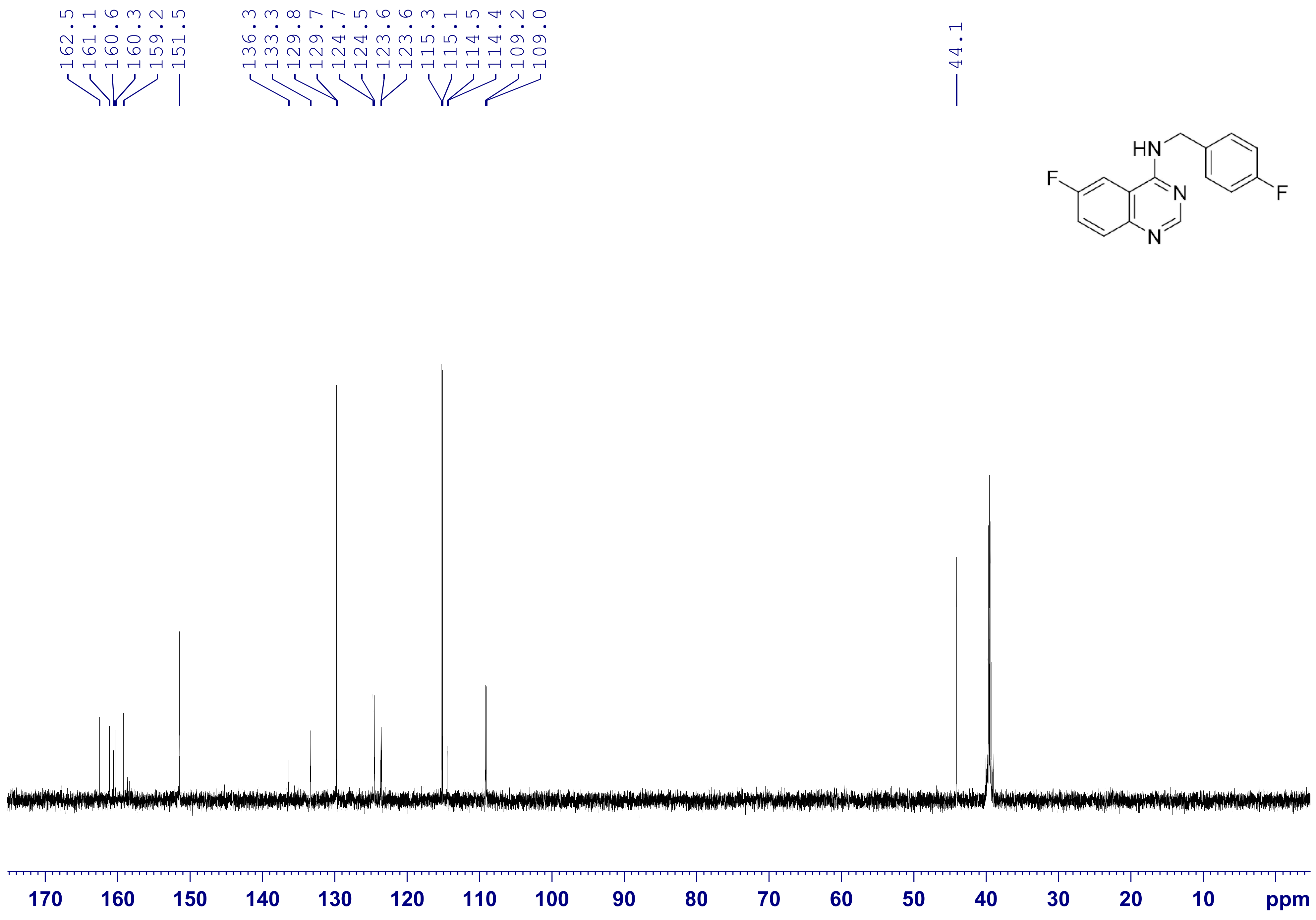
162.5
161.1
160.6
160.3
159.2
151.5
136.3
133.3
129.8
129.7
124.7
124.5
123.6
123.6
115.3
115.1
114.5
114.4
109.2
109.0
44.1
HN
F
N
N
F
170
160
150
140
130
120
110
100
90
80
70
60
50
40
30
20
10
ppm

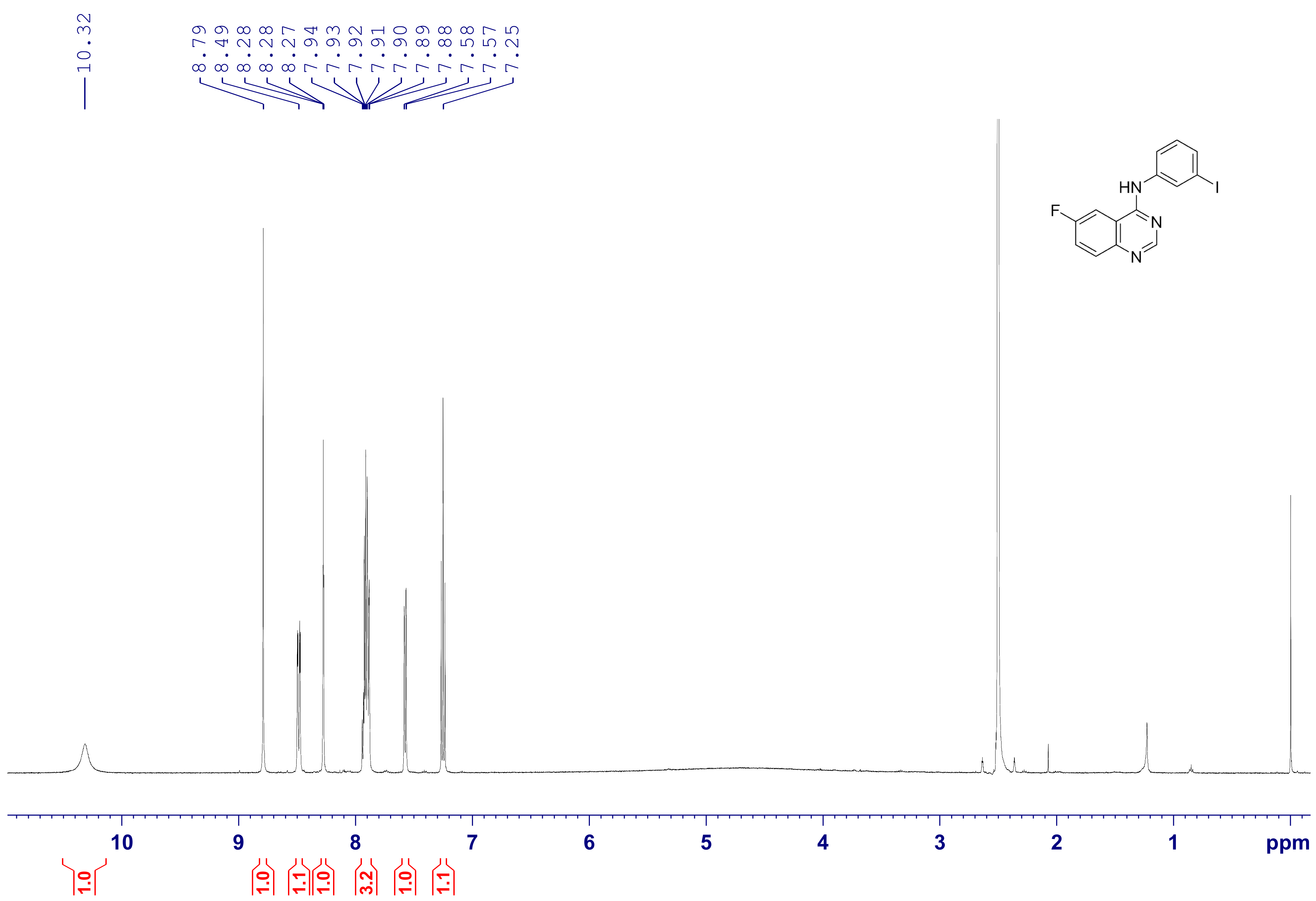
10.32
8.79
8.49
8.28
8.28
8.27
7.94
7.93
7.92
7.91
7.90
7.89
7.88
7.58
7.57
7.25
HN
F
N
N
I
10
9
8
7
6
5
4
3
2
1
ppm
1.0
1.0
1.1
1.0
3.2
1.0
1.1

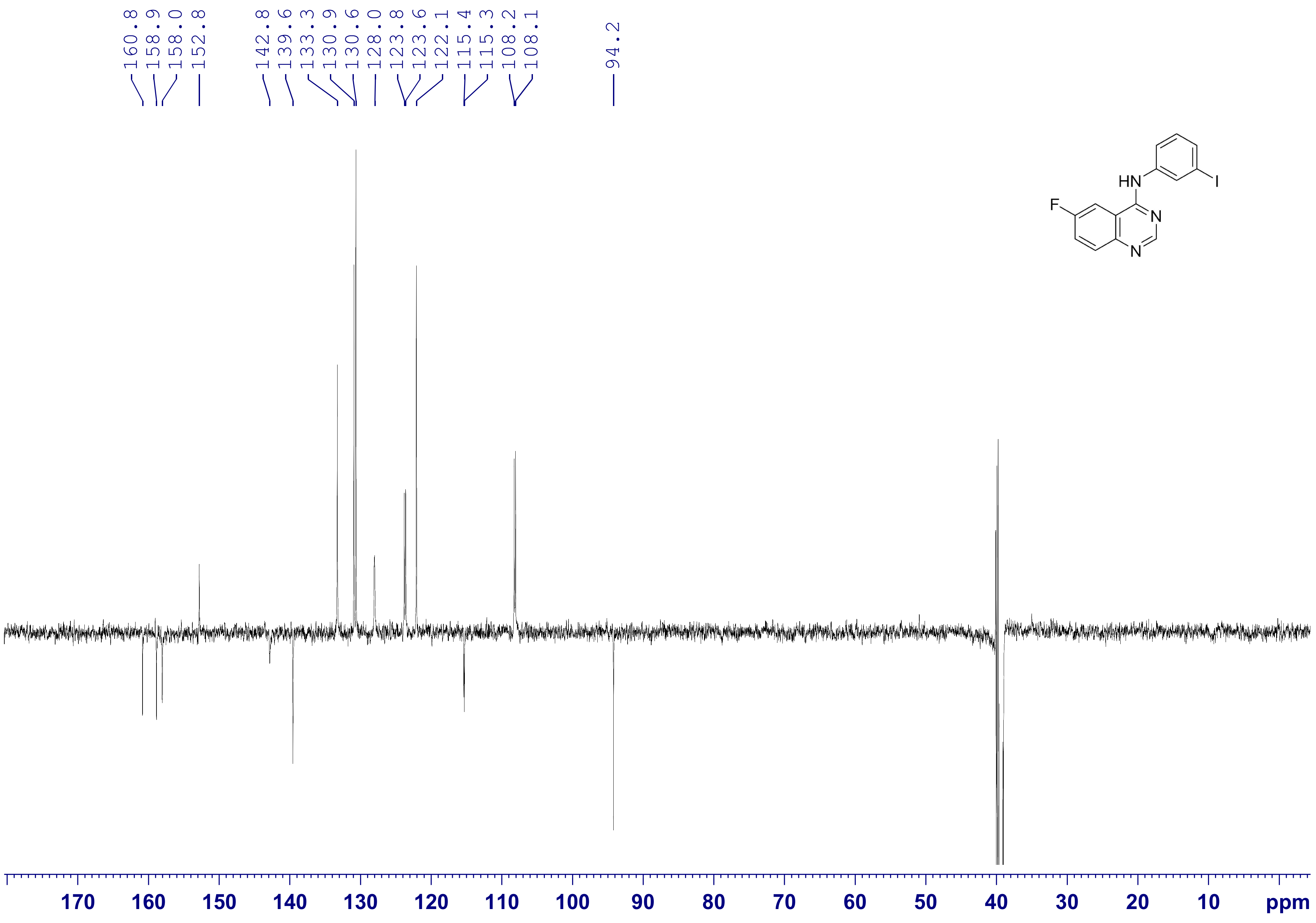
160.8
158.9
158.0
152.8
142.8
139.6
133.3
130.9
130.6
128.0
123.8
123.6
122.1
115.4
115.3
108.2
108.1
94.2
HN
F
N
N
I
170
160
150
140
130
120
110
100
90
80
70
60
50
40
30
20
10
ppm

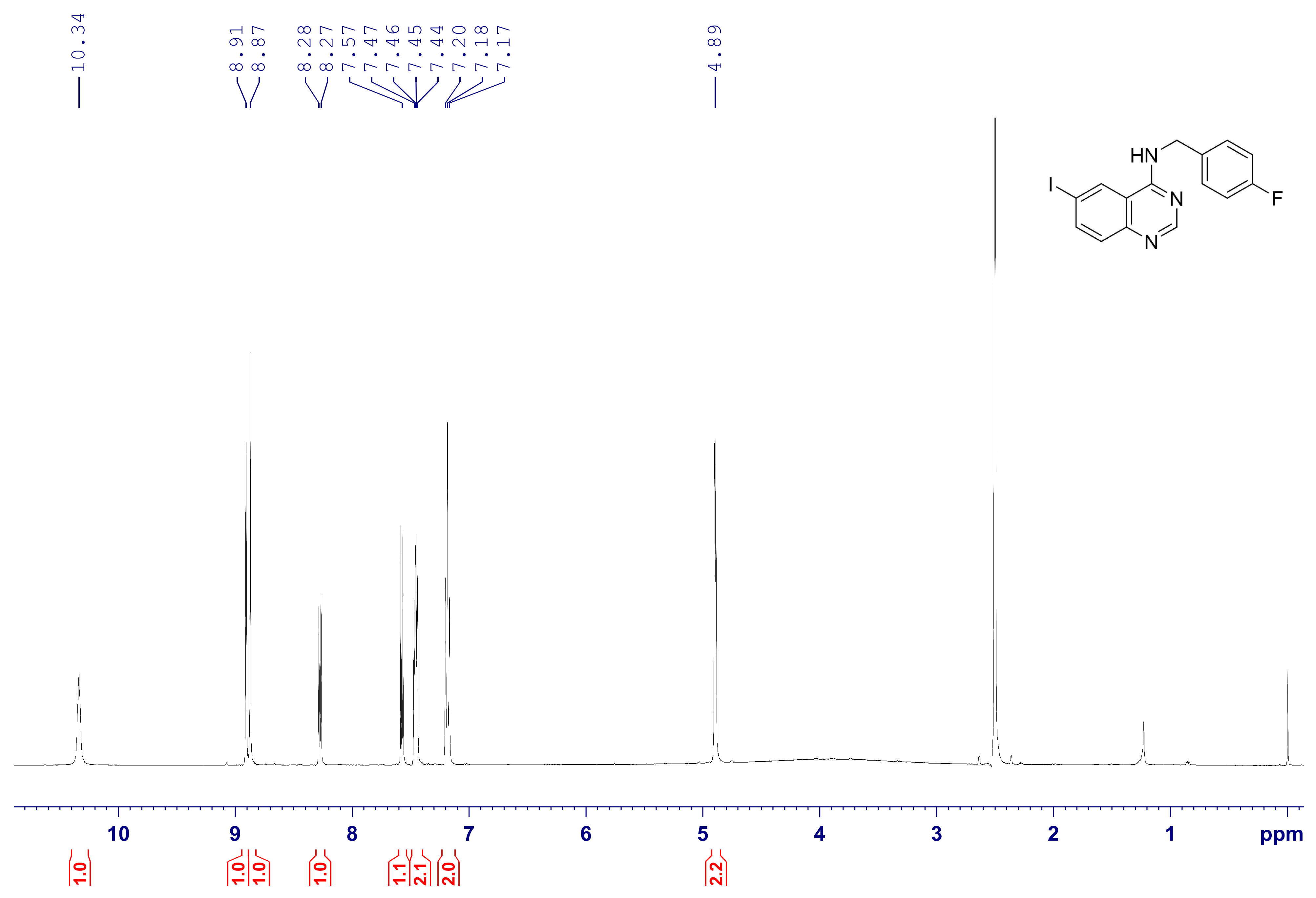

10.34
8.91
8.87
8.28
8.27
7.57
7.47
7.46
7.45
7.44
7.20
7.18
7.17
4.89
HN
I
N
N
F
10
9
8
7
6
5
4
3
2
1
ppm
1.0
1.0
1.0
1.0
1.1
2.1
2.0
2.2

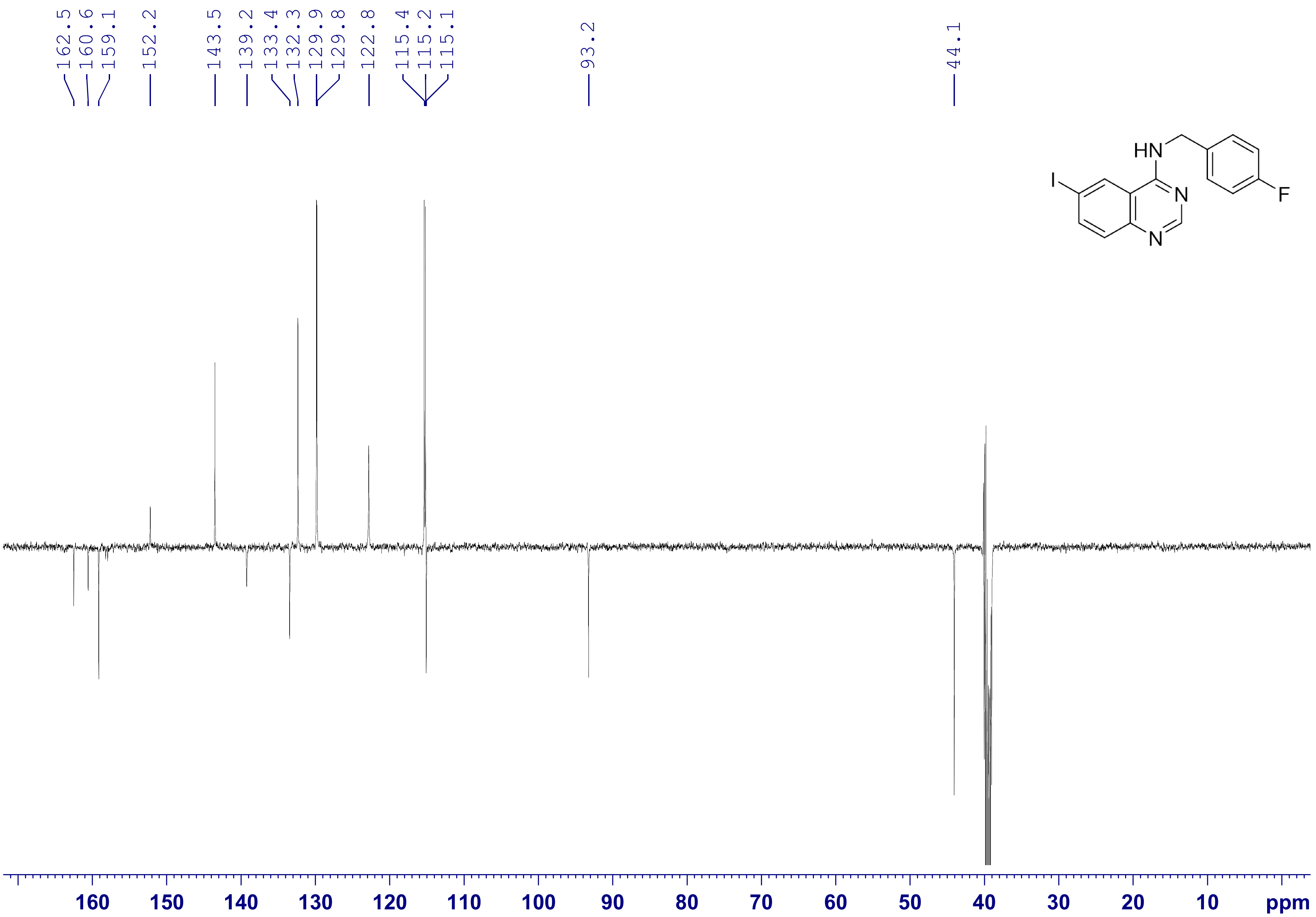
162.5
160.6
159.1
152.2
143.5
139.2
133.4
132.3
129.9
129.8
122.8
115.4
115.2
115.1
93.2
44.1
HN
I
N
N
F
160
150
140
130
120
110
100
90
80
70
60
50
40
30
20
10
ppm

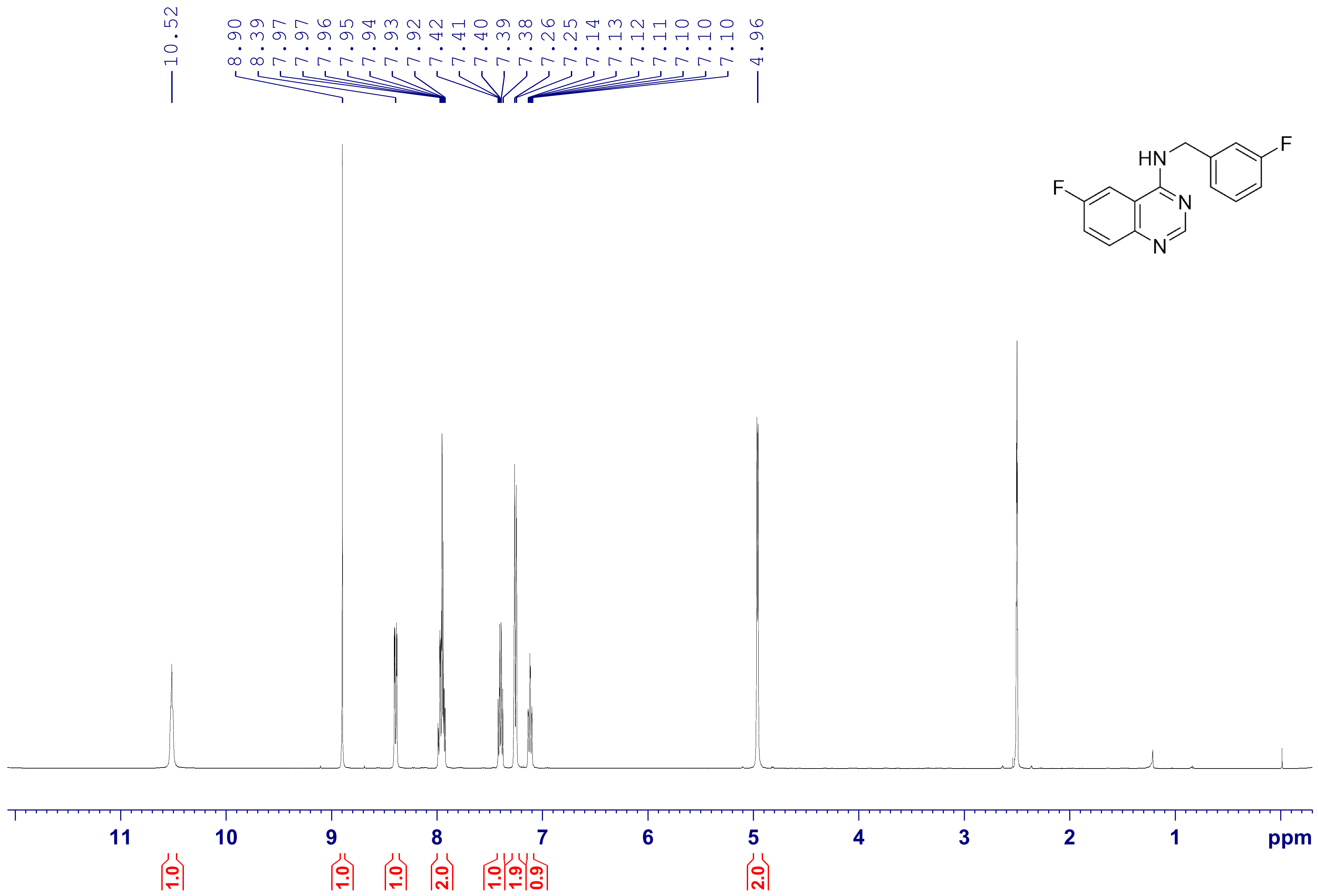
10.52
8.90
8.39
7.97
7.97
7.96
7.95
7.94
7.93
7.92
7.42
7.41
7.40
7.39
7.38
7.26
7.25
7.14
7.13
7.12
7.11
7.10
7.10
7.10
4.96
HN
F
N
N
F
11
10
9
8
7
6
5
4
3
2
1
ppm
1.0
1.0
1.0
2.0
1.0
1.9
0.9
2.0

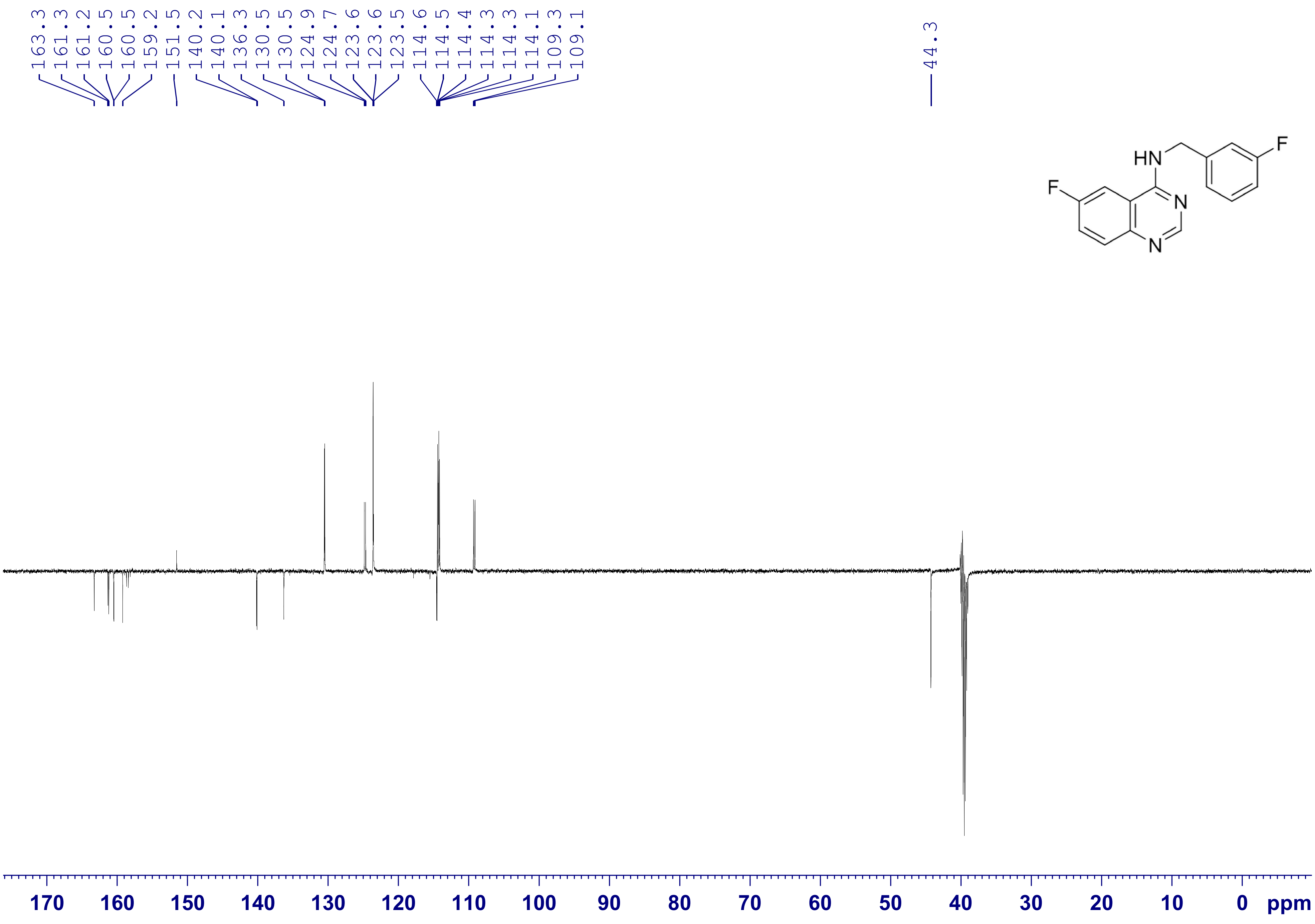
163.3
161.3
161.2
160.5
160.5
159.2
151.5
140.2
140.1
136.3
130.5
130.5
124.9
124.7
123.6
123.6
123.5
114.6
114.5
114.4
114.3
114.3
114.1
109.3
109.1
44.3
F
HN
N
N
F
170 160 150 140 130 120 110 100 90 80 70 60 50 40 30 20 10 0 ppm

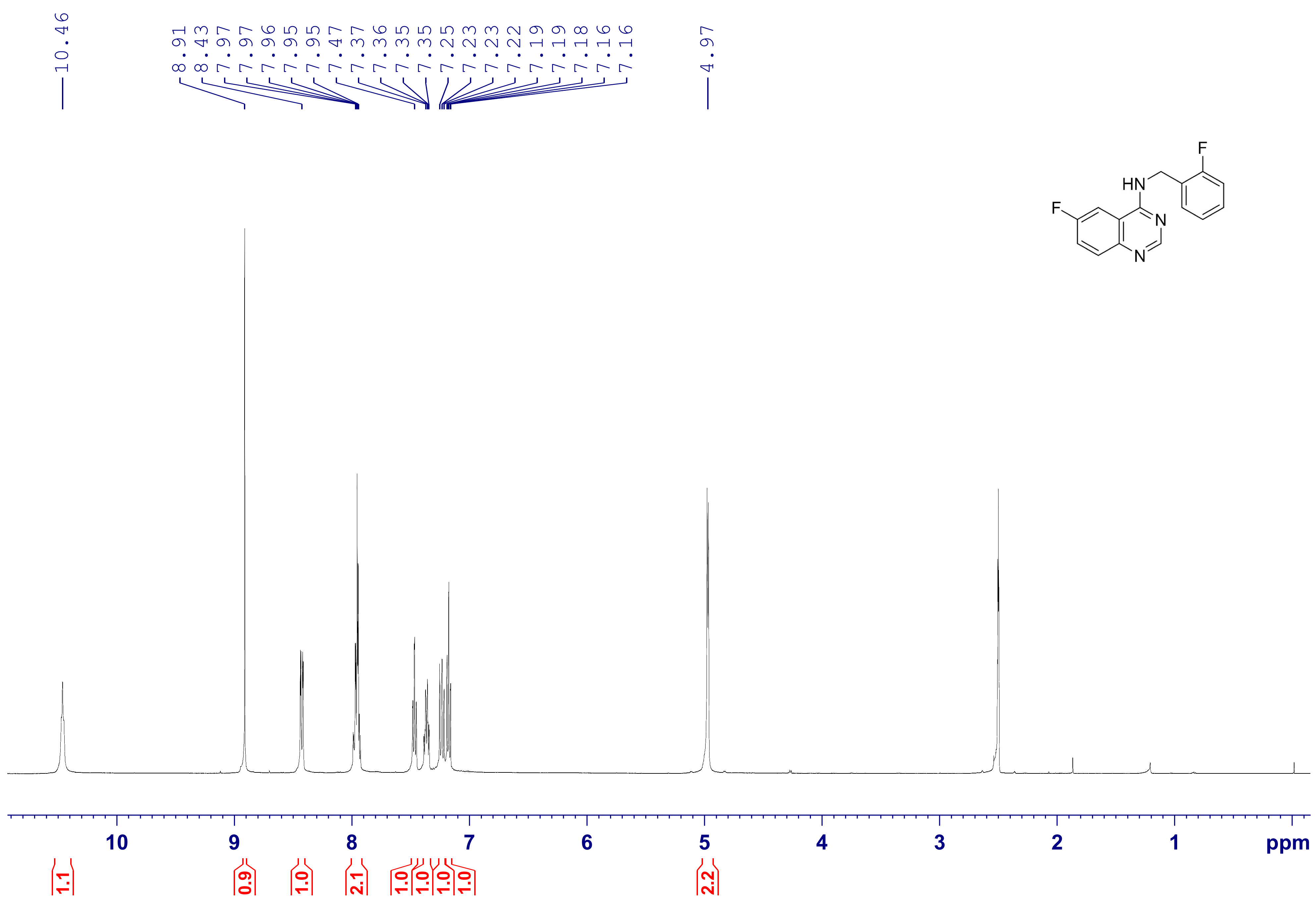
10.46
8.91
8.43
7.97
7.97
7.96
7.95
7.95
7.47
7.37
7.36
7.35
7.35
7.25
7.23
7.23
7.22
7.19
7.19
7.18
7.16
7.16
4.97
F
HN
N
N
F
10
9
8
7
6
5
4
3
2
1
ppm
1.1
0.9
1.0
2.1
1.0
1.0
1.0
1.0
2.2

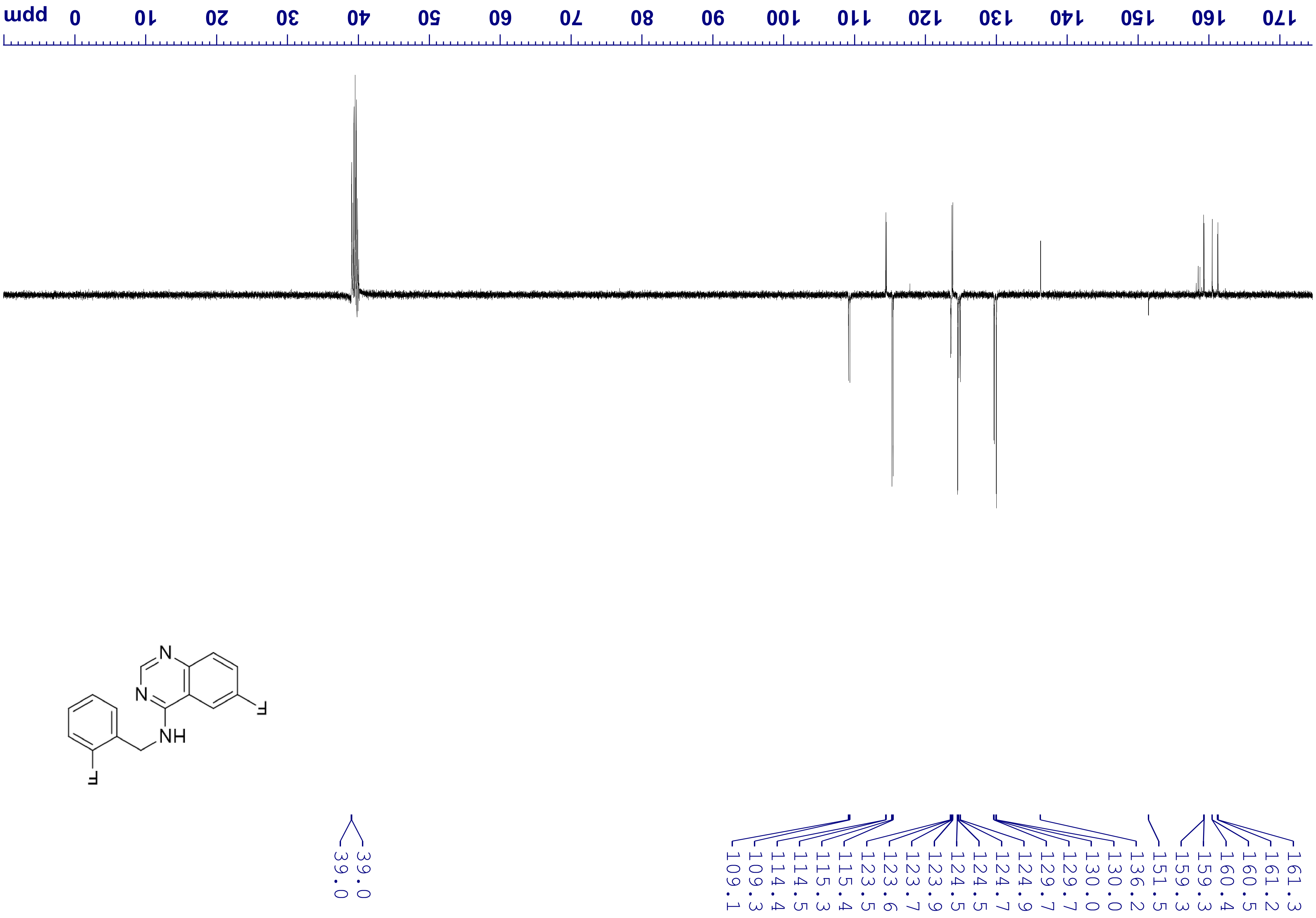
ppm
0
10
20
30
40
50
60
70
80
90
100
110
120
130
140
150
160
170
39.0
39.0
109.1
109.3
114.4
114.5
115.3
115.4
123.5
123.6
123.7
123.9
124.5
124.5
124.7
124.9
129.7
129.7
130.0
130.0
136.2
151.5
159.3
159.3
160.4
160.5
161.2
161.3
N
N
NH
F
F

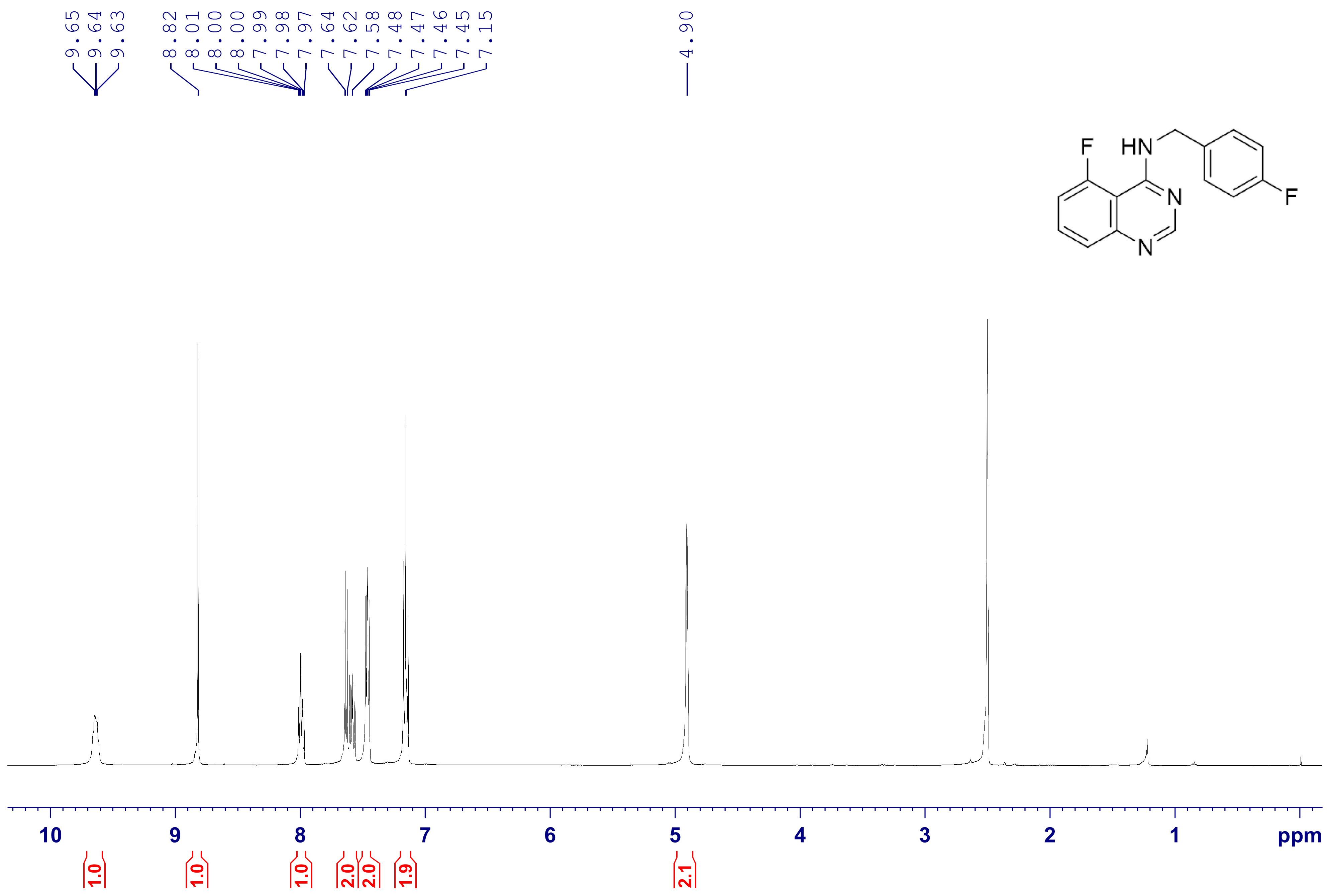
9.65
9.64
9.63
8.82
8.01
8.00
8.00
7.99
7.98
7.97
7.64
7.62
7.58
7.48
7.47
7.46
7.45
7.15
4.90
F
HN
N
N
F
10
9
8
7
6
5
4
3
2
1
ppm
1.0
1.0
1.0
2.0
2.0
1.9
2.1

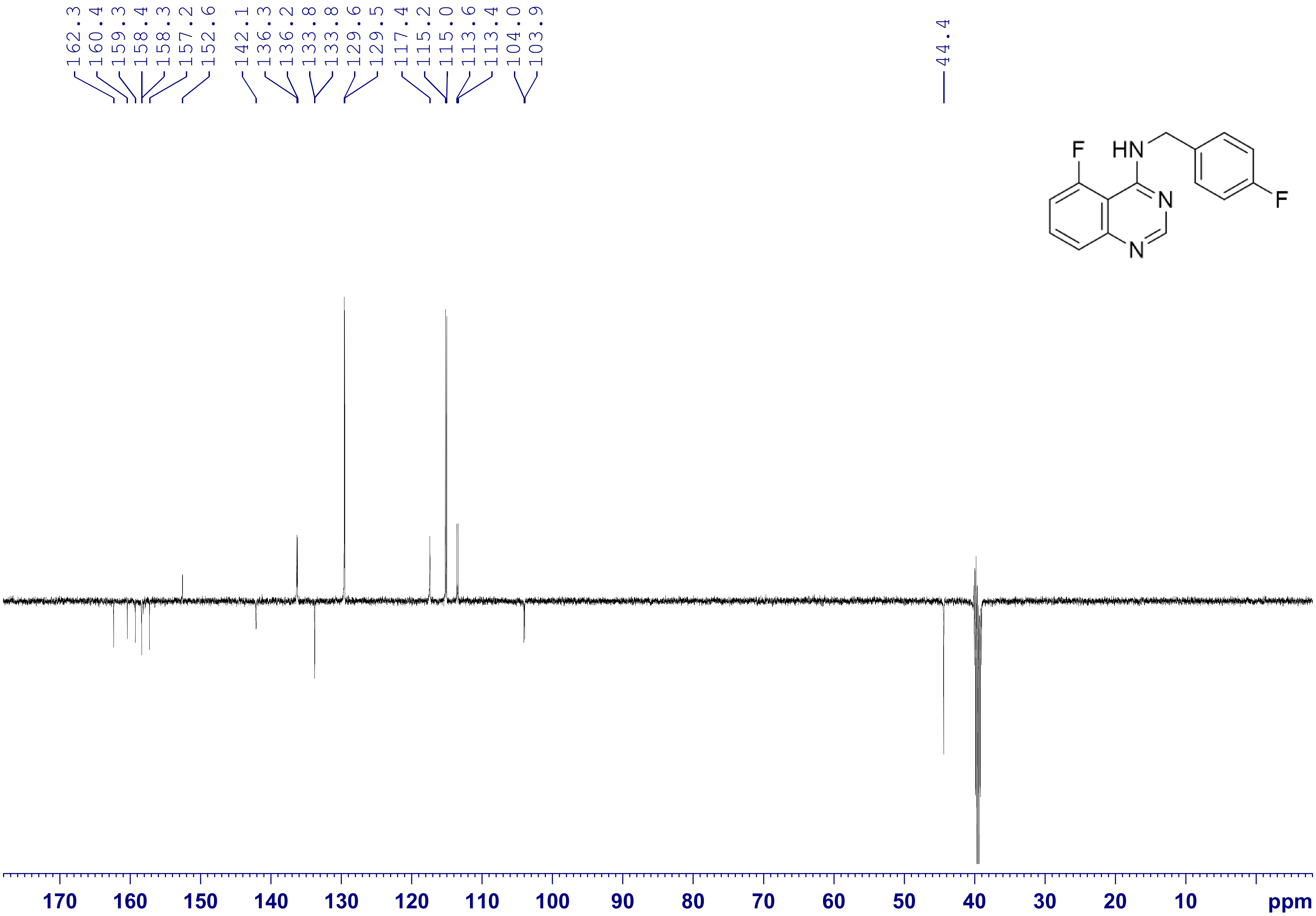
162.3
160.4
159.3
158.4
158.3
157.2
152.6
142.1
136.3
136.2
133.8
133.8
129.6
129.5
117.4
115.2
115.0
113.6
113.4
104.0
103.9
44.4
F
HN
N
N
F
170
160
150
140
130
120
110
100
90
80
70
60
50
40
30
20
10
ppm

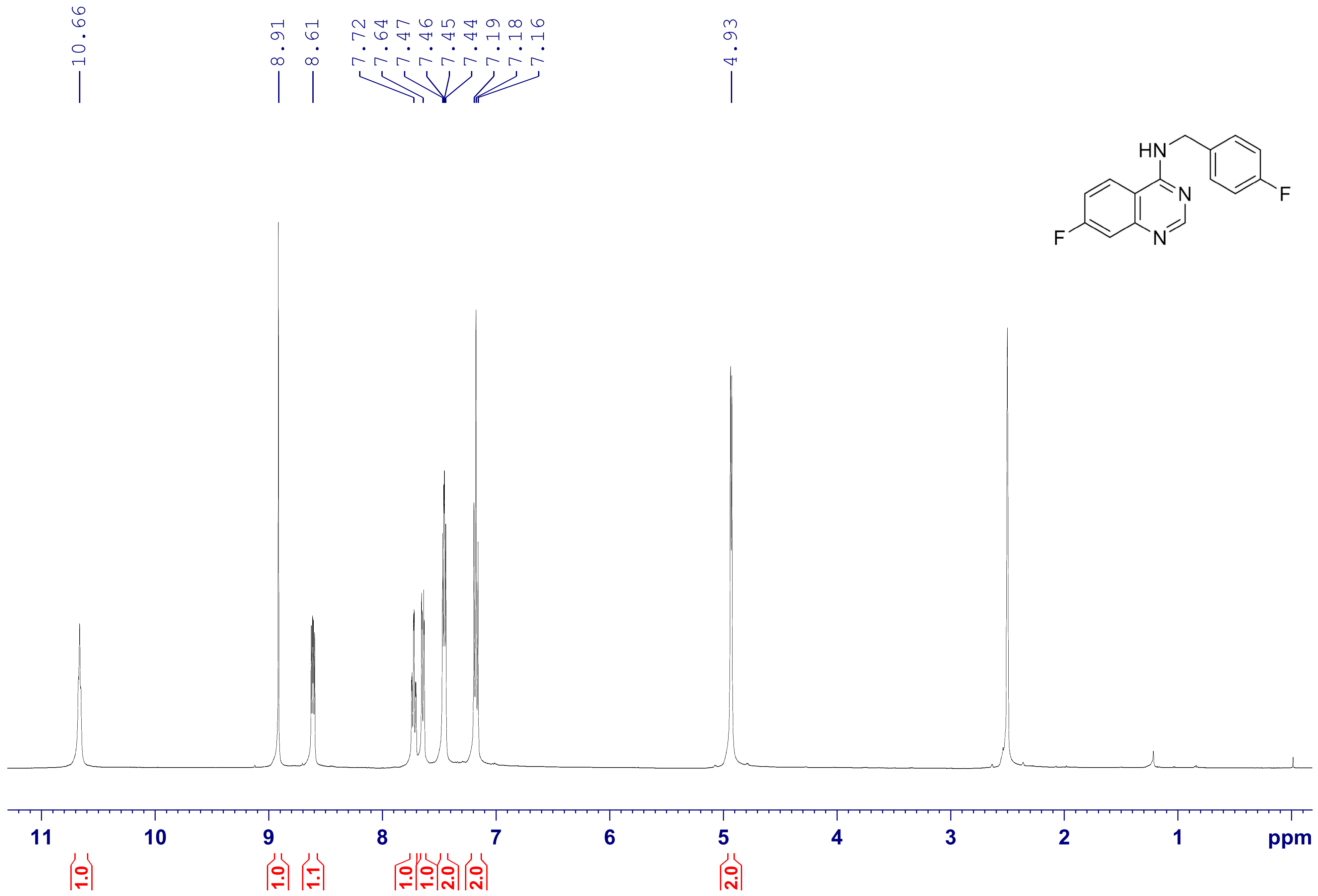
10.66
8.91
8.61
7.72
7.64
7.47
7.46
7.45
7.44
7.19
7.18
7.16
4.93
HN
N
N
F
F
11
10
9
8
7
6
5
4
3
2
1
ppm
1.0
1.0
1.1
1.0
1.0
2.0
2.0
2.0

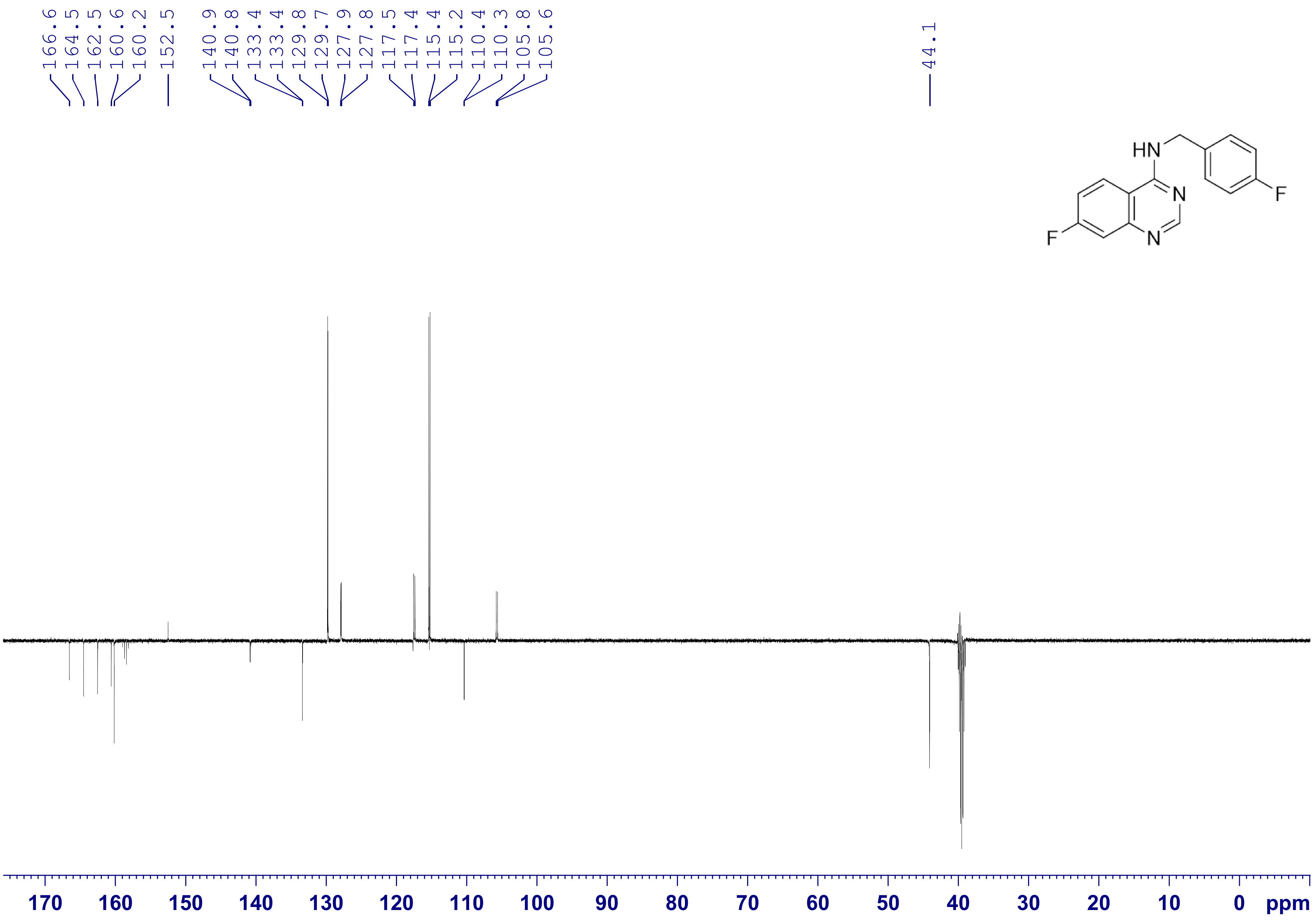
166.6
164.5
162.5
160.6
160.2
152.5
140.9
140.8
133.4
133.4
129.8
129.7
127.9
127.8
117.5
117.4
115.4
115.2
110.4
110.3
105.8
105.6
44.1
HN
N
N
F
F
170 160 150 140 130 120 110 100 90 80 70 60 50 40 30 20 10 0 ppm

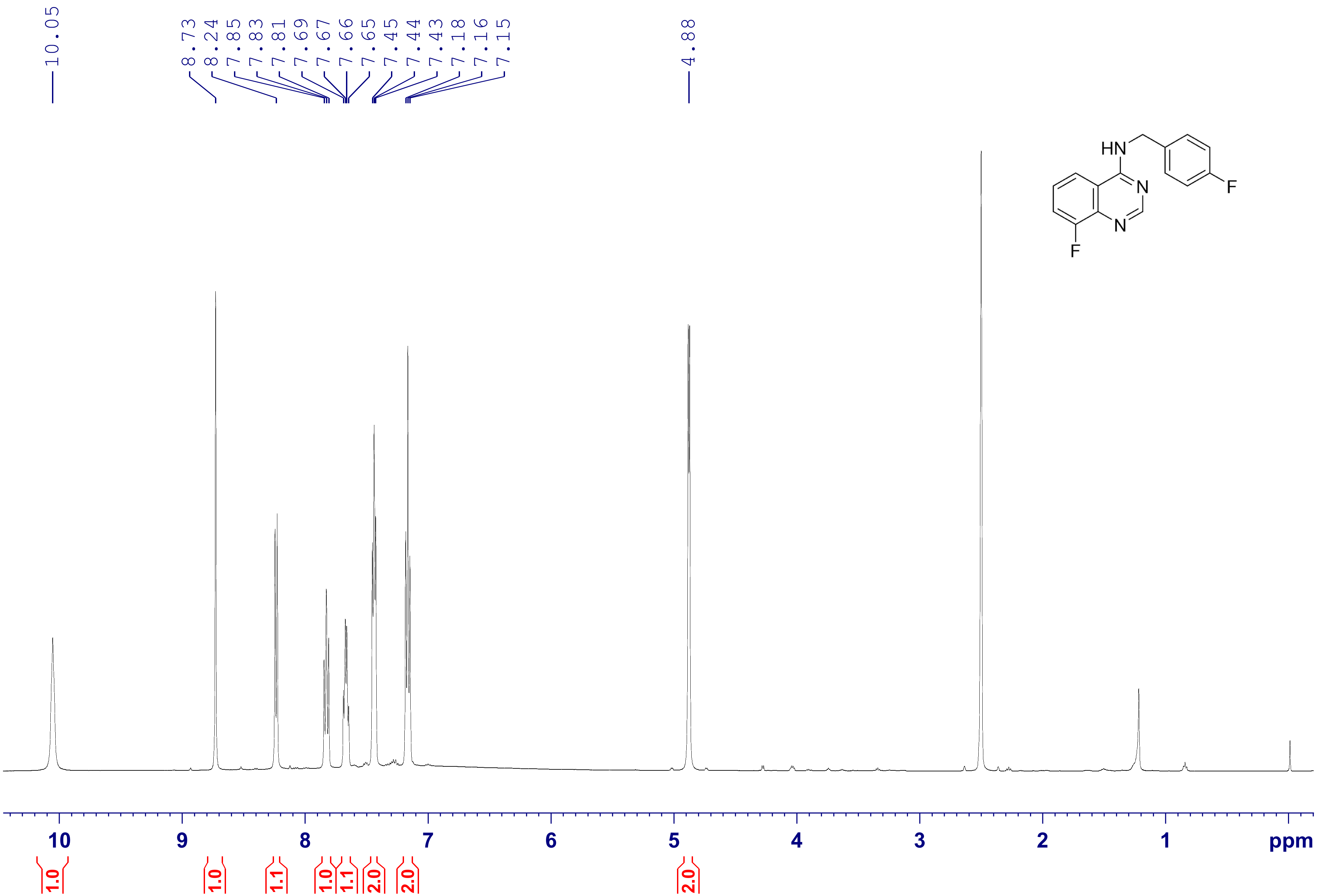

10.05
8.73
8.24
7.85
7.83
7.81
7.69
7.67
7.66
7.65
7.45
7.44
7.43
7.18
7.16
7.15
4.88
HN
N
N
F
F
10
9
8
7
6
5
4
3
2
1
ppm
1.0
1.0
1.1
1.0
1.1
2.0
2.0
2.0

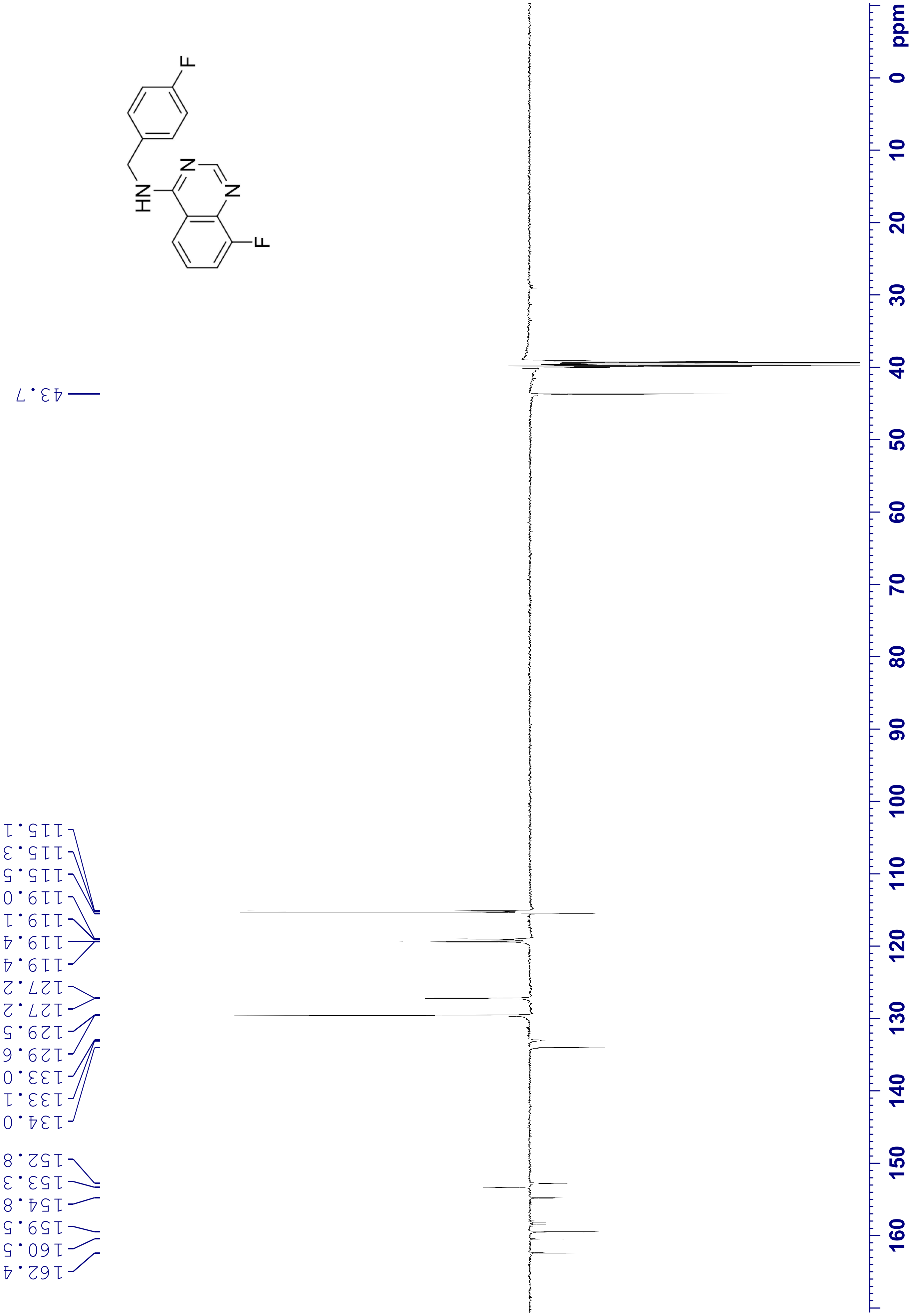

F
HN
N
N
F
ppm
0
10
20
30
40
50
60
70
80
90
100
110
120
130
140
150
160
43.7
115.1
115.3
115.5
119.0
119.1
119.4
119.4
127.2
127.2
129.5
129.6
133.0
133.1
134.0
152.8
153.3
154.8
159.5
160.5
162.4

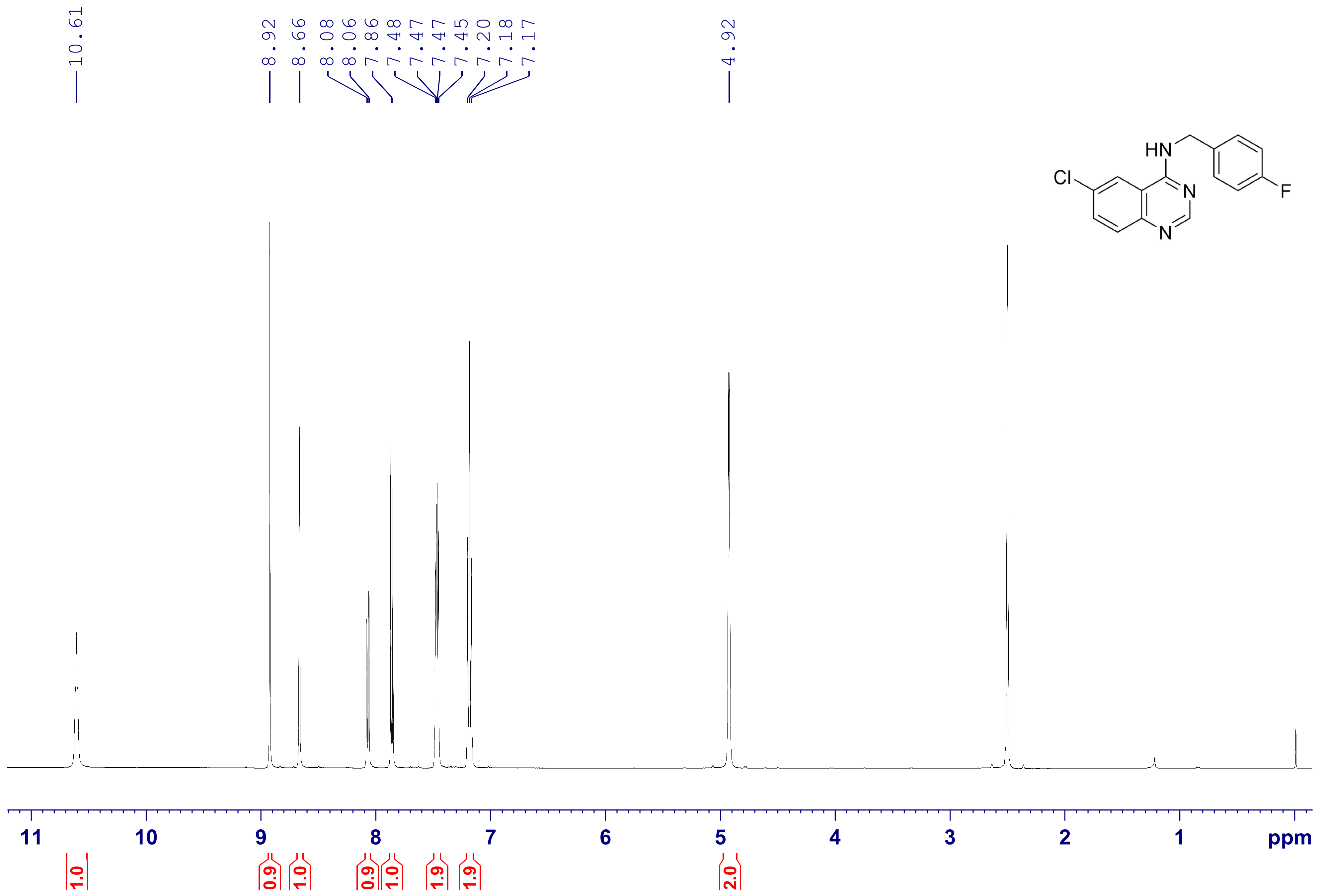
10.61
8.92
8.66
8.08
8.06
7.86
7.48
7.47
7.47
7.45
7.20
7.18
7.17
4.92
HN
Cl
N
N
F
11
10
9
8
7
6
5
4
3
2
1
ppm
1.0
0.9
1.0
0.9
1.0
1.9
1.9
2.0

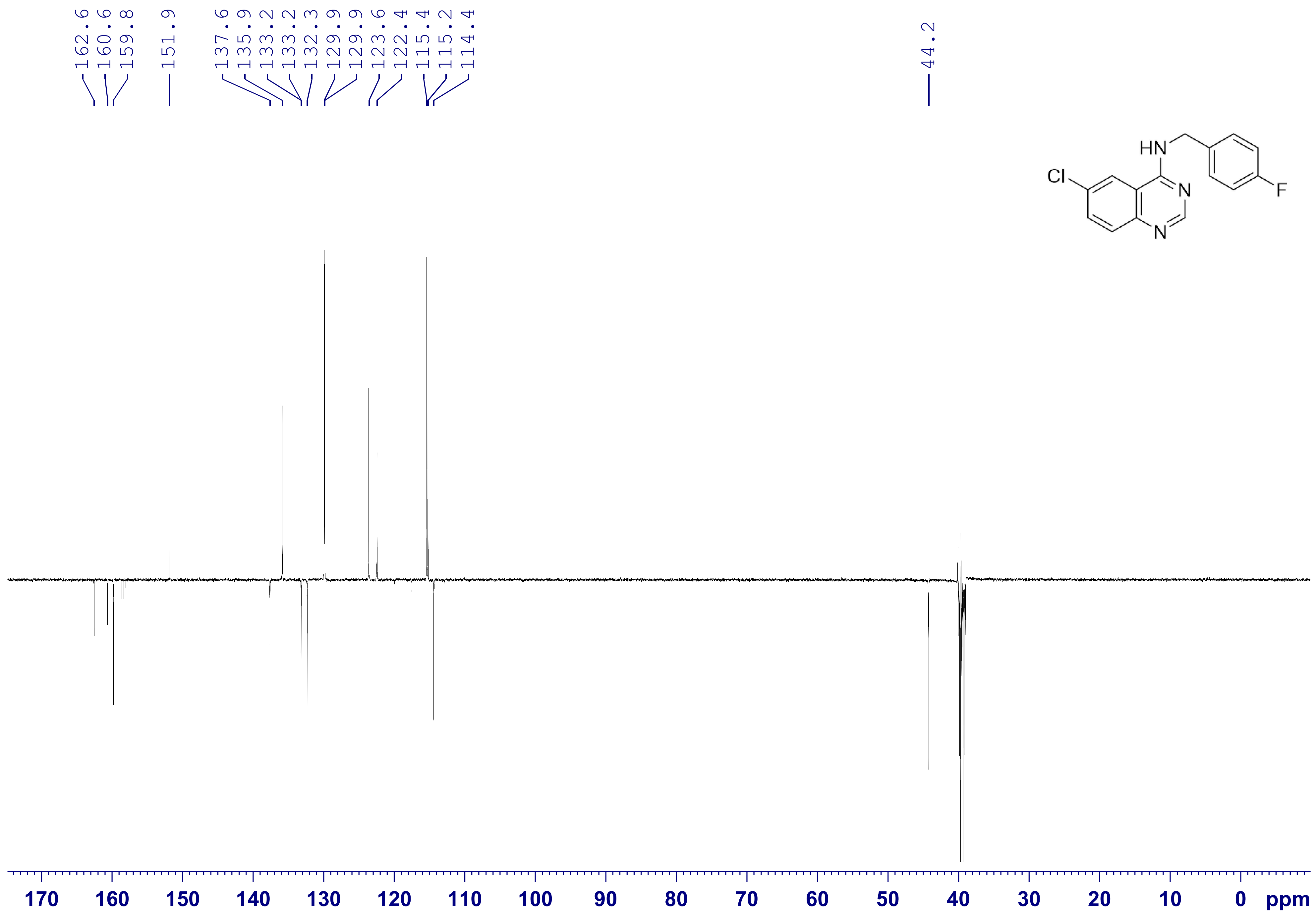

162.6
160.6
159.8
151.9
137.6
135.9
133.2
133.2
132.3
129.9
129.9
123.6
122.4
115.4
115.2
114.4
44.2
Cl
HN
N
N
F
170 160 150 140 130 120 110 100 90 80 70 60 50 40 30 20 10 0 ppm

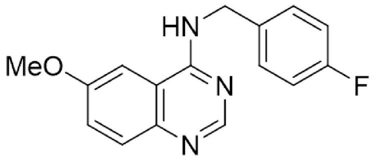
MeO
HN
N
N
F

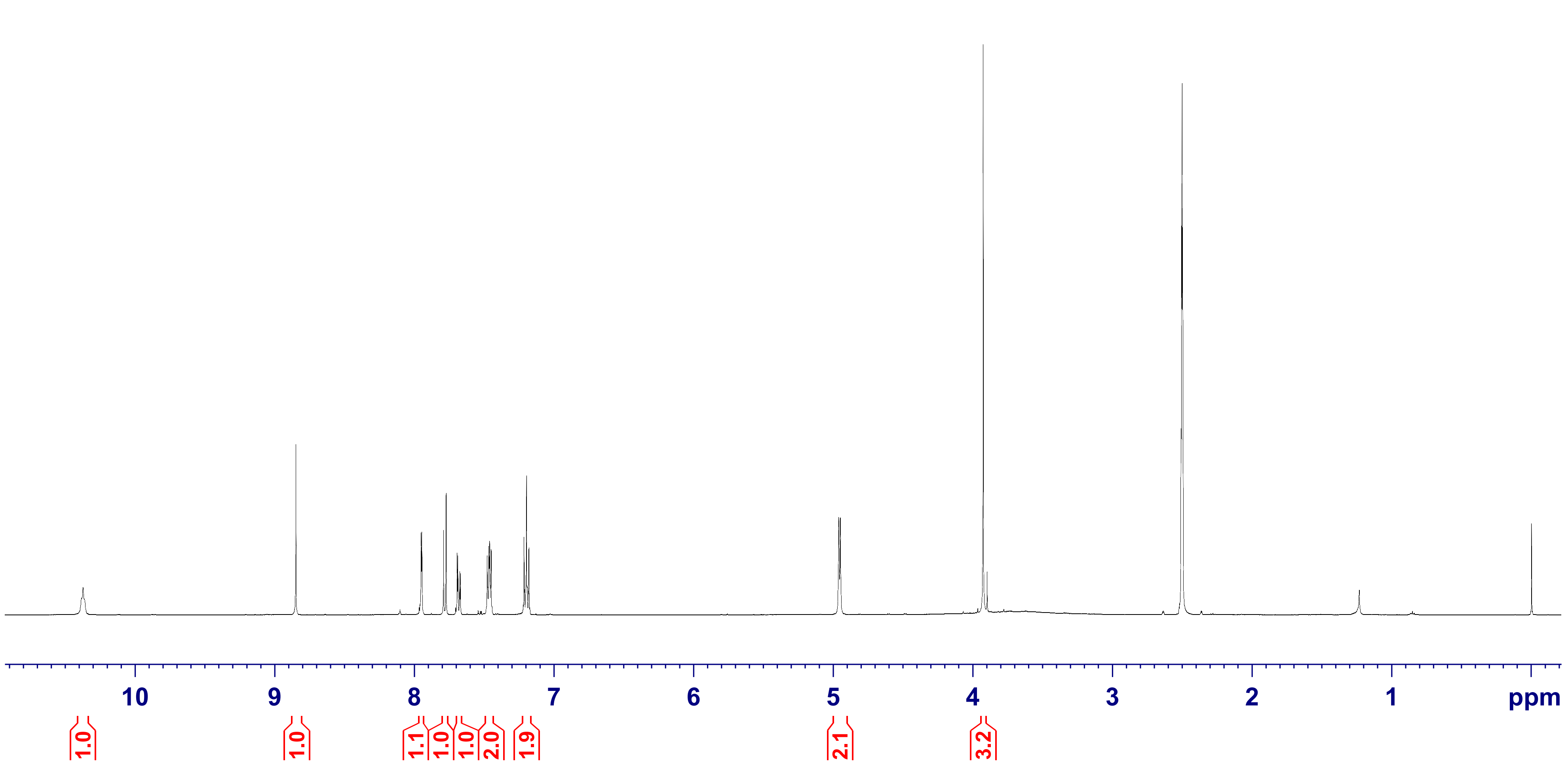
10.37
8.85
7.95
7.79
7.77
7.69
7.69
7.67
7.67
7.48
7.47
7.47
7.46
7.45
7.45
7.21
7.20
7.20
7.19
7.18
4.95
3.92
10
9
8
7
6
5
4
3
2
1
ppm
1.0
1.0
1.1
1.0
1.0
2.0
1.9
2.1
3.2

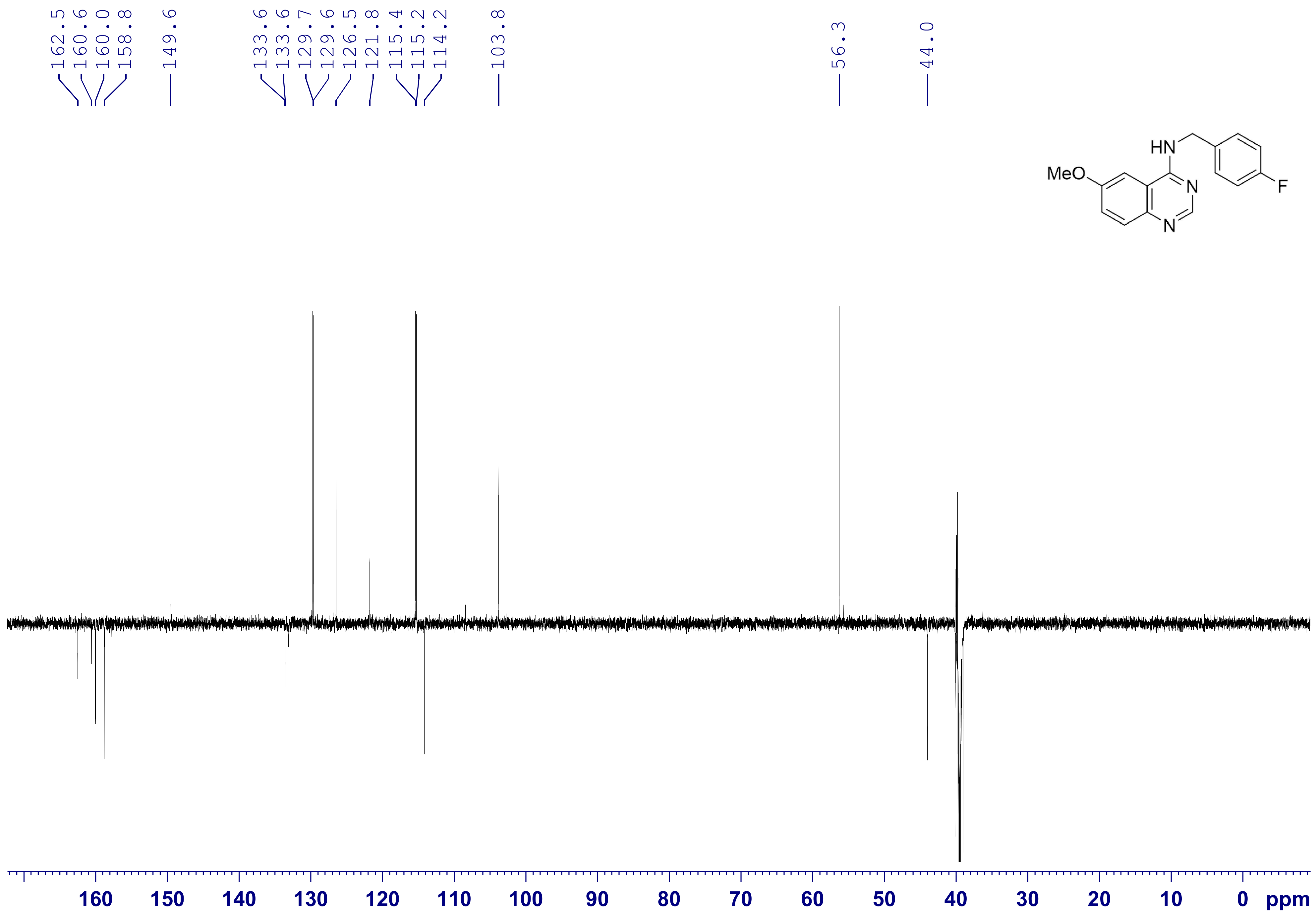

162.5
160.6
160.0
158.8
149.6
133.6
133.6
129.7
129.6
126.5
121.8
115.4
115.2
114.2
103.8
56.3
44.0
HN
MeO
N
N
F
160
150
140
130
120
110
100
90
80
70
60
50
40
30
20
10
0
ppm

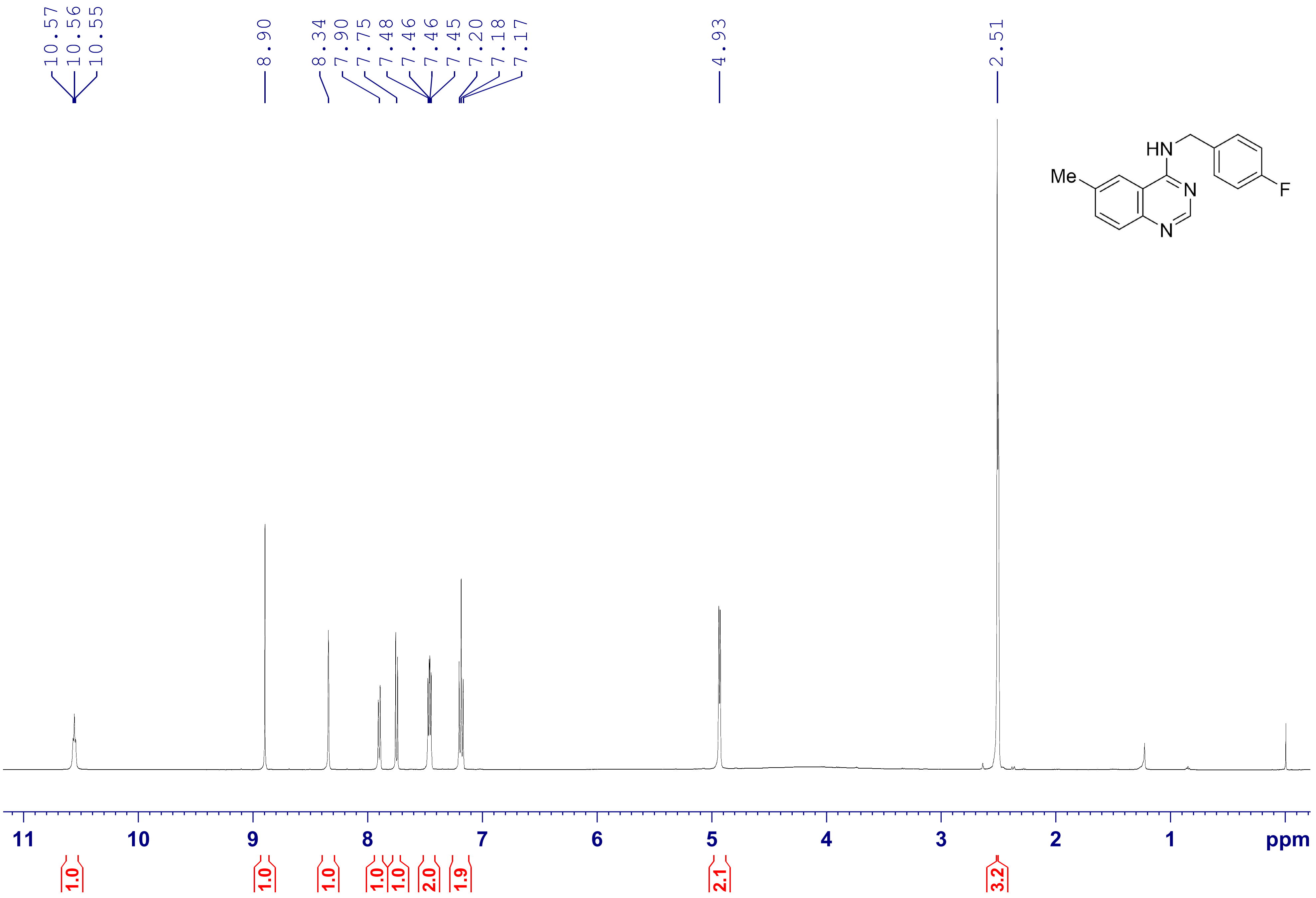
10.57
10.56
10.55
8.90
8.34
7.90
7.75
7.48
7.46
7.46
7.45
7.20
7.18
7.17
4.93
2.51
HN
N
N
Me
F
11
10
9
8
7
6
5
4
3
2
1
ppm
1.0
1.0
1.0
1.0
1.0
2.0
1.9
2.1
3.2

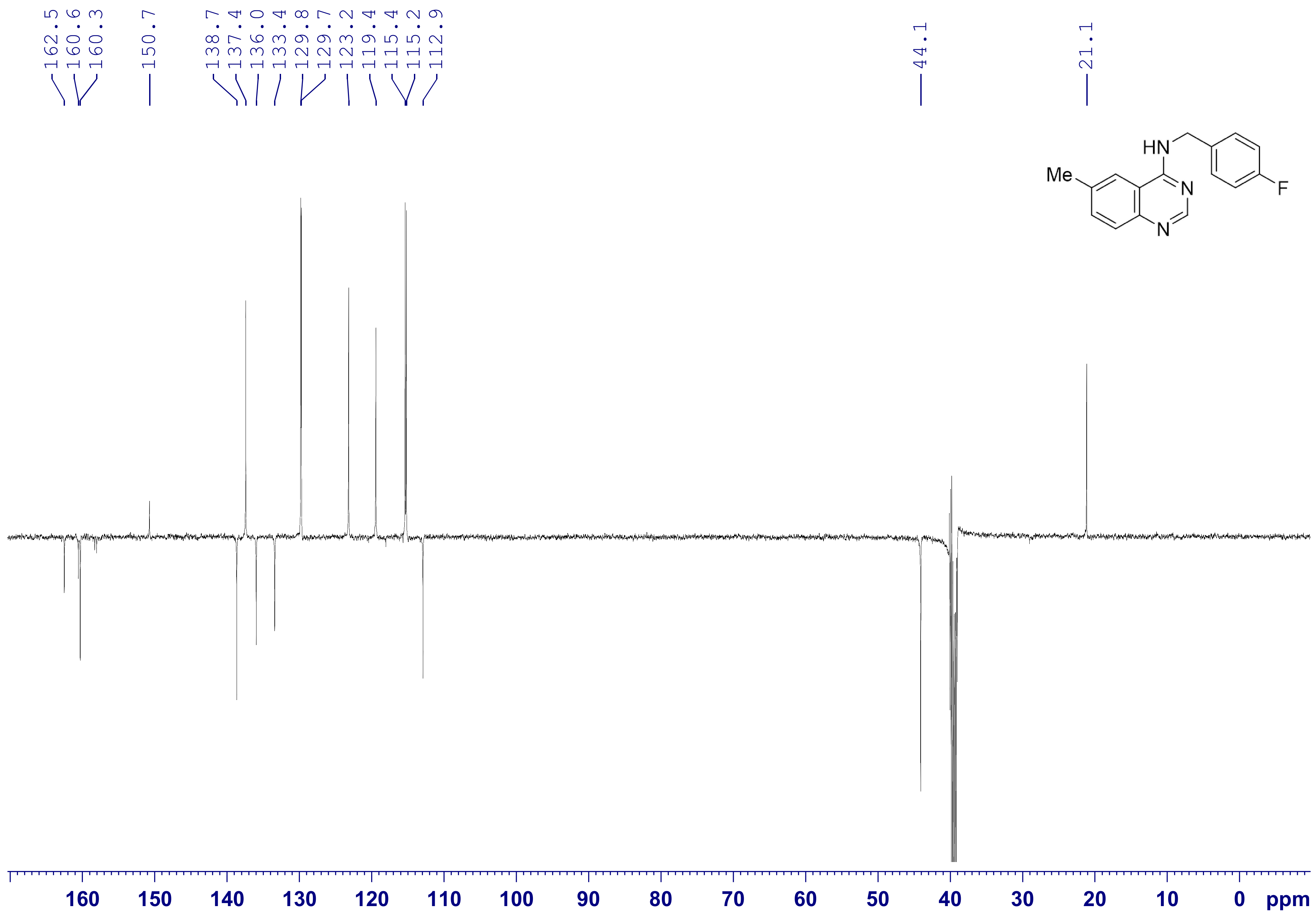
162.5
160.6
160.3
150.7
138.7
137.4
136.0
133.4
129.8
129.7
123.2
119.4
115.4
115.2
112.9
44.1
21.1
Me
HN
N
N
F
160
150
140
130
120
110
100
90
80
70
60
50
40
30
20
10
0
ppm

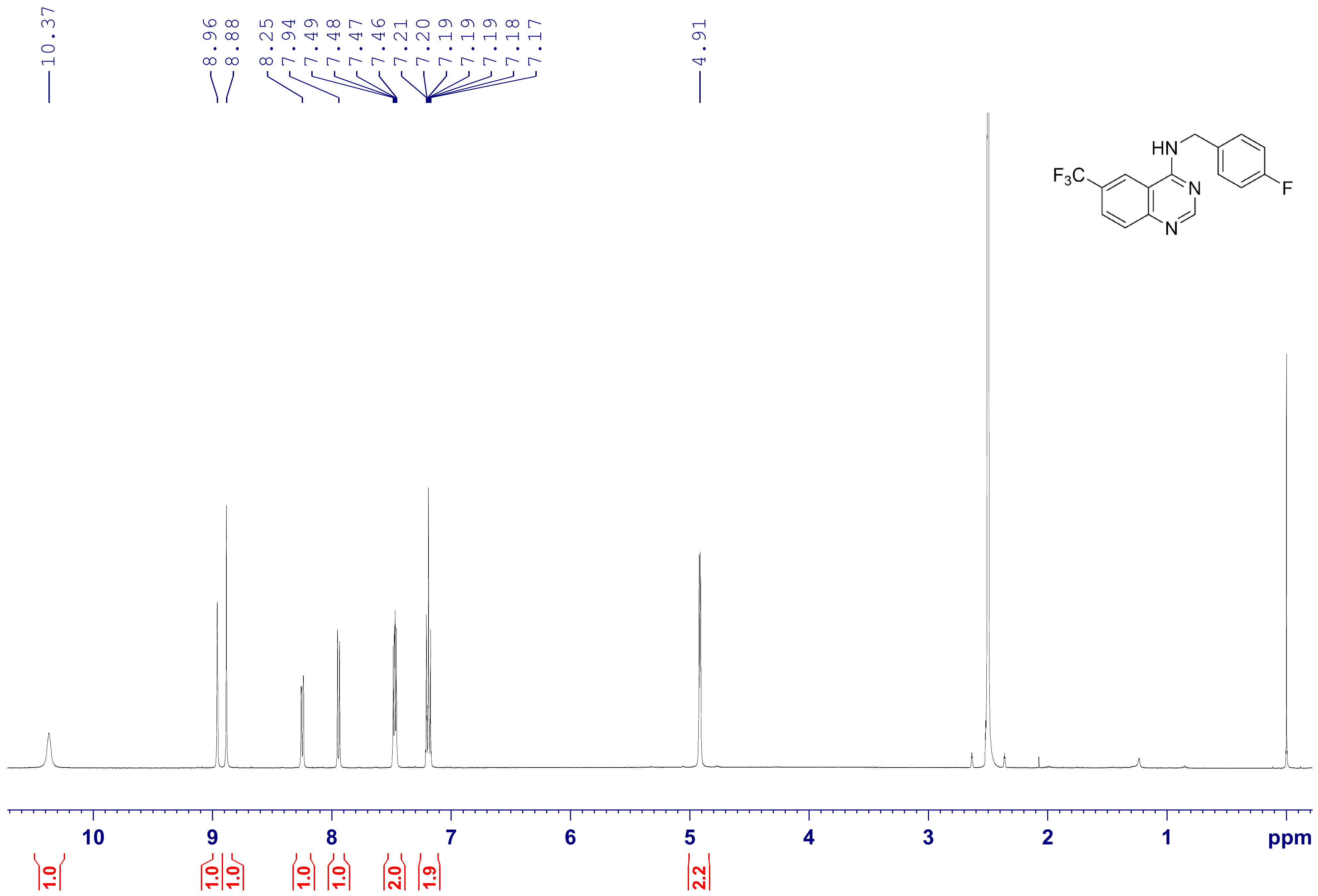

10.37
8.96
8.88
8.25
7.94
7.49
7.48
7.47
7.46
7.21
7.20
7.19
7.19
7.19
7.18
7.17
4.91
HN
N
N
F3C
F
10
9
8
7
6
5
4
3
2
1
ppm
1.0
1.0
1.0
1.0
1.0
2.0
1.9
2.2

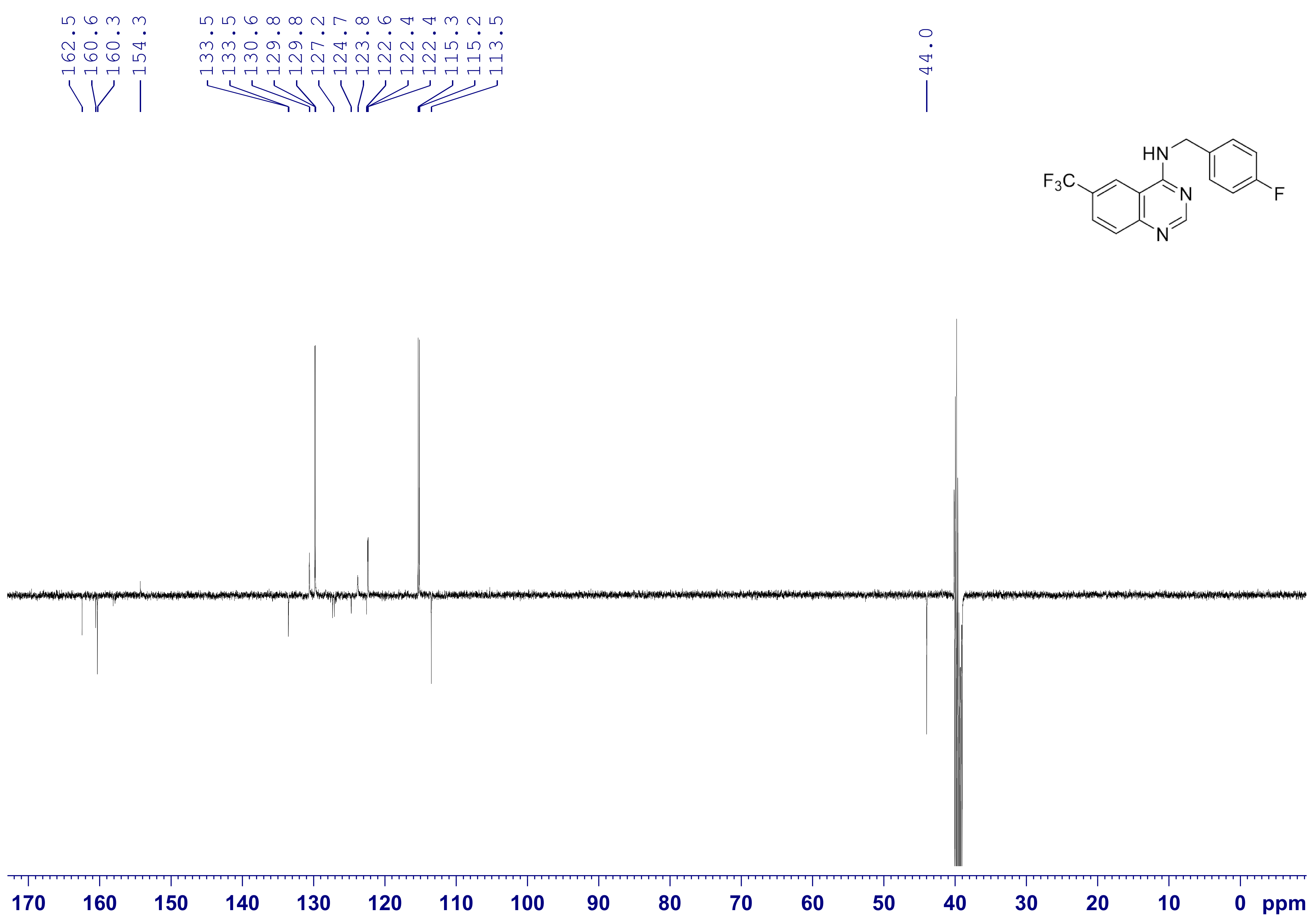

162.5
160.6
160.3
154.3
133.5
133.5
130.6
129.8
129.8
127.2
124.7
123.8
122.6
122.4
122.4
115.3
115.2
113.5
44.0
HN
F3C
N
N
F
170
160
150
140
130
120
110
100
90
80
70
60
50
40
30
20
10
0
ppm

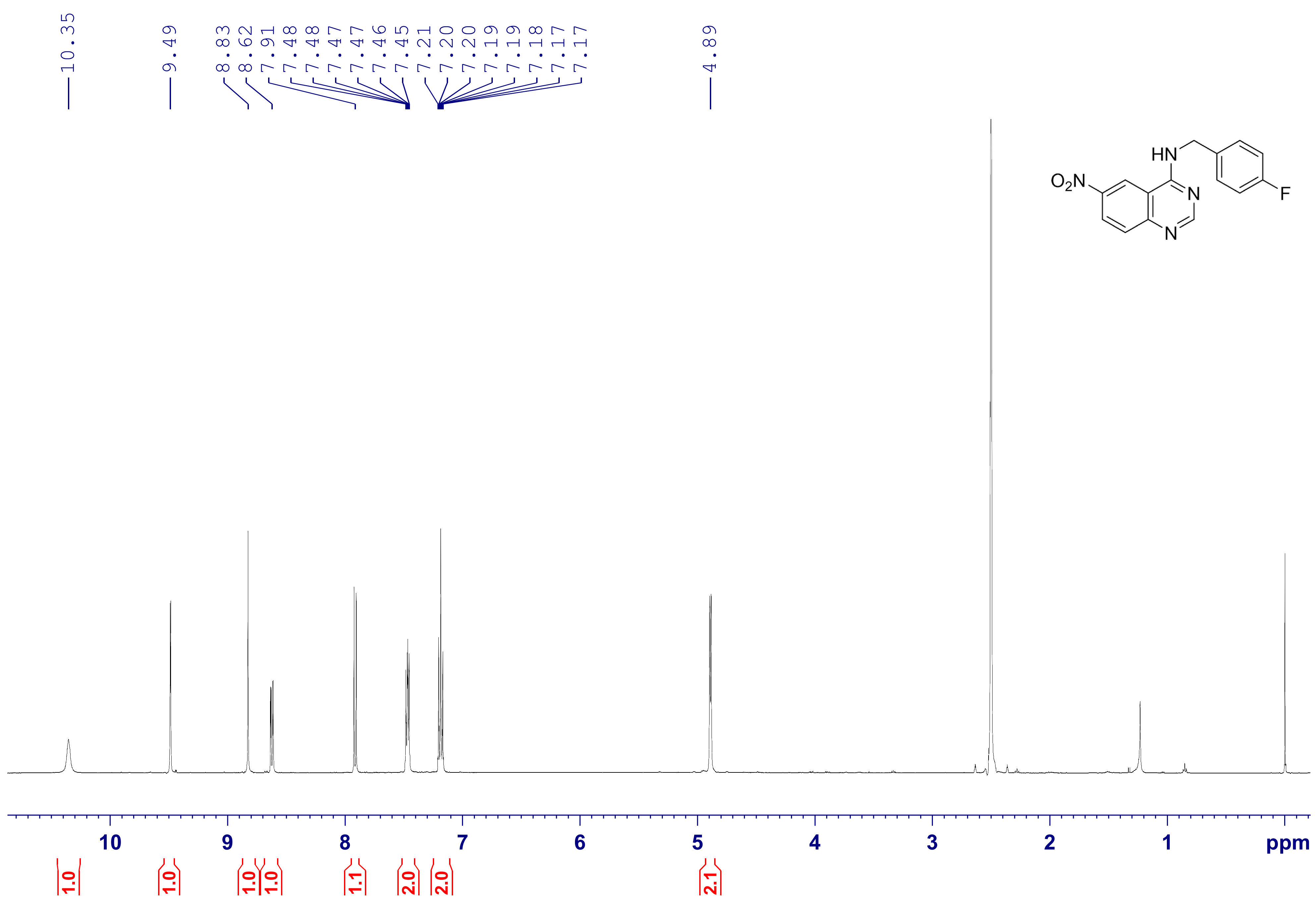

10.35
9.49
8.83
8.62
7.91
7.48
7.48
7.47
7.47
7.46
7.45
7.21
7.20
7.20
7.19
7.19
7.18
7.17
7.17
4.89
HN
O_2N
N
N
F
10
9
8
7
6
5
4
3
2
1
ppm
1.0
1.0
1.0
1.0
1.1
2.0
2.0
2.1

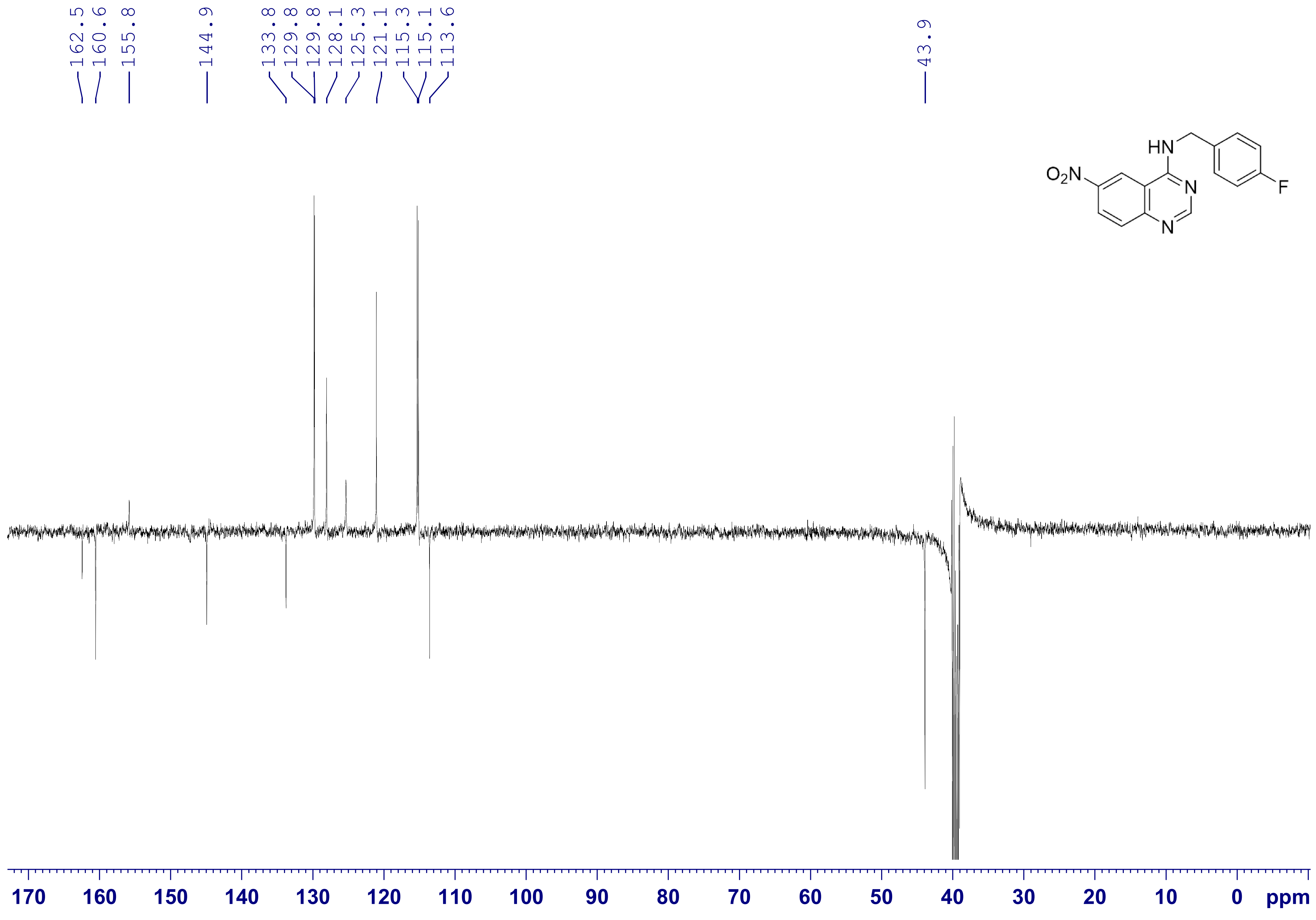
162.5
160.6
155.8
144.9
133.8
129.8
129.8
128.1
125.3
121.1
115.3
115.1
113.6
43.9
HN
O_2N
N
N
F
170
160
150
140
130
120
110
100
90
80
70
60
50
40
30
20
10
0
ppm

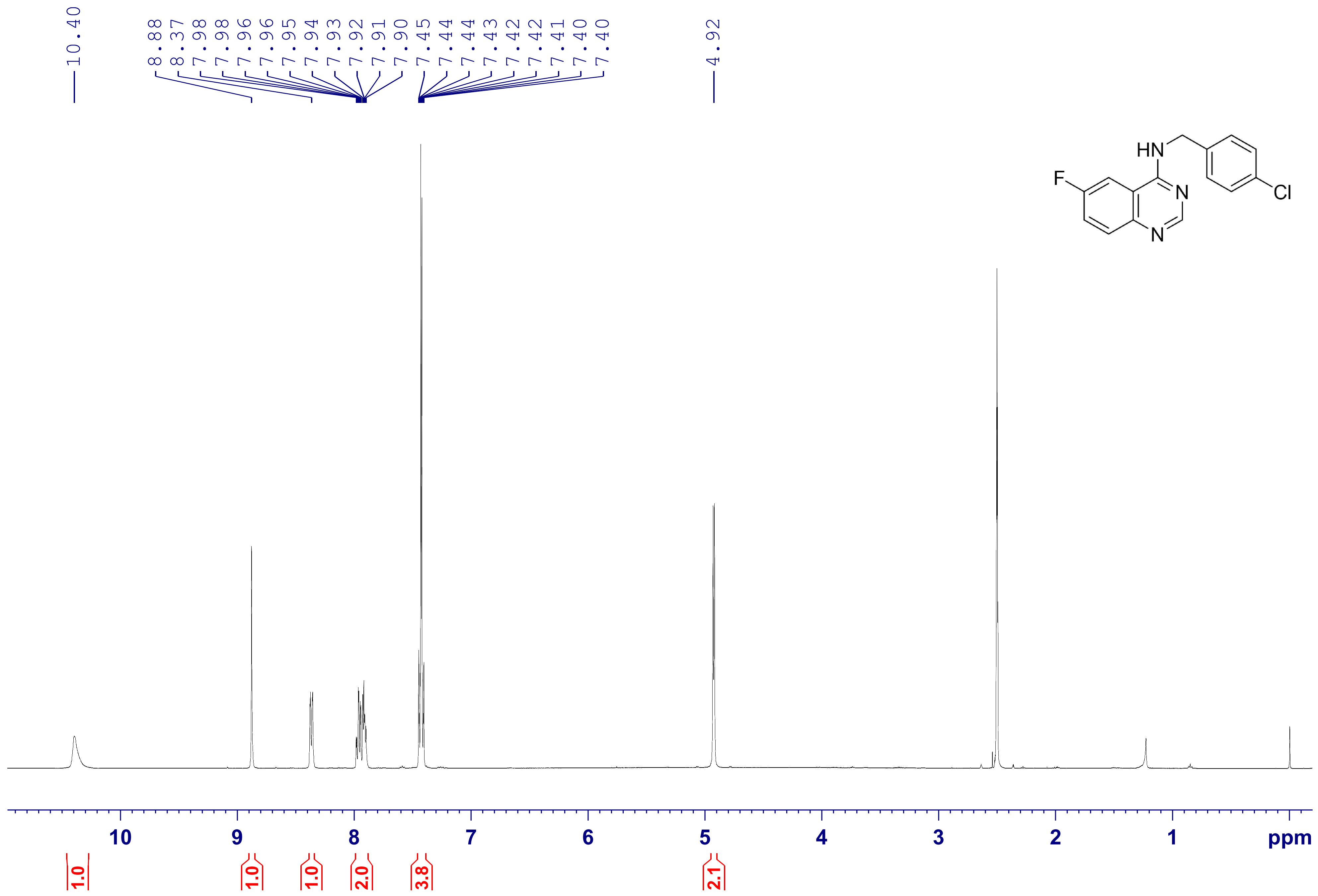

10.40
8.88
8.37
7.98
7.98
7.96
7.96
7.95
7.94
7.93
7.92
7.91
7.90
7.45
7.44
7.44
7.43
7.42
7.42
7.41
7.40
7.40
4.92
HN
F
N
N
Cl
10
9
8
7
6
5
4
3
2
1
ppm
1.0
1.0
1.0
2.0
3.8
2.1

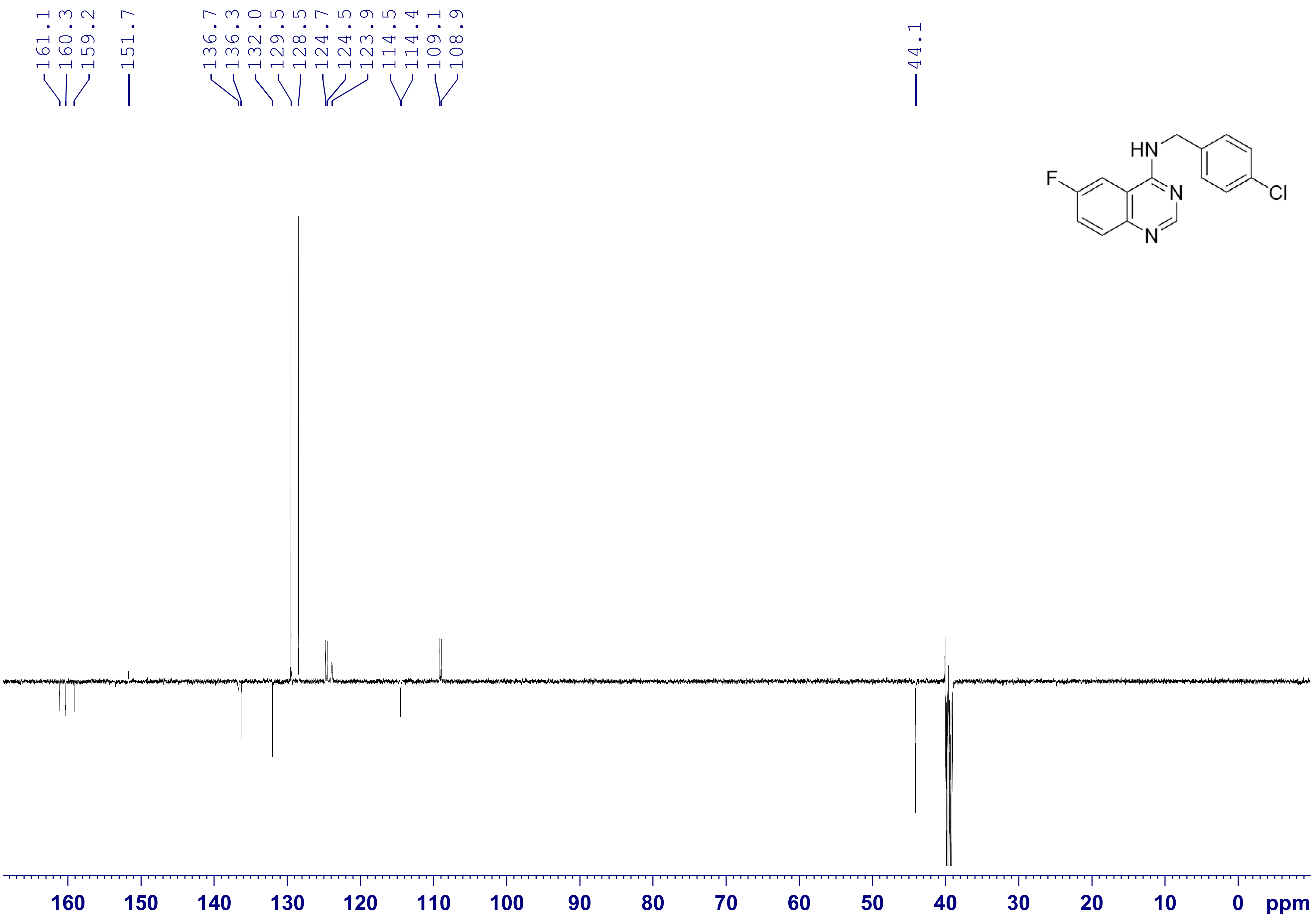
161.1
160.3
159.2
151.7
136.7
136.3
132.0
129.5
128.5
124.7
124.5
123.9
114.5
114.4
109.1
108.9
44.1
HN
F
N
N
Cl
160
150
140
130
120
110
100
90
80
70
60
50
40
30
20
10
0
ppm

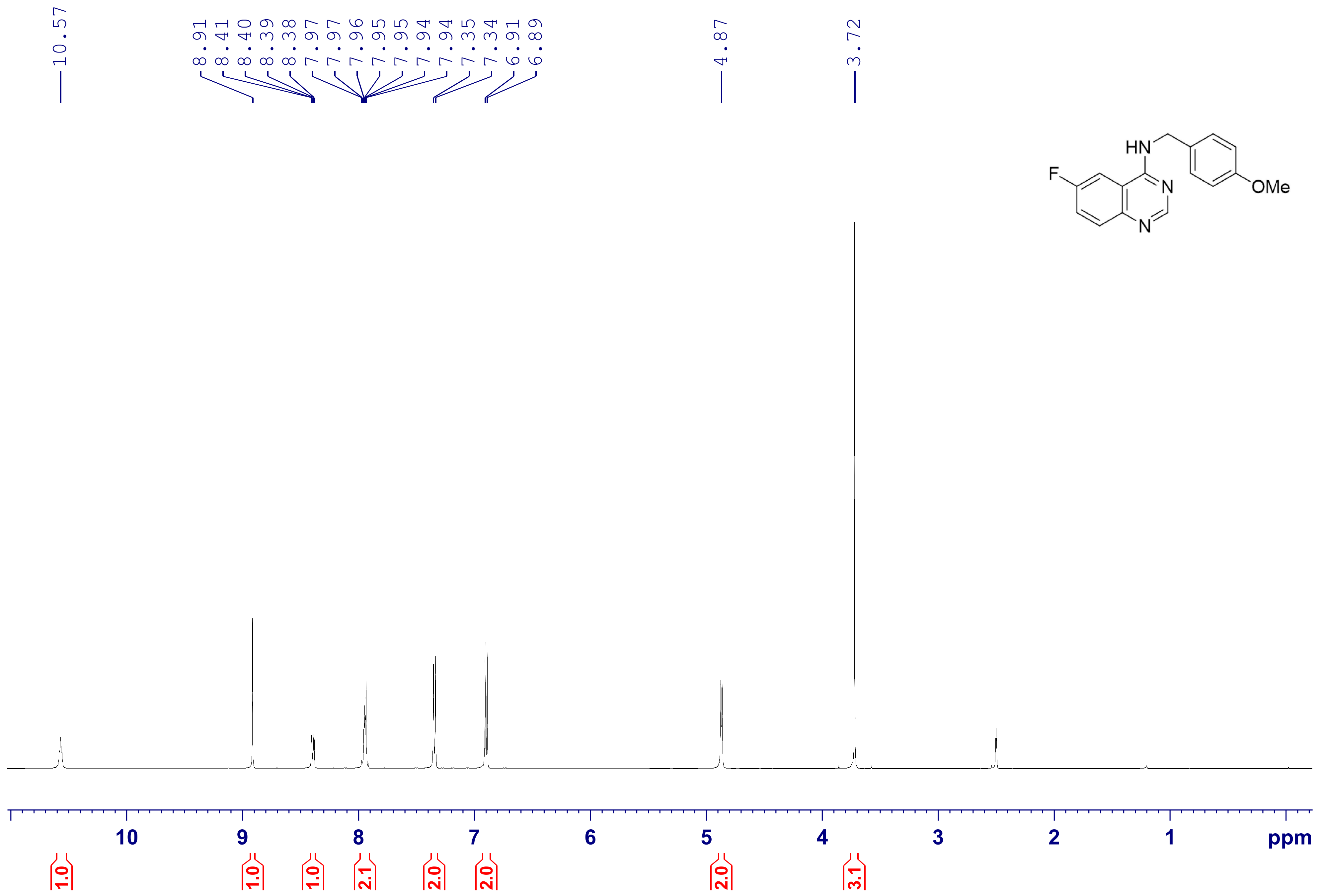
10.57
8.91
8.41
8.40
8.39
8.38
7.97
7.97
7.96
7.95
7.95
7.94
7.94
7.35
7.34
6.91
6.89
4.87
3.72
F
HN
N
N
OMe
10
9
8
7
6
5
4
3
2
1
ppm
1.0
1.0
1.0
2.1
2.0
2.0
2.0
3.1

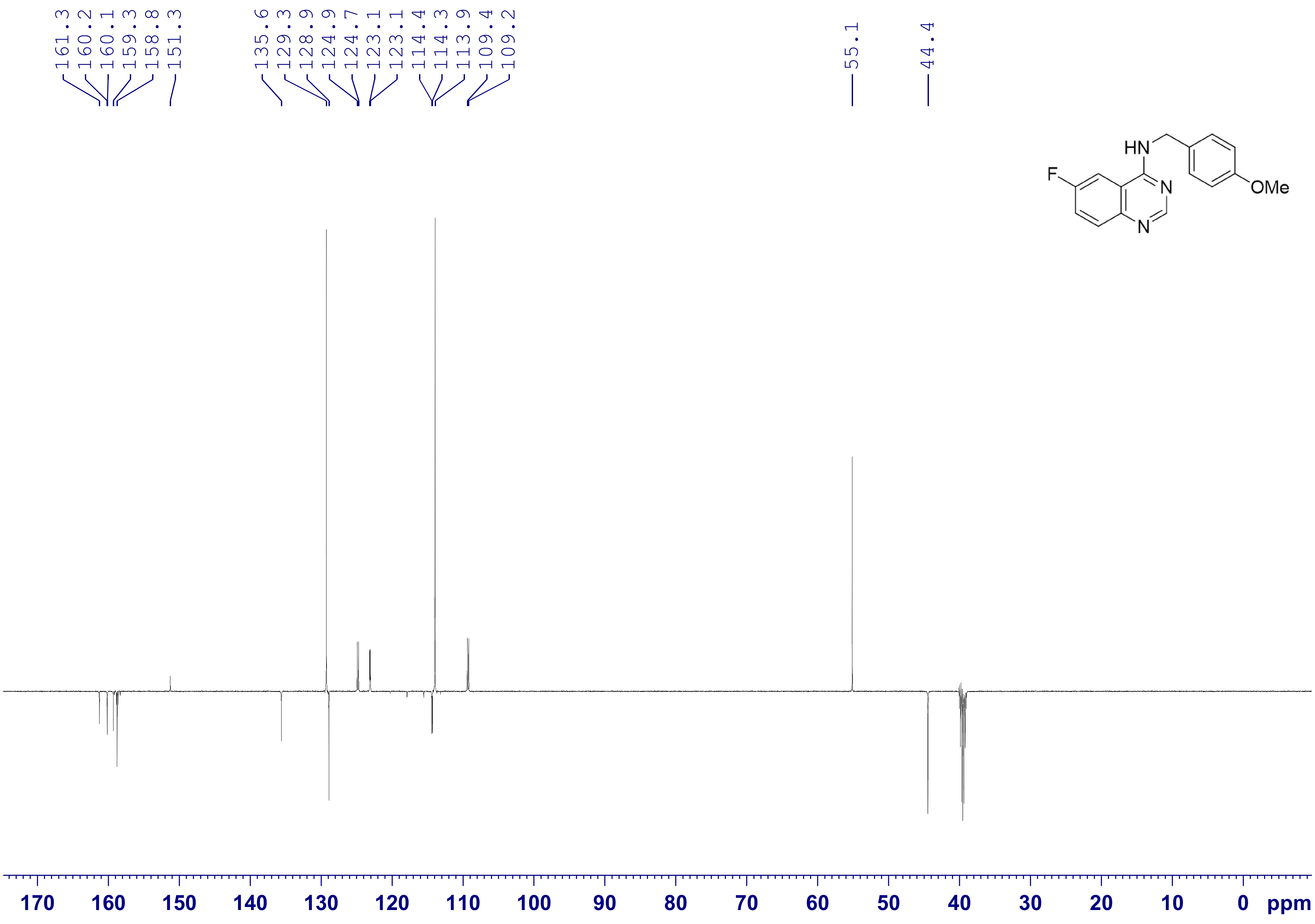
161.3
160.2
160.1
159.3
158.8
151.3
135.6
129.3
128.9
124.9
124.7
123.1
123.1
114.4
114.3
113.9
109.4
109.2
55.1
44.4
HN
F
N
N
OMe
170
160
150
140
130
120
110
100
90
80
70
60
50
40
30
20
10
0
ppm

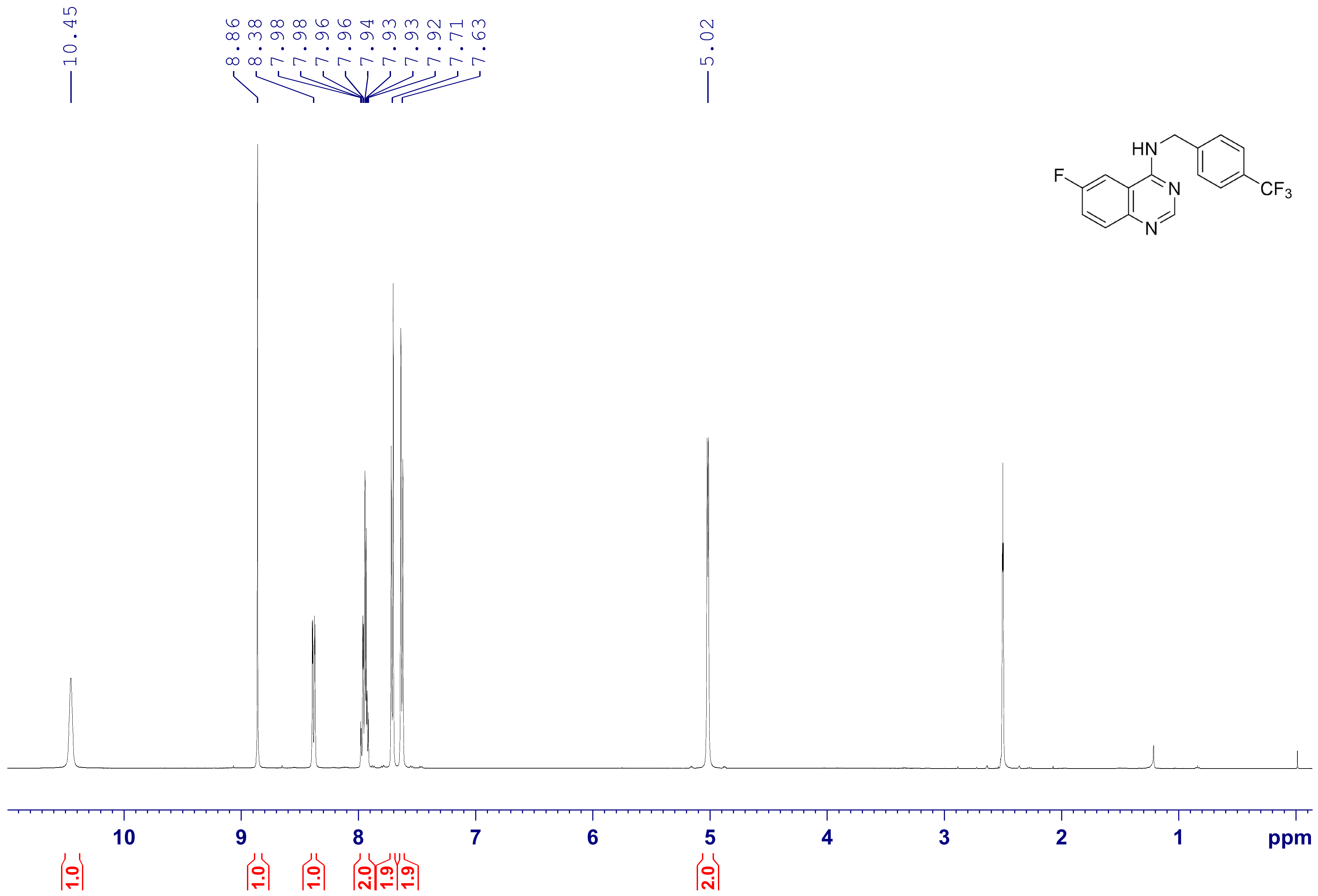
10.45
8.86
8.38
7.98
7.98
7.96
7.96
7.94
7.93
7.93
7.92
7.71
7.63
5.02
10
9
8
7
6
5
4
3
2
1
ppm
1.0
1.0
1.0
2.0
1.9
1.9
2.0
HN
F
N
N
CF3

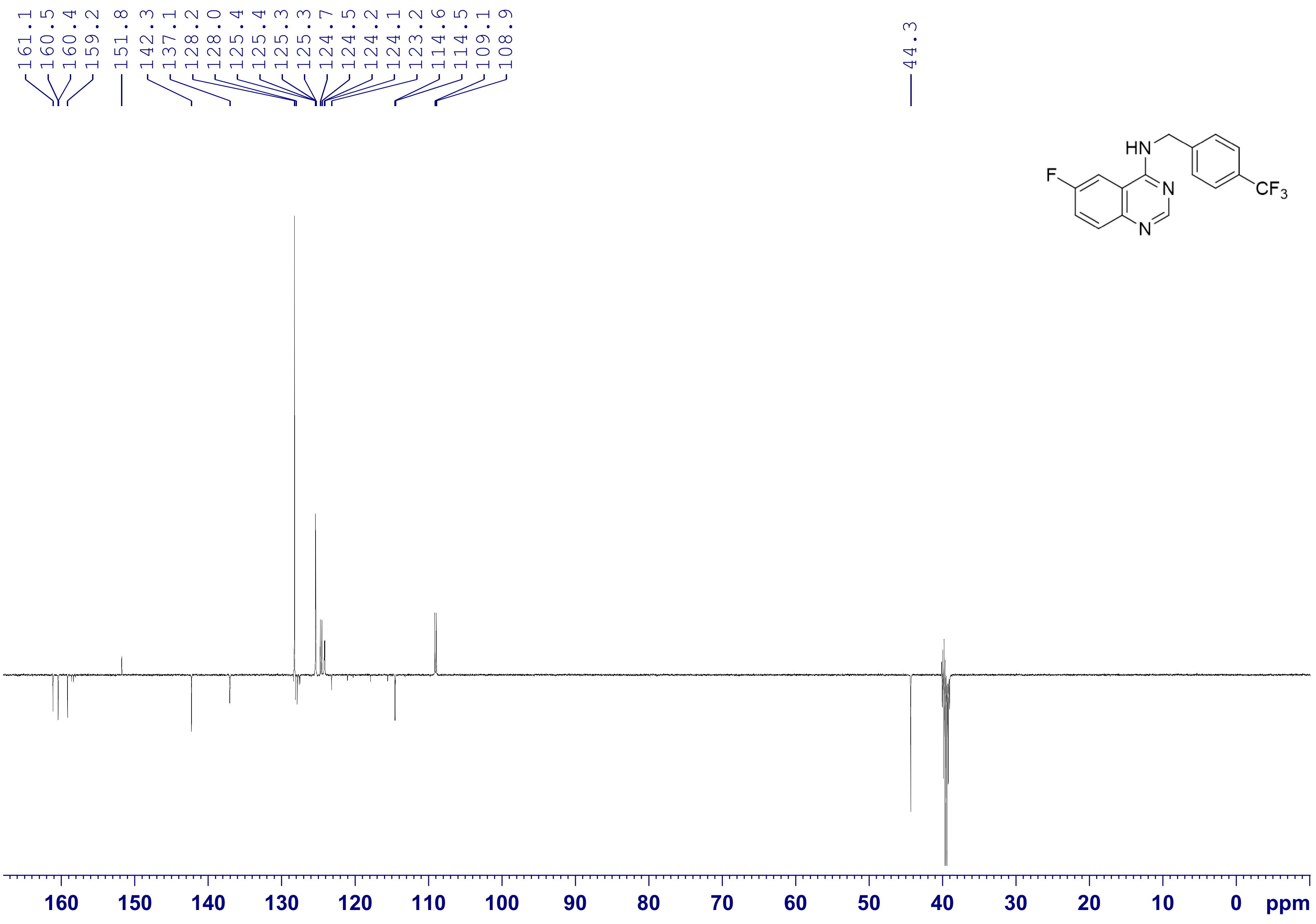
161.1
160.5
160.4
159.2
151.8
142.3
137.1
128.2
128.0
125.4
125.4
125.3
125.3
124.7
124.5
124.2
124.1
123.2
114.6
114.5
109.1
108.9
44.3
HN
F
N
N
CF_3
160
150
140
130
120
110
100
90
80
70
60
50
40
30
20
10
0
ppm

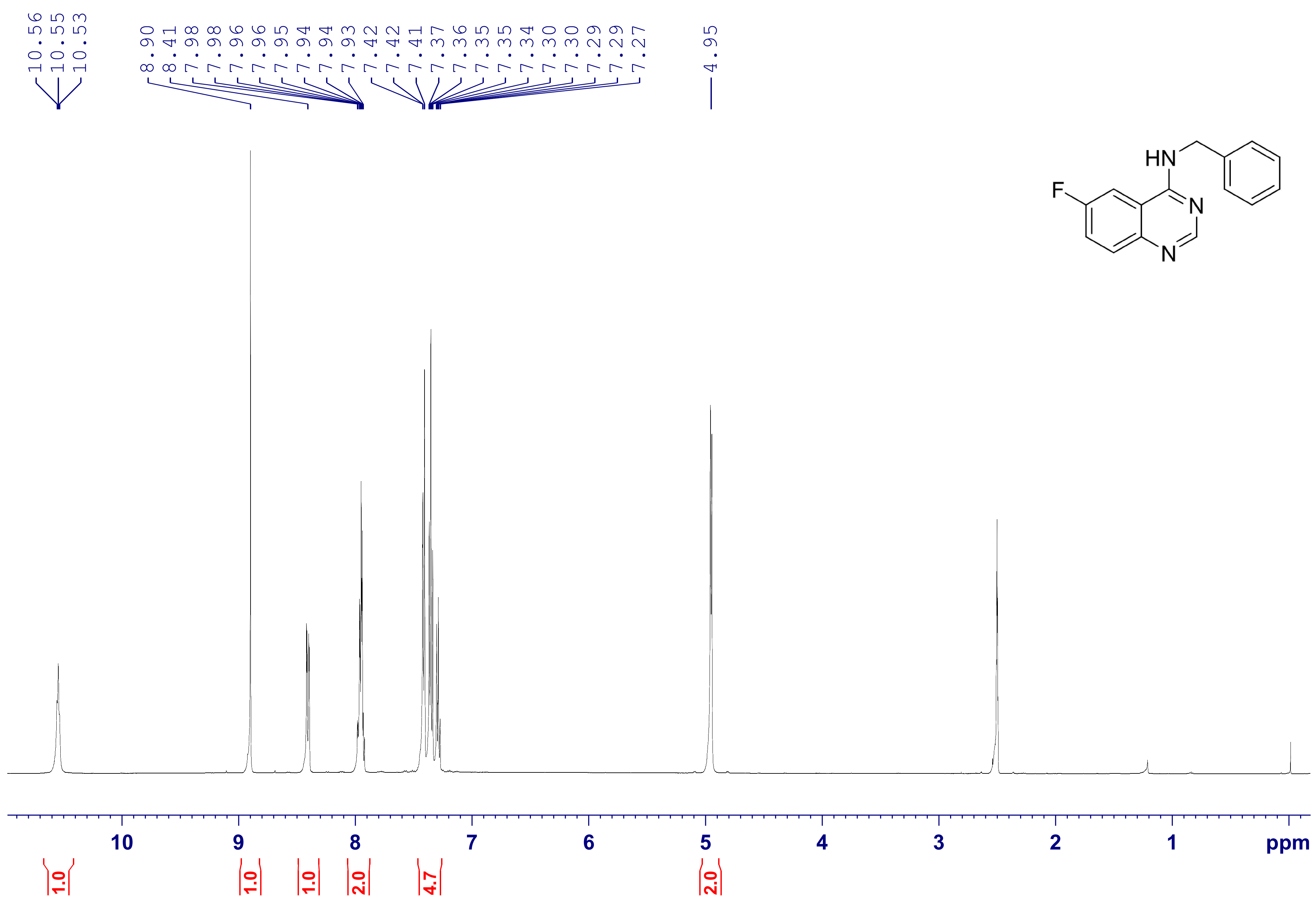
10.56
10.55
10.53
8.90
8.41
7.98
7.98
7.96
7.96
7.95
7.94
7.94
7.93
7.42
7.42
7.41
7.37
7.36
7.35
7.35
7.34
7.30
7.30
7.29
7.29
7.27
4.95
F
HN
N
N
10
9
8
7
6
5
4
3
2
1
ppm
1.0
1.0
1.0
2.0
4.7
2.0

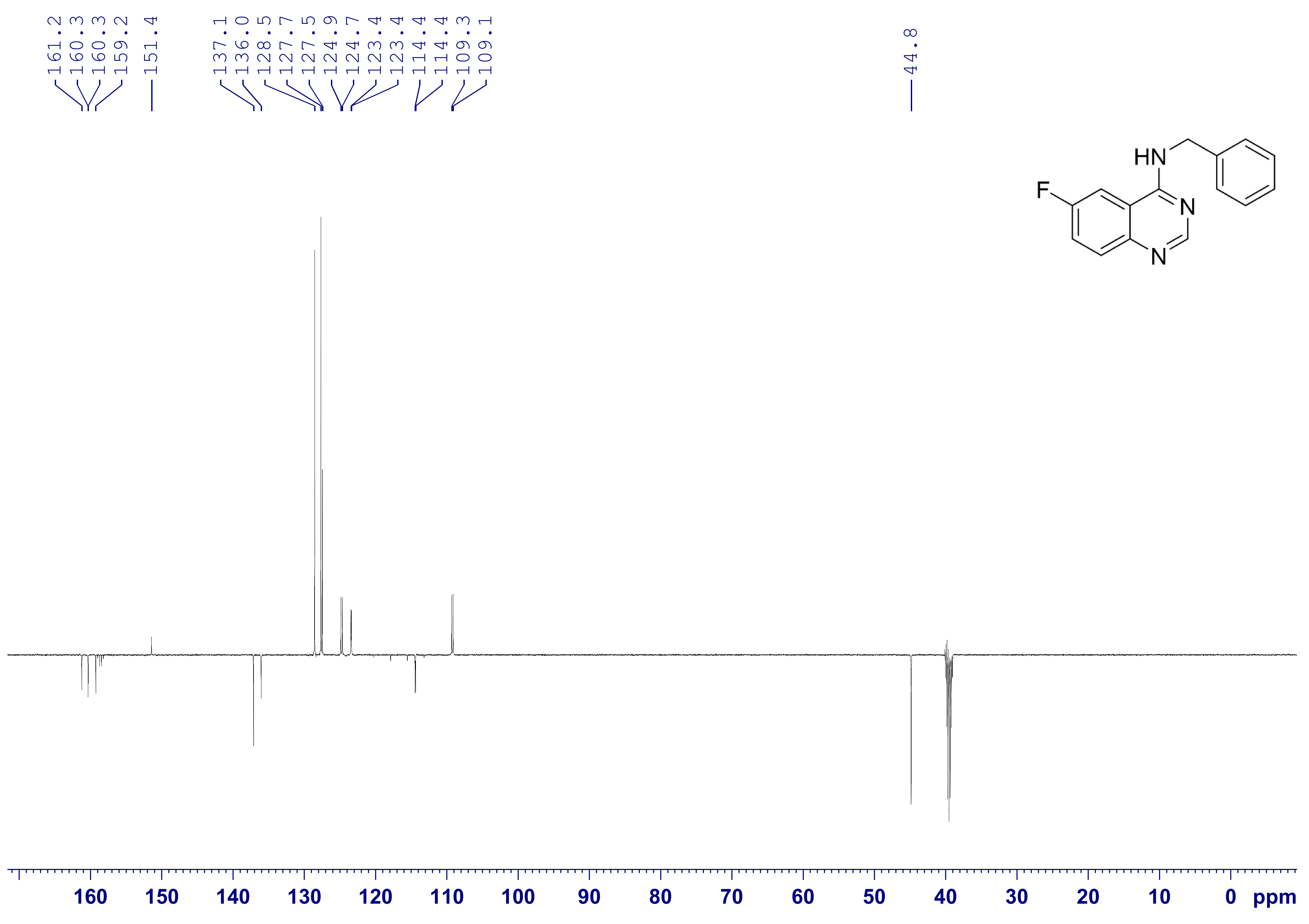

161.2
160.3
160.3
159.2
151.4
137.1
136.0
128.5
127.7
127.5
124.9
124.7
123.4
123.4
114.4
114.4
109.3
109.1
44.8
F
HN
N
N
160
150
140
130
120
110
100
90
80
70
60
50
40
30
20
10
0
ppm

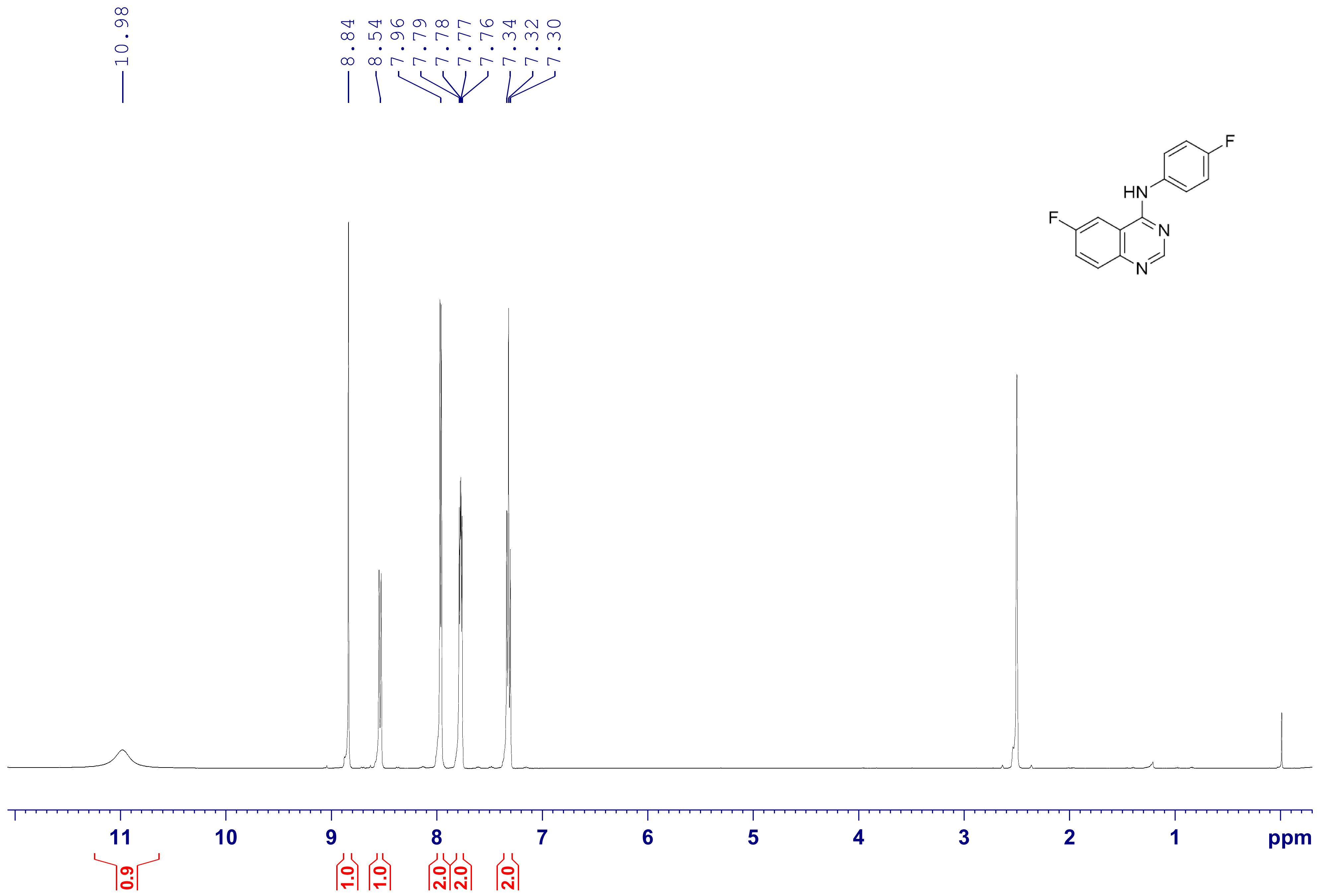
10.98
8.84
8.54
7.96
7.79
7.78
7.77
7.76
7.34
7.32
7.30
F
HN
N
N
F
11
10
9
8
7
6
5
4
3
2
1
ppm
0.9
1.0
1.0
2.0
2.0
2.0

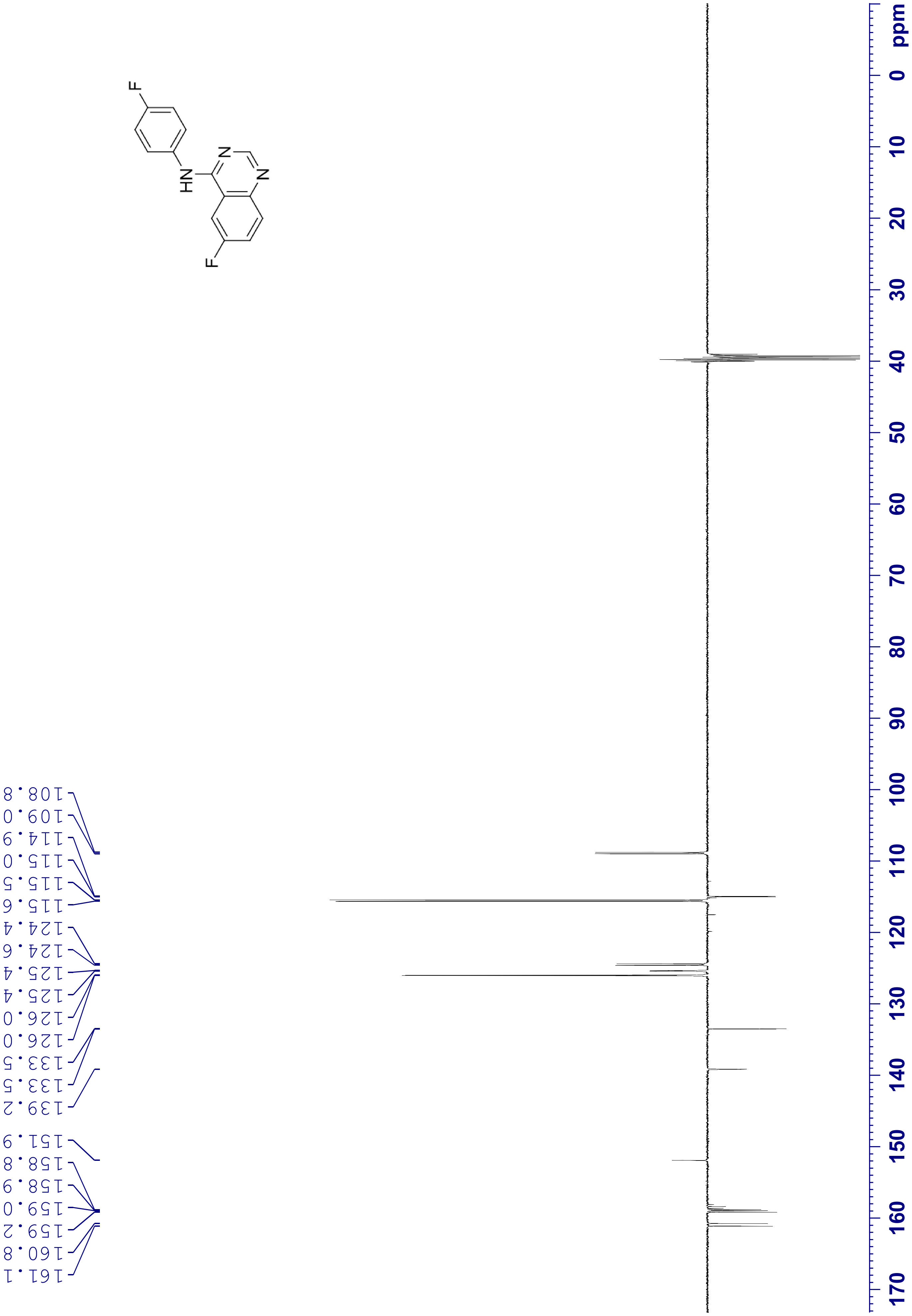

108.8
109.0
114.9
115.0
115.5
115.6
124.4
124.6
125.4
125.4
126.0
126.0
133.5
133.5
139.2
151.9
158.8
158.9
159.0
159.2
160.8
161.1
ppm
HN
N
F

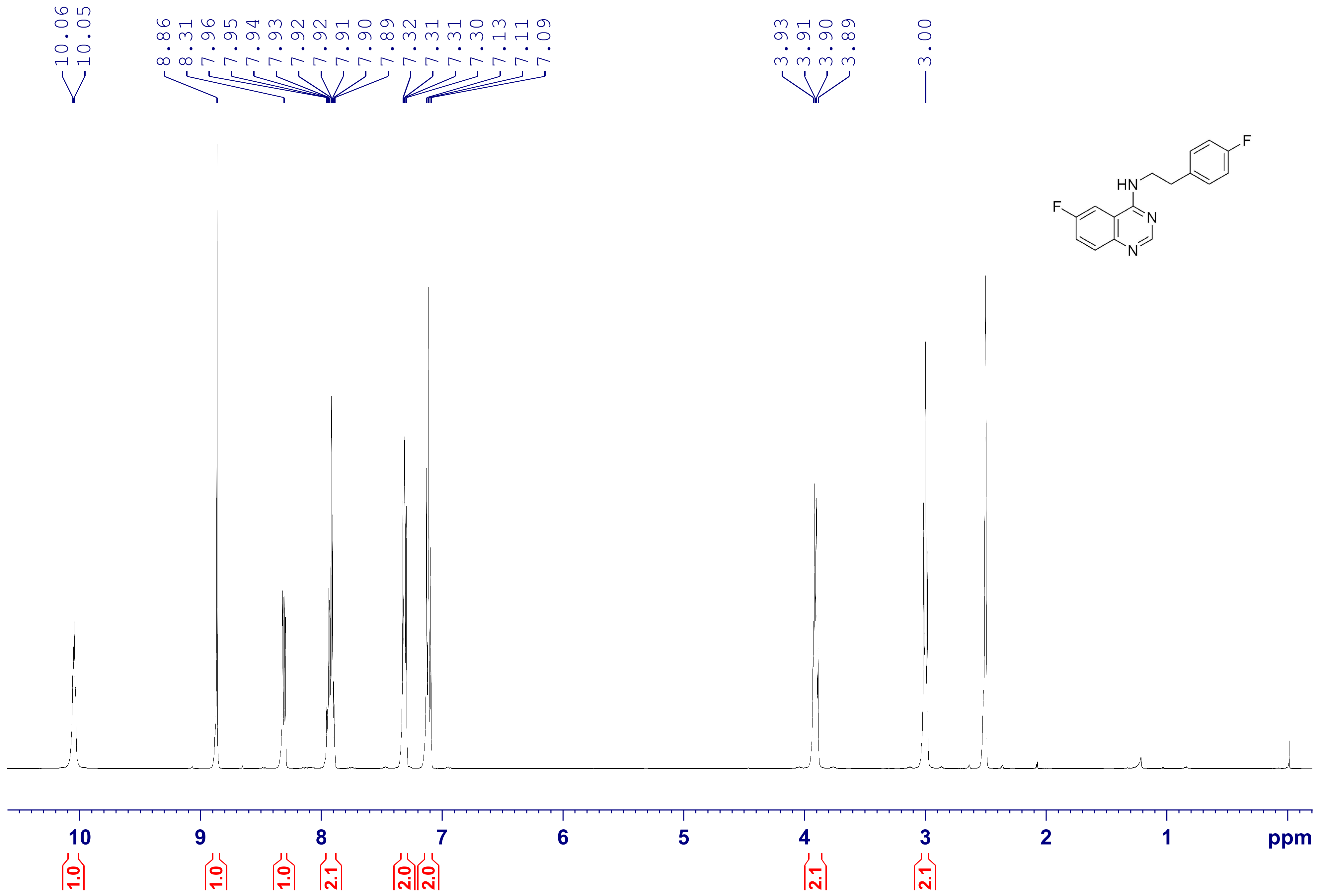
10.06
10.05
8.86
8.31
7.96
7.95
7.94
7.93
7.92
7.92
7.91
7.90
7.89
7.32
7.31
7.31
7.30
7.13
7.11
7.09
3.93
3.91
3.90
3.89
3.00
HN
N
N
F
F
10
9
8
7
6
5
4
3
2
1
ppm
1.0
1.0
1.0
2.1
2.0
2.0
2.1
2.1

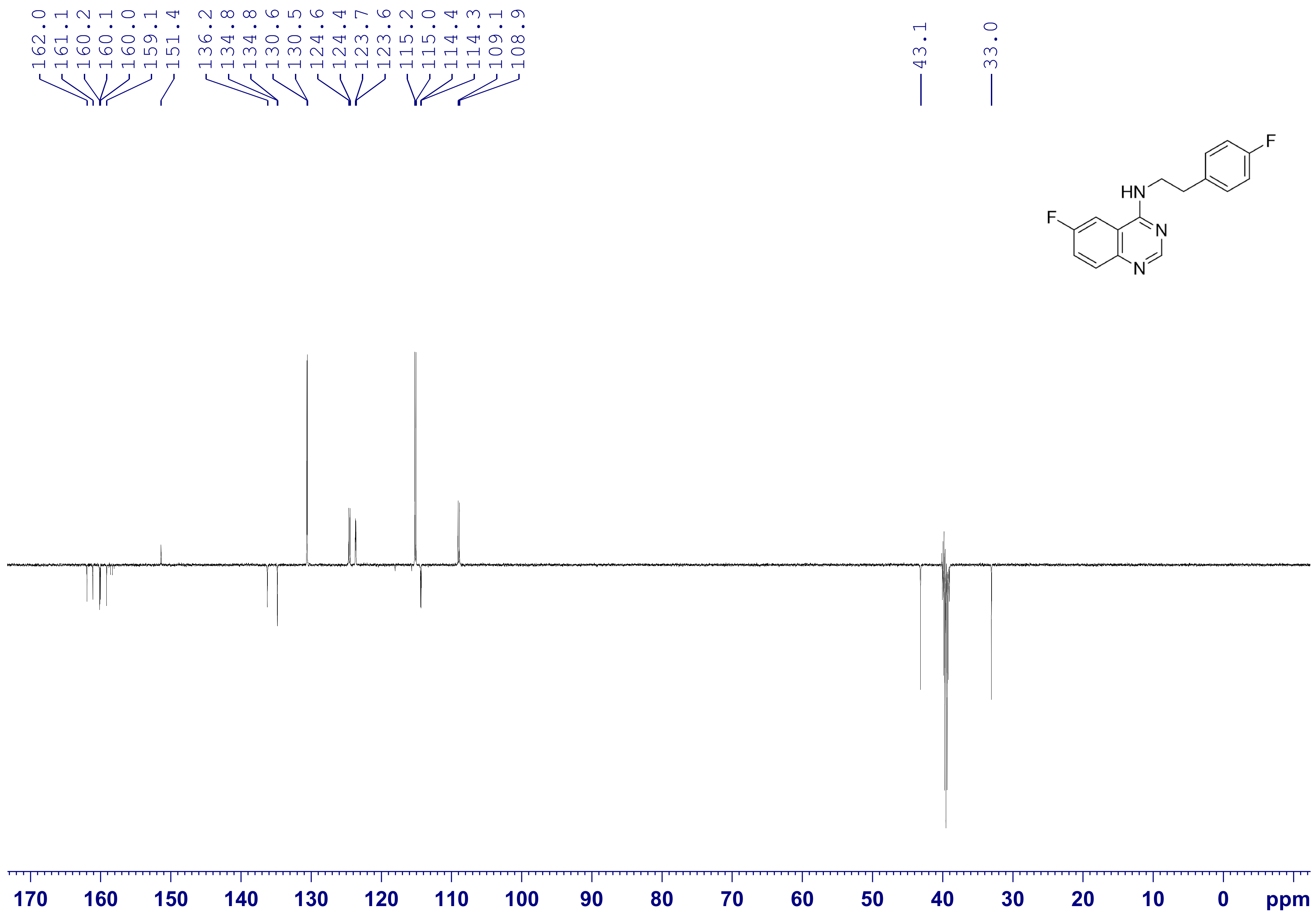
162.0
161.1
160.2
160.1
160.0
159.1
151.4
136.2
134.8
134.8
130.6
130.5
124.6
124.4
123.7
123.6
115.2
115.0
114.4
114.3
109.1
108.9
43.1
33.0
F
HN
F
N
N
170 160 150 140 130 120 110 100 90 80 70 60 50 40 30 20 10 0 ppm

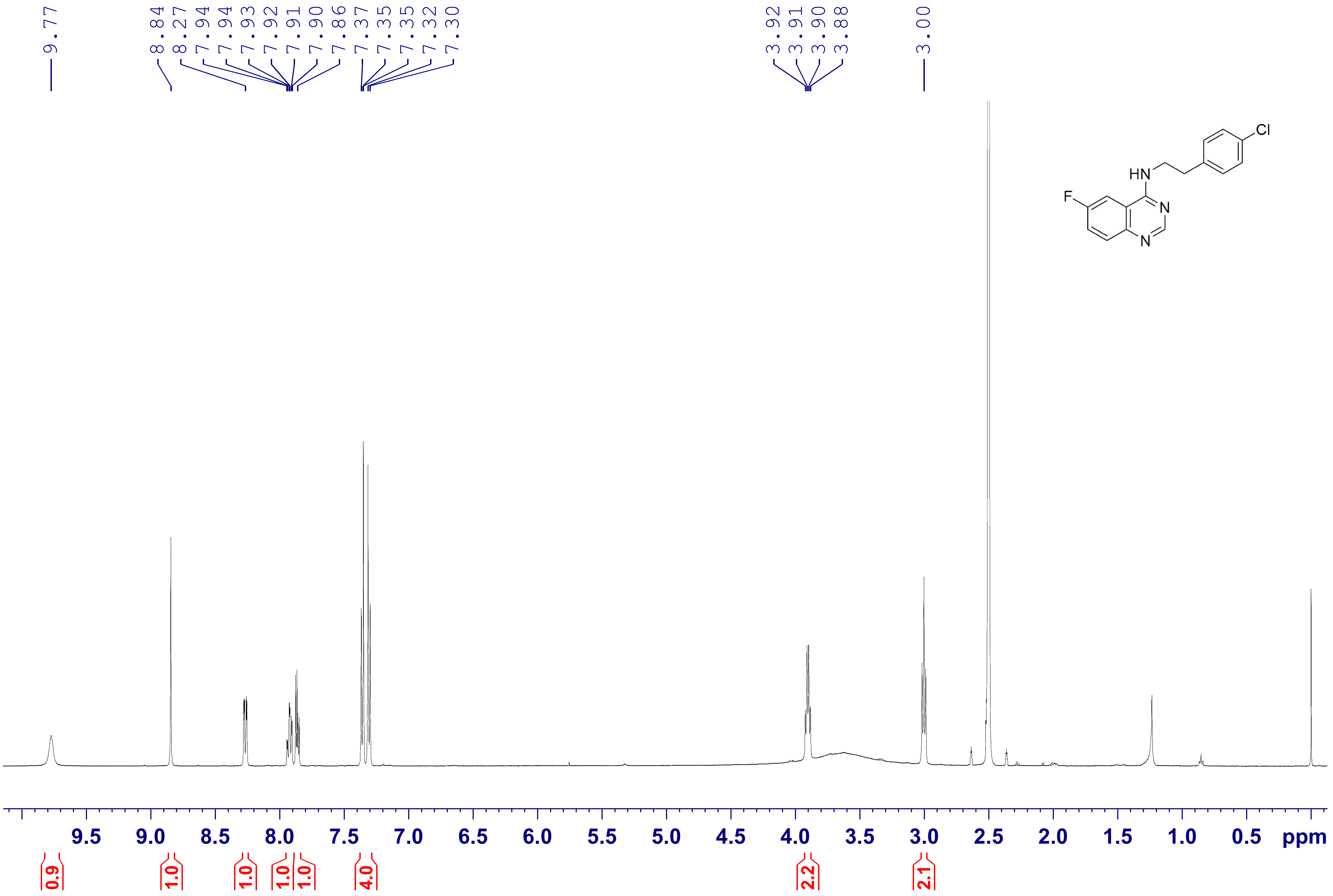
9.77
8.84
8.27
7.94
7.94
7.93
7.92
7.91
7.90
7.86
7.37
7.35
7.35
7.32
7.30
3.92
3.91
3.90
3.88
3.00
HN
N
N
F
Cl
9.5
9.0
8.5
8.0
7.5
7.0
6.5
6.0
5.5
5.0
4.5
4.0
3.5
3.0
2.5
2.0
1.5
1.0
0.5
ppm
0.9
1.0
1.0
1.0
1.0
4.0
2.2
2.1

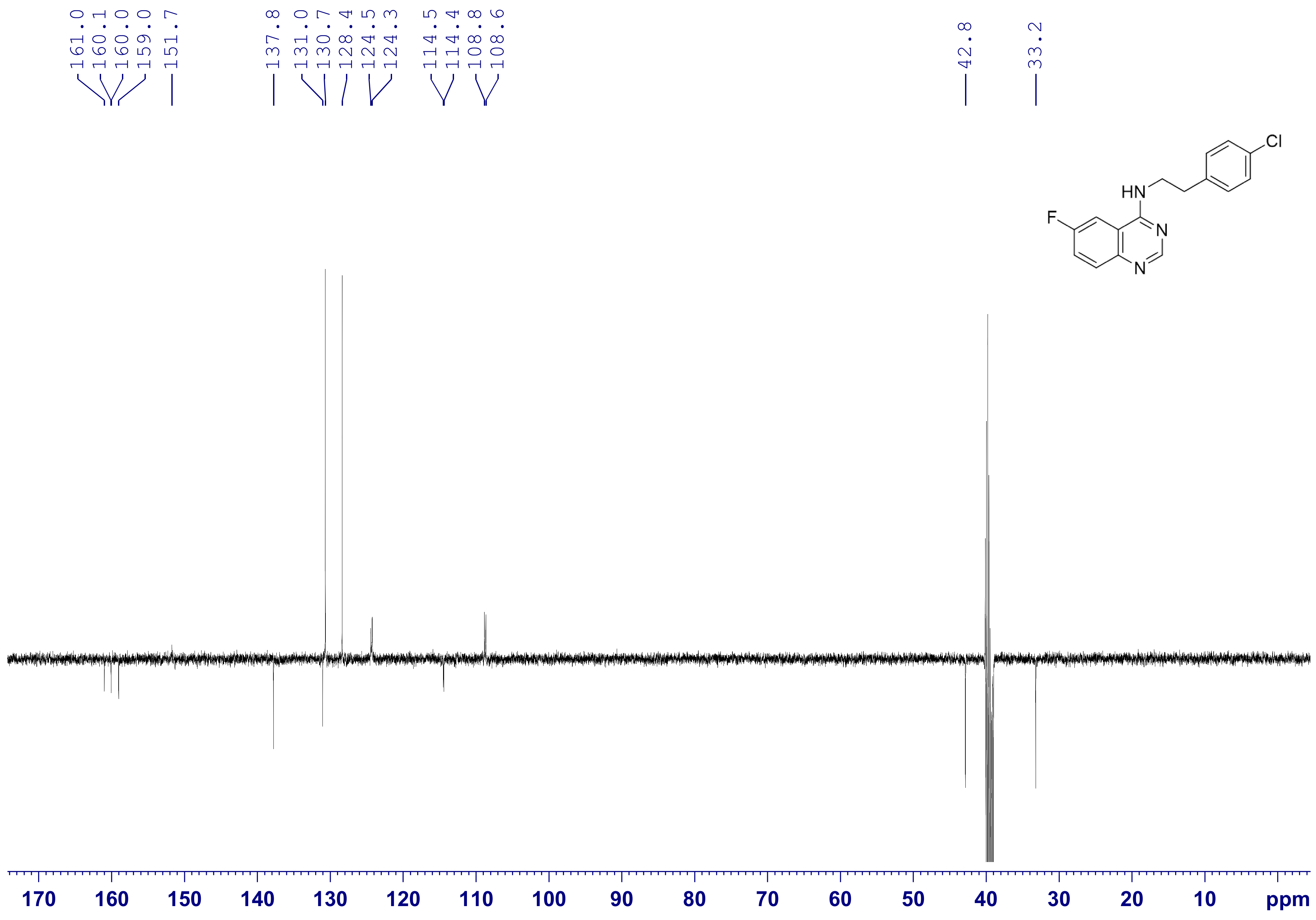
161.0
160.1
160.0
159.0
151.7
137.8
131.0
130.7
128.4
124.5
124.3
114.5
114.4
108.8
108.6
42.8
33.2
F
HN
N
N
Cl
170
160
150
140
130
120
110
100
90
80
70
60
50
40
30
20
10
ppm

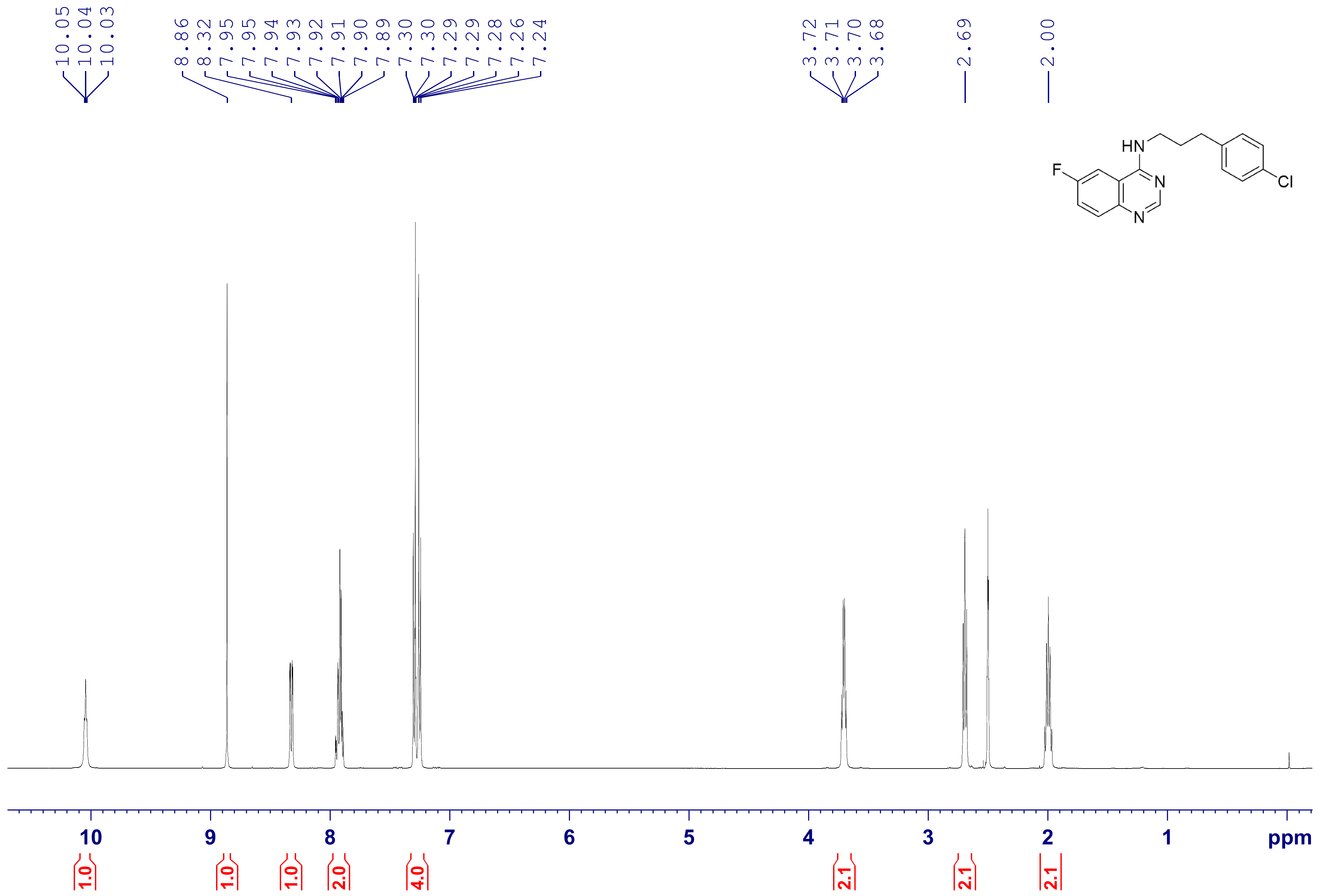
10.05
10.04
10.03
8.86
8.32
7.95
7.95
7.94
7.93
7.92
7.91
7.90
7.89
7.30
7.30
7.29
7.29
7.28
7.26
7.24
3.72
3.71
3.70
3.68
2.69
2.00
HN
F
N
N
Cl
10
9
8
7
6
5
4
3
2
1
ppm
1.0
1.0
1.0
2.0
4.0
2.1
2.1
2.1

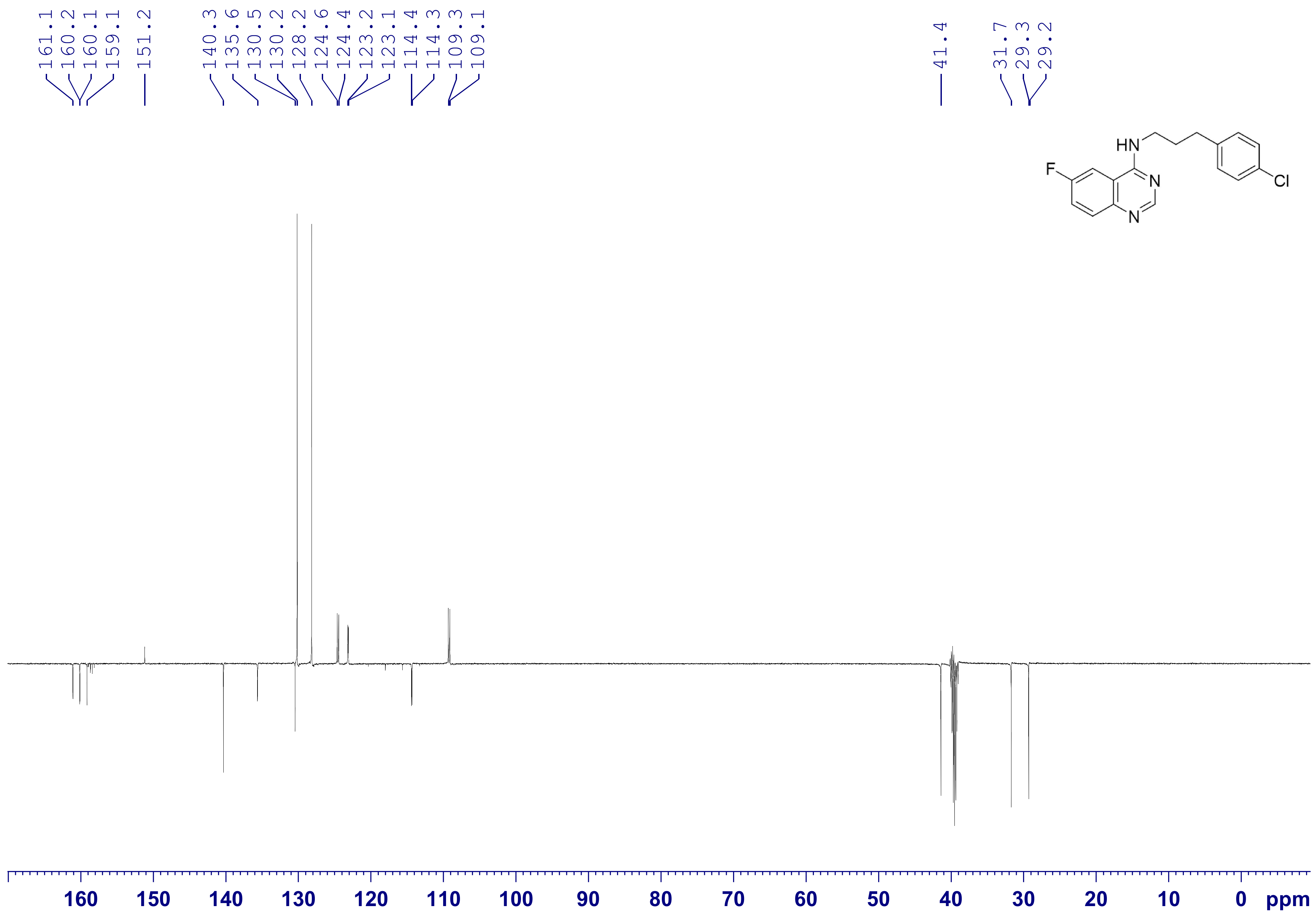
161.1
160.2
160.1
159.1
151.2
140.3
135.6
130.5
130.2
128.2
124.6
124.4
123.2
123.1
114.4
114.3
109.3
109.1
41.4
31.7
29.3
29.2
HN
F
N
N
Cl
160
150
140
130
120
110
100
90
80
70
60
50
40
30
20
10
0
ppm

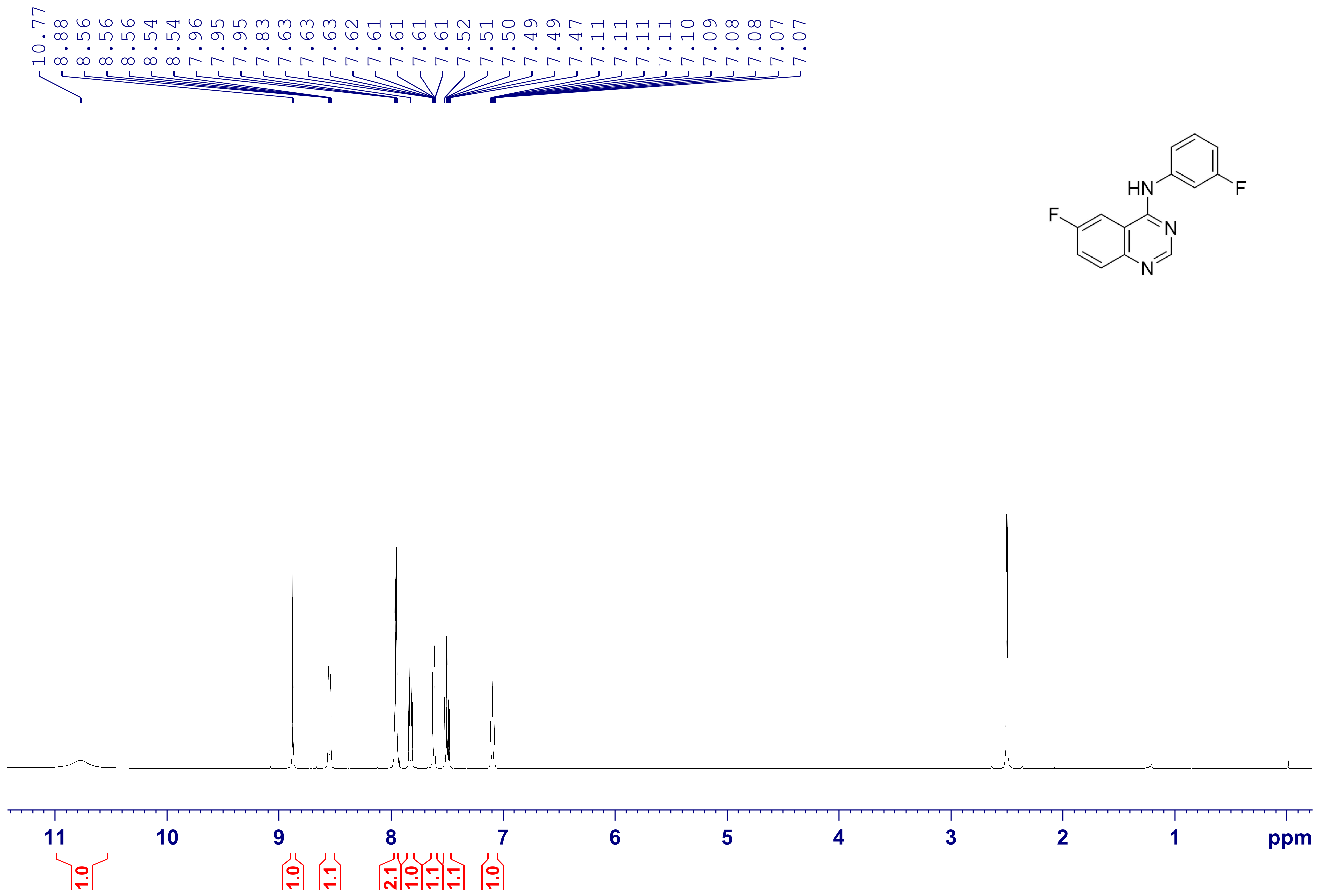
10.77
8.88
8.56
8.56
8.56
8.54
8.54
7.96
7.95
7.95
7.83
7.63
7.63
7.63
7.62
7.61
7.61
7.61
7.61
7.52
7.51
7.50
7.49
7.49
7.47
7.11
7.11
7.11
7.11
7.10
7.09
7.08
7.08
7.07
7.07
HN
F
F
N
N
11
10
9
8
7
6
5
4
3
2
1
ppm
1.0
1.0
1.1
2.1
1.0
1.1
1.1
1.0

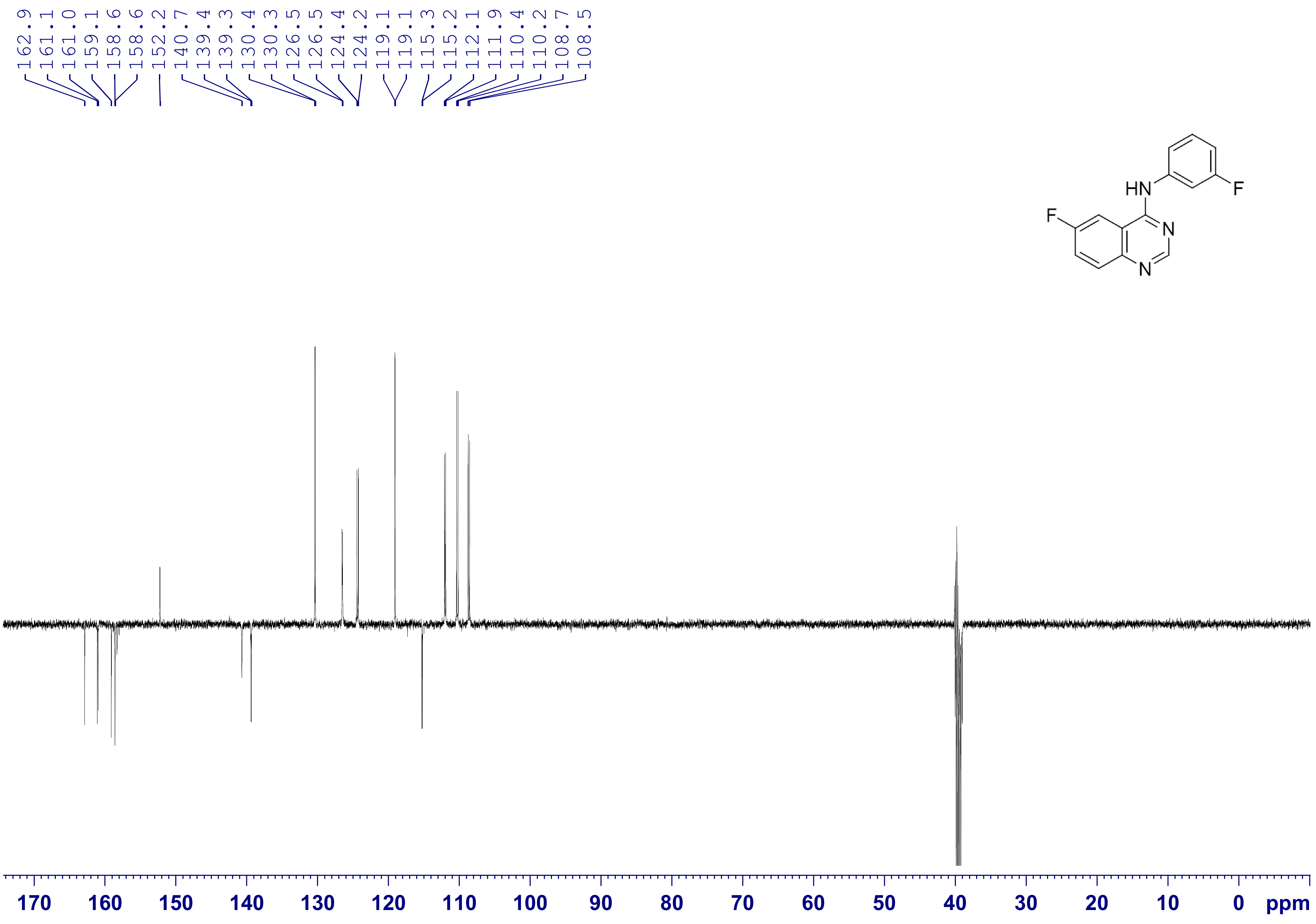

162.9
161.1
161.0
159.1
158.6
158.6
152.2
140.7
139.4
139.3
130.4
130.3
126.5
126.5
124.4
124.2
119.1
119.1
115.3
115.2
112.1
111.9
110.4
110.2
108.7
108.5
HN
F
F
N
N
170 160 150 140 130 120 110 100 90 80 70 60 50 40 30 20 10 0 ppm

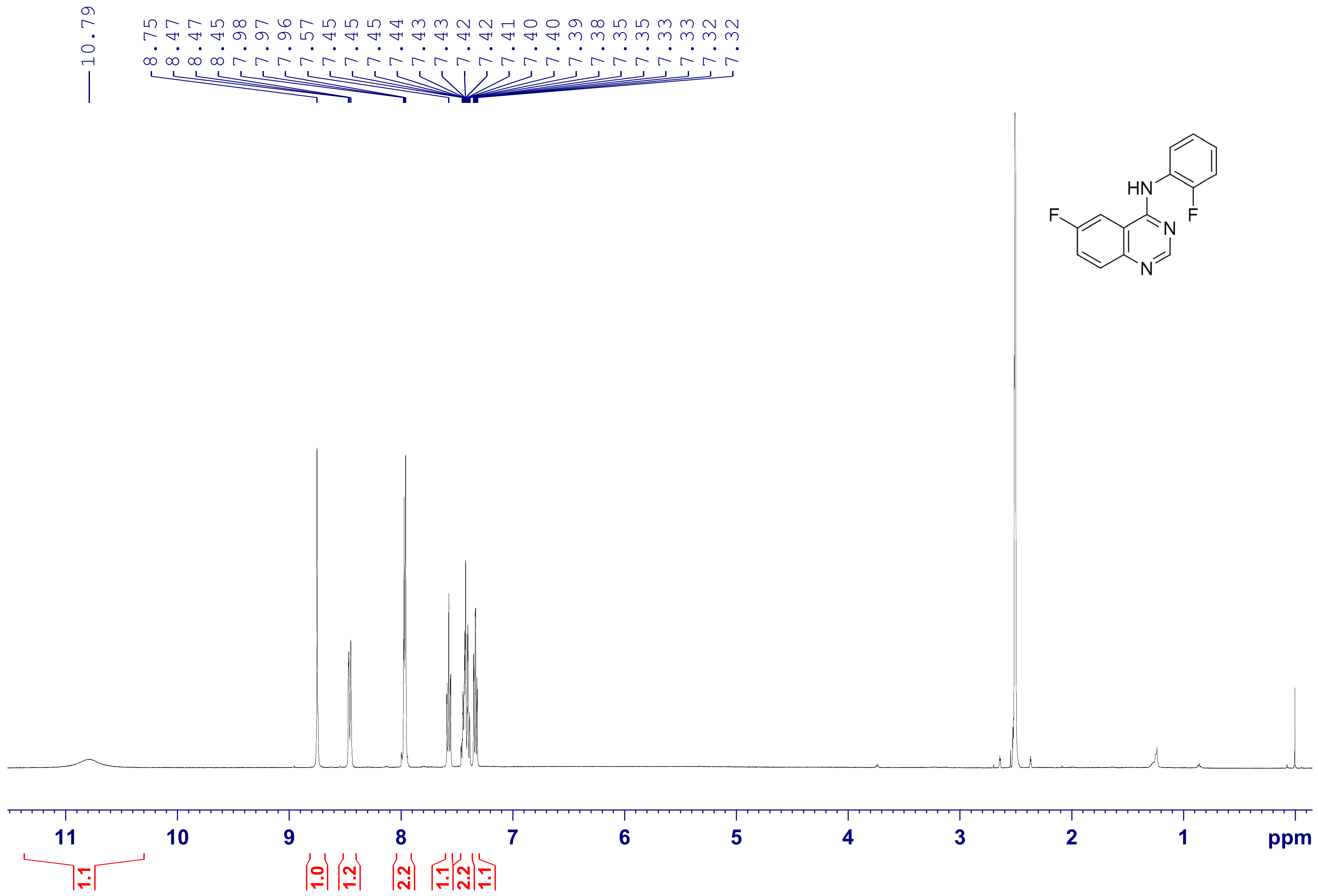

10.79
8.75
8.47
8.47
8.45
7.98
7.97
7.96
7.57
7.45
7.45
7.45
7.44
7.43
7.43
7.42
7.42
7.41
7.40
7.40
7.39
7.38
7.35
7.35
7.33
7.33
7.32
7.32
HN
F
F
N
N
11
10
9
8
7
6
5
4
3
2
1
ppm
1.1
1.0
1.2
2.2
1.1
2.2
1.1

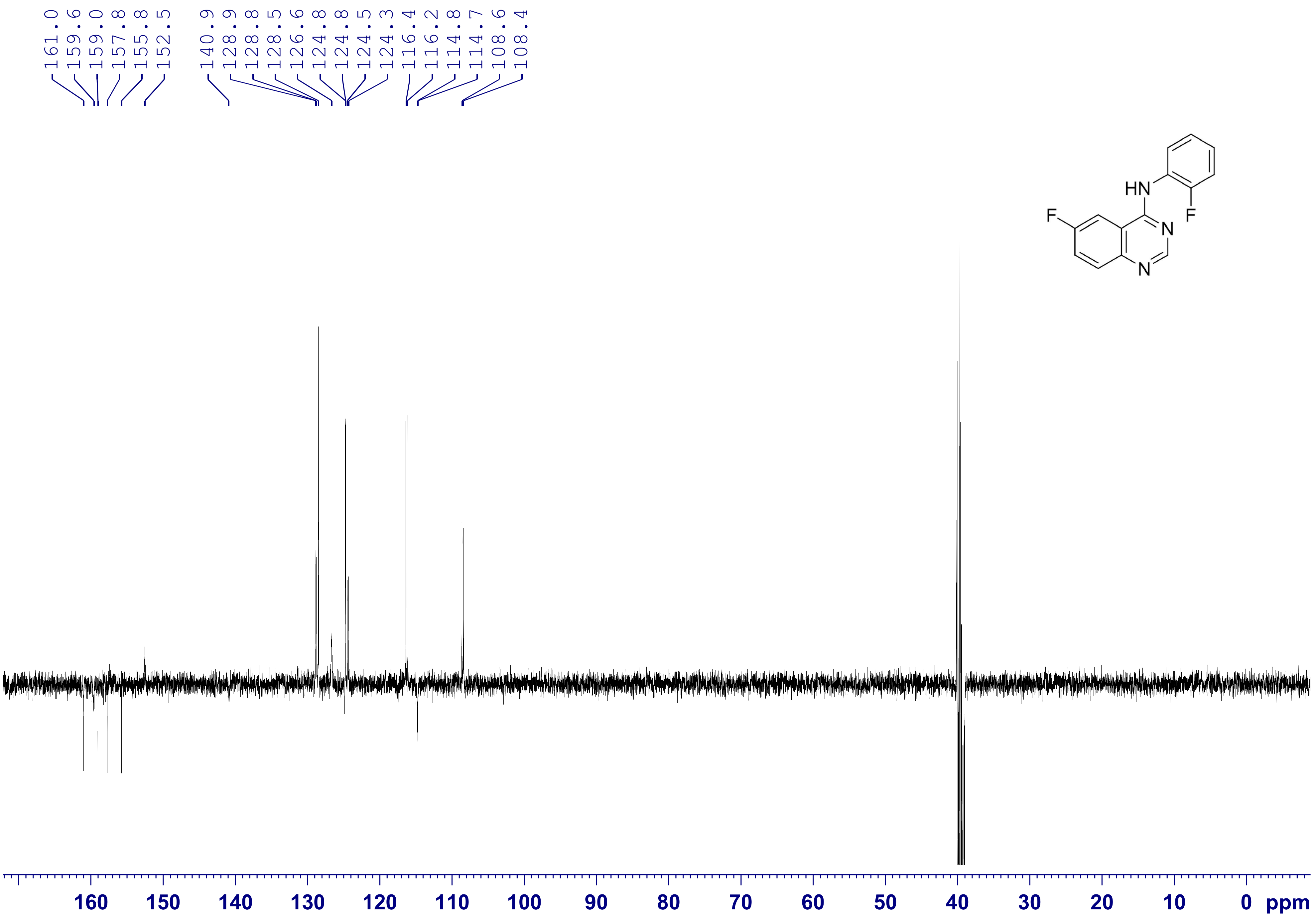
161.0
159.6
159.0
157.8
155.8
152.5
140.9
128.9
128.8
128.5
126.6
124.8
124.8
124.5
124.3
116.4
116.2
114.8
114.7
108.6
108.4
HN
F
N
N
160
150
140
130
120
110
100
90
80
70
60
50
40
30
20
10
0
ppm

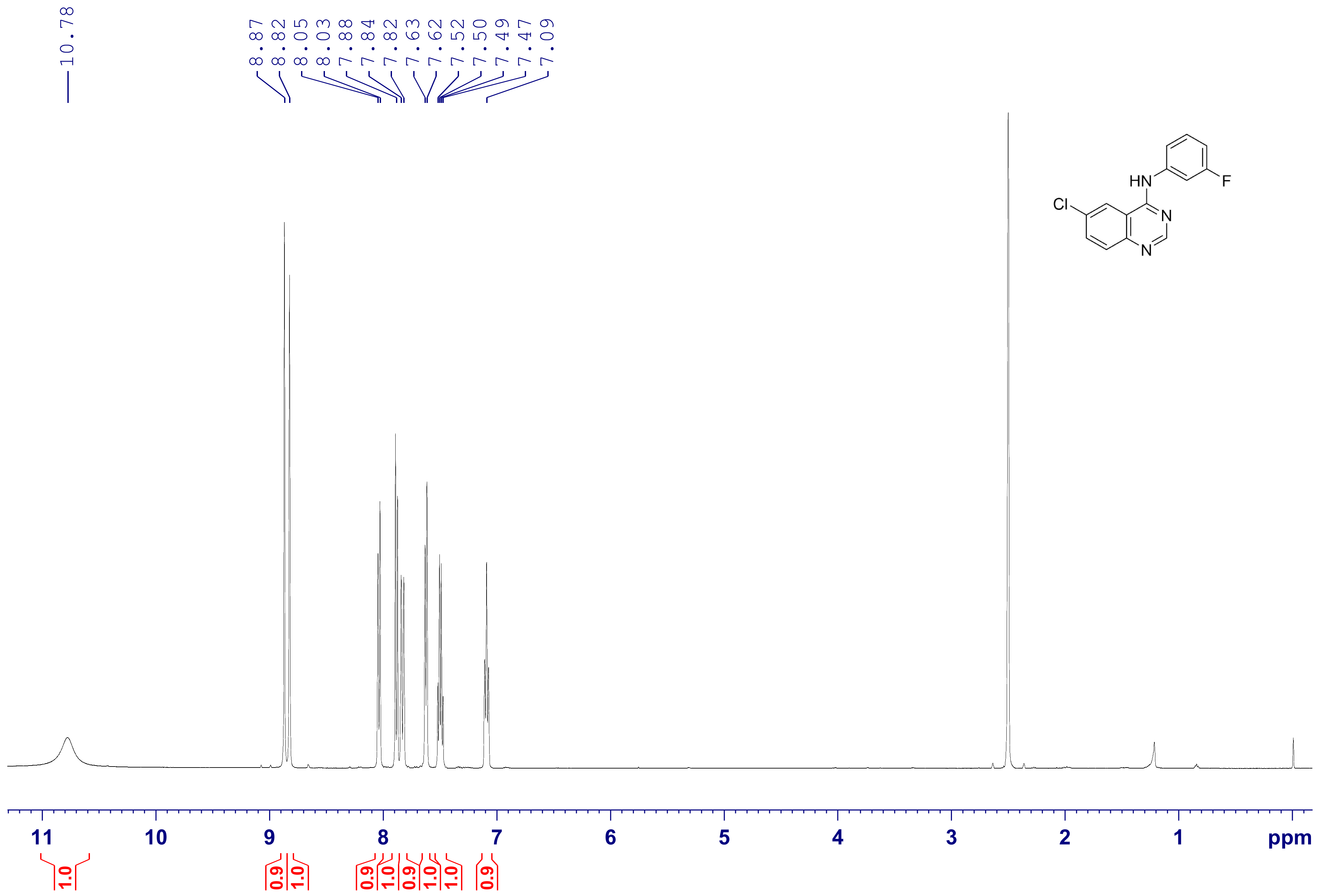

10.78
8.87
8.82
8.05
8.03
7.88
7.84
7.82
7.63
7.62
7.52
7.50
7.49
7.47
7.09
HN
F
Cl
N
N
11
10
9
8
7
6
5
4
3
2
1
ppm
1.0
0.9
1.0
0.9
1.0
0.9
1.0
1.0
0.9

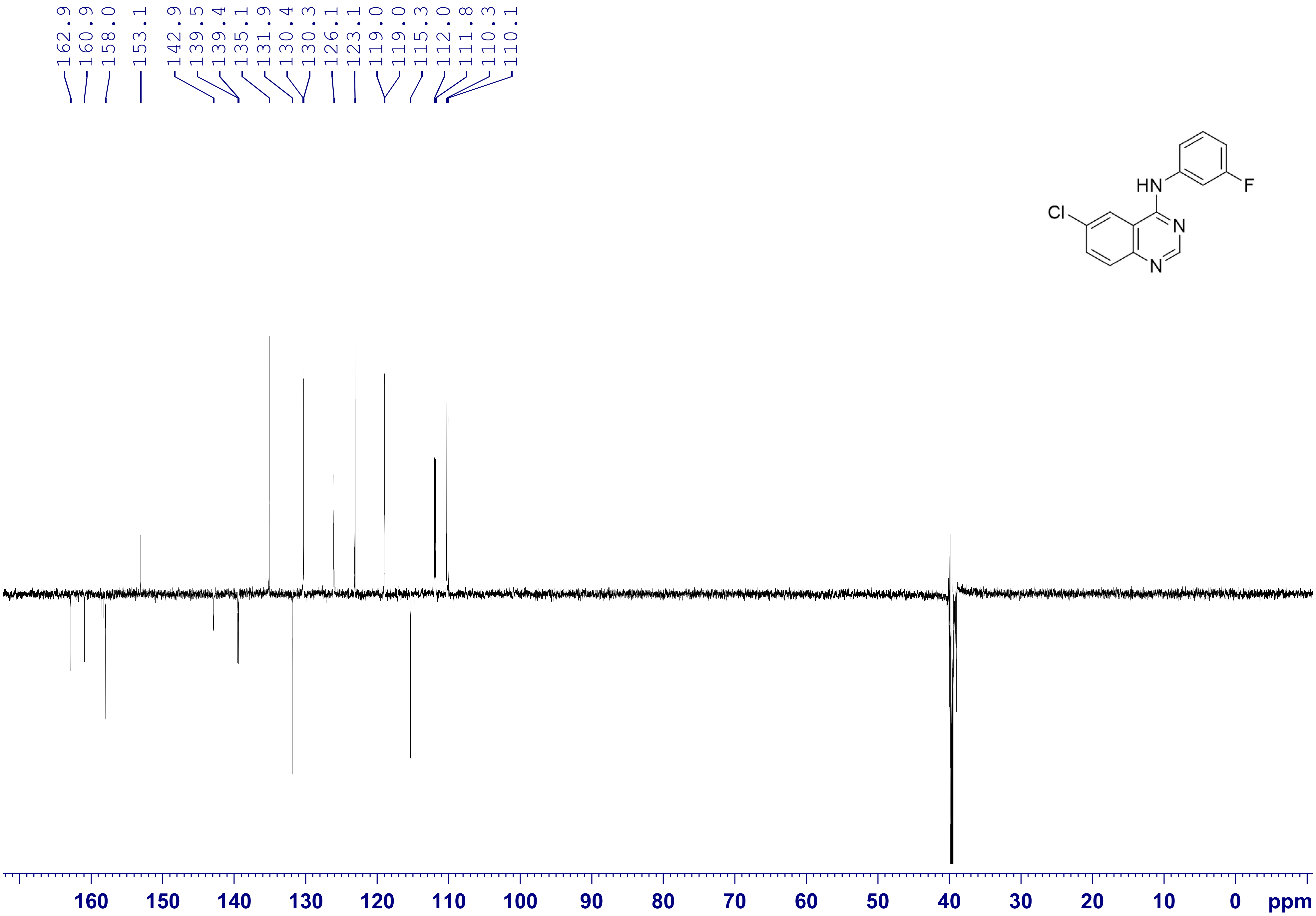
162.9
160.9
158.0
153.1
142.9
139.5
139.4
135.1
131.9
130.4
130.3
126.1
123.1
119.0
119.0
115.3
112.0
111.8
110.3
110.1
HN
F
Cl
N
N
160
150
140
130
120
110
100
90
80
70
60
50
40
30
20
10
0
ppm

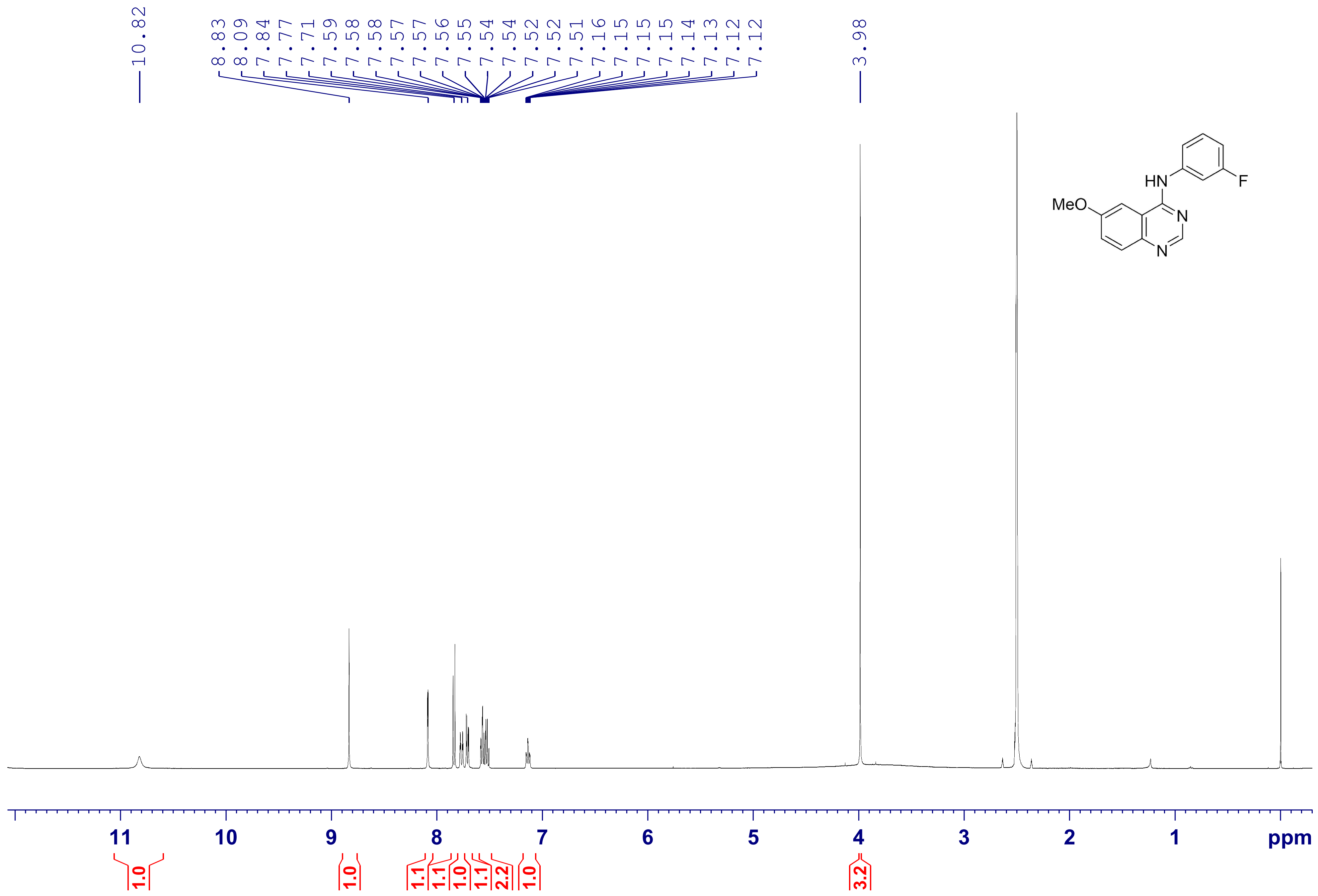

10.82
8.83
8.09
7.84
7.77
7.71
7.59
7.58
7.58
7.57
7.57
7.56
7.55
7.54
7.54
7.52
7.52
7.51
7.16
7.15
7.15
7.15
7.14
7.13
7.12
7.12
3.98
MeO
HN
N
N
F
11
10
9
8
7
6
5
4
3
2
1
ppm
1.0
1.0
1.1
1.1
1.0
1.1
2.2
1.0
3.2

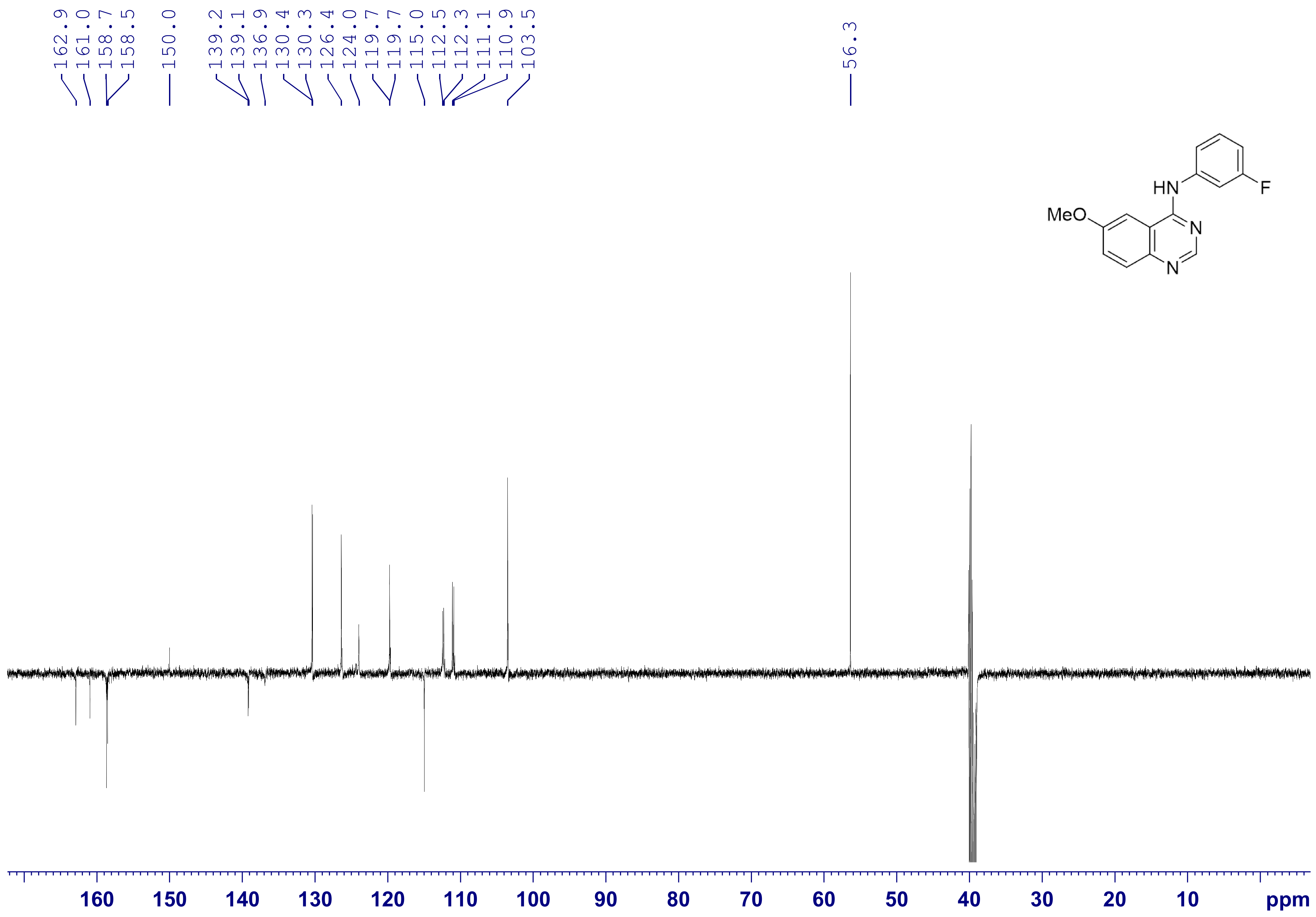
162.9
161.0
158.7
158.5
150.0
139.2
139.1
136.9
130.4
130.3
126.4
124.0
119.7
119.7
115.0
112.5
112.3
111.1
110.9
103.5
56.3
MeO
HN
N
N
F
160
150
140
130
120
110
100
90
80
70
60
50
40
30
20
10
ppm

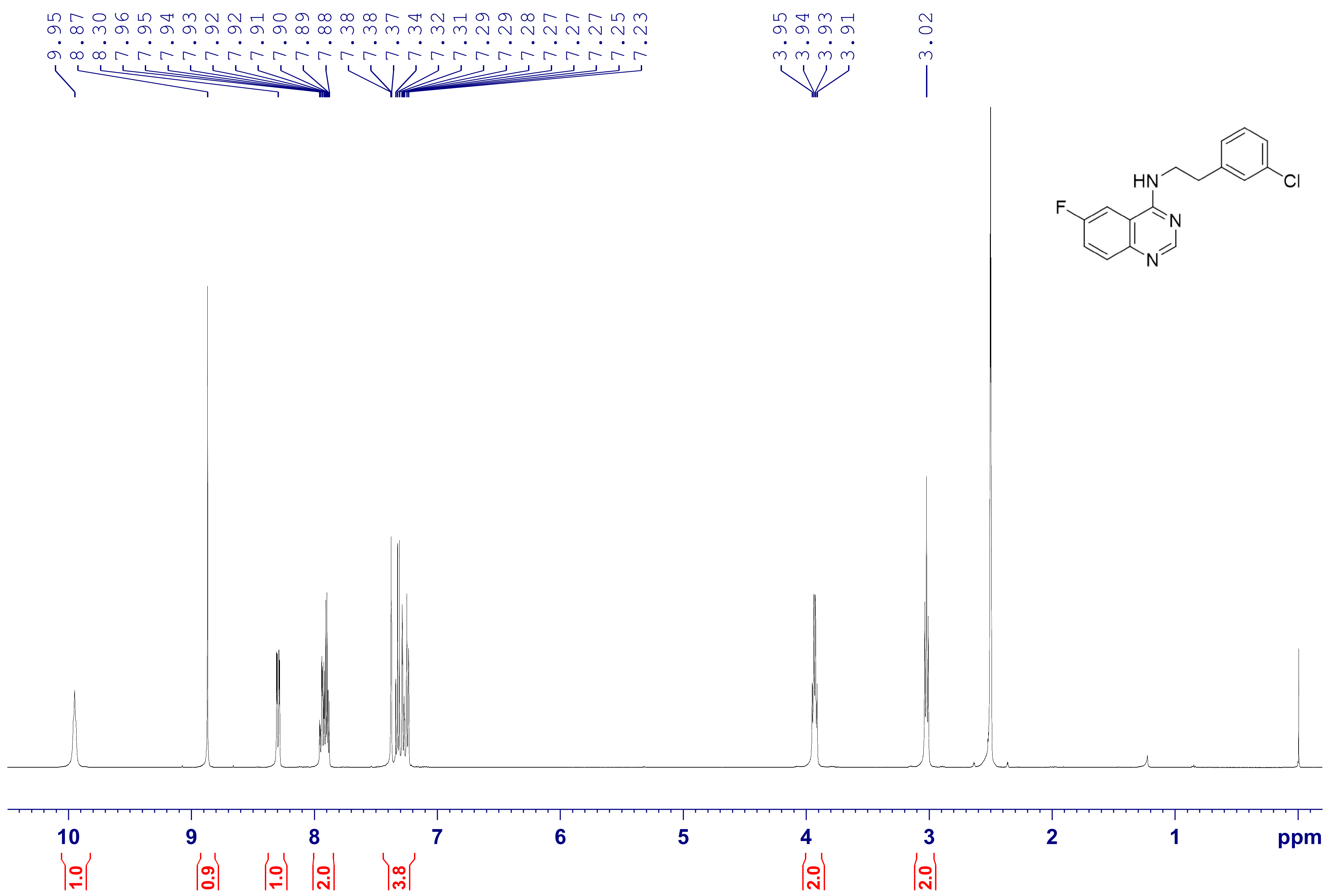
9.95
8.87
8.30
7.96
7.95
7.94
7.93
7.92
7.92
7.91
7.90
7.89
7.88
7.38
7.38
7.37
7.34
7.32
7.31
7.29
7.29
7.28
7.27
7.27
7.27
7.25
7.23
3.95
3.94
3.93
3.91
3.02
HN
F
N
N
Cl
10
9
8
7
6
5
4
3
2
1
ppm
1.0
0.9
1.0
2.0
3.8
2.0
2.0

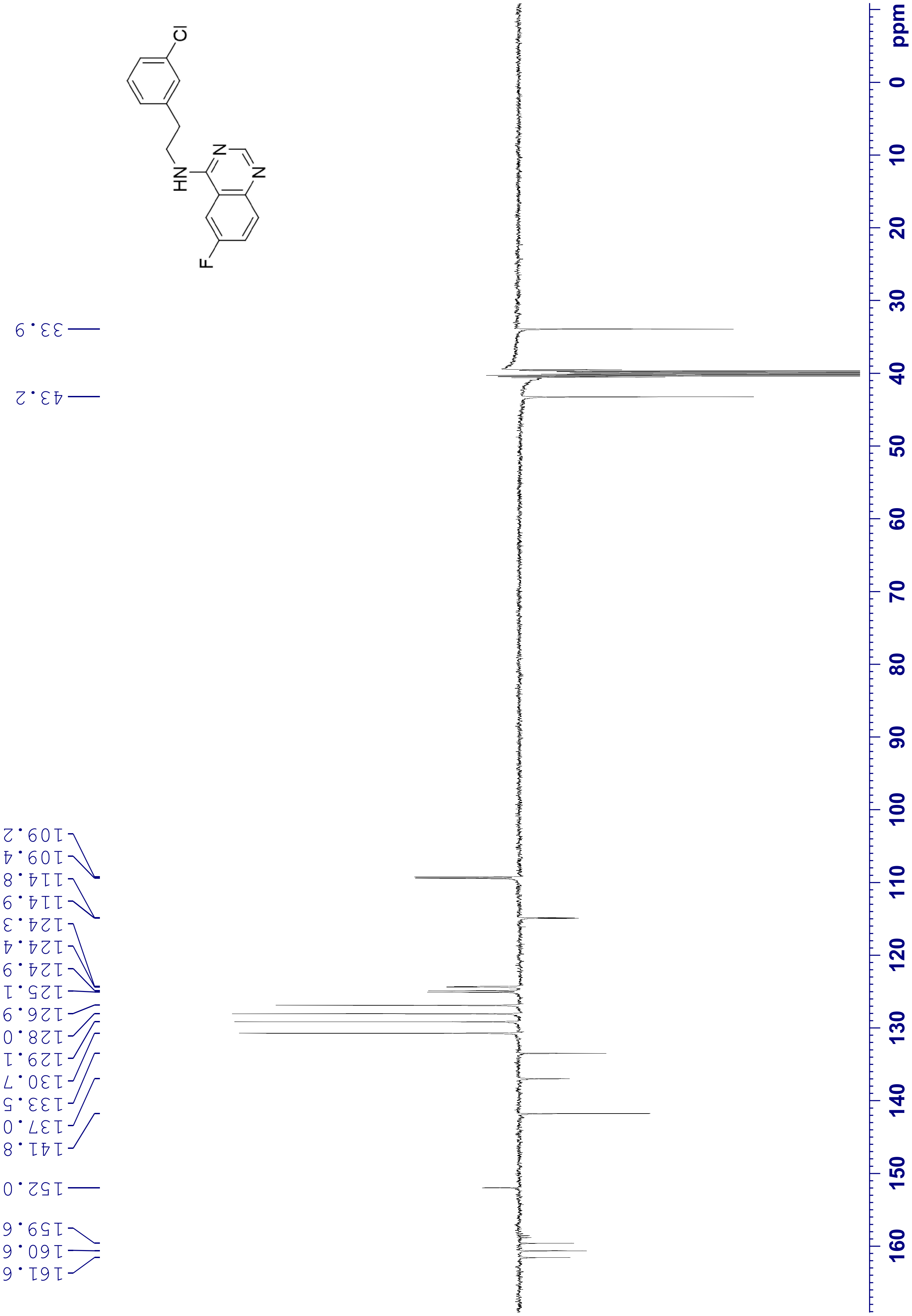

F
HN
N
N
Cl
33.9
43.2
109.2
109.4
114.8
114.9
124.3
124.4
124.9
125.1
126.9
128.0
129.1
130.7
133.5
137.0
141.8
152.0
159.6
160.6
161.6
ppm
0
10
20
30
40
50
60
70
80
90
100
110
120
130
140
150
160

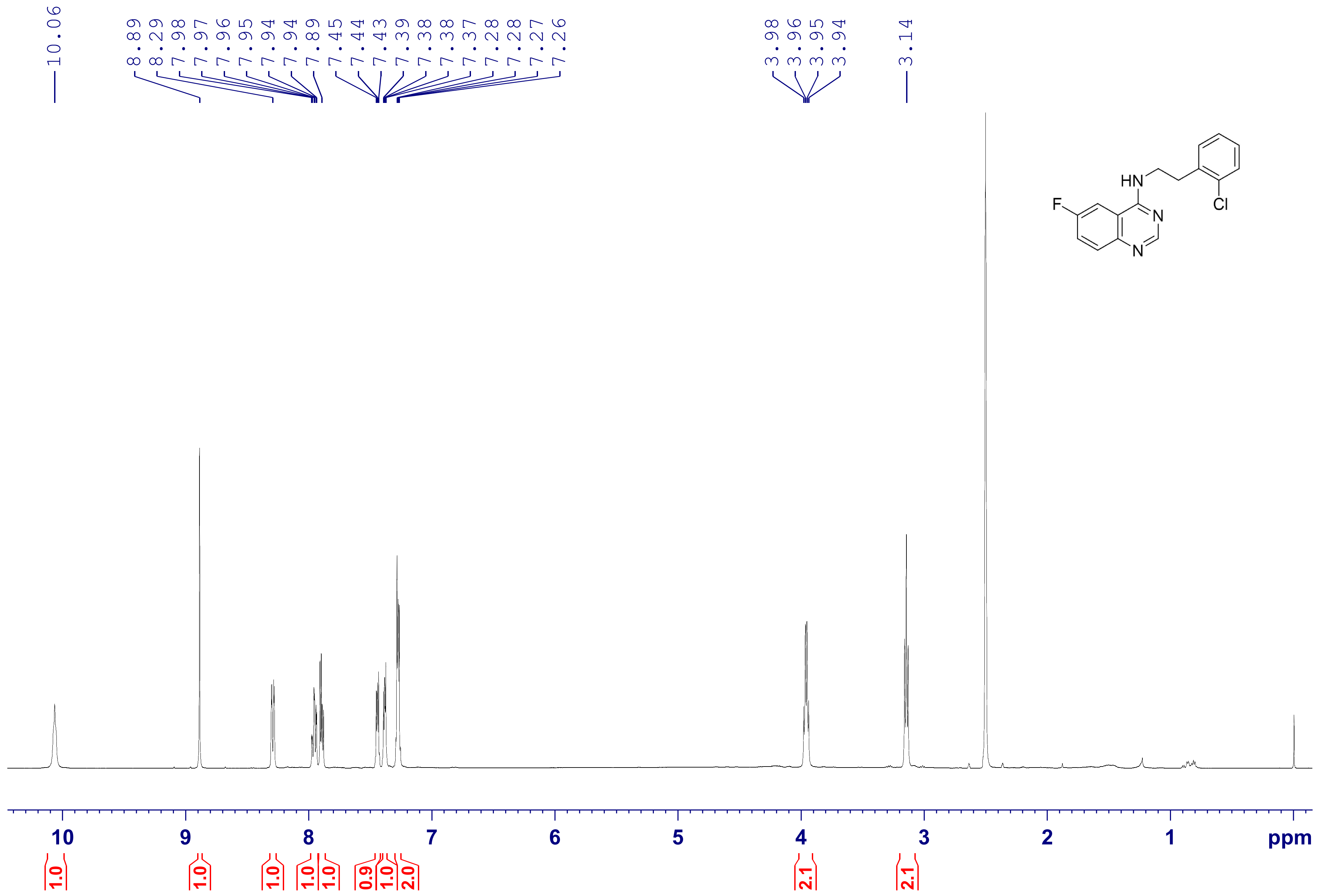

10.06
8.89
8.29
7.98
7.97
7.96
7.95
7.94
7.94
7.89
7.45
7.44
7.43
7.39
7.38
7.38
7.37
7.28
7.28
7.27
7.26
3.98
3.96
3.95
3.94
3.14
HN
F
N
N
Cl
10
9
8
7
6
5
4
3
2
1
ppm
1.0
1.0
1.0
1.0
1.0
0.9
1.0
2.0
2.1
2.1

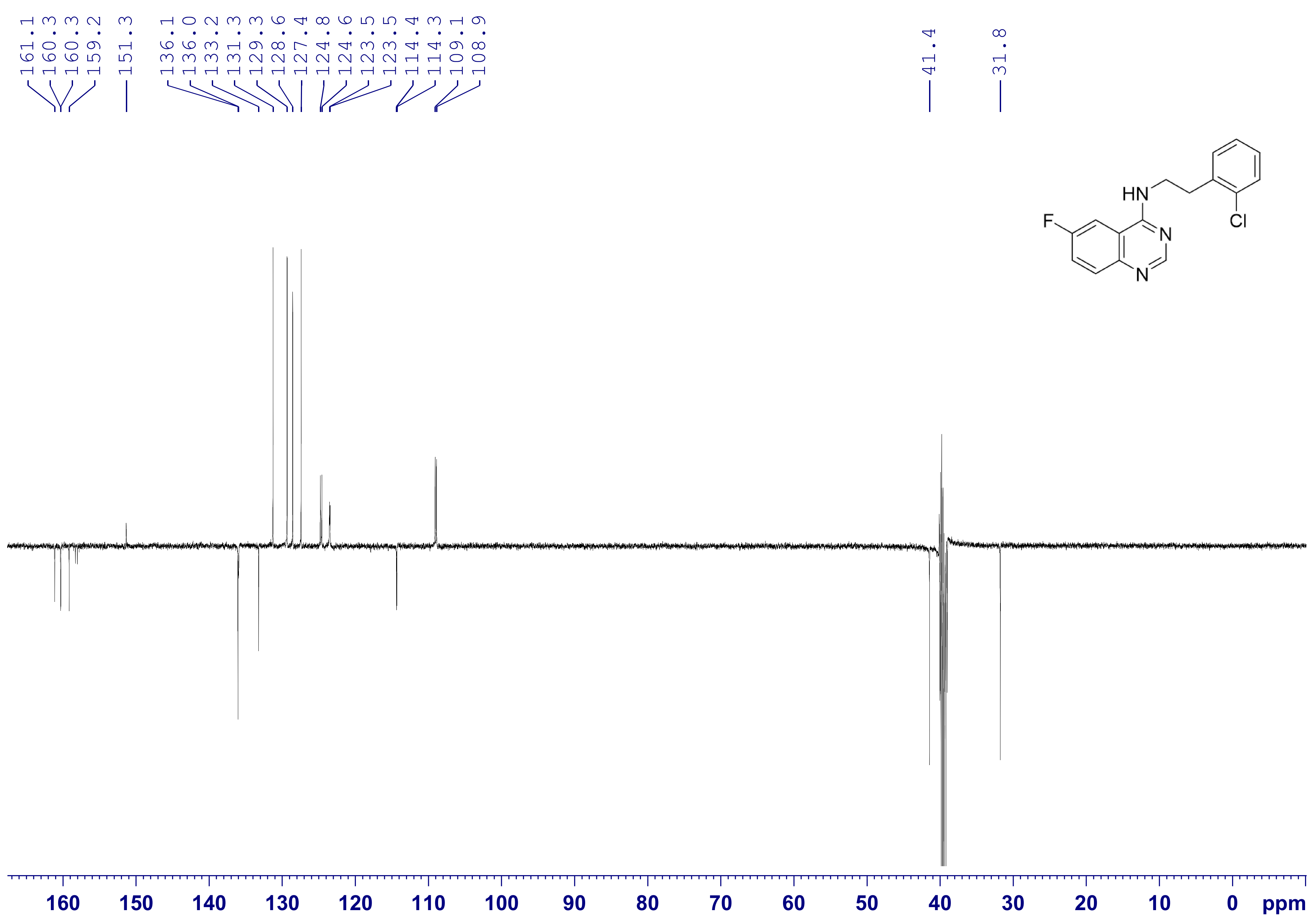
161.1
160.3
160.3
159.2
151.3
136.1
136.0
133.2
131.3
129.3
128.6
127.4
124.8
124.6
123.5
123.5
114.4
114.3
109.1
108.9
41.4
31.8
HN
F
N
N
Cl
160
150
140
130
120
110
100
90
80
70
60
50
40
30
20
10
0
ppm

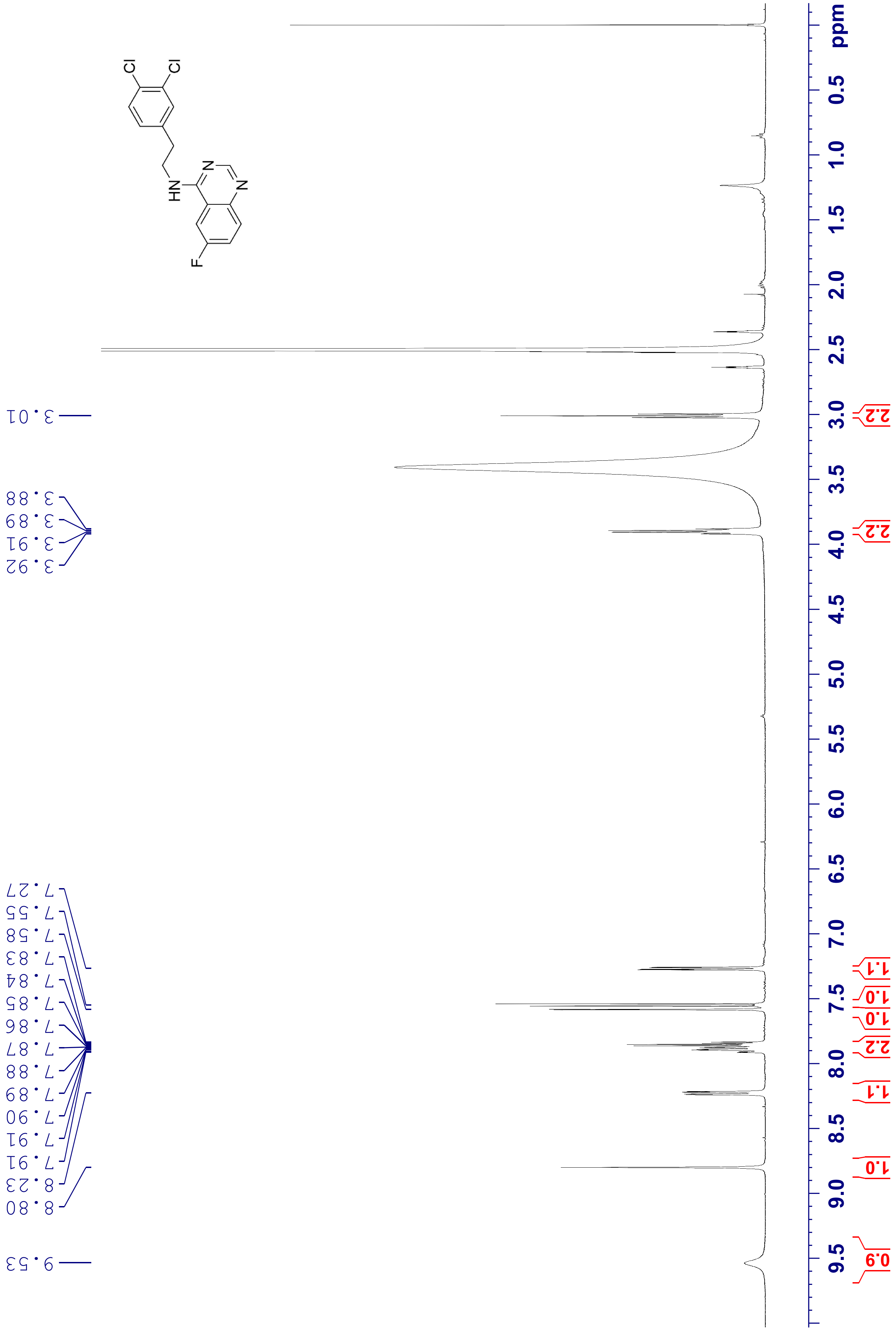
ppm
0.5
1.0
1.5
2.0
2.5
3.0
3.5
4.0
4.5
5.0
5.5
6.0
6.5
7.0
7.5
8.0
8.5
9.0
9.5
2.2
2.2
1.1
1.0
1.0
2.2
1.1
1.0
0.9
3.01
3.88
3.89
3.91
3.92
7.27
7.55
7.58
7.83
7.84
7.85
7.86
7.87
7.88
7.89
7.90
7.91
7.91
8.23
8.80
9.53
Cl
Cl
HN
N
N
F

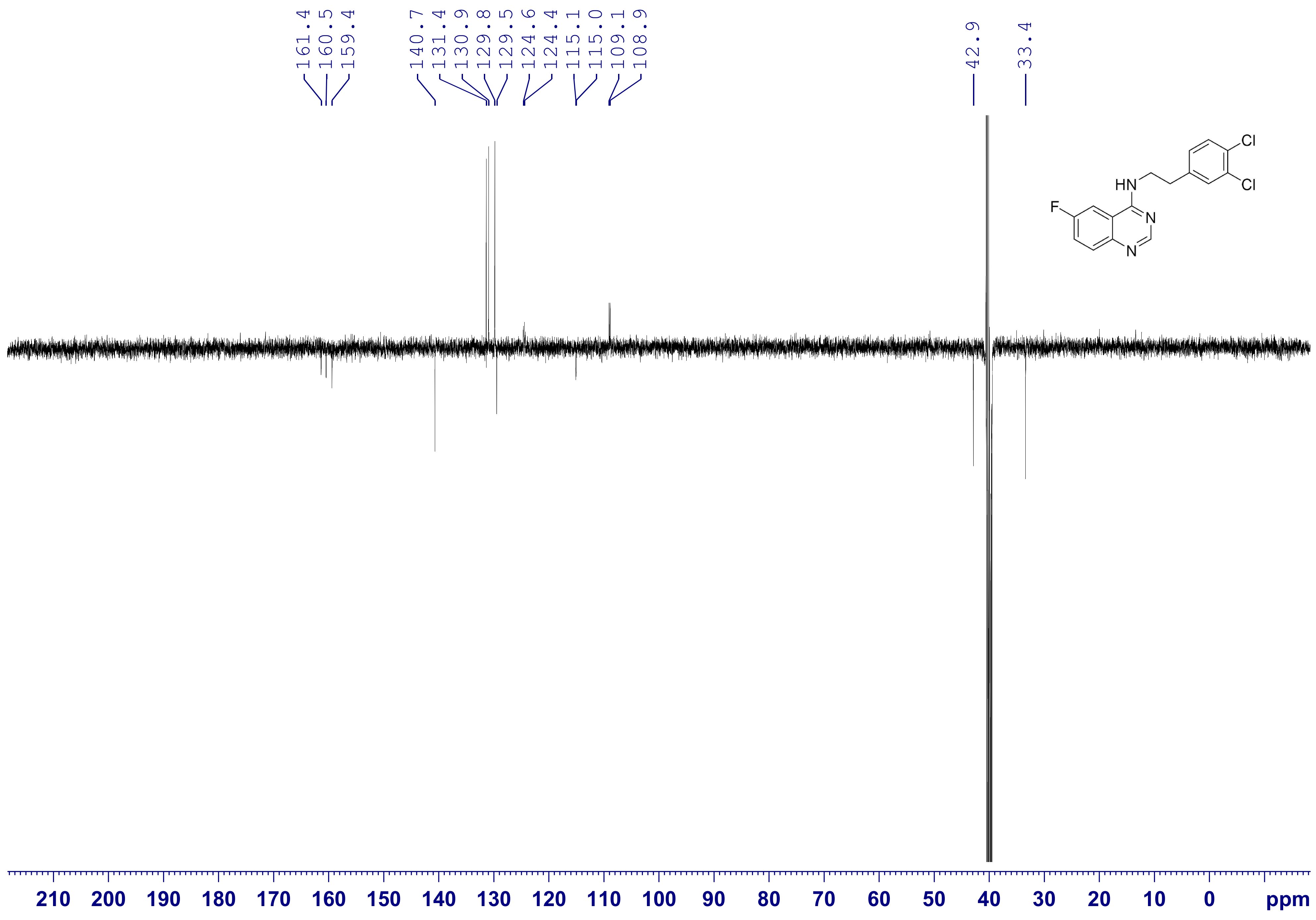
161.4
160.5
159.4
140.7
131.4
130.9
129.8
129.5
124.6
124.4
115.1
115.0
109.1
108.9
42.9
33.4
HN
Cl
Cl
F
N
N
210
200
190
180
170
160
150
140
130
120
110
100
90
80
70
60
50
40
30
20
10
0
ppm

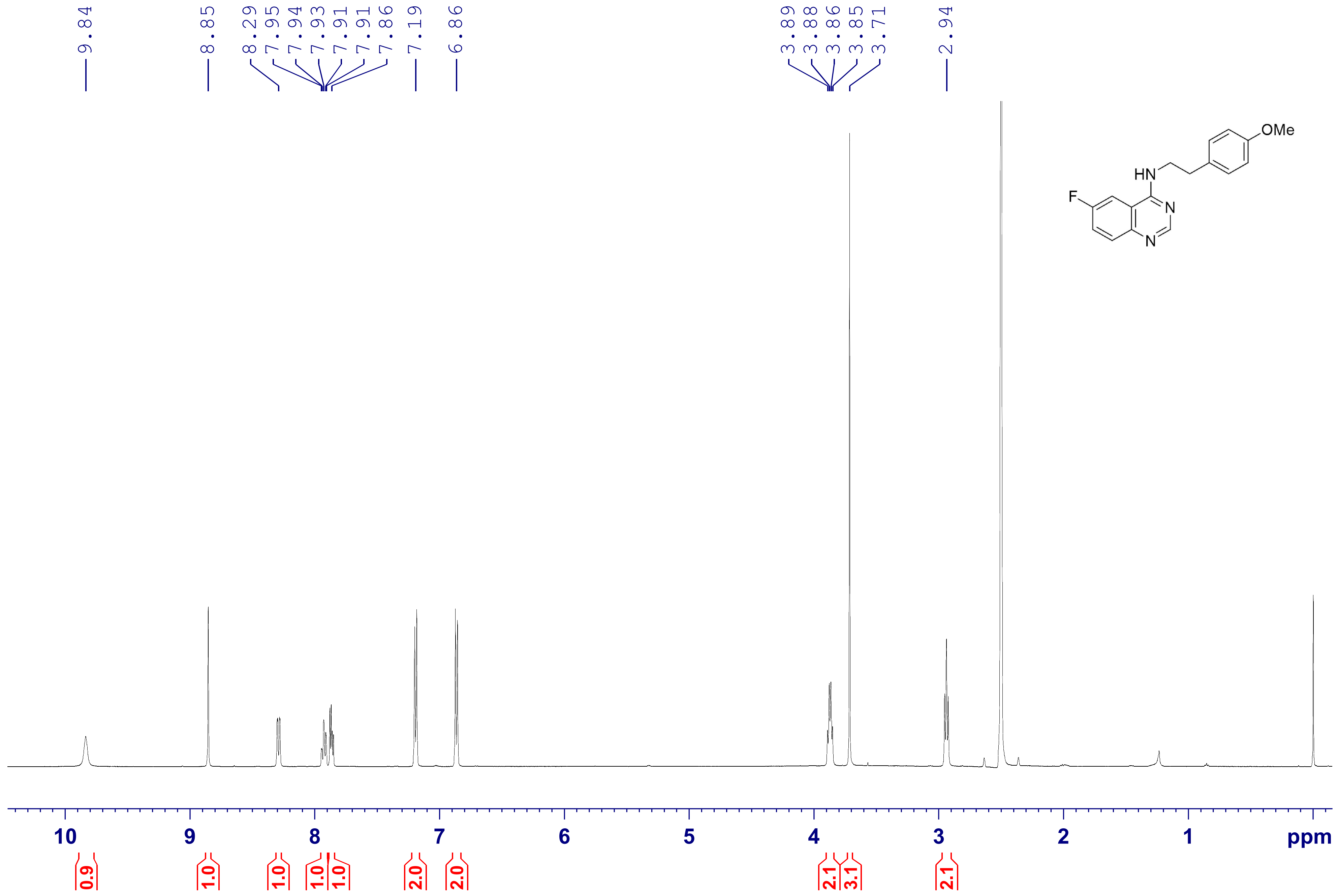
9.84
8.85
8.29
7.95
7.94
7.93
7.91
7.91
7.86
7.19
6.86
3.89
3.88
3.86
3.85
3.71
2.94
OMe
HN
F
N
N
10
9
8
7
6
5
4
3
2
1
ppm
0.9
1.0
1.0
1.0
1.0
2.0
2.0
2.1
3.1
2.1

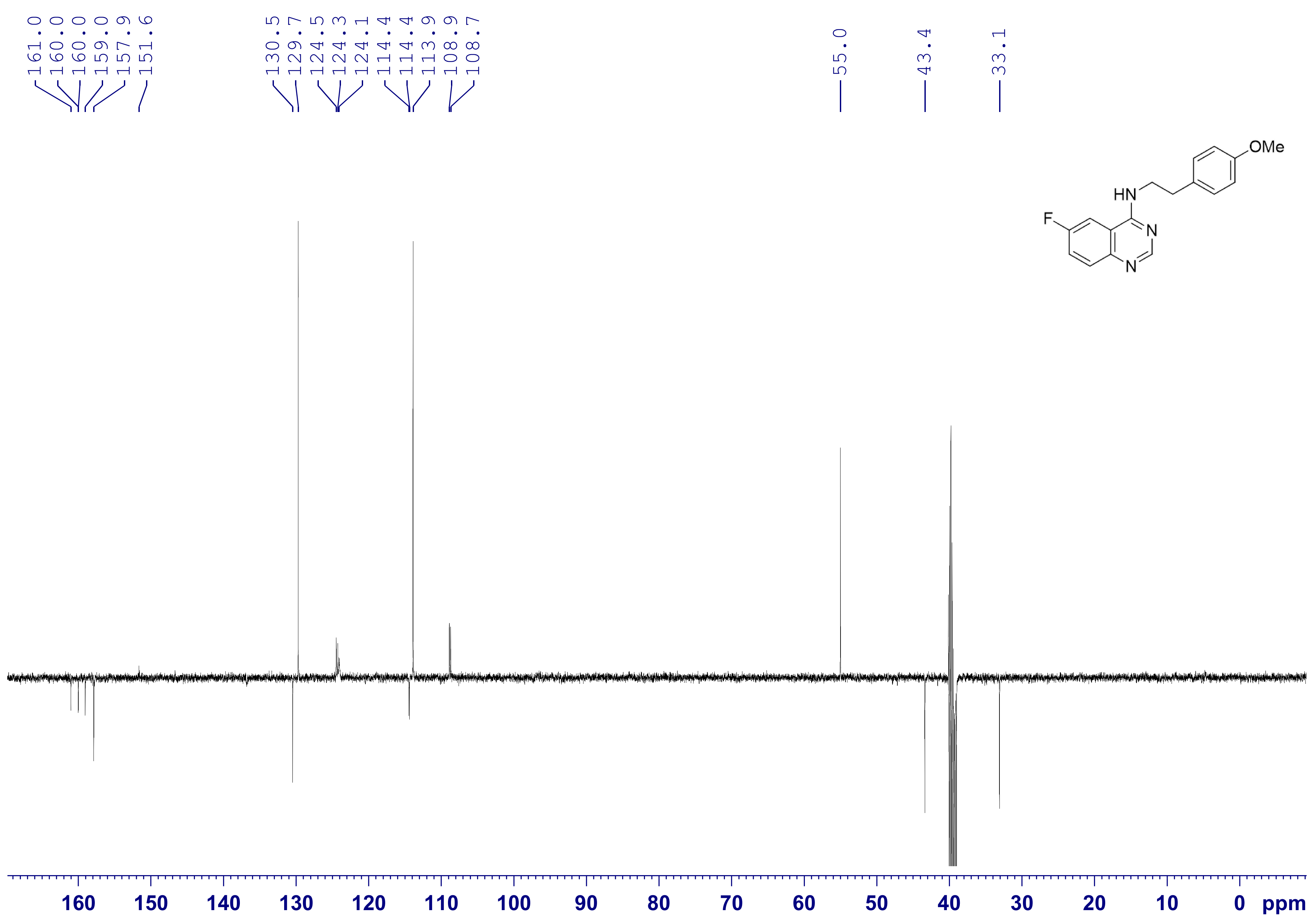

161.0
160.0
160.0
159.0
157.9
151.6
130.5
129.7
124.5
124.3
124.1
114.4
114.4
113.9
108.9
108.7
55.0
43.4
33.1
OMe
HN
F
N
N
160
150
140
130
120
110
100
90
80
70
60
50
40
30
20
10
0
ppm

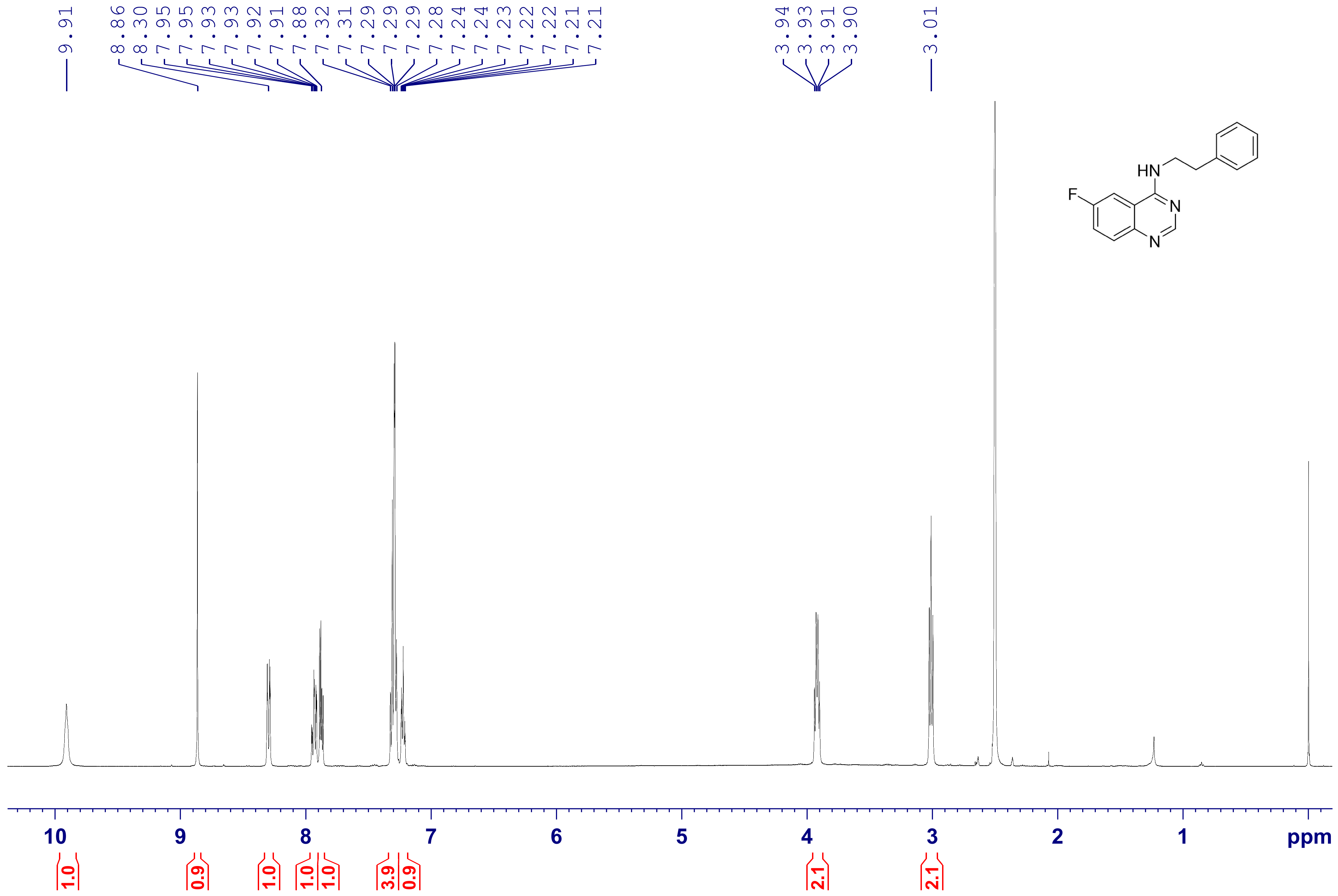
9.91
8.86
8.30
7.95
7.95
7.93
7.93
7.92
7.91
7.88
7.32
7.31
7.29
7.29
7.29
7.28
7.24
7.24
7.23
7.22
7.22
7.21
7.21
3.94
3.93
3.91
3.90
3.01
F
HN
N
N
10
9
8
7
6
5
4
3
2
1
ppm
1.0
0.9
1.0
1.0
1.0
3.9
0.9
2.1
2.1

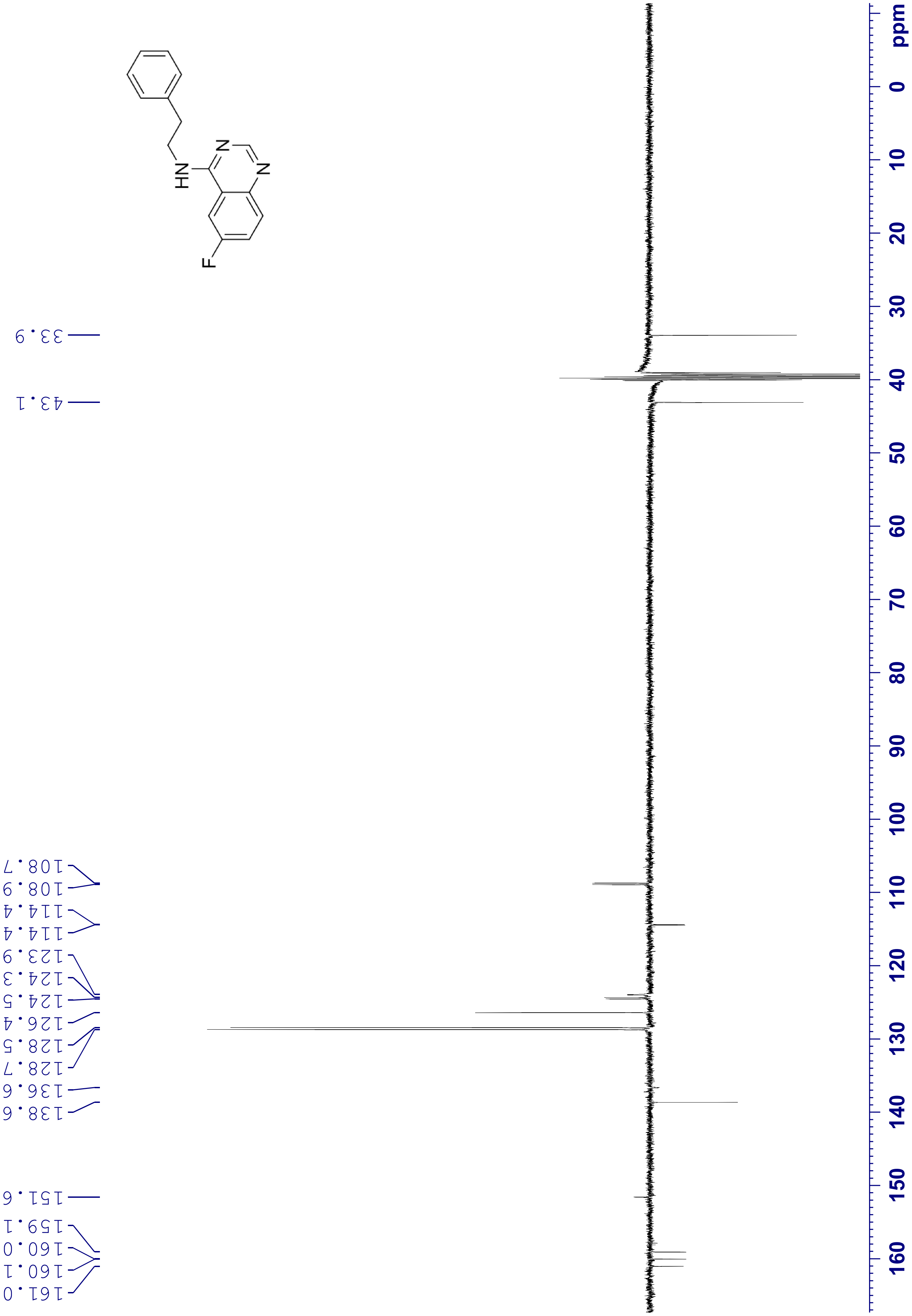

ppm
0
10
20
30
40
50
60
70
80
90
100
110
120
130
140
150
160
33.9
43.1
108.7
108.9
114.4
114.4
123.9
124.3
124.5
126.4
128.5
128.7
136.6
138.6
151.6
159.1
160.0
160.1
161.0
F
HN
N
N

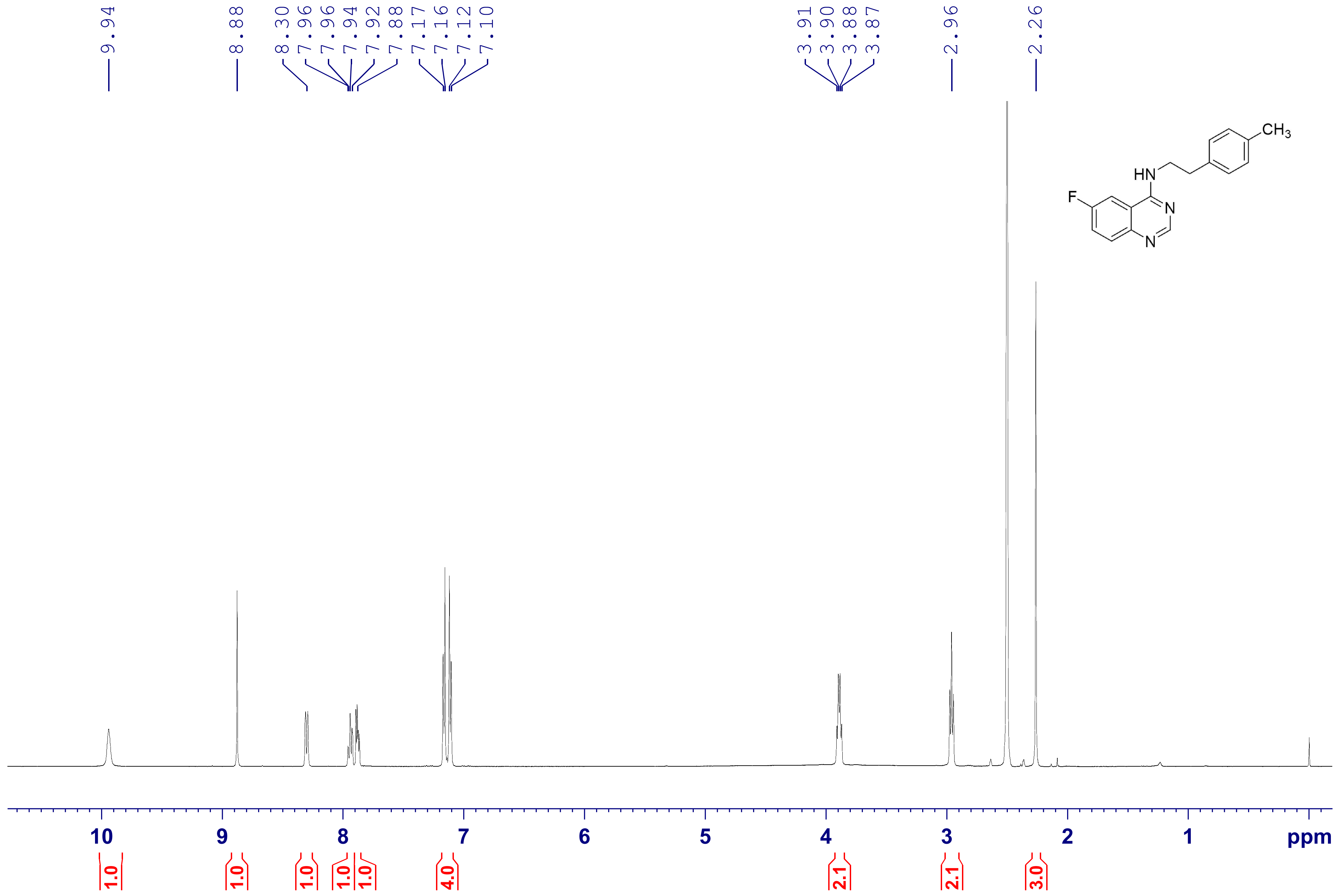
9.94
8.88
8.30
7.96
7.96
7.94
7.92
7.88
7.17
7.16
7.12
7.10
3.91
3.90
3.88
3.87
2.96
2.26
F
HN
N
N
CH3
10
9
8
7
6
5
4
3
2
1
ppm
1.0
1.0
1.0
1.0
1.0
4.0
2.1
2.1
3.0

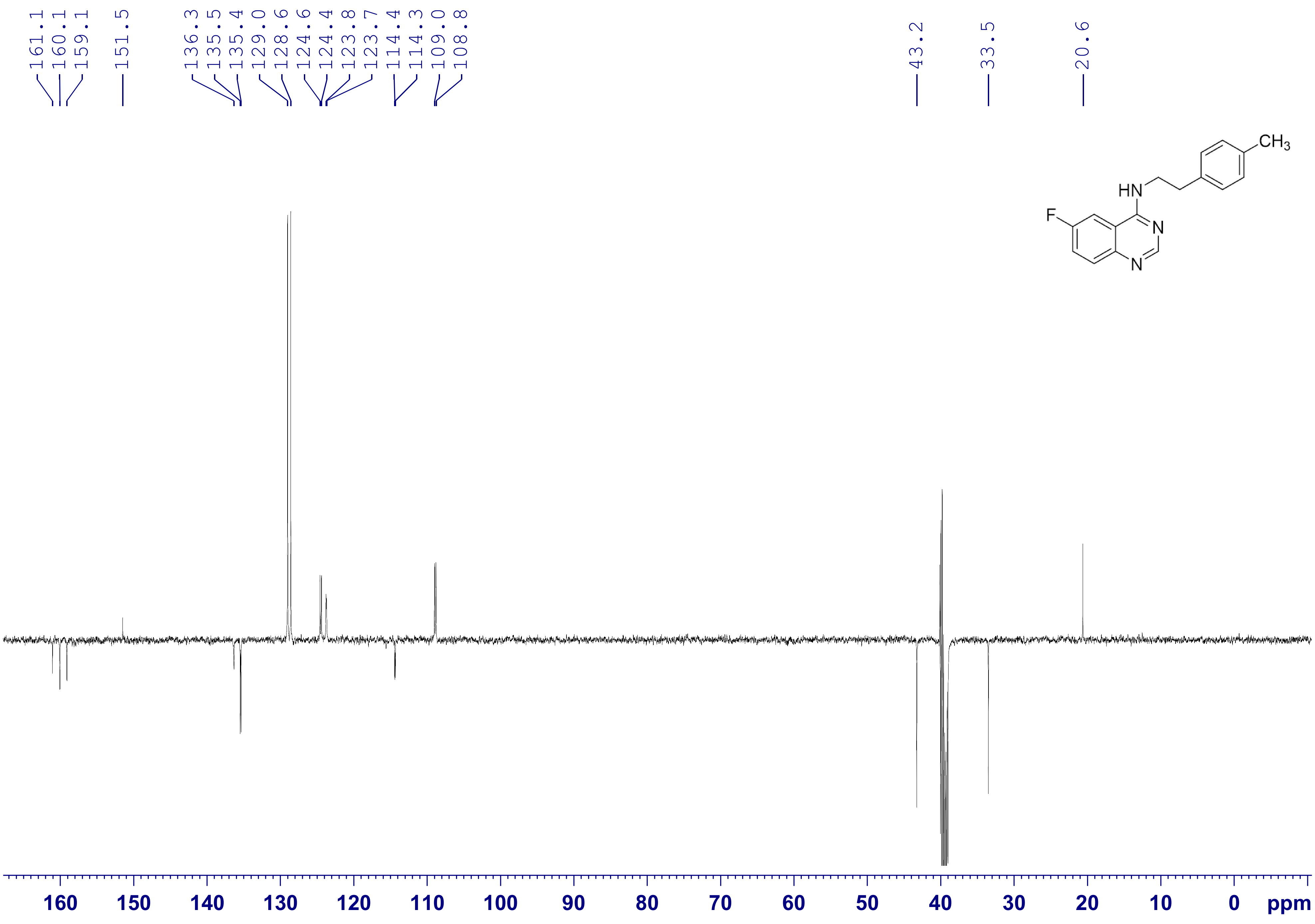
161.1
160.1
159.1
151.5
136.3
135.5
135.4
129.0
128.6
124.6
124.4
123.8
123.7
114.4
114.3
109.0
108.8
43.2
33.5
20.6
CH3
HN
F
N
N
160
150
140
130
120
110
100
90
80
70
60
50
40
30
20
10
0
ppm

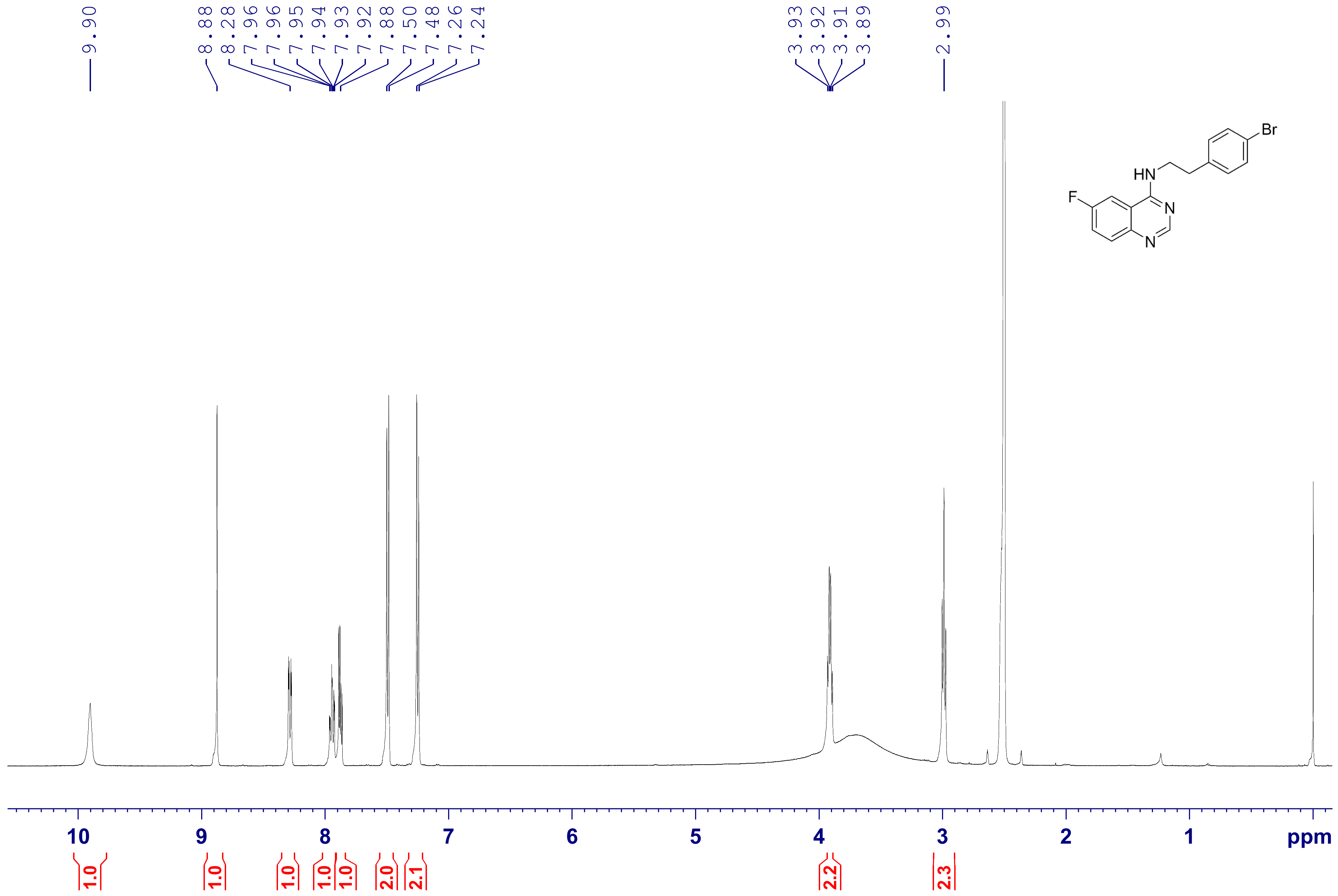

9.90
8.88
8.28
7.96
7.96
7.95
7.94
7.93
7.92
7.88
7.50
7.48
7.26
7.24
3.93
3.92
3.91
3.89
2.99
HN
F
N
N
Br
10
9
8
7
6
5
4
3
2
1
ppm
1.0
1.0
1.0
1.0
1.0
2.0
2.1
2.2
2.3

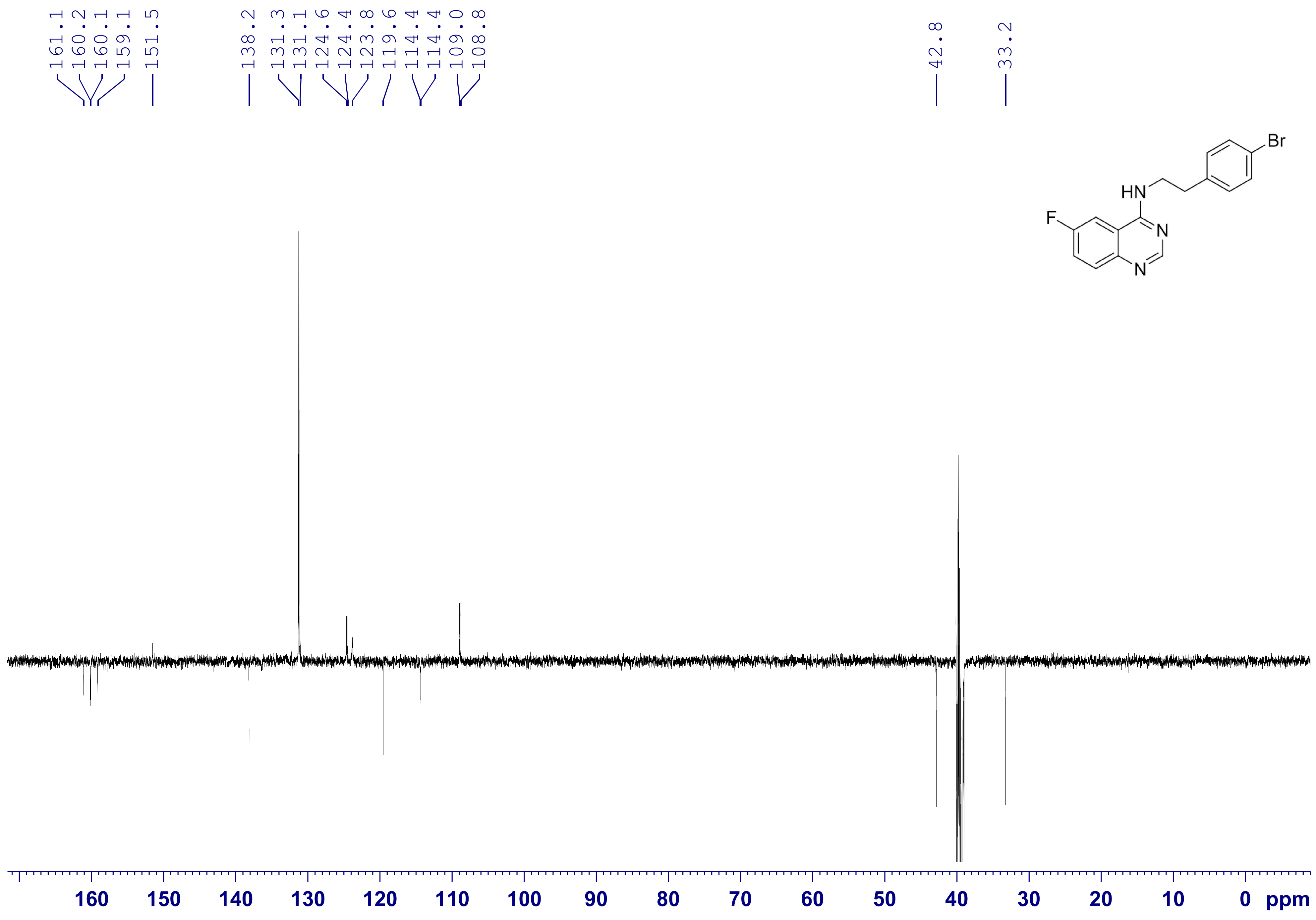
161.1
160.2
160.1
159.1
151.5
138.2
131.3
131.1
124.6
124.4
123.8
119.6
114.4
114.4
109.0
108.8
42.8
33.2
HN
F
N
N
Br
160
150
140
130
120
110
100
90
80
70
60
50
40
30
20
10
0 ppm

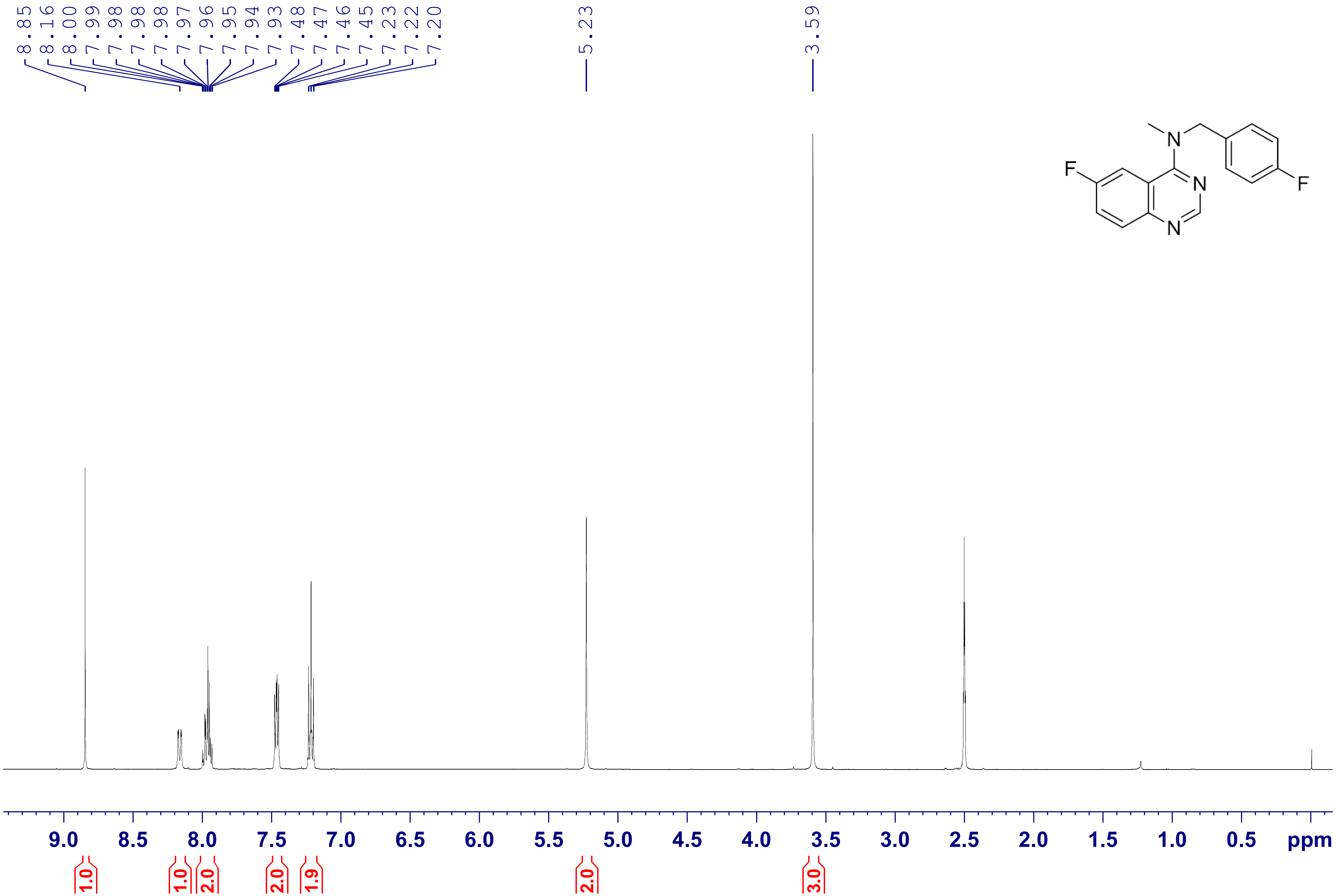
8.85
8.16
8.00
7.99
7.98
7.98
7.98
7.97
7.96
7.95
7.94
7.93
7.48
7.47
7.46
7.45
7.23
7.22
7.20
5.23
3.59
F
N
N
N
F
9.0
8.5
8.0
7.5
7.0
6.5
6.0
5.5
5.0
4.5
4.0
3.5
3.0
2.5
2.0
1.5
1.0
0.5
ppm
1.0
1.0
2.0
2.0
1.9
2.0
3.0

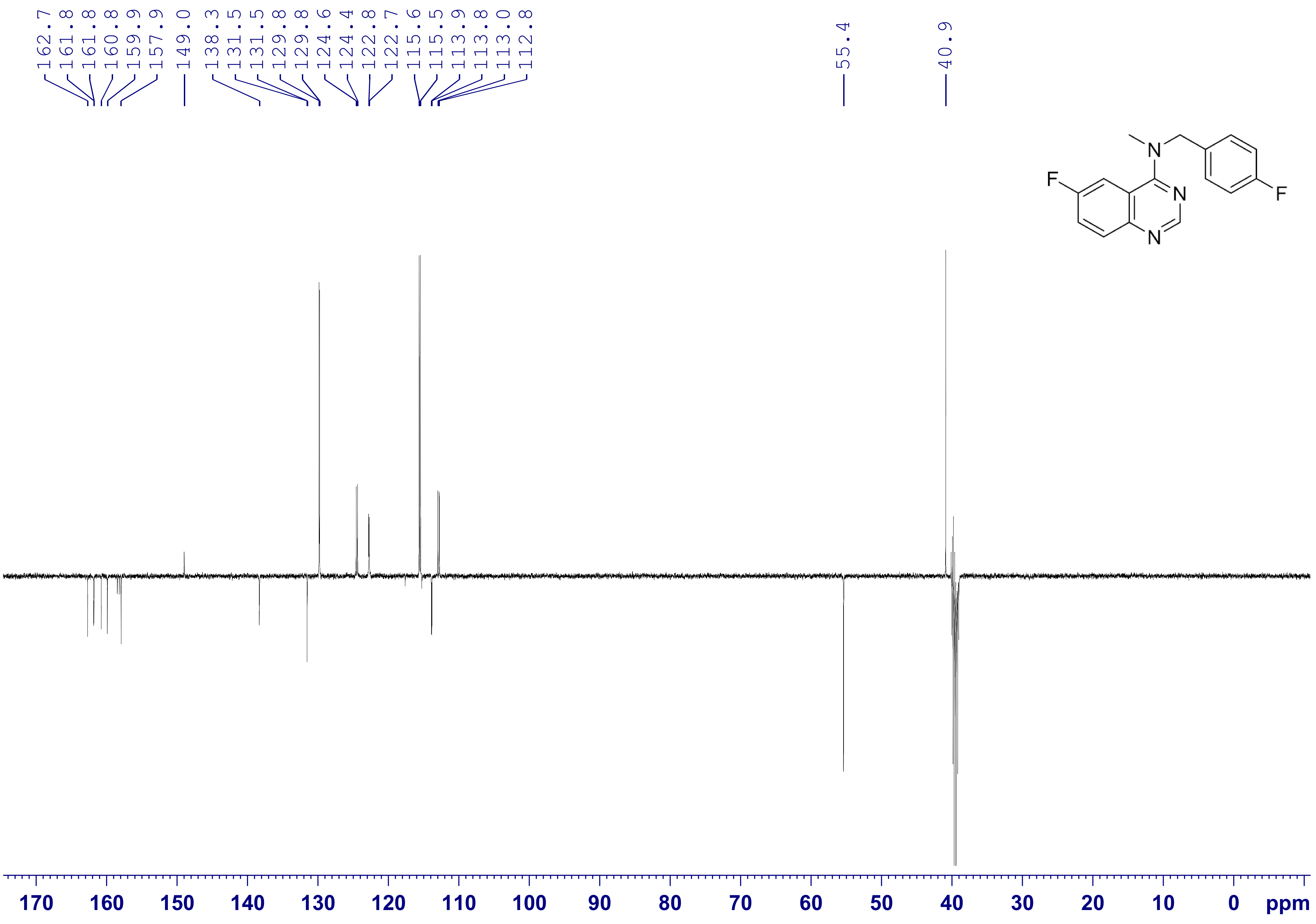
162.7
161.8
161.8
160.8
159.9
157.9
149.0
138.3
131.5
131.5
129.8
129.8
124.6
124.4
122.8
122.7
115.6
115.5
113.9
113.8
113.0
112.8
55.4
40.9
170
160
150
140
130
120
110
100
90
80
70
60
50
40
30
20
10
0
ppm

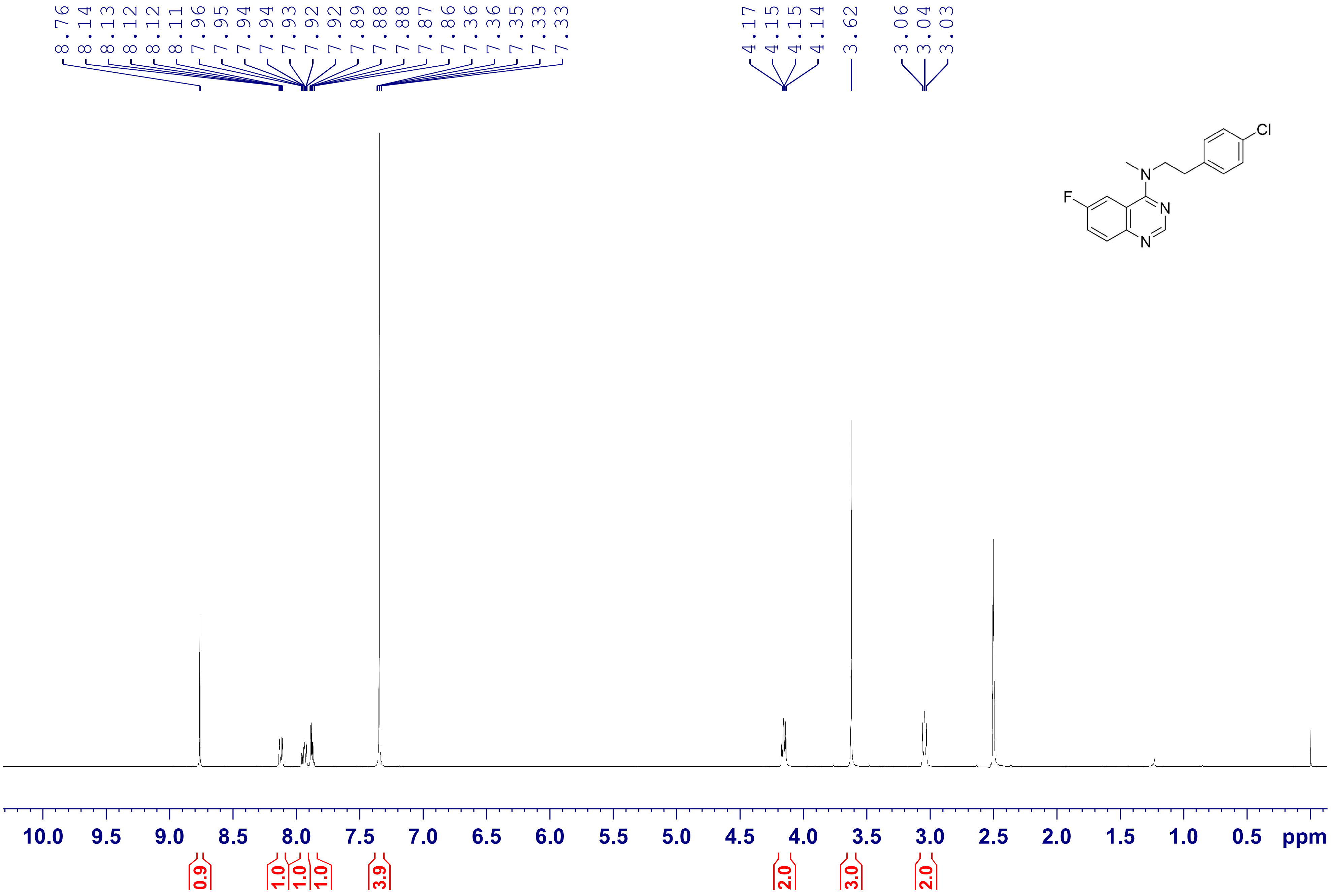
8.76
8.14
8.13
8.12
8.12
8.11
7.96
7.95
7.94
7.94
7.93
7.92
7.92
7.89
7.88
7.88
7.87
7.86
7.36
7.36
7.35
7.33
7.33
4.17
4.15
4.15
4.14
3.62
3.06
3.04
3.03
F
N
N
N
Cl
10.0
9.5
9.0
8.5
8.0
7.5
7.0
6.5
6.0
5.5
5.0
4.5
4.0
3.5
3.0
2.5
2.0
1.5
1.0
0.5
ppm
0.9
1.0
1.0
1.0
3.9
2.0
3.0
2.0

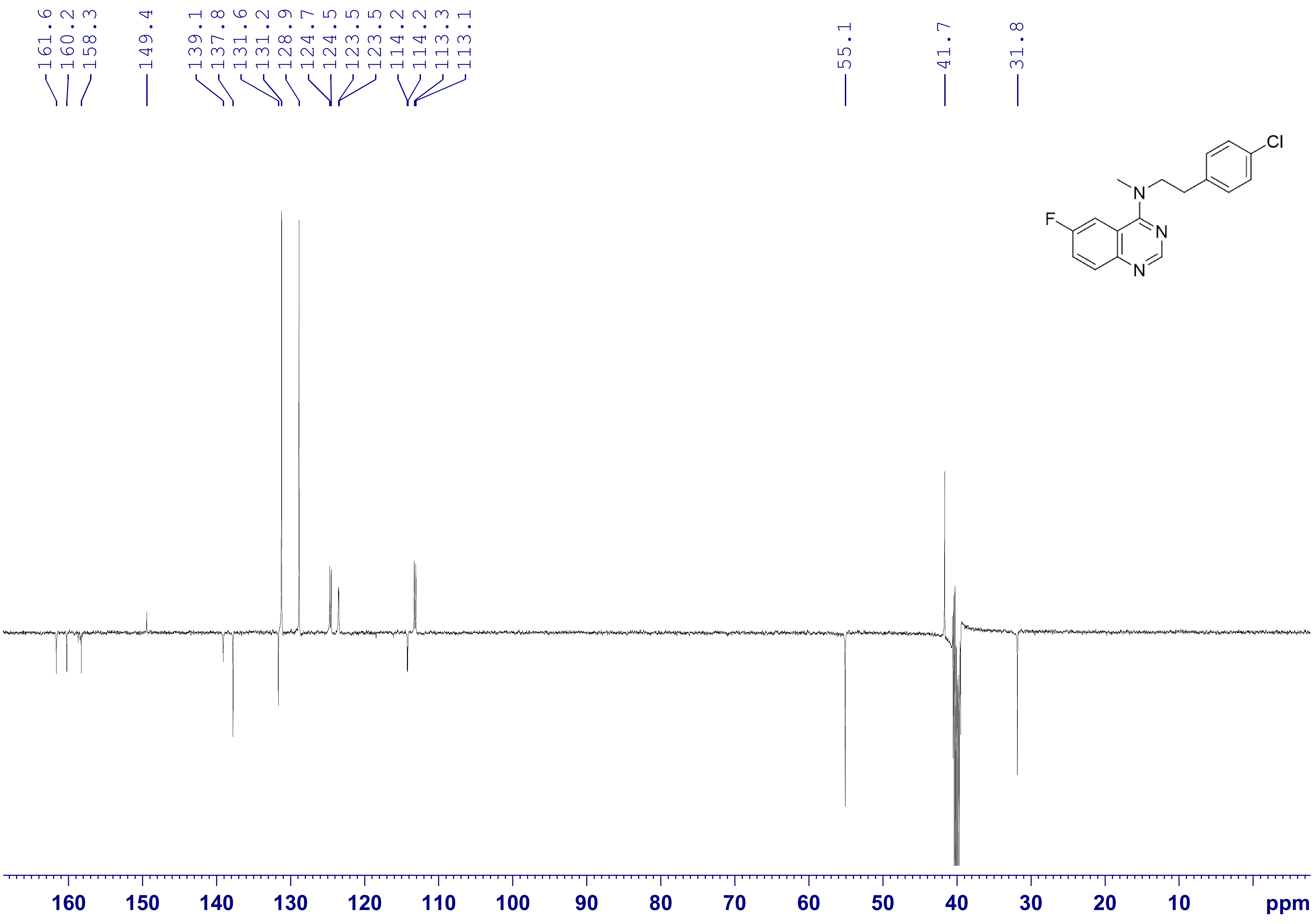
161.6
160.2
158.3
149.4
139.1
137.8
131.6
131.2
128.9
124.7
124.5
123.5
123.5
114.2
114.2
113.3
113.1
55.1
41.7
31.8
F
N
N
N
Cl
160
150
140
130
120
110
100
90
80
70
60
50
40
30
20
10
ppm

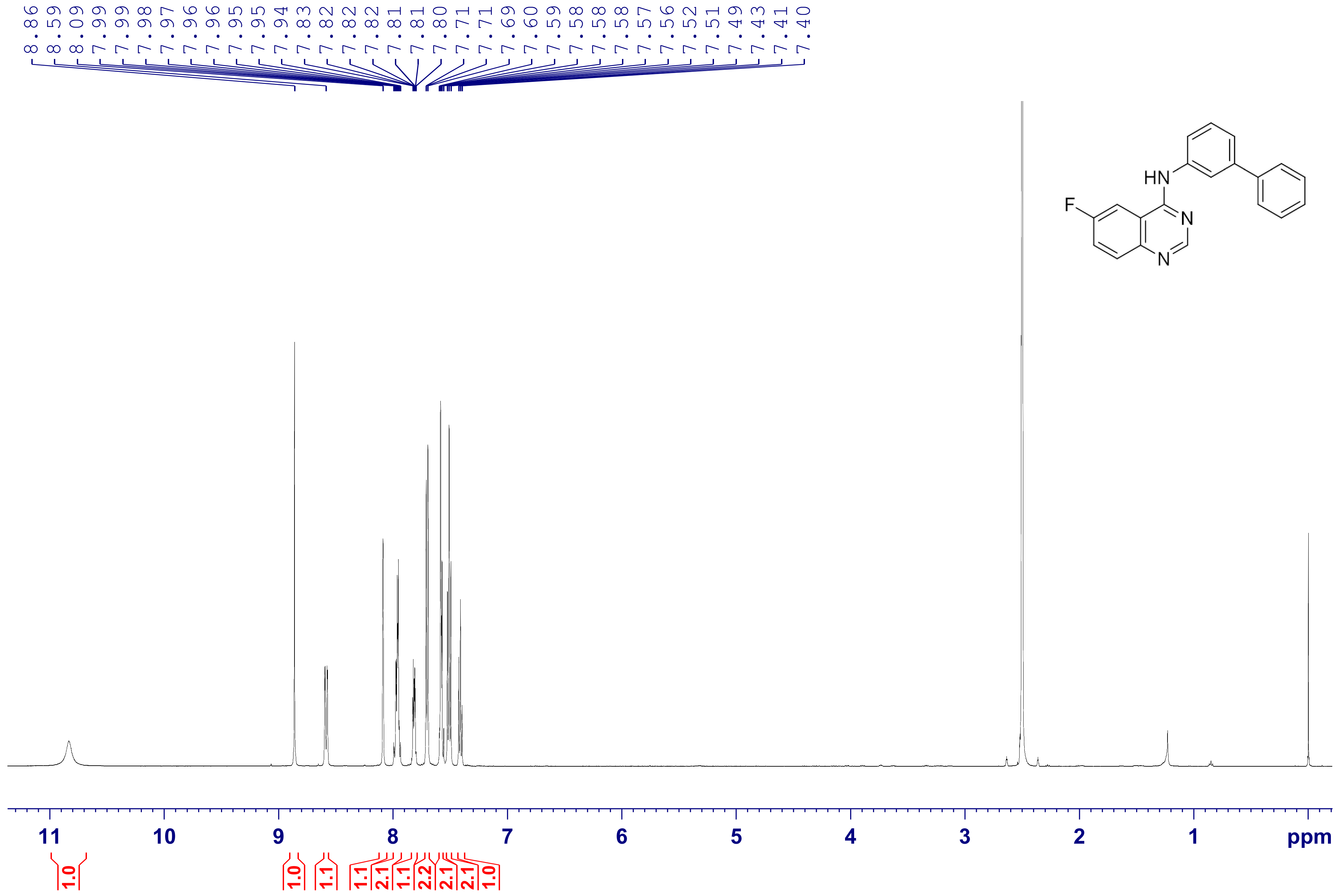

8.86
8.59
8.09
7.99
7.99
7.98
7.97
7.96
7.96
7.95
7.95
7.94
7.83
7.82
7.82
7.82
7.81
7.81
7.80
7.71
7.71
7.69
7.60
7.59
7.58
7.58
7.58
7.57
7.56
7.52
7.51
7.49
7.43
7.41
7.40
HN
F
N
N
11
10
9
8
7
6
5
4
3
2
1
ppm
1.0
1.0
1.1
1.1
2.1
1.1
2.2
2.1
2.1
1.0

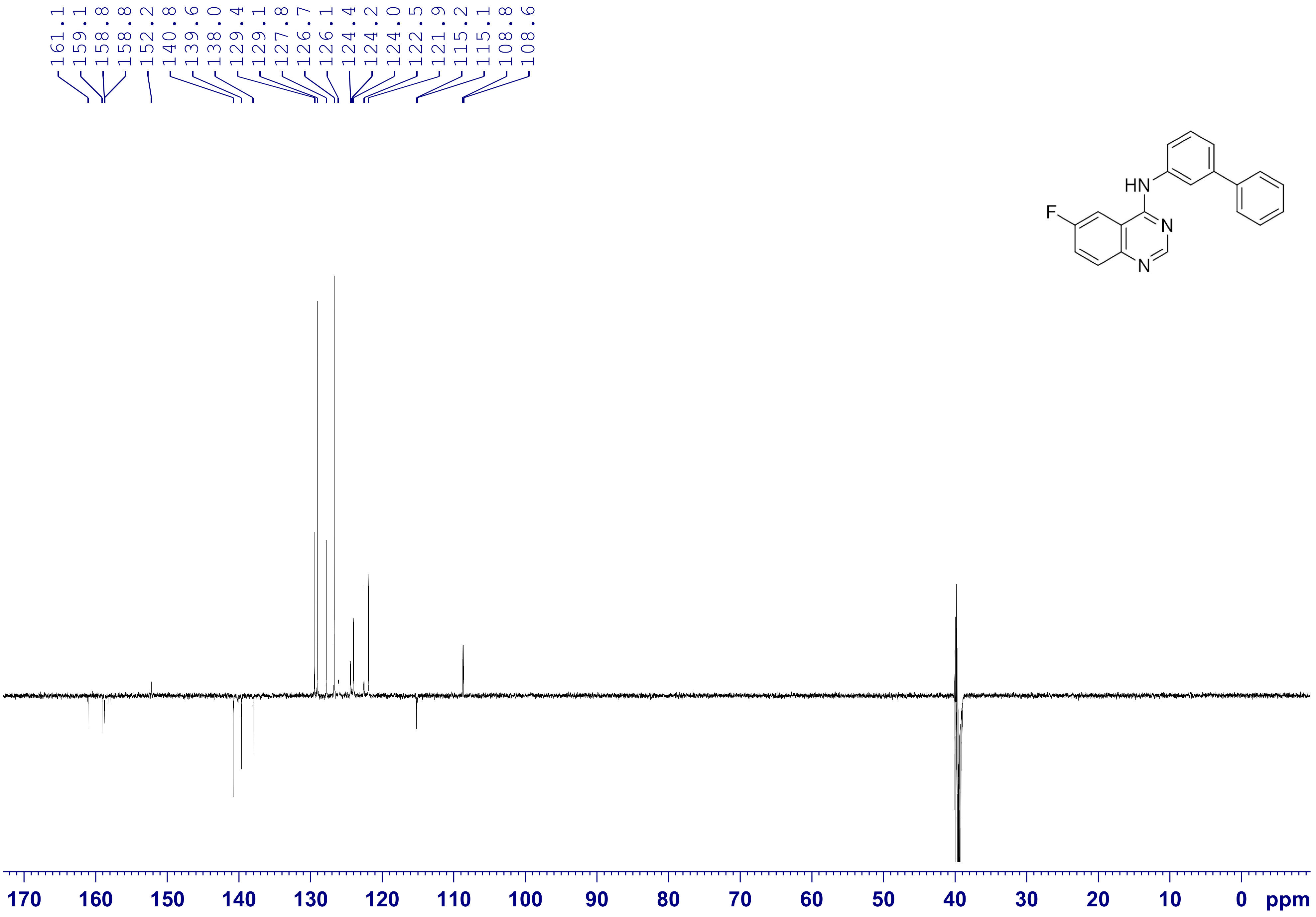
161.1
159.1
158.8
158.8
152.2
140.8
139.6
138.0
129.4
129.1
127.8
126.7
126.1
124.4
124.2
124.0
122.5
121.9
115.2
115.1
108.8
108.6
HN
F
N
N
170 160 150 140 130 120 110 100 90 80 70 60 50 40 30 20 10 0 ppm

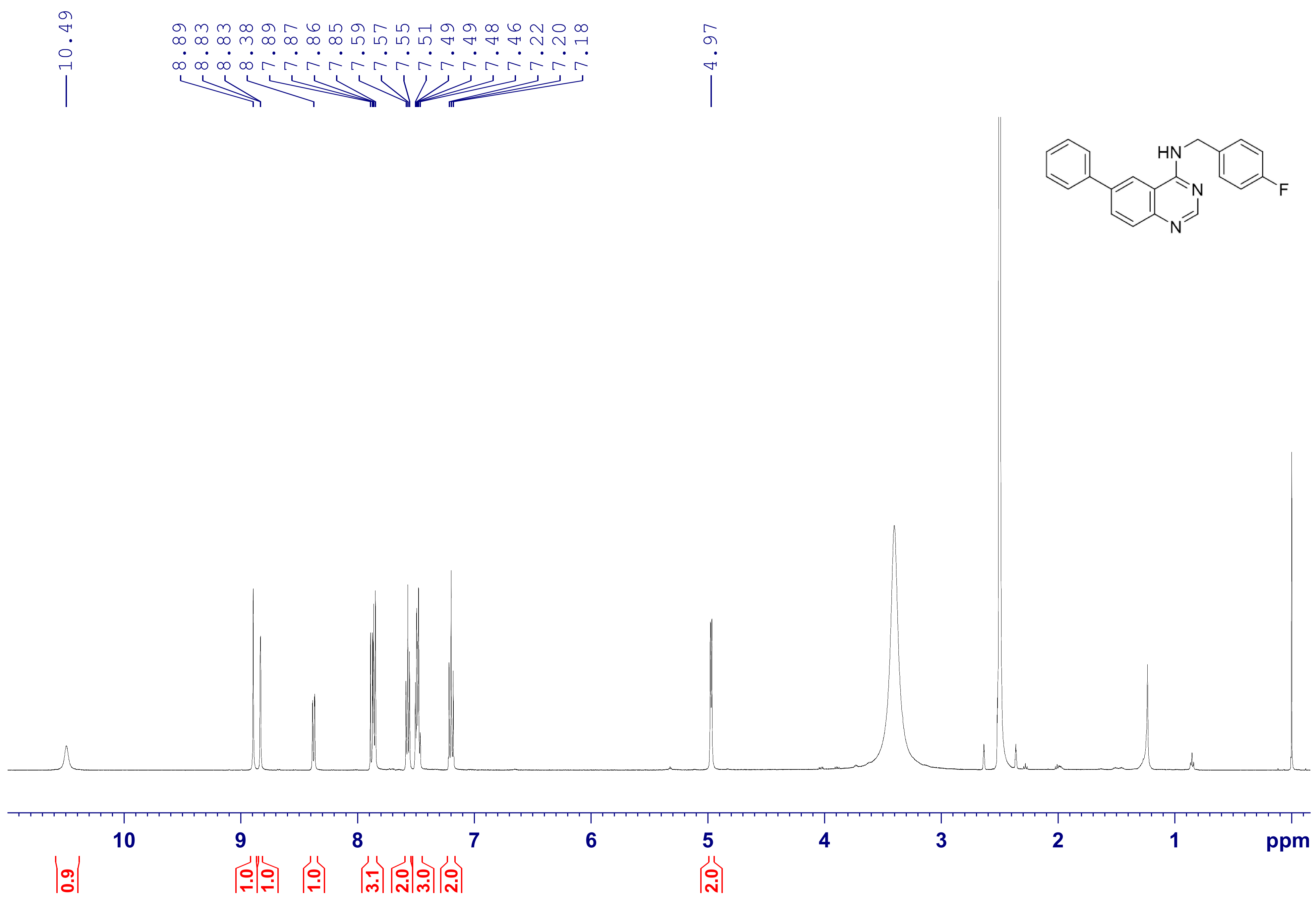
10.49
8.89
8.83
8.83
8.38
7.89
7.87
7.86
7.85
7.59
7.57
7.55
7.51
7.49
7.49
7.48
7.46
7.22
7.20
7.18
4.97
HN
N
N
F
10
9
8
7
6
5
4
3
2
1
ppm
0.9
1.0
1.0
1.0
3.1
2.0
3.0
2.0
2.0

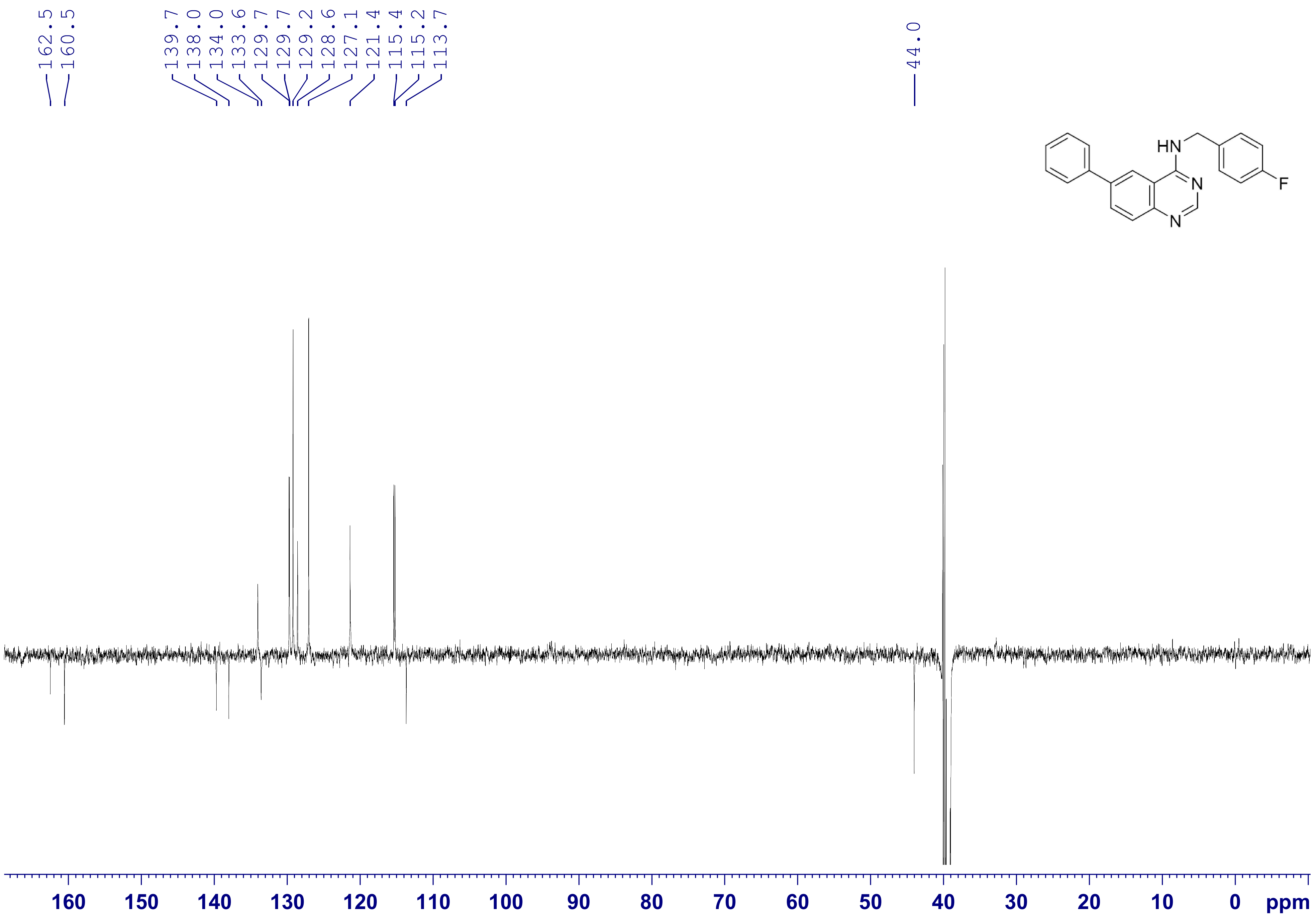

162.5
160.5
139.7
138.0
134.0
133.6
129.7
129.7
129.2
128.6
127.1
121.4
115.4
115.2
113.7
44.0
HN
N
N
F
160
150
140
130
120
110
100
90
80
70
60
50
40
30
20
10
0
ppm

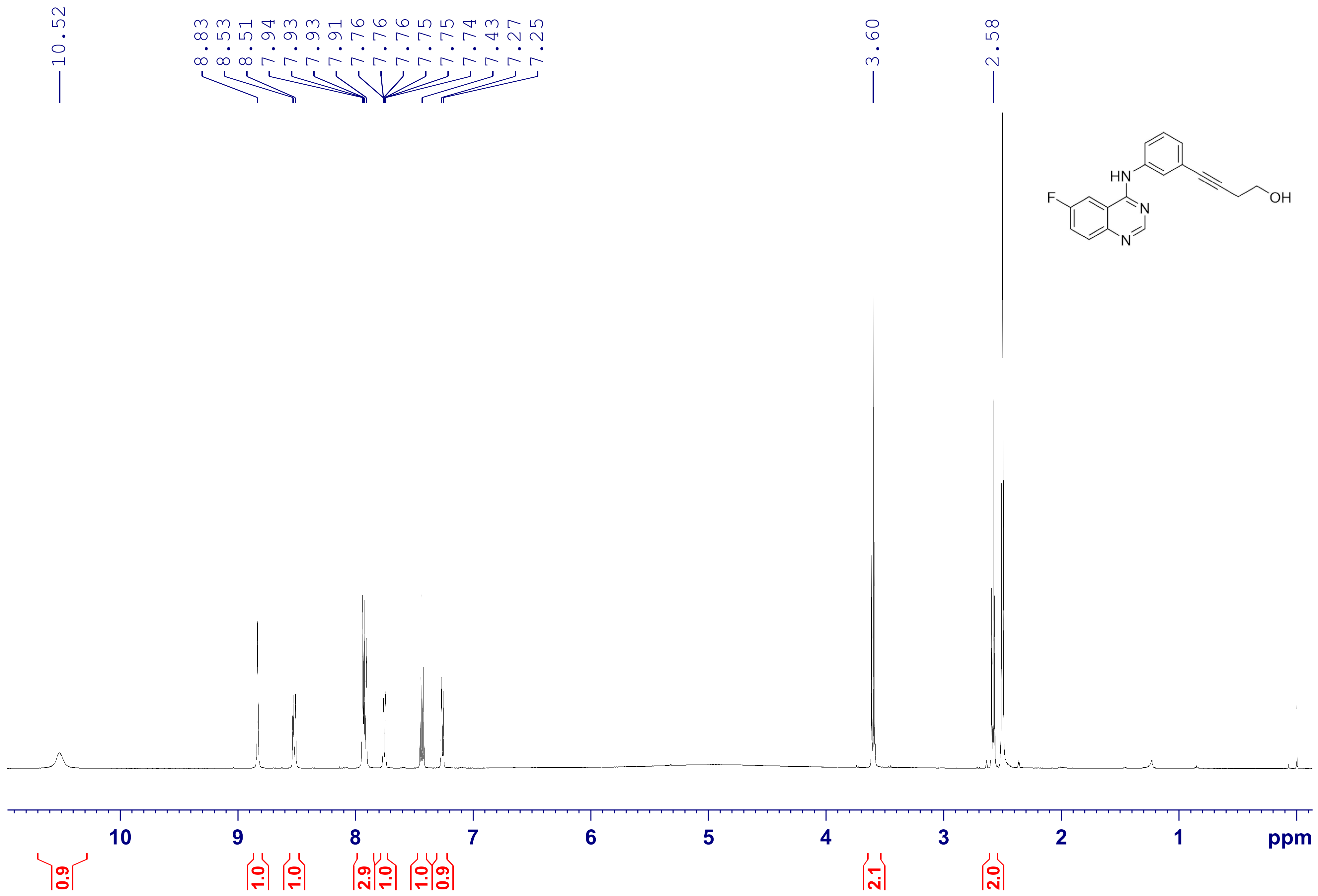
10.52
8.83
8.53
8.51
7.94
7.93
7.93
7.91
7.76
7.76
7.76
7.75
7.75
7.74
7.43
7.27
7.25
3.60
2.58
F
HN
N
N
OH
10
9
8
7
6
5
4
3
2
1
ppm
0.9
1.0
1.0
2.9
1.0
1.0
0.9
2.1
2.0

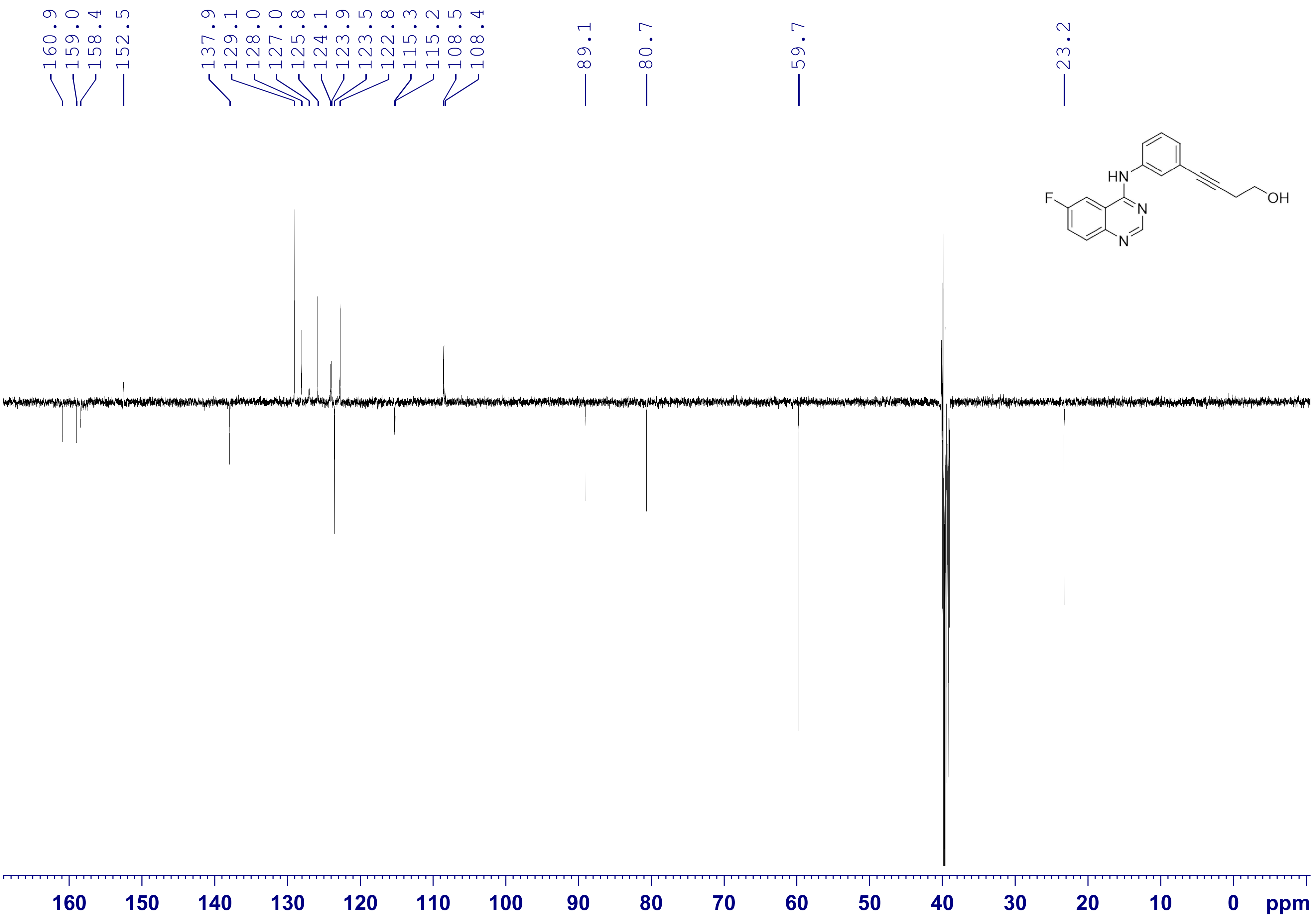
160.9
159.0
158.4
152.5
137.9
129.1
128.0
127.0
125.8
124.1
123.9
123.5
122.8
115.3
115.2
108.5
108.4
89.1
80.7
59.7
23.2
F
HN
N
N
OH
160
150
140
130
120
110
100
90
80
70
60
50
40
30
20
10
0
ppm

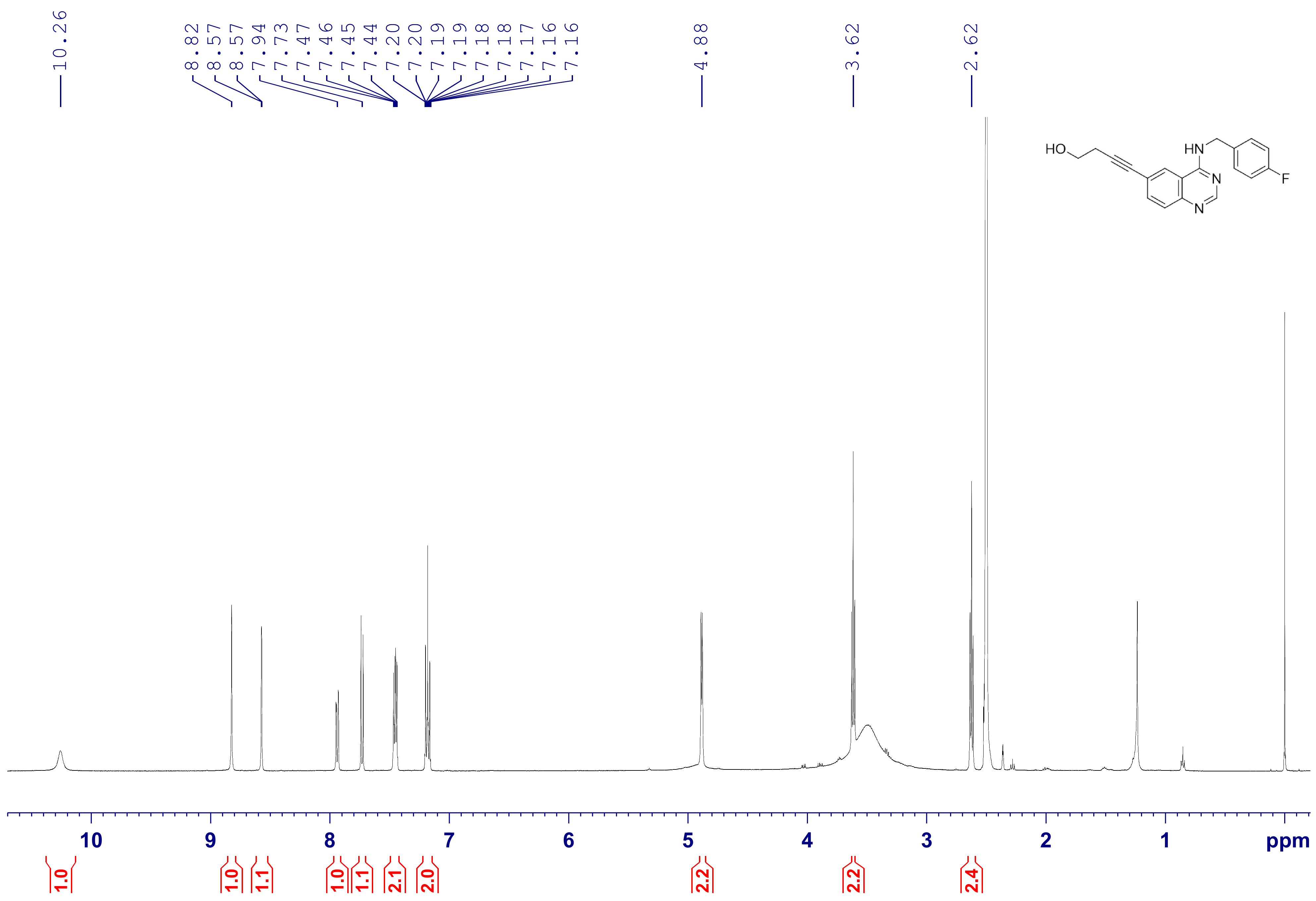
10.26
8.82
8.57
8.57
7.94
7.73
7.47
7.46
7.45
7.44
7.20
7.20
7.19
7.19
7.18
7.18
7.17
7.16
7.16
4.88
3.62
2.62
HO
HN
N
N
F
10
9
8
7
6
5
4
3
2
1
ppm
1.0
1.0
1.1
1.0
1.1
2.1
2.0
2.2
2.2
2.4

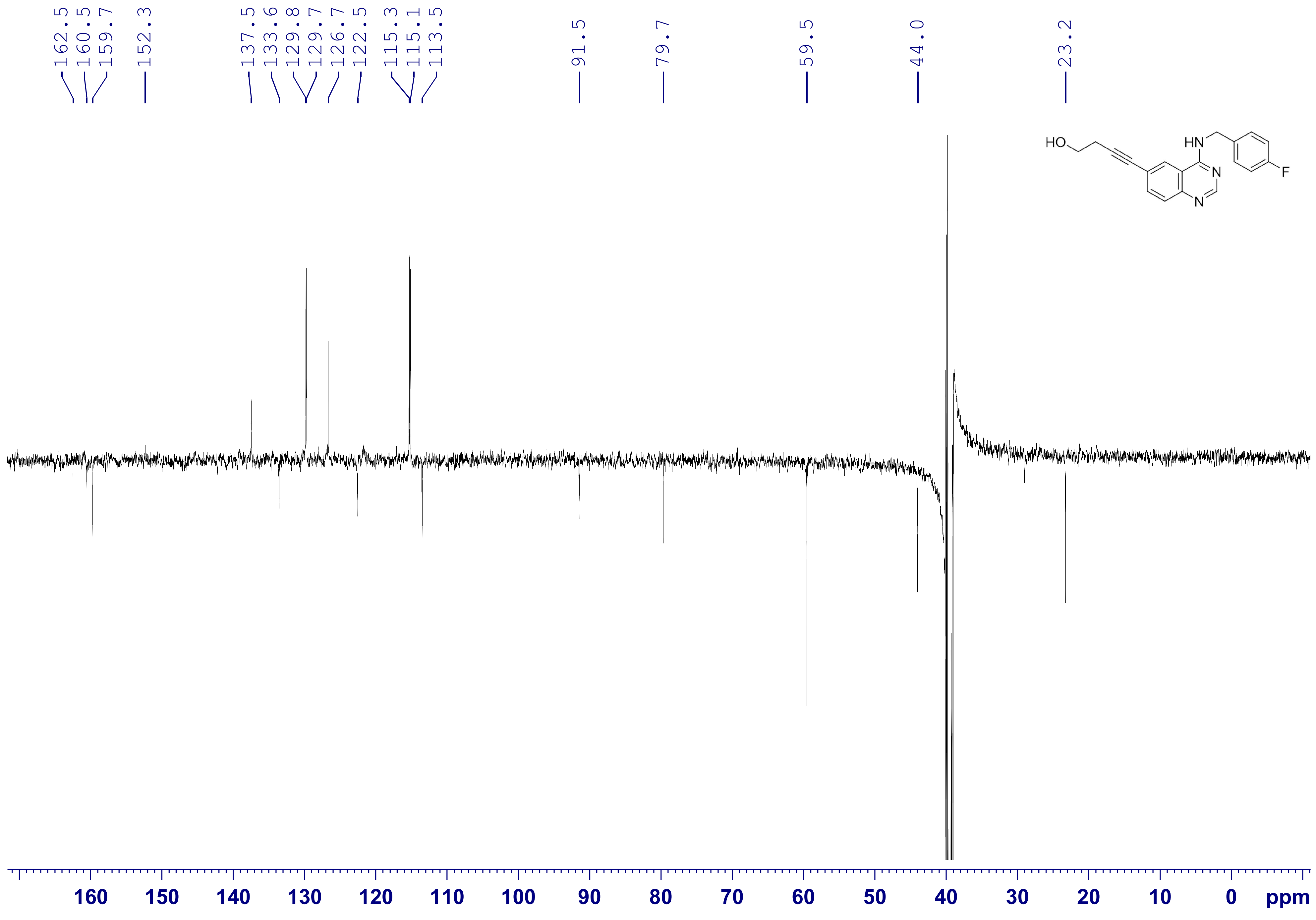
162.5
160.5
159.7
152.3
137.5
133.6
129.8
129.7
126.7
122.5
115.3
115.1
113.5
91.5
79.7
59.5
44.0
23.2
HO
HN
N
N
F
160
150
140
130
120
110
100
90
80
70
60
50
40
30
20
10
0
ppm

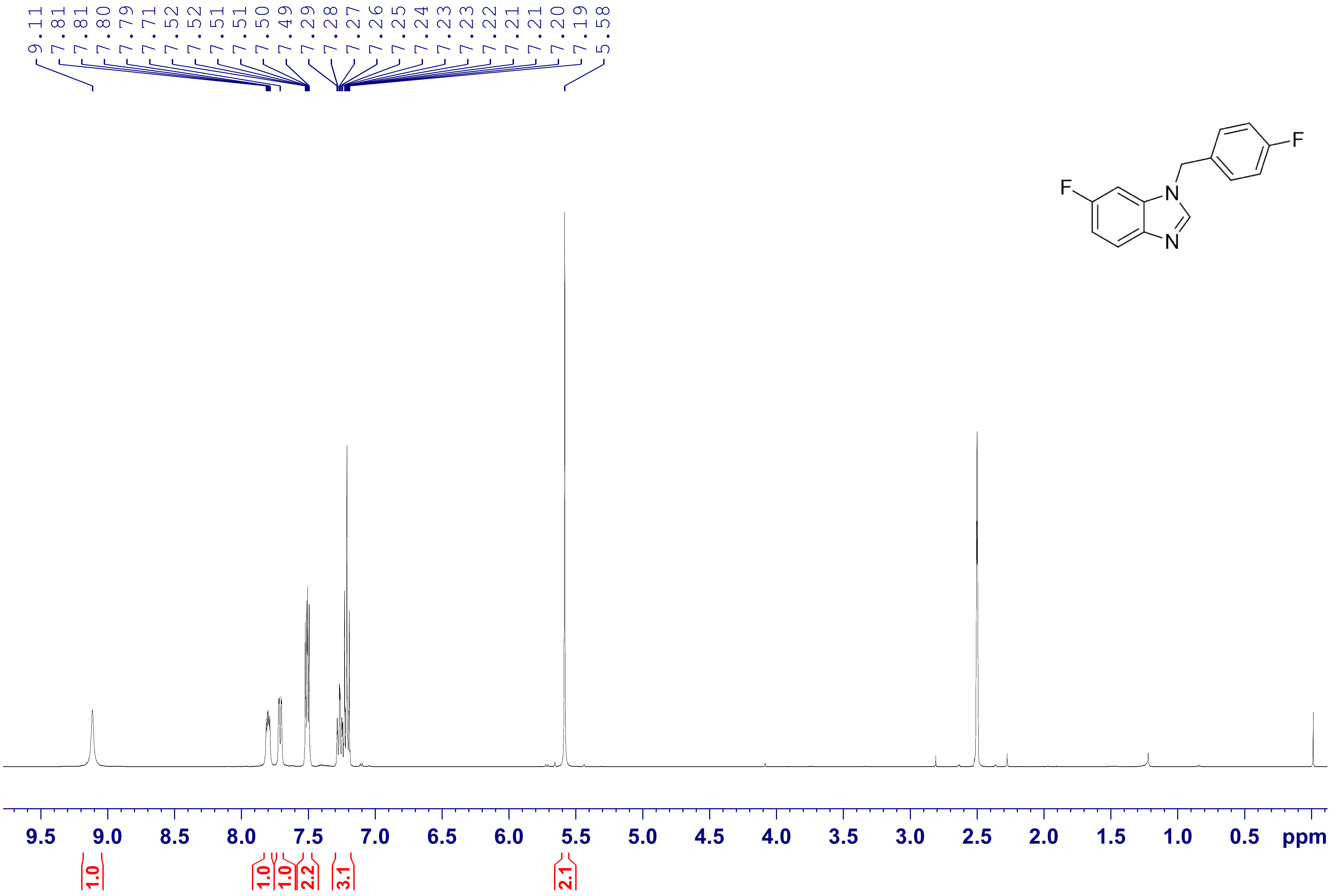
9.11
7.81
7.81
7.80
7.79
7.71
7.52
7.52
7.51
7.51
7.50
7.49
7.29
7.28
7.27
7.26
7.25
7.24
7.23
7.23
7.22
7.21
7.21
7.20
7.19
5.58
F
N
N
F
9.5
9.0
8.5
8.0
7.5
7.0
6.5
6.0
5.5
5.0
4.5
4.0
3.5
3.0
2.5
2.0
1.5
1.0
0.5
ppm
1.0
1.0
1.0
2.2
3.1
2.1

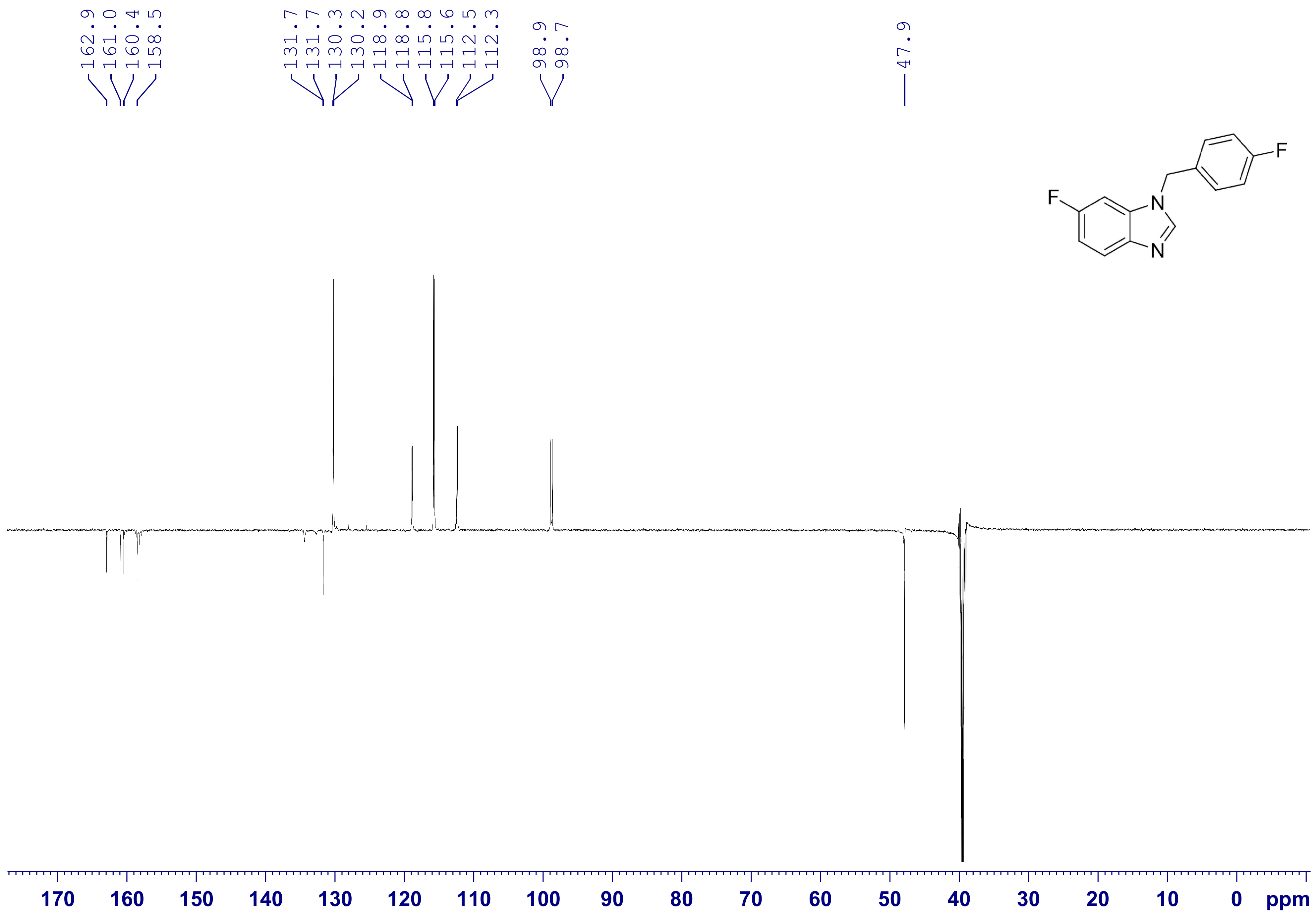

162.9
161.0
160.4
158.5
131.7
131.7
130.3
130.2
118.9
118.8
115.8
115.6
112.5
112.3
98.9
98.7
47.9
F
N
N
F
170 160 150 140 130 120 110 100 90 80 70 60 50 40 30 20 10 0 ppm

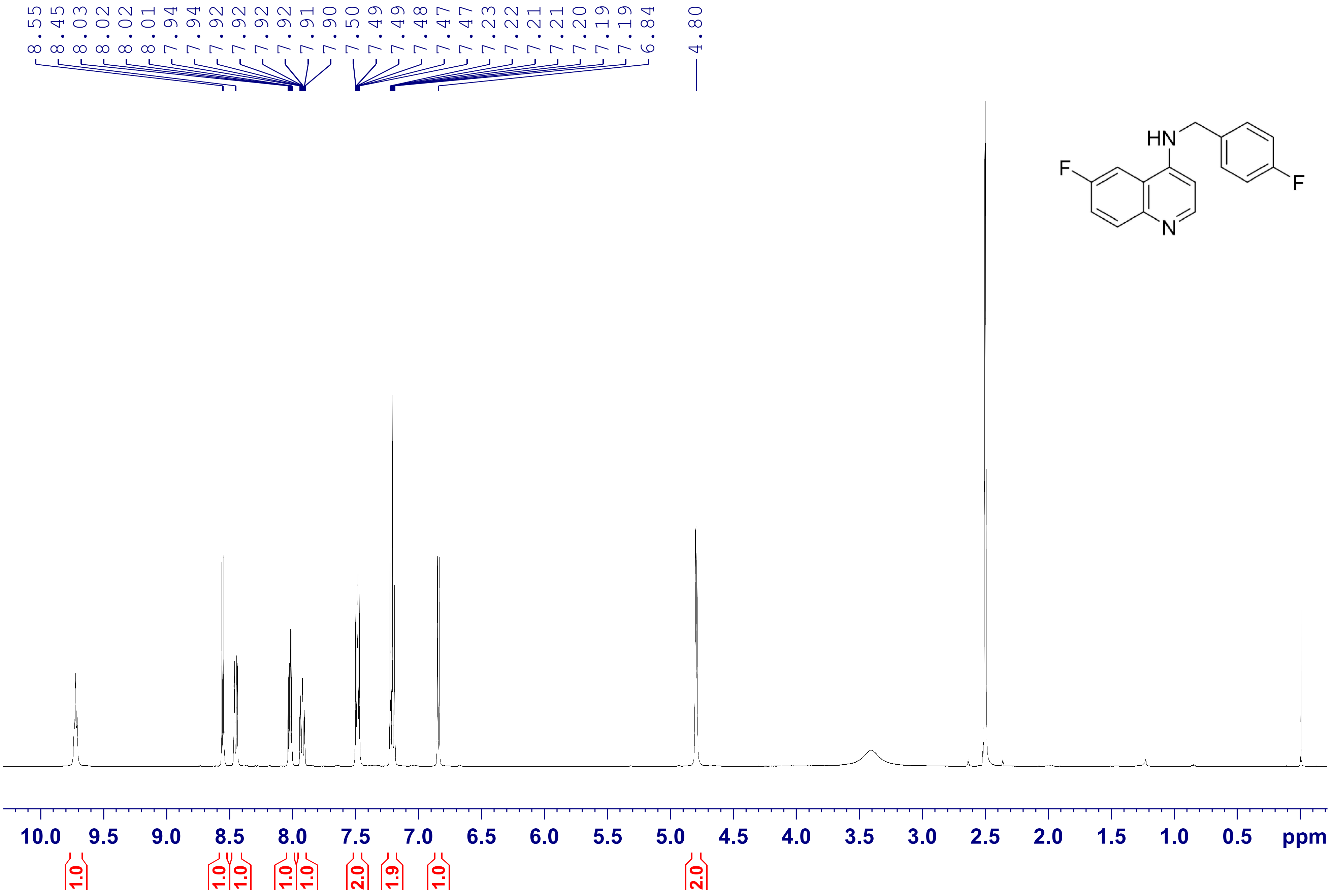
8.55
8.45
8.03
8.02
8.02
8.01
7.94
7.94
7.92
7.92
7.92
7.92
7.91
7.90
7.50
7.49
7.49
7.48
7.47
7.47
7.23
7.22
7.21
7.21
7.20
7.19
7.19
6.84
4.80
HN
F
F
N
1.0
1.0
1.0
1.0
1.0
2.0
1.9
1.0
2.0
10.0
9.5
9.0
8.5
8.0
7.5
7.0
6.5
6.0
5.5
5.0
4.5
4.0
3.5
3.0
2.5
2.0
1.5
1.0
0.5
ppm

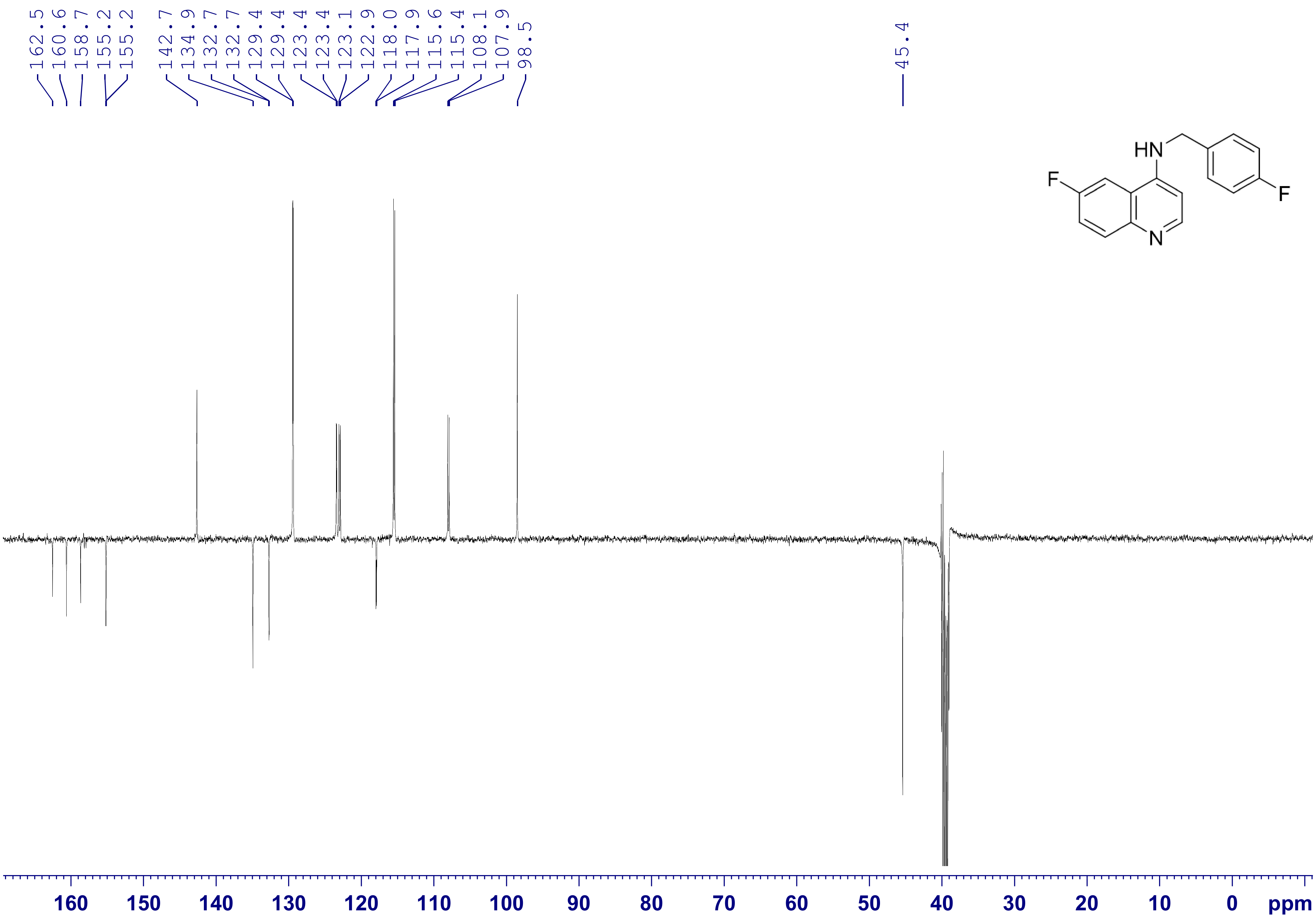
162.5
160.6
158.7
155.2
155.2
142.7
134.9
132.7
132.7
129.4
129.4
123.4
123.4
123.1
122.9
118.0
117.9
115.6
115.4
108.1
107.9
98.5
45.4
HN
F
F
N
160 150 140 130 120 110 100 90 80 70 60 50 40 30 20 10 0 ppm

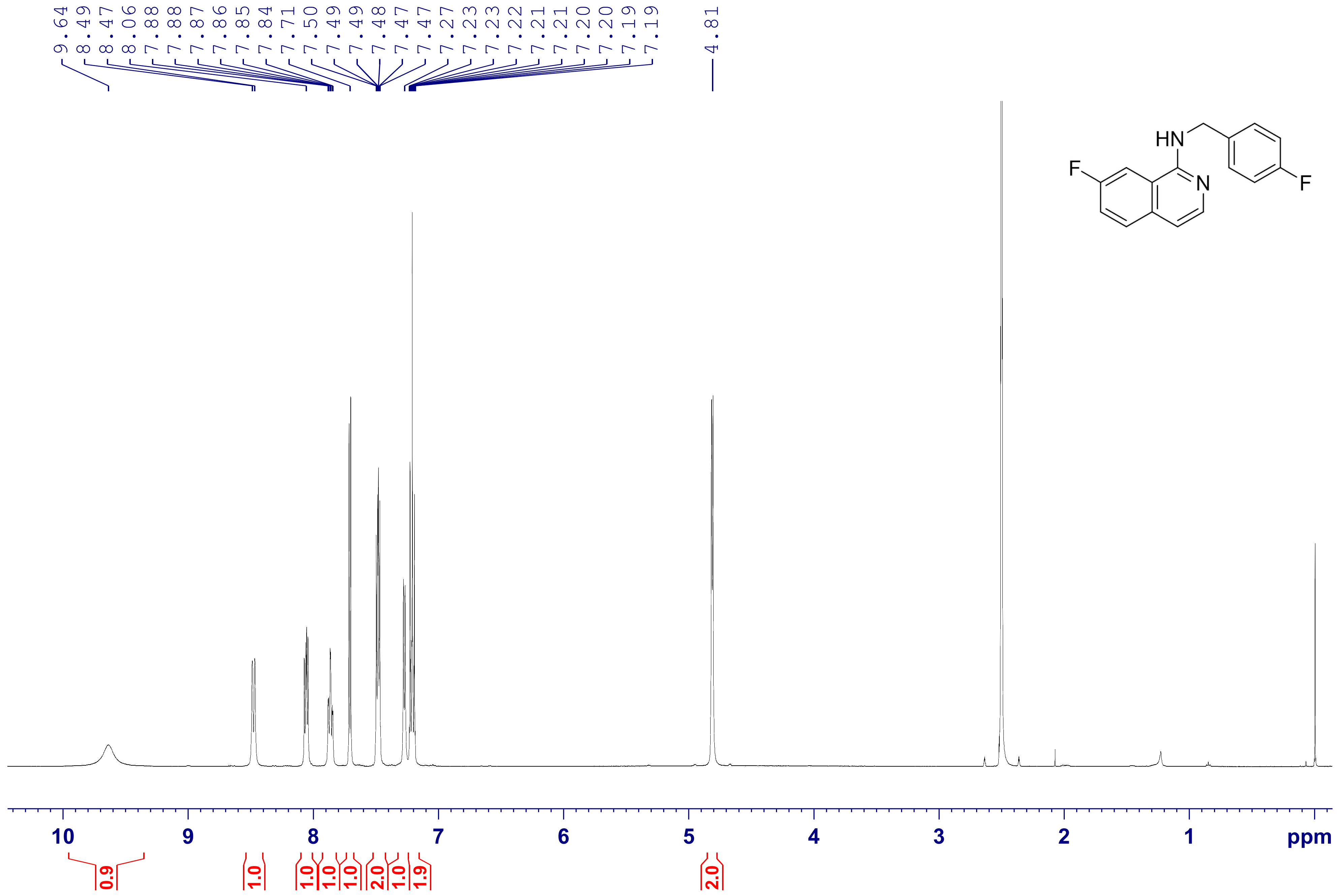
9.64
8.49
8.47
8.06
7.88
7.88
7.87
7.86
7.85
7.84
7.71
7.50
7.49
7.49
7.48
7.47
7.47
7.27
7.23
7.23
7.22
7.21
7.21
7.20
7.20
7.19
7.19
4.81
HN
F
N
F
10
9
8
7
6
5
4
3
2
1
ppm
0.9
1.0
1.0
1.0
1.0
2.0
1.0
1.9
2.0

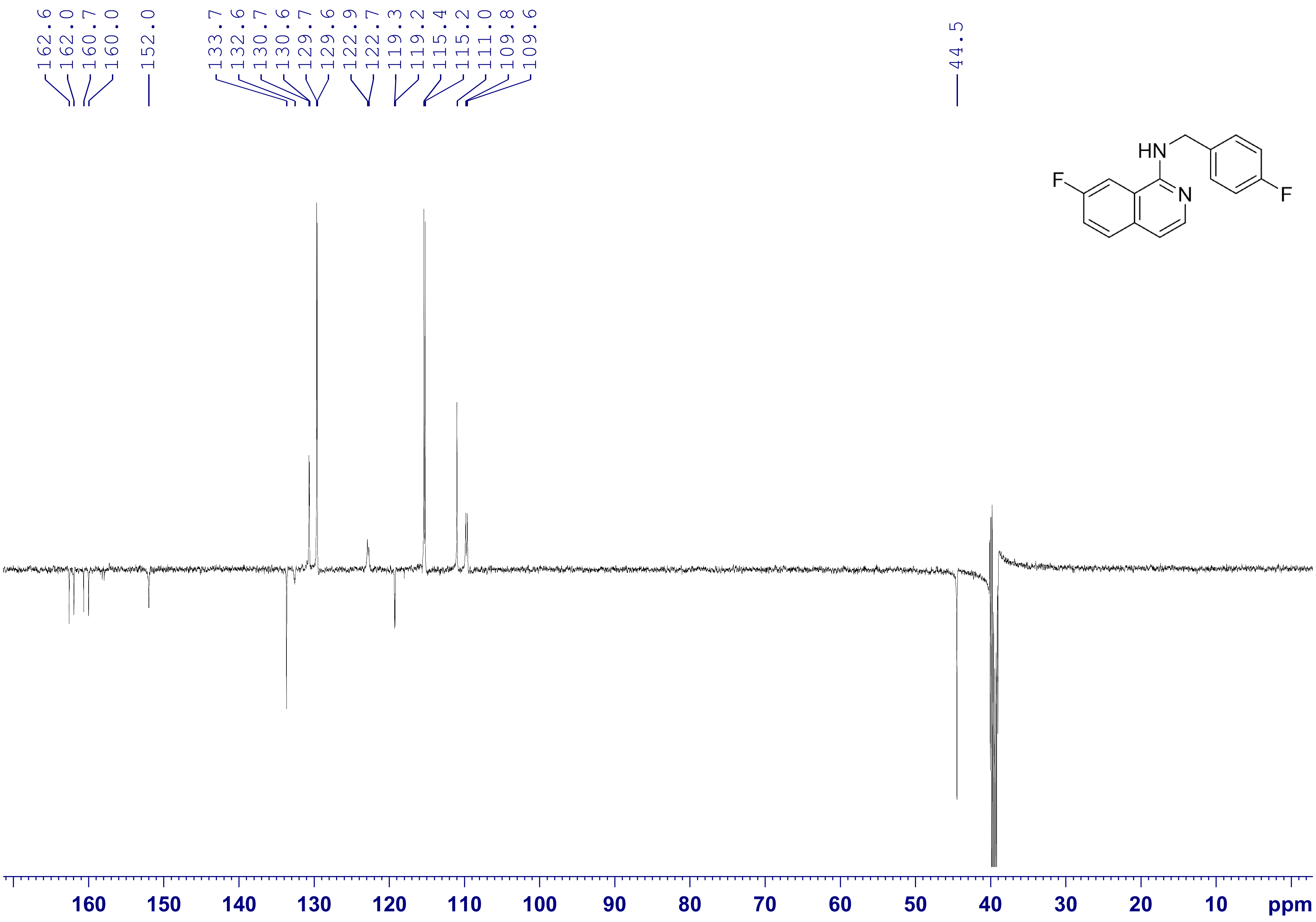
162.6
162.0
160.7
160.0
152.0
133.7
132.6
130.7
130.6
129.7
129.6
122.9
122.7
119.3
119.2
115.4
115.2
111.0
109.8
109.6
44.5
HN
F
N
F
160
150
140
130
120
110
100
90
80
70
60
50
40
30
20
10
ppm

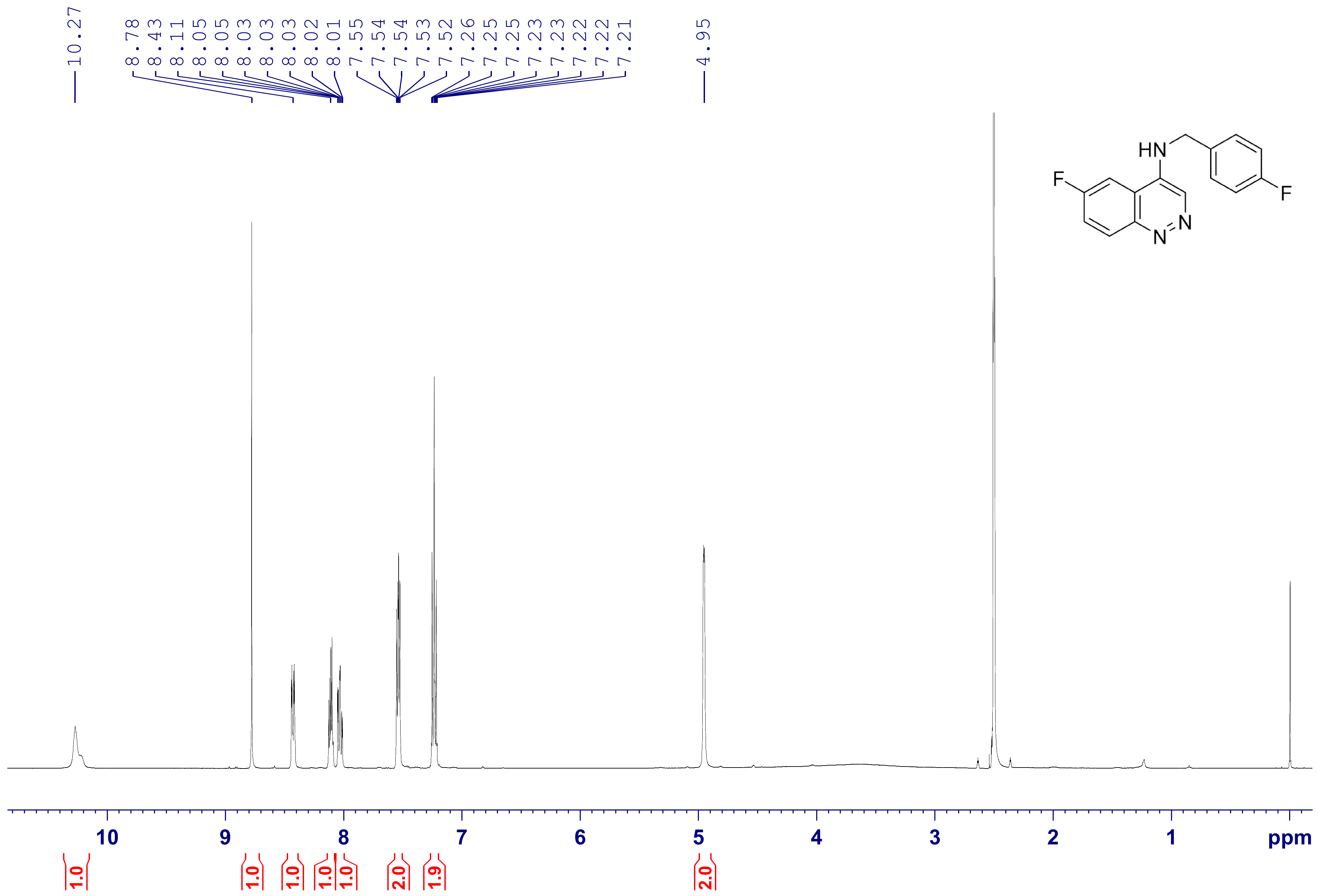
10.27
8.78
8.43
8.11
8.05
8.05
8.03
8.03
8.03
8.02
8.01
7.55
7.54
7.54
7.53
7.52
7.26
7.25
7.25
7.23
7.23
7.22
7.22
7.21
4.95
HN
F
F
N
N
10
9
8
7
6
5
4
3
2
1
ppm
1.0
1.0
1.0
1.0
1.0
2.0
1.9
2.0

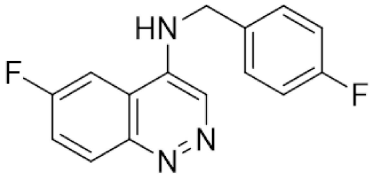
F
HN
N
N
F

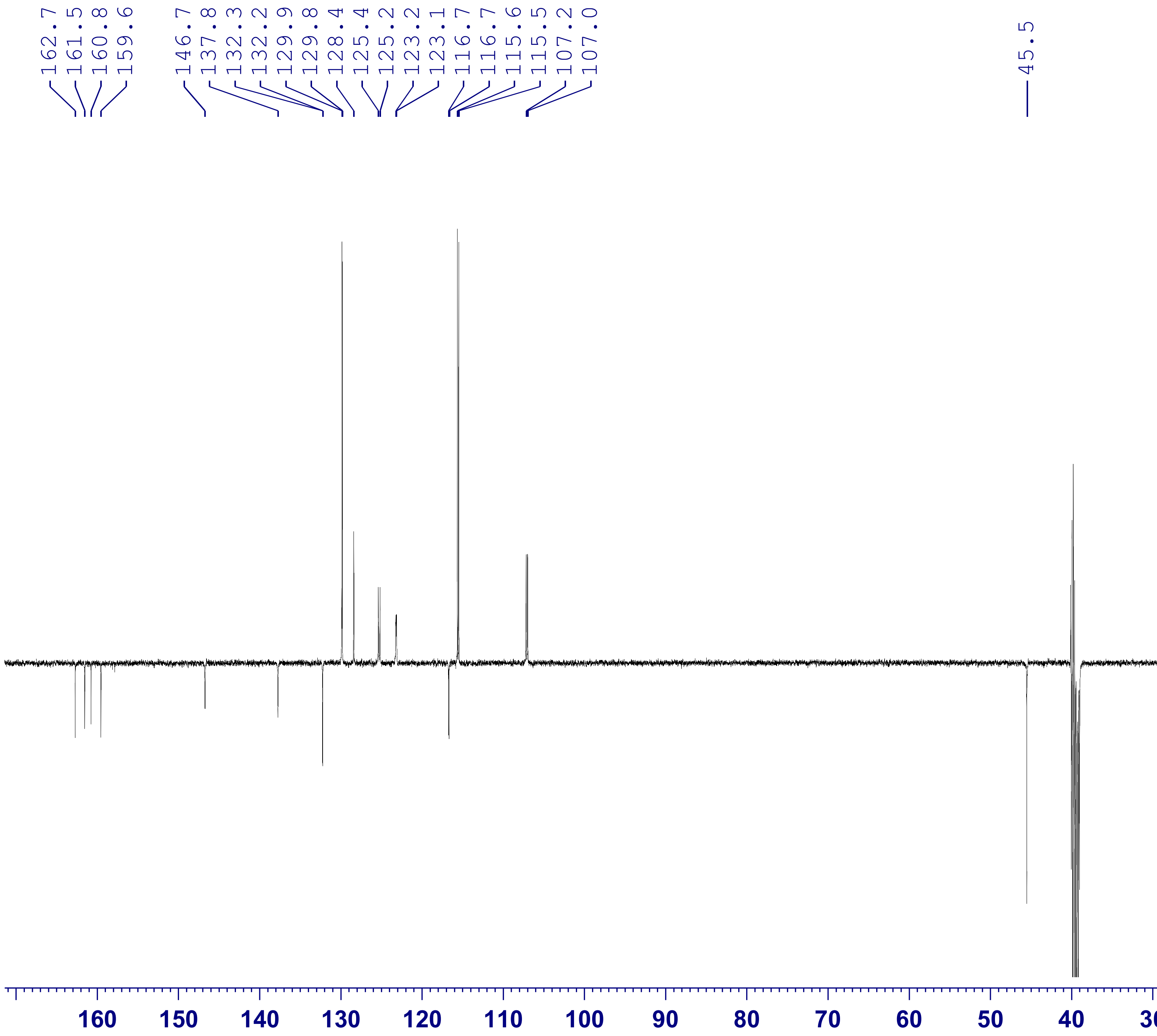
162.7
161.5
160.8
159.6
146.7
137.8
132.3
132.2
129.9
129.8
128.4
125.4
125.2
123.2
123.1
116.7
116.7
115.6
115.5
107.2
107.0
45.5
160
150
140
130
120
110
100
90
80
70
60
50
40
30
20
10
0
ppm

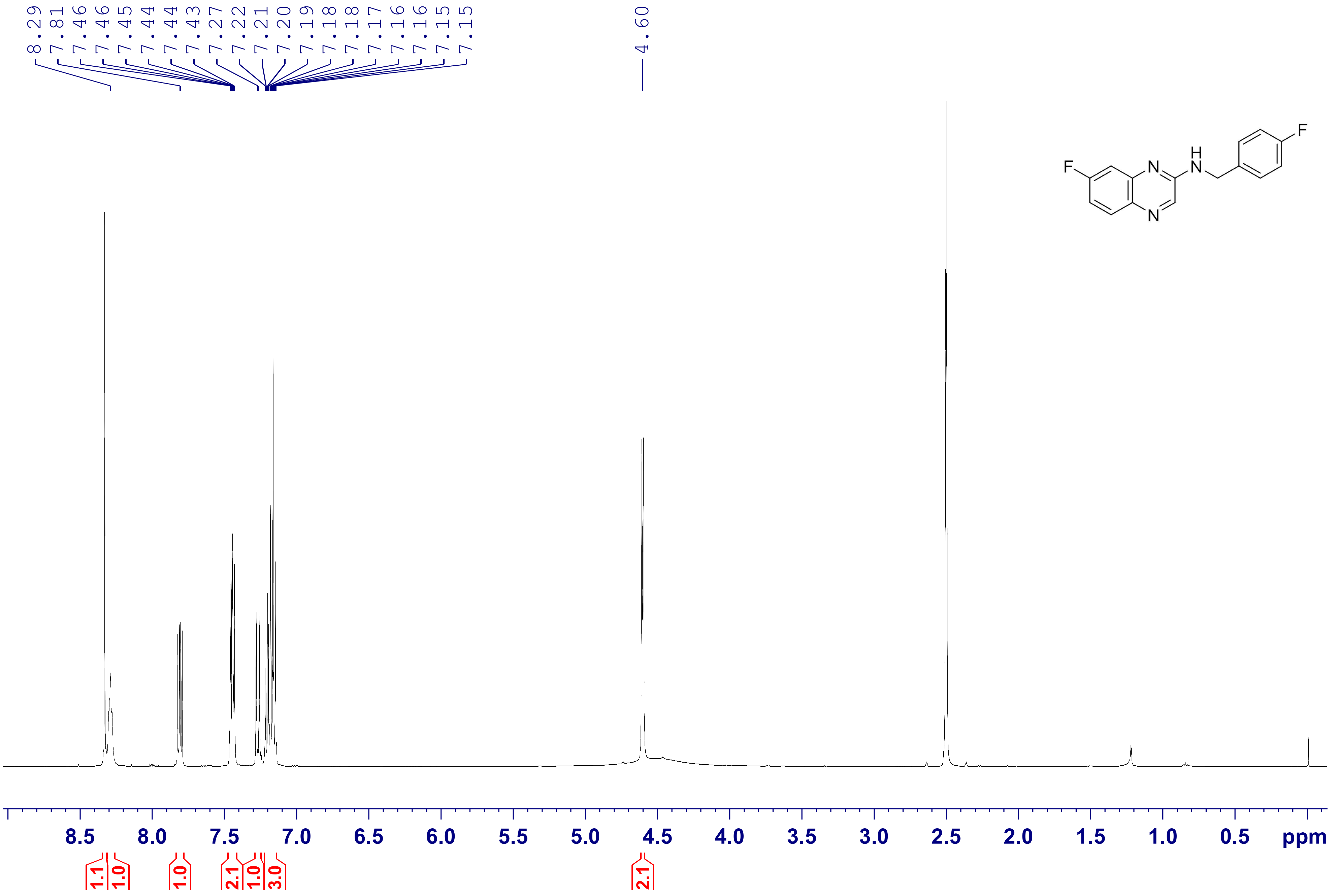

8.29
7.81
7.46
7.46
7.45
7.44
7.44
7.43
7.27
7.22
7.21
7.20
7.19
7.18
7.18
7.17
7.16
7.16
7.15
7.15
4.60
F
N
N
H
N
F
8.5
8.0
7.5
7.0
6.5
6.0
5.5
5.0
4.5
4.0
3.5
3.0
2.5
2.0
1.5
1.0
0.5
ppm
1.1
1.0
1.0
2.1
1.0
3.0
2.1

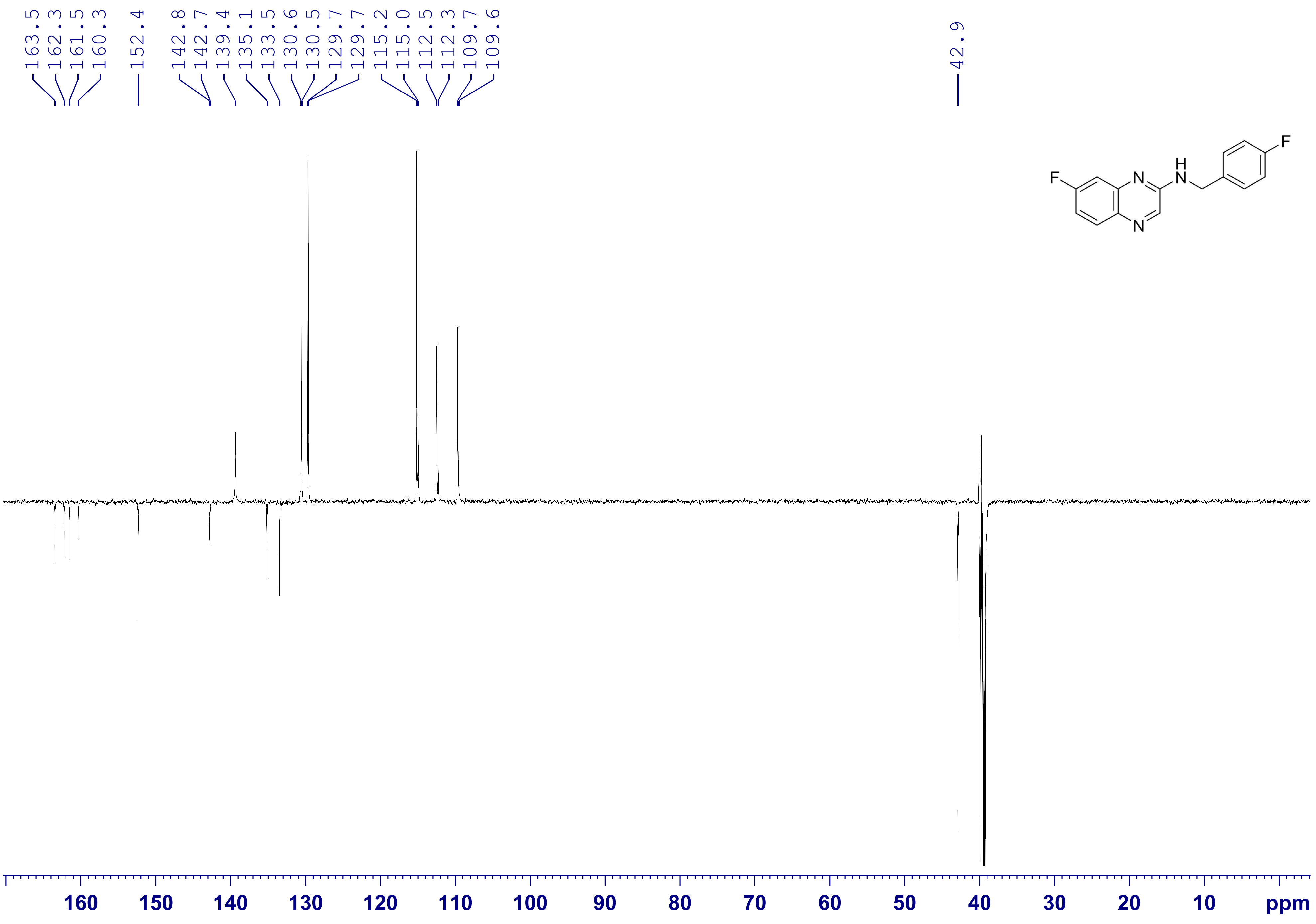
163.5
162.3
161.5
160.3
152.4
142.8
142.7
139.4
135.1
133.5
130.6
130.5
129.7
129.7
115.2
115.0
112.5
112.3
109.7
109.6
42.9
F
N
H
N
F
N
160
150
140
130
120
110
100
90
80
70
60
50
40
30
20
10
ppm
